# Synthesis of Trifluoromethylated Pyrimido[1,2-*b*]indazole Derivatives through the Cyclocondensation of 3-Aminoindazoles with Ketoester and Their Functionalization via Suzuki-Miyaura Cross-Coupling and SN_Ar_ Reactions

**DOI:** 10.3390/molecules29010044

**Published:** 2023-12-20

**Authors:** Sakina Tellal, Badr Jismy, Djamila Hikem-Oukacha, Mohamed Abarbri

**Affiliations:** 1 Laboratoire de Physico-Chimie des Matériaux et des Electrolytes pour l’Energie (PCM2E), EA 6299, Avenue Monge Faculté des Sciences, Parc de Grandmont, 37200 Tours, France; sakinatellal26@gmail.com; 2 Laboratory of Physics and Chemistry Materials LPCM, Department of Chemistry, Faculty of Sciences, University Mouloud Mammeri, Tizi-Ouzou 15000, Algeria; djamilaoukacha@gmail.com

**Keywords:** 3-aminoindazole derivatives, ethyl 4,4,4-trifluoro 3-oxobutanoate, Suzuki-Miyaura cross-coupling, SN_Ar_, trifluoromethylated pyrimido[1,2-*b*]indazole derivatives

## Abstract

A new series of trifluoromethylated pyrimido[1,2-*b*]indazol-4(1*H*)-one derivatives was synthesized with good to excellent yields through a simple condensation of 3-aminoindazole derivatives with ethyl 4,4,4-trifluoro 3-oxobutanoate. The functionalization of the corresponding chlorinated fused tricyclic scaffolds via Suzuki-Miyaura and aromatic nucleophilic substitution reactions led to the synthesis of highly diverse trifluoromethylated pyrimido[1,2-*b*]indazole derivatives with good yields.

## 1. Introduction

**Figure 1:**
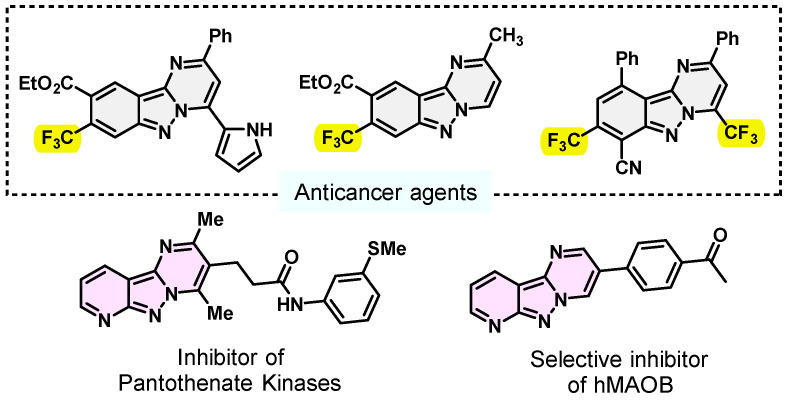
Some representatively bioactive pyrimido[1,2-*b*]indazole derivatives.

Nitrogen-containing heterocycles are of great and immense research interest due to their highly chemical, biological and pharmaceutical significance [1,2,3,4,5,6]. They play an essential role in natural and synthetic organic chemistry [7]. Among them, pyrimido[1,2-*b*]indazoles are known to exhibit a wide range of prominent biological and pharmaceutical activities, such as anticancer [8,9], MAO-B [10] and antibacterial [11] activity, as well as the inhibition of phosphodiesterase 10A (PDE10A) [2] and pantothenate kinases [12] (Figure 1).

**Figure 2:**
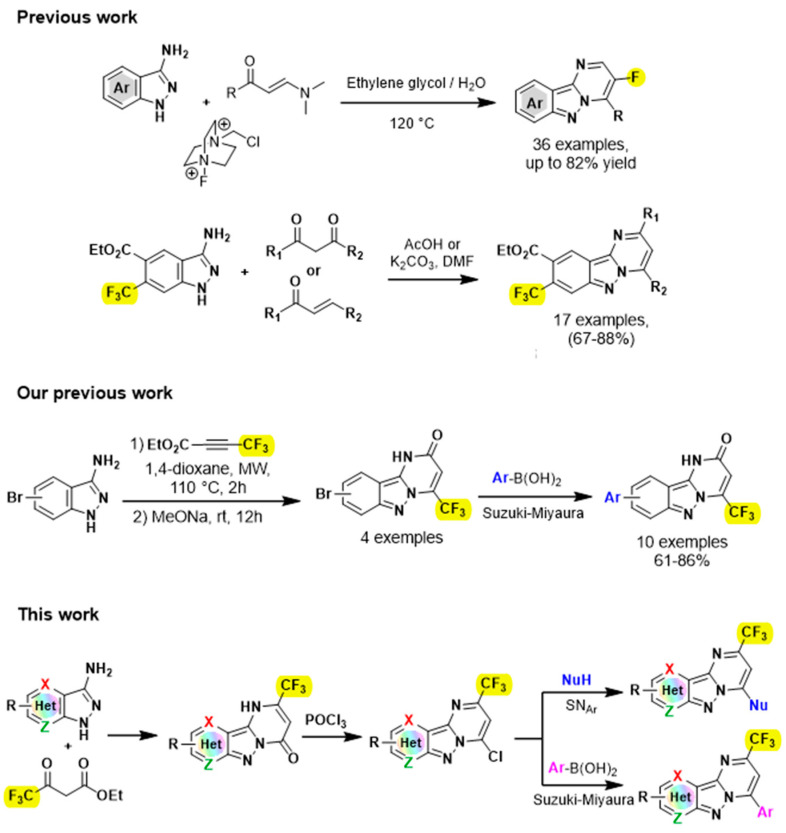
Convergent approach for the synthesis of fluorinated pyrimido[1,2-*b*]indazole derivatives [9,25,26].

Unfortunately, despite their importance, a limited number of syntheses for the construction of these fused pyrimido[1,2-*b*]indazoles have been reported. A well-known procedure for the synthesis of this rigid tricyclic *N*-fused moiety is cyclocondensation between 3-aminoindazoles and carbonyl compounds [13,14,15,16,17]. For example, Song’s group reported BF_3_.Et_2_O-promoted intermolecular cyclocondensation between 3-aminoindazoles and 3-ethoxycyclobutanones [17]. Gao and his group developed a three-component reaction mediated by NH_4_I between 3-aminoindazoles, aromatic aldehydes and ethylamines [18]. Other excellent approaches were developed by Cao’s group by performing either cascade cyclization reactions for the synthesis of a series of chalcogens facilitated by pyrimido[1,2-*b*]indazoles [19], or by condensing the 3-aminoindazoles on the ynals [20]. Surprisingly, despite the pharmaceutical and synthetic importance of fluorine-containing heterocycles [21,22,23,24], methods for synthesizing fluorinated pyrimido[1,2-*b*]indazoles remain scarce. Only a few publications dealing with the synthesis of fluorinated pyrimido[1,2-*b*]indazoles has been reported recently in the literature (Figure 2) [9,25], notably by Wang’s group who disclosed an interesting multicomponent reaction of enaminones, 3-aminoindazoles and selectfluor for the synthesis of fluorinated pyrimido[1,2-*b*]indazoles [25]. Therefore, the development of an efficient and facile method to approach fluorinated tricyclic compounds is highly desirable.

As part of our on-going effort in the synthesis of novel fluorinated heterocycles [27,28,29,30,31], we report herein a convenient and efficient synthetic strategy that enables the construction of trifluoromethylated pyrimido[1,2-*b*]indazole derivatives.

## 2. Results and Discussion

Our strategy started with the gram-scale synthesis of 2-(trifluoromethyl)pyrimido[1,2-*b*]indazol-4(1*H*)-one derivatives **2** by cyclocondensation of 3-aminoindazole derivatives **1** on ethyl 4,4,4-trifluoroacetoacetate. The starting materials **1a–h** were synthesized according to well-known procedure [30,31]. The reaction between 3-aminoindazole derivatives **1** and ethyl 4,4,4-trifluoroacetoacetate was carried out in a MeOH/H_3_PO_4_ mixture (4/1) at reflux for 24 h. Note that the use of methanol as the sole solvent led to the formation of the desired product **2a** with 27% of yield, but unfortunately with only 36% conversion of the starting material **1a** after 72 h. A mixture of methanol/AcOH (4/1) was also screened but was found to be ineffective, giving the expected product **2a** in a yield of 39% with a partial conversion of 78% after 24 h.

**Scheme 1:**
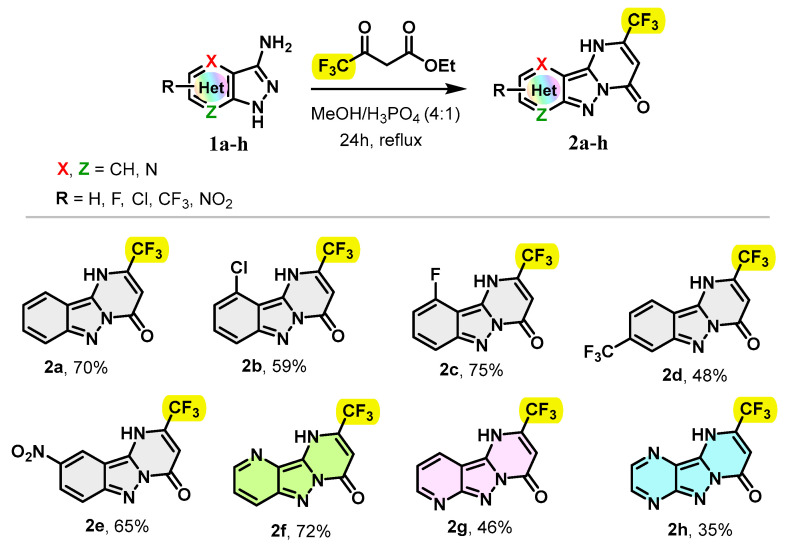
Scope of 3-aminoindazole derivatives.

Under the conditions indicated above, substituted 3-amino-1*H*-pyrazolo[4,3-*b*]pyridine, 3-amino-1*H*-pyrazolo[3,4-*b*]pyridine and 3-amino-1*H*-pyrazolo[3,4-*b*]pyrazine were successfully condensed and yielded regioselectively the trifluoromethylated pyrimido[1,2-*b*]indazol-4(1*H*)-one derivatives **2f–h** with yields ranging from 35 to 75% (Scheme 1). Overall, the 3-amino indazole derivatives were found to exhibit comparable reactivities toward ethyl 4,4,4-trifluoro-3-oxobutanoate, except in the case of 3-amino-1*H*-pyrazolo[3,4-*b*]pyrazine which afforded the cyclocondensation product **2h** with a yield of 35%. Furthermore, the presence of the halo groups, including the chloro-, fluoro- and trifluoromethyl groups, in substrates **1** was compatible under this synthetic protocol, affording the desired tricyclic compounds **2b–d** with yields of 59, 75 and 48%, respectively, while, 3-aminoindazole with the strongly electron-withdrawing nitro group was condensed to generate the targeted product **2e** in 65% yield.

**Scheme 2:**
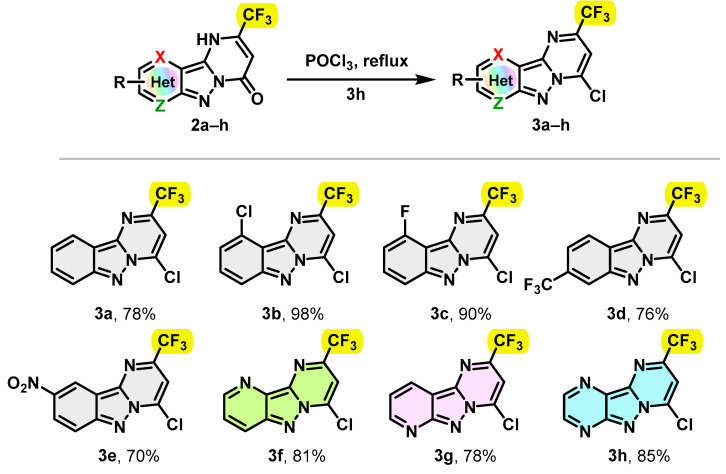
Synthesis of trifluoromethylated 4-chloro-2-(trifluoromethyl)pyrimido[1,2-*b*]indazole derivatives **3a–h**.

In the next step, some 2-trifluromethyl-1*H*-pyrimido[1,2-*b*]indazole-4-one derivatives **2** were converted into the corresponding 4-chloro-2-(trifluoromethyl)pyrimido[1,2-*b*]indazole derivatives **3a–h** in gram-scale synthesis. The reactions were performed in POCl_3_ at reflux. The conversion was found to be complete after 3 h, providing the desired chlorinated products **3a–h** in good to excellent yields. Notably, the unsubstituted 4-chloro-2-(trifluoromethyl)pyrimido[1,2-*b*]indazole derivatives **3a** and **3f–h** were successfully isolated with yields ranging from 78 to 85%. Similarly, 4-chloro-2-(trifluoromethyl)pyrimido[1,2-*b*]indazole derivatives **3b–e** substituted with withdrawing groups such as Cl, F, CF_3_ and NO_2_ were easily accessible with yields of 98, 90, 76 and 70%, respectively. The results are summarized in Scheme 2.

**Scheme 3:**
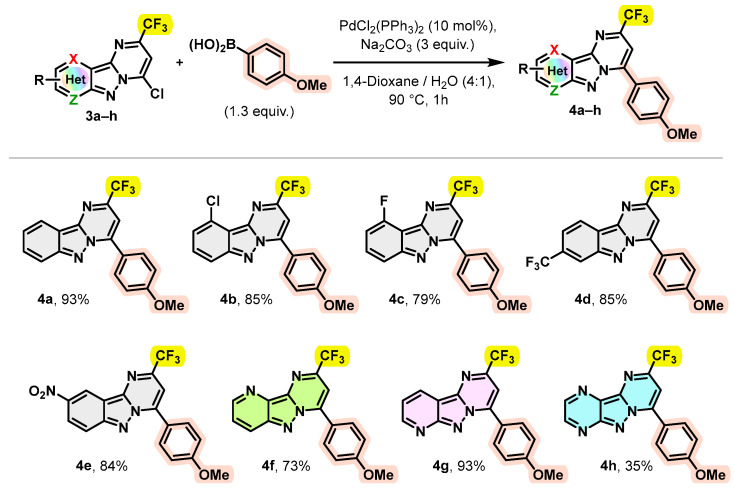
Behavior of 4-chloro-2-(trifluoromethyl)pyrimido[1,2-*b*]indazole derivatives **3a–h** in the Suzuki–Miyaura cross coupling.

Subsequently, compounds **3a–h** were used as building blocks to generate a novel chemical library of substituted pyrimido[1,2-*b*]indazole derivatives. As products, **3a–h** carry a reactive carbon–chloride bond in position C-4, it seemed important to us to test the reactivity of this bond in order to achieve further transformations. Hence, the chlorine at the C-4 position of **3a–h** was subjected to Suzuki–Miyaura cross-coupling using 4-methoxyphenylboronic acid and typical conditions [32,33,34], i.e., PdCl_2_(PPh_3_)_2_ (10 mol%) as catalyst, Na_2_CO_3_ (3 equivalents) as base in dioxane/H_2_O (4:1) at 90 °C for 1 h. The synthetic scope of this coupling reaction was examined using a variety of trifluoromethylated pyrimido[1,2-*b*]indazole derivatives **3a–h**. The results are summarized in Scheme 3.

As shown in Scheme 3, a number of trifluoromethylated pyrimido[1,2-*b*]indazoles and pyrido[2′,3′:3,4]pyrazolo[1,5-*a*]pyrimidines **3a–g** were efficiently arylated at position 4 by 4-methoxy phenyl, leading to the desired products **4a–g** with excellent yields (>73%). However, this arylation appeared to be less effective in the case of trifluoromethylated pyrazino[2′,3′:3,4]pyrazolo[1,5-*a*]pyrimidine **3h,** since the expected product **4h** was isolated with a yield not exceeding 35%. We believe that complexation of boronic acid with a polynitrogen system would reduce the yield by inactivation of the boronic acid.

**Scheme 4:**
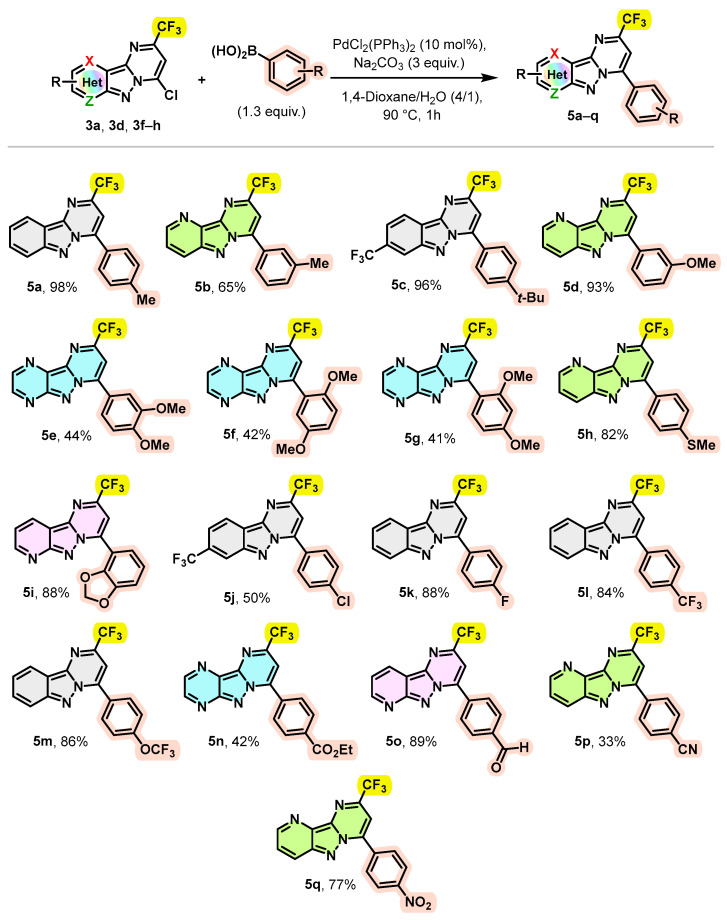
Scope of the Suzuki–Miyaura cross-coupling.

Encouraged by these results, we explored the scope of this coupling reaction using a wide variety of commercially available arylboronic acids to access new trifluoromethylated pyrimido[1,2-*b*]indazole derivatives with a high structural diversity. The obtained results are summarized in Scheme 4.

As illustrated in Scheme 4, different boronic acids bearing electron-donating or electron-withdrawing groups on the aromatic ring provided the arylated products **5a–q** in good to excellent yields. Notably, 4-methyl and 3-methylphenylboronic acids were successfully coupled with compounds **3a** and **3f** leading to the arylation products **5a** and **5b** with yields of 98 and 65%, respectively. Likewise, phenyl boronic acid with a *tert*-butyl substituent at the *para* position also smoothly underwent reaction with **3d**, giving the desired product **5c** in a 96% yield. Arylboronic acids bearing strong electron-donating groups, such as a methoxy group in the *meta*-position or a thiomethyl group in the *para*-position, were readily coupled with **3f** to provide the corresponding products **5d** (93%) and **5h** (82%), respectively. As observed previously, trifluoromethylated pyrazino[2′,3′:3,4]pyrazolo[1,5-*a*]pyrimidine **3h** afforded poorer yields again when it was engaged in a Suzuki–Miyaura coupling reaction; the coupling of compound **3h** with 3,4-, 2,5- and 2,4-dimethoxyphenylboronic acids provided the desired arylation products **5e g** in yields of only 44%, 42% and 41%, respectively. These results seem to indicate that steric hindrance does not play a significant role in the efficiency of this coupling reaction. Moreover, the coupling reaction of compound **3g** with 1,3-benzodioxol-4-ylboronic acid was easily converted to the desired product **5i** with a yield of 88%. Likewise, phenylboronic acid substituted at the *para* position with an electron-withdrawing group such as Cl, F, CF_3_, OCF_3_, CO_2_Et, CHO, CN or NO_2_ provided the expected products **5j–q** with yields ranging from 33% to 89%.

**Scheme 5:**
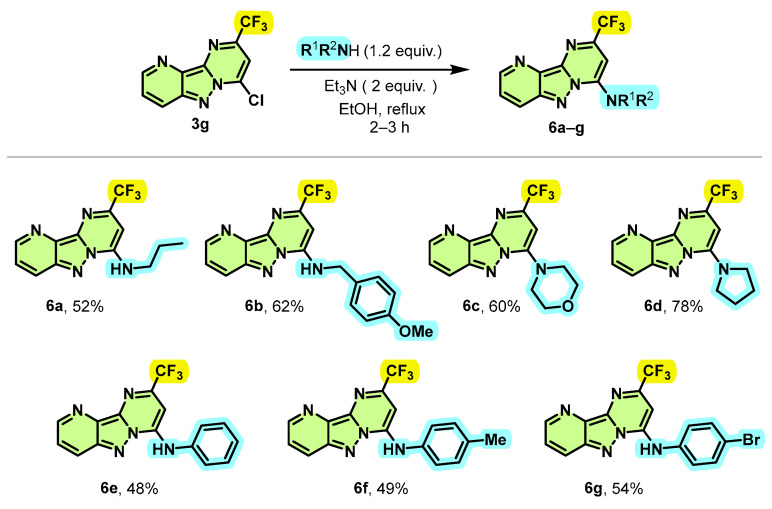
Synthesis route of 4-amino-2-trifluoromethyl pyrimido[1,2-*b*]indazole derivatives **6a–g**.

Given the success of this Suzuki–Miyaura coupling reaction allowing the introduction of aryl groups at position 4 of 2-(trifluoromethyl)pyrimido[1,2-*b*]indazole derivatives, and in order to introduce more functional diversity into these compounds, we therefore focused our investigations synthesizing new trifluoromethylated pyrimido[1,2-*b*]indazole derivatives by aromatic nucleophilic substitution (SN_Ar_) displacement of the chloride moiety. To validate the feasibility of our hypothesis, we first sought to test the amination of compound **3g** by using morpholine as the model substrate. Initially, when the reaction was carried out under reflux of EtOH in the absence of base, the desired compound **6c** was obtained in 12% of yield with a very low conversion of 25% after 24 h. The desired product **6c** was obtained this time in 31% of the yield with a conversion of 67% after 12 h when equivalent 1 of Et_3_N was used as a base. To our delight, we discovered that equivalent 2 of Et_3_N enables the total conversion of the starting material **3g** into **6c** with a yield of 60% after 2 h. Another base, such as K_2_CO_3_, was also effective for the reaction, but less efficient than Et_3_N, providing **6c** in 55% yield after 2 h. Various amines were incorporated at the C-4 position of substrate **3f**, as a representative example of chlorinated products, to provide the expected products **6a–g** in moderate to good yields (Scheme 5). Primary amines such as *n*-propylamine and benzylamine reacted effectively to give the aminated products **6a** and **6b** with yields of 52% and 62%, respectively. This reaction was also extended to secondary amines, such as morpholine and pyrrolidine, leading to the new trifluoromethylated pyrimido[1,2-*b*]indazole derivatives **6c** and **6d** in yields of 60% and 78%, respectively.

Interestingly, this SN_Ar_ reaction proved to be compatible with aromatic amines, since unsubstituted or substituted aniline by electron-donating or electron-withdrawing groups such as methyl and bromine led to *N*-arylation products **6e–g** with acceptable yields (48% to 54%).

**Scheme 6:**
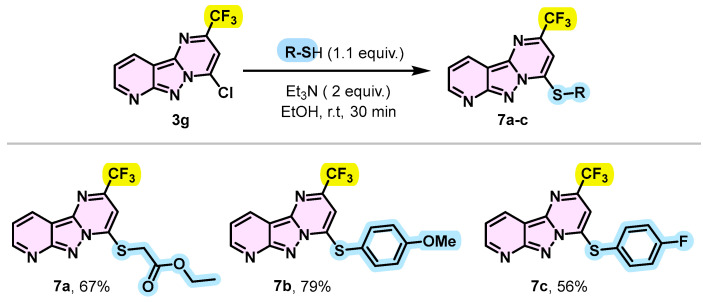
Synthesis route of 4-(alkylthio or arylthiol)-2-(trifluoromethyl)pyrido[2′,3′:3,4]pyrazolo[1,5-*a*]pyrimidine **7a–c**.

To demonstrate other practical applications of SN_Ar,_ an alkylthiol such as ethyl 2-mercapto acetate was found to be reactive towards 4-chloro-2-(trifluoromethy)pyrido[2′,3′;3,4]pyrazolo[1,5-*a*]pyrimidine **3g**, providing the desired thiolation product **7a** in 67% yield. The reaction was carried out in the presence of triethylamine (2 equivalents) in ethanol (EtOH) at room temperature for 30 min. Under the same conditions, thiophenols were also successfully introduced into the C-4 position. In fact, thiophenol substituted by an electron-donating group such as methoxy or an electron-withdrawing group such as fluorine led to the expected products **7b** and **7c** with respective yields of 79% and 56% (Scheme 6). This observed difference in yields could be explained by the difference in nucleophilicity of the thiols caused by the nature of the substituents on the aromatic ring.

**Scheme 7:**
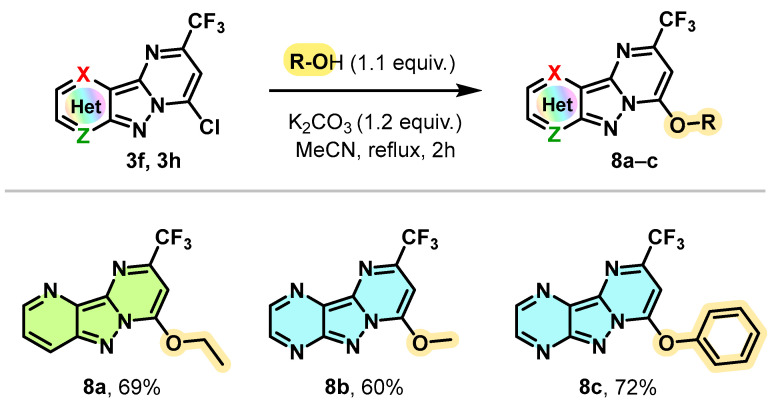
Synthesis of 7-alkoxy (or 7-phenoxy)-2-trifluoromethyl pyrimido[1,2-*b*]indazole derivatives **7a–c**.

However, under the same conditions, alcohols and phenol showed no reactivity in the SN_Ar_ reaction (Scheme 7). Under modified conditions and operating this time in the presence of K_2_CO_3_ as a base (1.2 equivalent) at the reflux of acetonitrile for 2 h, EtOH and methanol reacted efficiently with the chlorinated derivatives **3f** and **3h** to provide the O-alkylation products **8a** and **8b** with yields of 69% and 60%, respectively. The conditions used were also compatible with the use of phenol as a nucleophile by allowing the O-arylation of compound **3h** with a yield of 72%.

The flexibility of our strategy allows the synthesis of an original library of trifluoromethylated pyrimido[1,2-*b*]indazole derivatives with a large substrate scope and with reasonable to good yields (46 examples). All the synthesized trifluoromethylated pyrimido[1,2-*b*]indazole derivatives are new and have been fully characterized by ^1^H, ^13^C, ^19^F NMR spectroscopy and HRMS (Supplementary Materials).

## 3. Materials and Methods

### 3.1. General Methods

The reagents used were purchased from commercial suppliers and were used without further purification. The following solvents were distilled as follows: Triethylamine and MeCN on calcium hydride, and 1,4-dioxane on sodium and benzophenone. The reactions were monitored by thin layer chromatography (TLC) analysis using silica gel plates (60 F254). Compounds were visualized by long-wave (365 nm) or short-wave (254 nm) UV light. Purification of the products by column chromatography was carried out using silica gel 60 (230 to 400 mesh, 0.040 to 0.063 mm)

All ^1^H NMR, ^13^C NMR and ^19^F NMR spectra were recorded with a Bruker Avance FT-NMR spectrometer at 300 MHz (300 MHz, 75 MHz or 282 MHz, respectively). Chemical shifts are reported in ppm and tetramethylsilane (TMS) is used as an internal standard. 1H NMR assignment abbreviations are given as follows: singlet(s), doublet (d), triplet (t), quartet (q), wide singlet (br s), doublet of doublets (dd), triplet of doublets (td), doublet of a triplet (dt) and a multiplet (m). Coupling constants (J) are expressed in Hertz (Hz). High-resolution electrospray ionization mass spectrometry experiments were performed with a hybrid tandem quadrupole/time-of-flight (Q-TOF) instrument, equipped with a pneumatically assisted electrospray (Zspray) ion source (Micromass, Manchester, United Kingdom) operating in positive mode. The melting points were determined on a Kofler Bench apparatus.

### 3.2. General Procedure for the Synthesis of 2-Trifluromethyl-1H-pyrimido[1,2-b]indazole-4-one Derivatives ***2a–h***

To a mixture of aminoindazole derivative **1** (2 g, 1 equiv.) and ethyl 4,4,4-trifluoroacetoacetate (2 equiv.) in 4 mL of dry methanol was added dropwise 1 mL of polyphosphoric acid (H_3_PO_4_). The solution was refluxed for 24 h under Argon. The progress of the reaction was monitored by TLC (eluent: petroleum ether/ethyl acetate, 5:5). After cooling to room temperature, the solvent was removed under reduced pressure. The crude solid was suspended in a small quantity of water. The resulting precipitate was collected by filtration and washed with Et_2_O to provide the pure desired product. This procedure was employed to prepare all the 2-trifluromethyl-1*H*-pyrimido[1,2-*b*]indazole-4-one derivatives **2a–h**. (^1^H-NMR, ^19^F-NMR and ^13^C-NMR of compounds **2a–h** are shown in Supplementary Materials).

2-Trifluoromethyl-1*H*-pyrimido[1,2-*b*]indazol-4-one **(2a)**.

Compound **2a** was obtained as a white solid with a yield of 70%; m.p. 274–276 °C; ^1^H NMR (300 MHz, DMSO-*d*_6_): *δ* 8.20 (d, *J* = 8.1 Hz, 1H), 7.81 (t, *J* = 7.5 Hz, 1H), 7.58 (d, *J* = 8.1 Hz, 1H), 7.39 (t, *J* = 7.5 Hz, 1H), 6.77 (s, 1H); ^13^C NMR (75 MHz, DMSO-*d*_6_): *δ* 154.4, 149.8 (q, *J* = 33.8 Hz), 147.3, 141.7, 133.9, 122.9, 122.8, 121.8 (q, *J* = 273.5 Hz), 115.2, 111.6, 101.0 (q, *J* = 2.8 Hz); ^19^F NMR (282 MHz, DMSO-*d*_6_): *δ* −67.54; HRMS (ESI) *m*/*z* [M+H]^+^ calcd for **C_11_H_7_F_3_N_3_O**: 254.0535; found: 254.0531.

10-Chloro-2-trifluoromethyl-1*H*-pyrimido[1,2-*b*]indazol-4-one **(2b)**.

Compound **2b** was obtained as a white solid with a yield of 59%; m.p. 289–291 °C; ^1^H NMR (300 MHz, DMSO-*d*_6_): *δ* 7.75 (t, *J* = 7.5 Hz, 1H), 7.54 (d, *J* = 8.4 Hz, 1H), 7.58 (d, *J* = 8.4 Hz, 1H), 6.81 (s, 1H); ^13^C NMR (75 MHz, DMSO-*d*_6_): *δ* 154.2, 149.8 (q, *J* = 33,8 Hz), 146.1, 143.0, 134.3, 128.8, 123.0, 121.8 (q, *J* = 273.5 Hz), 112.5, 110.4, 101.3 (q, *J* = 2.7 Hz); ^19^F NMR (282 MHz, DMSO-*d*_6_): *δ* −67.47; HRMS (ESI) *m*/*z* [M+H]^+^ calcd for **C_11_H_6_ClF_3_N_3_O**: 288.0146; found: 288.0141.

10-Fluoro-2-trifluoromethyl-1*H*-pyrimido[1,2-*b*]indazol-4-one **(2c)**.

Compound **2c** was obtained as a beige powder with a yield of 75%; m.p. 304–306 °C; ^1^H NMR (300 MHz, DMSO-*d*_6_): *δ* 7.79 (dd, *J* = 12.9, 7.8 Hz, 1H), 7.40 (d, *J* = 8.4 Hz, 1H), 7.16 (dd, *J* = 12.9, 7.8 Hz, 1H), 6.80 (s, 1H); ^13^C NMR (75 MHz, THF-*d*_8_): *δ* 158.0 (d, *J* = 258.4 Hz), 153.4, 151.1 (q, *J* = 34.4 Hz), 145.7 (d, *J* = 3.7 Hz), 143.2 (d, *J* = 6.3 Hz), 134.6 (d, *J* = 8.5 Hz), 121.4 (q, *J* = 273.1 Hz), 107.3 (d, *J* = 17.7 Hz), 106.3 (d, *J* = 4.7 Hz), 105.6 (d, *J* = 19.2 Hz), 101.4 (q, *J* = 3.0 Hz); ^19^F NMR (282 MHz, DMSO-*d*_6_): *δ* −67.47, −115.32; HRMS (ESI) *m*/*z* [M+H]^+^ calcd for **C_11_H_6_ F_4_N_3_O**: 272.0441; found: 272.0436.

2,8-Bis-trifluoromethyl-1*H*-pyrimido[1,2-*b*]indazol-4-one **(2d)**.

Compound **2d** was obtained as a white crystal with a yield of 48%; m.p. 277–279 °C; ^1^H NMR (300 MHz, DMSO-*d*_6_): *δ* 8.46 (d, *J* = 8.4 Hz, 1H), 7.89 (s, 1H), 7.62 (d, *J* = 8.4 Hz, 1H), 6.83 (s, 1H); ^13^C NMR (75 MHz, THF-*d*_8_): *δ* 153.6, 151.0 (q, *J* = 34.4 Hz), 147.3, 140.8, 134.3 (q, *J* = 32.3 Hz), 124.2, 123.9 (q, *J* = 271.2 Hz), 121.4 (q, *J* = 273.1 Hz), 118.6, 118.5 (q, *J* = 3.4 Hz), 108.2 (q, *J* = 4.4 Hz), 102.2 (q, *J* = 2.9 Hz); ^19^F NMR (282 MHz, DMSO-*d*_6_): *δ* −61.07, −67.38; HRMS (ESI) *m*/*z* [M+H]^+^ calcd for **C_12_H_6_F_6_N_3_O**: 322.0409; found: 322.0403.

9-Nitro-2-trifluoromethyl-1*H*-pyrimido[1,2-*b*]indazol-4-one **(2e)**.

Compound **2e** was obtained as a yellow solid with a yield of 65%; m.p. 300–302 °C; ^1^H NMR (300 MHz, DMSO-*d*_6_): *δ* 8.93 (s, 1H), 8.43 (d, *J* = 9.0 Hz, 1H), 7.66 (d, *J* = 9.0 Hz, 1H), 6.76 (s, 1H); ^13^C NMR (75 MHz, THF-*d*_8_): *δ* 153.6, 151.0 (q, *J* = 34.5 Hz), 147.9, 143.4, 143.0, 127.9, 121.4 (q, *J* = 273.1 Hz), 119.8, 115.5, 111.0, 102.7 (q, *J* = 3.0 Hz); ^19^F NMR (282 MHz, DMSO-*d*_6_): *δ* −67.40; HRMS (ESI) *m*/*z* [M+H]^+^ calcd for **C_11_H_6_F_3_N_4_O_3_**: 299.0386; found: 299.0382.

9-(Trifluoromethyl)pyrido[3′,2′:3,4]pyrazolo[1,5-*a*]pyrimidin-7(10*H*)-one **(2f)**.

Compound **2f** was obtained as a yellow solid with a yield of 72%; m.p. 296–298 °C; ^1^H NMR (300 MHz, DMSO-*d*_6_): *δ* 8.74 (dd, *J* = 4.5, 1.2 Hz, 1H), 8.23 (dd, *J* = 8.7, 1.2 Hz, 1H), 7.82 (dd, *J* = 8.7, 4.5 Hz, 1H), 6.77 (s, 1H); ^13^C NMR (75 MHz, DMSO): *δ* 155.0, 149.2 (q*, J* = 35.5 Hz), 145.5, 145.2, 138.2, 126.9, 123.3, 123.3, 122.0 (q*, J* = 273.1 Hz), 100.5 (q*, J* = 2.1 Hz); ^19^F NMR (282 MHz, DMSO-*d*_6_): *δ* −67.37; HRMS (ESI) *m*/*z* [M+H]^+^ calcd for **C_10_H_6_F_3_N_4_O**: 255.0488; found: 255.0484.

2-(Trifluoromethyl)pyrido[2′,3′:3,4]pyrazolo[1,5-*a*]pyrimidin-4(1*H*)-one **(2g)**.

Compound **2g** was obtained as an orange-yellow solid with a yield of 46%; m.p. 366–368 °C; ^1^H NMR (300 MHz, DMSO-*d*_6_): *δ* 9.04 (dd, *J* = 7.8, 0.9 Hz, 1H), 8.79 (dd, *J* = 5.7, 0.9 Hz, 1H), 7.28 (dd, *J* = 7.8, 5.7 Hz, 1H), 6.65 (s, 1H); ^13^C NMR (75 MHz, CDCl_3_/TFA-*d*_1_): *δ* 157.6, 148.3 (q, *J* = 36.2 Hz), 145.3, 144.6, 143.5, 135.6, 130.3, 120.1 (q, *J* = 274.4 Hz), 114.9, 102.6 (q, *J* = 2.1 Hz); ^19^F NMR (282 MHz, DMSO-*d*_6_): *δ* −67.25; HRMS (ESI) *m*/*z* [M+H]^+^ calcd for **C_10_H_6_F_3_N_4_O**: 255.0488; found: 255.0483.

9-(Trifluoromethyl)pyrazino[2′,3′:3,4]pyrazolo[1,5-*a*]pyrimidin-7(10*H*)-one **(2h)**.

Compound **2h** was obtained as a red solid with a yield of 35%; m.p. 261–263 °C; ^1^H NMR (300 MHz, DMSO-*d*_6_): *δ* 8.83 (d, *J* = 2.1 Hz, 1H), 8.69 (d, *J* = 2.1 Hz, 1H), 6.75 (s, 1H); ^13^C NMR (75 MHz, THF-*d*_8_): *δ* 153.2, 148.3, 147.4, 145.8, 141.9, 127.5, 124.4, 121.4 (q*, J* = 273.1 Hz), 103.6 (q, *J* = 2.8 Hz); ^19^F NMR (282 MHz, DMSO-*d*_6_): δ −67.32; HRMS (ESI) *m*/*z* [M+H]^+^ calcd for **C_9_H_5_F_3_N_5_O**: 256.0440; found: 256.0436.

### 3.3. General Procedure for the Synthesis of 4-Chloro-2-trifluromethyl-1H-pyrimido[1,2-b]indazole-4-one Derivatives ***3a–h***

A solution of 2-trifluromethyl-1*H*-pyrimido[1,2-*b*]indazole-4-one derivative **2** (2 g, 0.005 mol) and phosphorus oxychloride (POCl_3_) (0.107 mol) was heated under reflux for 3 h. The progress of the reaction was monitored by TLC (eluent: petroleum ether/ethyl ac-etate, 7:3). The reaction mixture was brought to room temperature and the excess of POCl_3_ was removed in vacuo. Water was added and the chlorinated product was extracted with dichloromethane. The organic layer was separated, dried over anhydrous MgSO_4_ and filtered. The filtrate was concentrated and purified by column chromatography on silica gel to provide the desired compounds **3a–h**. (^1^H-NMR, ^19^F-NMR and ^13^C-NMR of compounds **3a–h** are shown in Supplementary Materials).

4-Chloro-2-(trifluoromethyl)pyrimido[1,2-*b*]indazole **(3a)**.

The purification of the crude product using chromatography on silica gel was carried out using (PE/EtOAc: 9.6/0.4) to afford **3a** as a yellow solid with a yield of 78%; m.p. 165–167 °C; ^1^H NMR (300 MHz, CDCl_3_): *δ* 8.43 (d, *J* = 8.4 Hz, 1H), 8.05 (d, *J* = 8.4 Hz, 1H), 7.80 (td, *J* = 8.7, 1.2 Hz, 1H), 7.74 (s, 1H), 7.51 (td, *J* = 8.7, 1.2 Hz, 1H); ^13^C NMR (75 MHz, CDCl_3_): *δ* 152.1, 143.7, 141.3 (q, *J* = 37.2 Hz), 137.7, 131.5, 123.4, 121.3, 120.6 (q, *J* = 273.1 Hz), 117.3, 114.9, 107.9 (q, *J* = 2.4 Hz); ^19^F NMR (282 MHz, CDCl_3_): *δ* −66.78; HRMS (ESI) *m*/*z* [M+H]^+^ calcd for **C_11_H_6_ClF_3_N_3_**: 272.0196; found: 272.0192.

4,10-Dichloro-2-(trifluoromethyl)pyrimido[1,2-*b*]indazole **(3b)**.

The purification of the crude product using chromatography on silica gel was carried out using (PE/EtOAc: 9/1) to afford **3b** as a yellow solid with a yield of 98%; m.p. 136–138 °C; ^1^H NMR (300 MHz, CDCl_3_): *δ* 7.94 (dd, *J* = 8.7, 0.6 Hz, 1H), 7.79 (s, 1H), 7.69 (dd, *J* = 8.7, 7.2 Hz, 1H), 7.47 (dd, *J* = 7.2, 0.6 Hz, 1H); ^13^C NMR (75 MHz, CDCl_3_): *δ* 152.7, 143.0, 142.2 (q, *J* = 37.1 Hz), 138.0, 131.5, 128.2, 123.6, 120.4 (q, *J* = 273.1 Hz), 115.8, 112.9, 108.5 (q, *J* = 2.3 Hz); ^19^F NMR (282 MHz, CDCl_3_): *δ* −66.98; HRMS (ESI) *m*/*z* [M+H]^+^ calcd for **C_11_H_5_Cl_2_F_3_N_3_**: 305.9807; found: 305.9801.

4-Chloro-10-fluoro-2-(trifluoromethyl)pyrimido[1,2-*b*]indazole **(3c)**.

The purification of the crude product using chromatography on silica gel was carried out using (PE/EtOAc: 8/2) to afford **3c** as a yellow solid with a yield of 90%; m.p. 138–140 °C; ^1^H NMR (300 MHz, CDCl_3_): *δ* 7.84 (d, *J* = 8.4 Hz, 1H), 7.79 (s, 1H), 7.77–7.20 (m, 1H), 7.13 (dd, *J* = 9.6, 7.5 Hz, 1H); ^13^C NMR (75 MHz, CDCl_3_): *δ* 156.6 (d, *J* = 258.7 Hz), 153.3 (d, *J* = 4.0 Hz), 142.4 (q, *J* = 37.6 Hz), 141.9 (d, *J* = 5.2 Hz), 138.0, 132.0 (d, *J* = 7.6 Hz), 120.4 (q, *J* = 273.2 Hz), 113.2 (d, *J* = 4.9 Hz), 108.4 (q, *J* = 2.4 Hz), 107.2 (d, *J* = 17.3 Hz), 105.5 (d, *J* = 18.2 Hz); ^19^F NMR (282 MHz, CDCl_3_): *δ* −66.86, −114.27; HRMS (ESI) *m*/*z* [M+H]^+^ calcd for **C_11_H_5_ClF_4_N_3_**: 290.0102; found: 290.0097.

4-Chloro-2,8-bis-trifluoromethyl-pyrimido[1,2-*b*]indazole **(3d)**.

The purification of the crude product using chromatography on silica gel was carried out using (PE/EtOAc: 9/1) to afford **3d** as a yellow solid with a yield of 76%; m.p. 119–121 °C; ^1^H NMR (300 MHz, CDCl_3_): δ 8.56 (d, *J* = 8.7 Hz, 1H), 8.37 (s, 1H), 7.83 (s, 1H), 7.69 (d, *J* = 8.7 Hz, 1H); ^13^C NMR (75 MHz, CDCl_3_): δ 190.7, 143.5, 142.6 (q, *J* = 37.7 Hz), 138.7, 133.3 (q, *J* = 32.2 Hz), 123.9 (q, *J* = 271.2 Hz), 122.7, 120.4 (q, *J* = 273.2 Hz), 119.2 (q, *J* = 3.0 Hz), 116.3, 115.6 (q, *J* = 4.7 Hz), 109.0 (q, *J* = 2.4 Hz); ^19^F NMR (282 MHz, CDCl_3_): δ −66.61, −66.96; HRMS (ESI) *m*/*z* [M+H]^+^ calcd for **C_12_H_5_ClF_6_N_3_**: 340.0070; found: 340.0065.

4-Chloro-9-nitro-2-(trifluoromethyl)pyrimido[1,2-*b*]indazole **(3e)**.

The purification of the crude product using chromatography on silica gel was carried out using (PE/EtOAc: 9.2/0.8) to afford **3e** as a brown solid with a yield of 70%; m.p. 206–208 °C; ^1^H NMR (300 MHz, CDCl_3_): *δ* 9.41 (dd, *J* = 2.1, 0.6 Hz, 1H), 8.58 (dd, *J* = 9.3, 2.1 Hz, 1H), 8.09 (dd, *J* = 9.3, 0.6 Hz, 1H), 7.90 (s, 1H); ^13^C NMR (75 MHz, CDCl_3_): *δ* 153.1, 145.7, 144.2 (q, *J* = 37.1 Hz), 143.4, 139.8, 125.8, 120.2 (q, *J* = 273.3 Hz), 119.9, 118.3, 113.7, 109.9 (q, *J* = 2.3 Hz); ^19^F NMR (282 MHz, CDCl_3_): *δ* −67.11; HRMS (ESI) *m*/*z* [M+H]^+^ calcd for **C_11_H_5_ClF_3_N_4_O_2_**: 317.0047; found: 317.0041.

7-Chloro-9-(trifluoromethyl)pyrido[3′,2′:3,4]pyrazolo[1,5-*a*]pyrimidine **(3f)**.

The purification of the crude product using chromatography on silica gel was carried out using (PE/EtOAc: 8.4/0.6) to afford **3f** as a green solid with a yield of 81%; m.p. 181–183 °C; ^1^H NMR (300 MHz, DMSO-*d*_6_): *δ* 8.92 (dd, *J* = 4.2, 1.2 Hz, 1H), 8.60 (s, 1H), 8.53 (dd, *J* = 8.7, 1.2 Hz, 1H), 7.81 (dd, *J* = 8.7, 4.2 Hz, 1H); ^13^C NMR (75 MHz, CDCl_3_): *δ* 149.1, 145.9, 143.2 (q, *J* = 37.8 Hz), 143.0, 139.3, 131.0, 125.8, 125.6, 120.3 (q, *J* = 273.5 Hz), 109.2 (q, *J* = 2.3 Hz); ^19^F NMR (282 MHz, DMSO-*d*_6_): *δ* −66.62; HRMS (ESI) *m*/*z* [M+H]^+^ calcd for **C_10_H_5_ClF_3_N_4_**: 273.0149; found: 273.0143.

4-Chloro-2-(trifluoromethyl)pyrido[2′,3′:3,4]pyrazolo[1,5-*a*]pyrimidine **(3g)**.

The purification of the crude product using chromatography on silica gel was carried out using (PE/EtOAc: 8/2) to afford **3g** as a light brown solid with a yield of 78%; m.p. 168–170 °C; ^1^H NMR (300 MHz, DMSO-*d*_6_): *δ* 9.07 (dd, *J* = 4.2, 1.8 Hz, 1H), 8.89 (dd, *J* = 8.4, 1.8 Hz, 1H), 8.62 (s, 1H), 7.54 (dd, *J* = 8.4, 4.2 Hz, 1H); ^13^C NMR (75 MHz, CDCl_3_): *δ* 160.8, 156.0, 142.9 (q, *J* = 37.6 Hz), 142.8, 139.4, 131.2, 120.4 (q, *J* = 273.3 Hz), 119.0, 109.2 (q, *J* = 2.4 Hz), 107.8; ^19^F NMR (282 MHz, DMSO-*d*_6_): *δ* −66.52; HRMS (ESI) *m*/*z* [M+H]^+^ calcd for **C_10_H_5_ClF_3_N_4_**: 273.0149; found: 273.0143.

7-Chloro-9-(trifluoromethyl)pyrazino[2′,3′:3,4]pyrazolo[1,5-*a*]pyrimidine **(3h)**.

The purification of the crude product using chromatography on silica gel was carried out using (PE/EtOAc: 8/2) to afford **3h** as a brown solid with a yield of 85%; m.p. 181–183 °C; ^1^H NMR (300 MHz, DMSO-*d*_6_): *δ* 9.12 (d, *J* = 2.1 Hz, 1H), 8.99 (d, *J* = 2.1 Hz, 1H), 8.76 (s, 1H); ^13^C NMR (75 MHz, CDCl_3_): *δ* 155.0, 149.9, 144.9 (q, *J* = 38.2 Hz), 144.0, 142.4, 140.7, 124.2, 120.1 (q, *J* = 273.8 Hz), 110.4 (q, *J* = 2.2 Hz); ^19^F NMR (282 MHz, DMSO-*d*_6_): *δ* −66.84; HRMS (ESI) *m*/*z* [M+H]^+^ calcd for **C_9_H_4_ClF_3_N_5_**: 274.0101; found: 274.0096.

### 3.4. General Procedure for the Suzuki–Miyaura Cross-Coupling Reaction: Synthesis of 4-Arylated 2-Trifluoromethyl Pyrimido[1,2-b]indazole Derivatives ***4a–h*** and ***5a–q***

A solution of compounds **3** (1.0 equiv.) in the mixture of 1,4-dioxane/H_2_O (4/1) was degassed using argon bubbling. Sodium carbonate (3 equiv.), 4-methoxyphenyl boronic acid (1.3 equiv.) and PdCl_2_(PPh_3_)_2_ (0.1 equiv.) were then added and the reaction mixture was heated to 90 °C for 1h in a sealed tube. After the completion of the reaction monitored using TLC analysis (eluent: petroleum ether/ethyl acetate, 8:2), the solvents were evaporated under reduced pressure, and the crude residue was purified by silica gel column chromatography to provide the desired compounds **4a–h** and **5a–q**. (^1^H-NMR, ^19^F-NMR and ^13^C-NMR of compounds **4a–h** and **5a–q** are shown in the Supplementary Materials).

4-(4-Methoxy-phenyl)-2-(trifluoromethyl)pyrimido[1,2-*b*]indazole **(4a)**.

The purification of the crude product using chromatography on silica gel was carried out using (PE/EtOAc: 9/1) to afford **4a** as an orange solid with a yield of 93%; m.p. 155–157 °C; ^1^H NMR (300 MHz, CDCl_3_): *δ* 8.45 (dt, *J* = 8.4, 0.9 Hz, 1H), 8.33 (d, *J* = 9.0 Hz, 2H), 7.94 (dt, *J* = 8.4, 0.9 Hz, 1H), 7.72 (ddd, *J* = 8.4, 6.9, 1.2 Hz, 1H), 7.64 (s, 1H), 7.43 (ddd, *J* = 8.4, 6.9, 1.2 Hz, 1H), 7.18 (d, *J* = 9.0 Hz, 2H), 3.97 (s, 3H); ^13^C NMR (75 MHz, CDCl_3_): *δ* 162.4, 151.9, 145.6, 144.1, 142.0 (q, *J* = 36.2 Hz), 131.4 (2C), 130.6, 122.7, 122.1, 121.3 (q, *J* = 272.8 Hz), 121.2, 116.9, 114.4 (2C), 114.0, 106.0 (q, *J* = 2.4 Hz), 55.6; ^19^F NMR (282 MHz, CDCl_3_): *δ* −66.93; HRMS (ESI) *m*/*z* [M+H]^+^ calcd for **C_18_H_13_F_3_N_3_O**: 344.1005; found: 344.0998.

10-Chloro-4-(4-methoxy-phenyl)-2-(trifluoromethyl)pyrimido[1,2-*b*]indazole **(4b)**.

The purification of the crude product using chromatography on silica gel was carried out using (PE/EtOAc: 9.8/0.2) to afford **4b** as a yellow solid with a yield of 85%; m.p. 145–147 °C; ^1^H NMR (300 MHz, CDCl_3_): *δ* 8.29 (d, *J* = 8.7 Hz, 2H), 7.85 (d, *J* = 8.7 Hz, 1H), 7.68 (s, 1H), 7.61 (t, *J* = 7.5 Hz, 1H), 7.40 (d, *J* = 7.5 Hz, 1H), 7.17 (d, *J* = 8.7 Hz, 2H), 3.97 (s, 3H); ^13^C NMR (75 MHz, CDCl_3_): *δ* 162.5, 152.6, 145.9, 143.5, 142.9 (q, *J* = 36.5 Hz), 131.5 (2C), 130.6, 128.2, 122.4, 122.4, 121.2 (q, *J* = 273.0 Hz), 115.5, 114.5 (2C), 111.8, 106.6 (q, *J* = 2.2 Hz), 55.6; ^19^F NMR (282 MHz, CDCl_3_): *δ* −67.17; HRMS (ESI) *m*/*z* [M+H]^+^ calcd for **C_18_H_12_ClF_3_N_3_O**: 378.0615; found: 378.0608.

10-Fluoro-4-(4-methoxy-phenyl)-2-(trifluoromethyl)pyrimido[1,2-*b*]indazole **(4c)**.

The purification of the crude product using chromatography on silica gel was carried out using (PE/EtOAc: 9/1) to afford **4c** as a yellow solid with a yield of 79%; m.p. 172–174 °C; ^1^H NMR (300 MHz, CDCl_3_): *δ* 8.31 (d, *J* = 9.0 Hz, 2H), 7.73 (dd, *J* = 9.9, 7.2 Hz, 1H), 7.68 (s, 1H), 7.66–7.61 (m, 1H), 7.18 (d, *J* = 9.0 Hz, 2H), 7.05 (dd, *J* = 9.9, 7.2 Hz, 1H), 3.97 (s, 3H); ^13^C NMR (75 MHz, CDCl_3_): *δ* 162.5, 157.1 (d, *J* = 257.5 Hz), 153.4 (d, *J* = 4.2 Hz), 145.9, 143.2 (q, *J* = 36.5 Hz), 142.5, 131.5 (2C), 131.0 (d, *J* = 7.7 Hz), 122.3, 121.1 (q, *J* = 273.0 Hz), 114.4 (2C), 112.9 (d, *J* = 4.7 Hz), 106.5 (q, *J* = 2.2 Hz), 106.0 (d, *J* = 17.6 Hz), 104.6 (d, *J* = 17.7 Hz), 55.6; ^19^F NMR (282 MHz, CDCl_3_): *δ* −67.05, −114.97; HRMS (ESI) *m*/*z* [M+H]^+^ calcd for **C_18_H_12_F_4_N_3_O**: 362.0911; found: 362.0904.

4-(4-Methoxy-phenyl)-2,8-bis-trifluoromethyl-pyrimido[1,2-*b*]indazole **(4d)**.

The purification of the crude product using chromatography on silica gel was carried out using (PE/EtOAc: 9.6/0.4) to afford **4d** as a yellow solid with a yield of 85%; m.p. 186–188 °C; ^1^H NMR (300 MHz, CDCl_3_): *δ* 8.56 (dd, *J* = 8.7, 0.9 Hz, 1H), 8.33 (d, *J* = 9.0 Hz, 2H), 8.29 (br s, 1H), 7.72 (s, 1H), 7.60 (dd, *J* = 8.7, 0.9 Hz, 1H), 7.19 (d, *J* = 9.0 Hz, 2H), 3.98 (s, 3H); ^13^C NMR (75 MHz, CDCl_3_): *δ* 162.7, 150.5, 146.5, 144.0, 143.3 (q, *J* = 36.7 Hz), 132.4 (q, *J* = 32.0 Hz), 131.6 (2C), 124.2 (q, *J* = 271.0 Hz), 122.6, 122.1, 121.1 (q, *J* = 272.9 Hz), 117.91 (q, *J* = 3.0 Hz), 115.4, 115.2 (q, *J* = 4.8 Hz), 114.5 (2C), 106.9 (q, *J* = 2.4 Hz), 55.6; ^19^F NMR (282 MHz, CDCl_3_): *δ* −62.41, −67.12; HRMS (ESI) *m*/*z* [M+H]^+^ calcd for **C_19_H_12_F_6_N_3_O**: 412.0879; found: 412.0872.

4-(4-Methoxy-phenyl)-9-nitro-2-(trifluoromethyl)pyrimido[1,2-*b*]indazole **(4e)**.

The purification of the crude product using chromatography on silica gel was carried out using (PE/EtOAc: 9/1) to afford **4e** as an orange solid with a yield of 84%; m.p. 201–203 °C; ^1^H NMR (300 MHz, CDCl_3_): *δ* 9.47 (d, *J* = 1.8 Hz, 1H), 8.53 (dd, *J* = 9.6, 2.1 Hz, 1H), 8.34 (d, *J* = 9.0 Hz, 2H), 7.78 (dd, *J* = 9.6, 2.1 Hz, 1H), 7.80 (s, 1H), 7.21 (d, *J* = 9.0 Hz, 2H), 3.99 (s, 3H); ^13^C NMR (75 MHz, DMSO-*d*_6_): *δ* 163.0, 153.0, 147.6, 146.3, 144.9 (q, *J* = 36.8 Hz), 142.5, 131.8 (2C), 125.0, 121.6, 120.8 (q, *J* = 273.4 Hz), 120.1, 117.7, 114.6 (2C), 112.8, 107.7 (q, *J* = 2.3 Hz), 55.7; ^19^F NMR (282 MHz, CDCl_3_): *δ* −67.32; HRMS (ESI) *m*/*z* [M+H]^+^ calcd for **C_18_H_12_F_3_N_4_O_3_**: 389.0856; found: 389.0849.

7-(4-Methoxyphenyl)-9-(trifluoromethyl)pyrido[3′,2′:3,4]pyrazolo[1,5-*a*]pyrimidine **(4f)**.

The purification of the crude product using chromatography on silica gel was carried out using (PE/EtOAc: 8/2) to afford **4f** as a yellow solid with a yield of 73%; m.p. 186–188 °C; ^1^H NMR (300 MHz, CDCl_3_): *δ* 8.92 (dd, *J* = 4.2, 1.2 Hz, 1H), 8.31 (d, *J* = 9.0 Hz, 2H), 8.30 (dd, *J* = 8.7, 1.2 Hz, 1H), 7.72 (s, 1H), 7.63 (dd, *J* = 8.7, 4.2 Hz, 1H), 7.20 (d, *J* = 9.0 Hz, 2H), 3.97 (s, 3H); ^13^C NMR (75 MHz, CDCl_3_): *δ* 162.6, 147.9, 146.8, 145.4, 143.9 (q, *J* = 36.7 Hz), 143.3, 131.6 (2C), 130.6, 125.0, 122.2, 121.9, 121.0 (q, *J* = 273.4 Hz), 114.5 (2C), 107.1 (q, *J* = 2.2 Hz), 55.6; ^19^F NMR (282 MHz, CDCl_3_): *δ* −67.04; HRMS (ESI) *m*/*z* [M+H]^+^ calcd for **C_17_H_12_F_3_N_4_O**: 345.0957; found: 345.0950.

4-(4-Methoxyphenyl)-2-(trifluoromethyl)pyrido[2′,3′:3,4]pyrazolo[1,5-*a*]pyrimidine **(4g)**.

The purification of the crude product using chromatography on silica gel was carried out using (PE/EtOAc: 8/2) to afford **4g** as a yellow solid with a yield of 93%; m.p. 184–186 °C; ^1^H NMR (300 MHz, CDCl_3_): *δ* 9.06 (dd, *J* = 4.2, 1.5 Hz, 1H), 8.80 (dd, *J* = 8.7, 1.5 Hz, 1H), 8.47 (d, *J* = 9.0 Hz, 2H), 7.78 (s, 1H), 7.40 (dd, *J* = 8.7, 4.2 Hz, 1H), 7.17 (d, *J* = 9.0 Hz, 2H), 3.97 (s, 3H); ^13^C NMR (75 MHz, CDCl_3_): *δ* 162.7, 161.1, 155.3, 146.6, 143.6 (q, *J* = 36.4 Hz), 143.4, 132.0 (2C), 130.9, 121.9, 121.1 (q, *J* = 273.4 Hz), 117.9, 114.4 (2C), 106.8 (q, *J* = 2.3 Hz), 106.6, 55.6; ^19^F NMR (282 MHz, CDCl_3_): *δ* −67.22; HRMS (ESI) *m*/*z* [M+H]^+^ calcd for **C_17_H_12_F_3_N_4_O**: 345.0957; found: 345.0951.

7-(4-Methoxyphenyl)-9-(trifluoromethyl)pyrazino[2′,3′:3,4]pyrazolo[1,5-*a*]pyrimidine **(4h)**.

The purification of the crude product using chromatography on silica gel was carried out using (PE/EtOAc: 8/2) to afford **4h** as a yellow solid with a yield of 35%; m.p. 199–201 °C; ^1^H NMR (300 MHz, CDCl_3_): *δ* 8.99 (d, *J* = 1.8 Hz, 1H), 8.89 (d, *J* = 1.8 Hz, 1H), 8.43 (d, *J* = 8.7 Hz, 2H), 7.86 (s, 1H), 7.19 (d, *J* = 8.7 Hz, 2H), 3.98 (s, 3H); ^13^C NMR (75 MHz, CDCl_3_): *δ* 163.1, 154.7, 149.2, 147.8, 146.1, 145.4 (q, *J* = 37.6 Hz), 142.8, 132.1 (2C), 123.7, 121.3, 120.8 (q, *J* = 273.6 Hz), 114.5 (2C), 108.0 (q, *J* = 2.3 Hz), 55.7; ^19^F NMR (282 MHz, CDCl_3_): *δ* −67.28; HRMS (ESI) *m*/*z* [M+H]^+^ calcd for **C_16_H_11_F_3_N_5_O**: 346.0910; found: 346.0903.

4-*p*-Tolyl-2-(trifluoromethyl)pyrimido[1,2-*b*]indazole **(5a)**.

The purification of the crude product using chromatography on silica gel was carried out using (PE/EtOAc: 9.8/0.2) to afford **5a** as a yellow solid with a yield of 98%; m.p. 159–161 °C; ^1^H NMR (300 MHz, CDCl_3_): *δ* 8.46 (d, *J* = 8.7 Hz, 1H), 8.18 (d, *J* = 8.1 Hz, 2H), 7.96 (d, *J* = 8.7 Hz, 1H), 7.73 (td, *J* = 6.9, 1.2 Hz, 1H), 7.64 (s, 1H), 7.49 (d, *J* = 8.1 Hz, 2H), 7.43 (td, *J* = 6.9, 1.2 Hz, 1H), 2.53 (s, 3H); ^13^C NMR (75 MHz, CDCl_3_): *δ* 151.9, 146.0, 144.0, 142.5, 142.0 (q, *J* = 36.4 Hz), 130.6, 129.7 (2C), 129.5 (2C), 127.7, 122.2, 121.3 (q, *J* = 272.8 Hz), 121.2, 117.0, 114.1, 106.6 (q, *J* = 2.4 Hz), 21.7; ^19^F NMR (282 MHz, CDCl_3_): *δ* −66.94; HRMS (ESI) *m*/*z* [M+H]^+^ calcd for **C_18_H_13_F_3_N_3_**: 328.1056; found: 328.1051.

7-(3-Methylphenyl)-9-(trifluoromethyl)pyrido[3′,2′:3,4]pyrazolo[1,5-*a*]pyrimidine **(5b)**.

The purification of the crude product using chromatography on silica gel was carried out using (PE/EtOAc: 9/1) to afford **5b** as a yellow solid with a yield of 65%; m.p. 139–141 °C; ^1^H NMR (300 MHz, CDCl_3_): *δ* 8.93 (dd, *J* = 4.2, 1.5 Hz, 1H), 8.93 (dd, *J* = 8.7, 1.5 Hz, 1H), 8.04–8.00 (m, 2H), 7.72 (s, 1H), 7.64 (dd, *J* = 8.7, 4.2 Hz, 1H), 7.59 (t, *J* = 7.5 Hz, 1H), 7.51 (d, *J* = 7.5 Hz, 1H), 2.55 (s, 3H); ^13^C NMR (75 MHz, CDCl_3_): *δ* 148.1, 147.3, 145.5, 143.9 (q, *J* = 36.7 Hz), 143.2, 139.0, 132.9, 130.7, 130.0, 129.9, 129.0, 126.8, 125.2, 125.1, 121.0 (q, *J* = 273.4 Hz), 108.2 (q, *J* = 2.2 Hz), 21.6; ^19^F NMR (282 MHz, CDCl_3_): δ −67.04; HRMS (ESI) *m*/*z* [M+H]^+^ calcd for **C_17_H_12_F_3_N_4_**: 329.1008; found: 329.1003.

4-(4-*Tert*-Butyl-phenyl)-2,8-bis-trifluoromethyl-pyrimido[1,2-*b*]indazole **(5c)**.

The purification of the crude product using chromatography on silica gel was carried out using (PE/EtOAc: 9.8/0.2) to afford **5c** as a yellow solid with a yield of 96%; m.p. 170–172 °C; ^1^H NMR (300 MHz, CDCl_3_): *δ* 8.58 (dd, *J* = 8.4, 0.9 Hz, 1H), 8.30 (s, 1H), 8.23 (d, *J* = 8.4 Hz, 2H), 7.74 (s, 1H), 7.72 (d, *J* = 8.4 Hz, 2H), 7.62 (dd, *J* = 8.4, 0.9 Hz, 1H), 1.44 (s, 9H); ^13^C NMR (75 MHz, CDCl_3_): *δ* 156.0, 150.5, 146.8, 143.9, 143.1 (q, *J* = 36.7 Hz), 132.4 (q, *J* = 31.9 Hz), 129.4 (2C), 127.2, 126.2 (2C), 124.1 (q, *J* = 271.0 Hz), 122.6, 121.0 (q, *J* = 273.1 Hz), 118.0 (q, *J* = 3.0 Hz), 115.5, 115.3 (q, *J* = 4.8 Hz), 107.6 (q, *J* = 2.2 Hz), 35.2, 31.1 (3C); ^19^F NMR (282 MHz, CDCl_3_): δ −62.42, −67.13; HRMS (ESI) *m*/*z* [M+H]^+^ calcd for **C_22_H_18_F_6_N_3_**: 438.1399; found: 438.1397.

7-(3-Methoxyphenyl)-9-(trifluoromethyl)pyrido[3′,2′:3,4]pyrazolo[1,5-*a*]pyrimidine **(5d)**.

The purification of the crude product using chromatography on silica gel was carried out using (PE/EtOAc: 8/2) to afford **5d** as a yellow solid with a yield of 93%; m.p. 189–191 °C; ^1^H NMR (300 MHz, CDCl_3_): δ 8.94 (dd, *J* = 4.2, 1.5 Hz, 1H), 8.93 (dd, *J* = 8.7, 1.5 Hz, 1H), 7.81–7.74 (m, 2H), 7.74 (s, 1H), 7.64 (dd, *J* = 8.7, 4.2 Hz, 1H), 7.59 (d, *J* = 7.8 Hz, 1H), 7.23 (ddd, *J* = 8.4, 2.7, 0.9 Hz, 1H), 3.96 (s, 3H); ^13^C NMR (75 MHz, CDCl_3_): *δ* 159.8, 148.2, 146.9, 145.5, 143.9 (q, *J* = 36.9 Hz), 143.2, 131.1, 130.7, 130.2, 125.2, 125.1, 121.9, 120.9 (q, *J* = 272.3 Hz), 117.6, 115.3, 108.2 (q, *J* = 2.2 Hz), 55.6; ^19^F NMR (282 MHz, CDCl_3_): *δ* −67.02; HRMS (ESI) *m*/*z* [M+H]^+^ calcd for **C_17_H_12_F_3_N_4_O**: 345.0957; found: 345.0952.

7-(3,4-Dimethoxyphenyl)-9-(trifluoromethyl)pyrazino[2′,3′:3,4]pyrazolo[1,5-*a*]pyrimidine **(5e)**.

The purification of the crude product using chromatography on silica gel was carried out using (PE/EtOAc: 7/3) to afford **5e** as a yellow solid with a yield of 44%; m.p. 200–202 °C; ^1^H NMR (300 MHz, CDCl_3_): *δ* 9.0 (d, *J* = 1.8 Hz, 1H), 8.89 (d, *J* = 1.8 Hz, 1H), 8.03 (d, *J* = 2.4 Hz, 1H), 7.99 (dd, *J* = 8.4, 2.4 Hz, 1H), 7.87 (s, 1H), 7.15 (d, *J* = 8.4 Hz, 1H), 4.06 (s, 3H), 4.05 (s, 3H); ^13^C NMR (75 MHz, CDCl_3_): *δ* 154.7, 152.8, 149.2, 149.1, 147.8, 145.4 (q, *J* = 37.0 Hz), 143.3, 142.8, 124.3, 123.7, 121.5, 120.8 (q, *J* = 273.6 Hz), 112.7, 111.2, 108.3 (q, *J* = 2.2 Hz), 56.4, 56.2; ^19^F NMR (282 MHz, CDCl_3_): *δ* −67.24; HRMS (ESI) *m*/*z* [M+H]^+^ calcd for **C_17_H_13_F_3_N_5_O_2_**: 376.1015; found: 376.1008

7-(2,5-Dimethoxyphenyl)-9-(trifluoromethyl)pyrazino[2′,3′:3,4]pyrazolo[1,5-*a*]pyrimidine **(5f)**.

The purification of the crude product using chromatography on silica gel was carried out using (PE/EtOAc: 7/3) to afford **5f** as a yellow solid with a yield of 42%; m.p. 208–210 °C; ^1^H NMR (300 MHz, CDCl_3_): *δ* 8.97 (d, *J* = 1.8 Hz, 1H), 8.88 (d, *J* = 1.8 Hz, 1H), 7.86 (s, 1H), 7.33 (d, *J* = 3.0 Hz, 1H), 7.20 (dd, *J* = 9.0, 3.0 Hz, 1H), 7.15 (d, *J* = 9.0 Hz, 1H), 3.86 (s, 3H), 3.80 (s, 3H); ^13^C NMR (75 MHz, CDCl_3_): *δ* 154.7, 153.5, 151.8, 148.9, 146.5, 144.8 (q, *J* = 37.3 Hz), 142.7, 142.1, 123.9, 120.8 (q, *J* = 273.6 Hz), 118.9, 118.8, 116.0, 113.2, 111.4 (q, *J* = 2.2 Hz), 56.3, 56.0; ^19^F NMR (282 MHz, CDCl_3_): *δ* −67.18; HRMS (ESI) *m*/*z* [M+H]^+^ calcd for **C_17_H_13_F_3_N_5_O_2_**: 376.1015; found: 376.1008.

7-(2,4-dimethoxyphenyl)-9-(trifluoromethyl)pyrazino[2′,3′:3,4]pyrazolo[1,5-*a*]pyrimidine **(5g)**.

The purification of the crude product using chromatography on silica gel was carried out using (PE/EtOAc: 7/3) to afford **11g** as a yellow solid with a yield of 41%; m.p. 206–208 °C; ^1^H NMR (300 MHz, CDCl_3_): *δ* 8.95 (d, *J* = 1.5 Hz, 1H), 8.85 (d, *J* = 1.5 Hz, 1H), 7.95 (dd, *J* = 8.4, 2.1 Hz, 1H), 7.89 (s, 1H), 6.74 (dd, *J* = 8.4, 2.1 Hz, 1H), 6.68 (d, *J* = 2.1 Hz, 1H), 3.95 (s, 3H), 3.85 (s, 3H); ^13^C NMR (75 MHz, CDCl_3_): *δ* 164.1, 159.2, 154.5, 148.9, 146.3, 144.8 (q, *J* = 37.0 Hz), 142.4, 142.3, 132.5, 124.0, 120.9 (q, *J* = 273.6 Hz), 111.2 (q, *J* = 2.3 Hz), 110.9, 105.3, 99.2, 55.9, 55.7; ^19^F NMR (282 MHz, CDCl_3_): *δ* −67.24; HRMS (ESI) *m*/*z* [M+H]^+^ calcd for **C_17_H_13_F_3_N_5_O_2_**: 376.1015; found: 376.1009.

7-[4-(Methylsulfanyl)phenyl]-9-(trifluoromethyl)pyrido[3′,2′:3,4]pyrazolo[1,5-*a*]pyrimidine **(5h)**.

The purification of the crude product using chromatography on silica gel was carried out using (PE/EtOAc: 8/2) to afford **5h** as a yellow solid with a yield of 82%; m.p. 187–189 °C; ^1^H NMR (300 MHz, CDCl_3_): *δ* 8.93 (dd, *J* = 3.9, 1.2 Hz, 1H), 8.30 (dd, *J* = 8.7, 1.2 Hz, 1H), 8.24 (d, *J* = 8.4 Hz, 2H), 7.73 (s, 1H), 7.64 (dd, *J* = 8.7, 3.9 Hz, 1H), 7.51 (d, *J* = 8.4 Hz, 2H), 2.62 (s, 3H); ^13^C NMR (75 MHz, CDCl_3_): *δ* 148.1, 146.5, 145.5, 145.0, 143.9 (q, *J* = 36.9 Hz), 143.3, 130.6, 129.9 (2C), 125.7, 125.6 (2C), 125.1, 125.1, 121.0 (q, *J* = 273.4 Hz), 107.5 (q, *J* = 2.2 Hz), 14.9; ^19^F NMR (282 MHz, CDCl_3_): *δ* −67.03; HRMS (ESI) *m*/*z* [M+H]^+^ calcd for **C_17_H_12_F_3_N_4_S**: 361.0729; found: 361.0724.

4-(2*H*-1,3-Benzodioxol-4-yl)-2-(trifluoromethyl)pyrido[2′,3′:3,4]pyrazolo[1,5-*a*]pyrimidine **(5i)**.

The purification of the crude product using chromatography on silica gel was carried out using (PE/EtOAc: 8/2) to afford **5i** as a yellow solid with a yield of 88%; m.p. 212–214 °C; ^1^H NMR (300 MHz, CDCl_3_): *δ* 9.07 (dd, *J* = 4.2, 1.8 Hz, 1H), 8.80 (dd, *J* = 8.1, 1.8 Hz, 1H), 8.00 (d, *J* = 1.8 Hz, 1H), 7.93 (dd, *J* = 8.4, 1.8 Hz, 1H), 7.75 (s, 1H), 7.41 (dd, *J* = 8.1, 4.2 Hz, 1H), 7.08 (dd, *J* = 8.4, 1.8 Hz, 1H), 6.16 (s, 2H); ^13^C NMR (75 MHz, CDCl_3_): *δ* 161.0, 155.4, 151.0, 148.1, 146.4, 143.6 (q, *J* = 36.6 Hz), 143.4, 130.9, 125.6, 123.4, 121.0 (q, *J* = 273.1 Hz), 118.0, 110.0, 108.8, 107.2 (q, *J* = 2.4 Hz), 106.7, 102.1; ^19^F NMR (282 MHz, CDCl_3_): *δ* −67.22; HRMS (ESI) *m*/*z* [M+H]^+^ calcd for **C_17_H_10_F_3_N_4_O_2_**: 359.0750; found: 359.0746.

4-(4-Chloro-phenyl)-2,8-bis-trifluoromethyl-pyrimido[1,2-*b*]indazole **(5j)**.

The purification of the crude product using chromatography on silica gel was carried out using (PE/EtOAc: 9.8/0.2) to afford **5j** as a yellow solid with a yield of 50%; m.p. 137–139 °C; ^1^H NMR (300 MHz, CDCl_3_): *δ* 8.57 (dd, *J* = 8.7, 1.2 Hz, 1H), 8.27 (s, 1H), 8.25 (d, *J* = 8.7 Hz, 2H), 7.23 (s, 1H), 7.68 (d, *J* = 8.7 Hz, 2H), 7.63 (dd, *J* = 8.7, 1.2 Hz, 1H); ^13^C NMR (75 MHz, CDCl_3_): *δ* 150.5, 145.4, 143.9, 143.9, 143.0 (q, *J* = 36.8 Hz), 132.6 (q, *J* = 32.0 Hz), 131.0 (2C), 129.4 (2C), 128.4, 124.1 (q, *J* = 271.0 Hz), 122.6, 120.9 (q, *J* = 273.1 Hz), 118.4 (q, *J* = 3.1 Hz), 115.5, 115.3 (q, *J* = 4.7 Hz), 107.6 (q, *J* = 2.3 Hz); ^19^F NMR (282 MHz, CDCl_3_): *δ* −62.45, −67.08; HRMS (ESI) *m*/*z* [M+H]^+^ calcd for **C_18_H_9_ClF_6_N_3_**: 416.0383; found: 416.0379

4-(4-Fluoro-phenyl)-2-(trifluoromethyl)pyrimido[1,2-*b*]indazole **(5k)**.

The purification of the crude product using chromatography on silica gel was carried out using (PE/EtOAc: 9.9/0.1) to afford **5k** as an orange solid with a yield of 88%; m.p. 186–188 °C; ^1^H NMR (300 MHz, CDCl_3_): *δ* 8.47 (d, *J* = 8.4 Hz, 1H), 8.33 (dd, *J* = 8.7, 5.4 Hz, 2H), 7.95 (d, *J* = 8.4 Hz, 1H), 7.74 (td, *J* = 6.9, 1.2 Hz, 1H), 7.64 (s, 1H), 7.47 (td, *J* = 6.9, 1.2 Hz, 1H), 7.38 (dd, *J* = 8.7, 5.4 Hz, 2H); ^13^C NMR (75 MHz, CDCl_3_): *δ* 164.6 (d, *J* = 252.4 Hz), 151.9, 144.7, 144.0, 142.0 (q, *J* = 36.4 Hz), 132.0, 131.9, 130.8, 126.7 (d, *J* = 3.2 Hz), 122.5, 121.2, 121.2 (q, *J* = 272.8 Hz), 117.0, 116.4, 116.1, 114.1, 106.7 (q, *J* = 2.2 Hz); ^19^F NMR (282 MHz, CDCl_3_): *δ* −66.93, −106.56; HRMS (ESI) *m*/*z* [M+H]^+^ calcd for **C_17_H_10_F_4_N_3_**: 332.0805; found: 332.0799.

2-Trifluoromethyl-4-(4-trifluoromethyl-phenyl)-pyrimido[1,2-*b*]indazole **(5l)**.

The purification of the crude product using chromatography on silica gel was carried out using (PE/EtOAc: 9.8/0.2) to afford **5l** as an orange solid with a yield of 84%; m.p. 180–182 °C; ^1^H NMR (300 MHz, CDCl_3_): *δ* 8.47 (d, *J* = 8.4 Hz, 1H), 8.39 (d, *J* = 8.1 Hz, 2H), 7.95 (d, *J* = 8.4 Hz, 3H), 7.75 (td, *J* = 7.8, 0.9 Hz, 1H), 7.67 (s, 1H), 7.48 (td, *J* = 7.8, 0.9 Hz, 1H); ^13^C NMR (75 MHz, CDCl_3_): *δ* 152.0, 144.1, 144.0, 141.9 (q, *J* = 36.6 Hz), 134.0, 133.4 (q, *J* = 32.3 Hz), 131.0, 130.0 (2C), 129.0, 126.0 (q, *J* = 3.5 Hz), 123.6 (q, *J* = 271.4 Hz), 122.8, 121.2, 121.1 (q, *J* = 272.2 Hz), 117.0, 114.2, 107.3 (q, *J* = 1.6 Hz); ^19^F NMR (282 MHz, CDCl_3_): *δ* −63.10, −66.92; HRMS (ESI) *m*/*z* [M+H]^+^ calcd for **C_18_H_10_F_6_N_3_**: 382.0773; found: 382.0767.

4-(4-Trifluoromethoxy-phenyl)-2-(trifluoromethyl)pyrimido[1,2-*b*]indazole **(5m)**.

The purification of the crude product using chromatography on silica gel was carried out using (PE/EtOAc: 9.8/0.2) to afford **5m** as an orange solid with a yield of 86%; m.p. 118–120 °C; ^1^H NMR (300 MHz, CDCl_3_): *δ* 8.46 (d, *J* = 8.7 Hz, 1H), 8.36 (d, *J* = 9.0 Hz, 2H), 7.95 (d, *J* = 8.7 Hz, 1H), 7.75 (td, *J* = 7.5, 0.9 Hz, 1H), 7.65 (s, 1H), 7.53 (d, *J* = 9.0 Hz, 2H), 7.49 (td, *J* = 7.5, 0.9 Hz, 1H); ^13^C NMR (75 MHz, CDCl_3_): *δ* 152.0, 151.5, 151.4, 144.3, 144.0, 142.0 (q, *J* = 36.6 Hz), 131.5 (2C), 130.9, 129.0, 122.6, 121.2, 121.2 (q, *J* = 272.8 Hz), 121.1, 120.4 (q, *J* = 257.3 Hz), 117.0, 114.2, 107.0 (q, *J* = 2.2 Hz); ^19^F NMR (282 MHz, CDCl_3_): *δ* −57.53, −66.93; HRMS (ESI) *m*/*z* [M+H]^+^ calcd for **C_18_H_10_F_6_N_3_O**: 398.0722; found: 398.0718.

Ethyl 4-[9-(trifluoromethyl)pyrazino[2′,3′:3,4]pyrazolo[1,5-*a*]pyrimidin-7-yl]benzoate **(5n)**.

The purification of the crude product using chromatography on silica gel was carried out using (PE/EtOAc: 9/1) to afford **5n** as a yellow solid with a yield of 42%; m.p. 148–150 °C; ^1^H NMR (300 MHz, CDCl_3_): *δ* 9.02 (d, *J* = 1.8 Hz, 1H), 8.93 (d, *J* = 1.8 Hz, 1H), 8.38 (q, *J* = 7.8 Hz, 4H), 7.91 (s, 1H), 4.49 (q, *J* = 7.8 Hz, 2H), 1.48 (t, *J* = 6.9 Hz, 3H); ^13^C NMR (75 MHz, CDCl_3_): *δ* 165.4, 154.9, 149.4, 147.0, 145.4 (q, *J* = 36.0 Hz), 143.3, 142.6, 133.9, 133.1, 130.1 (2C), 130.0 (2C), 123.8, 120.6 (q, *J* = 273.7 Hz), 109.5 (q, *J* = 2.2 Hz), 61.6, 14.3; ^19^F NMR (282 MHz, CDCl_3_): *δ* −67.22; HRMS (ESI) *m*/*z* [M+H]^+^ calcd for **C_18_H_13_F_3_N_5_O_2_**: 388.1015; found: 388.1010.

4-[2-(Trifluoromethyl)pyrido[2′,3′:3,4]pyrazolo[1,5-*a*]pyrimidin-4-yl]benzaldehyde **(5o)**.

The purification of the crude product using chromatography on silica gel was carried out using (PE/EtOAc: 8/2) to afford **5o** as a yellow solid with a yield of 89%; m.p. 237–239 °C; ^1^H NMR (300 MHz, CDCl_3_): *δ* 10.20 (s, 1H), 9.10 (dd, *J* = 4.2, 1.8 Hz, 1H), 8.84 (dd, *J* = 8.1, 1.8 Hz, 1H), 8.55 (d, *J* = 8.4 Hz, 2H), 8.18 (d, *J* = 8.4 Hz, 2H), 7.85 (s, 1H), 7.47 (dd, *J* = 8.1, 4.2 Hz, 1H); ^13^C NMR (75 MHz, CDCl_3_): *δ* 191.3, 161.2, 155.7, 145.3, 143.5 (q, *J* = 37.0 Hz), 143.2, 138.3, 135.2, 130.9, 130.6 (2C), 129.9 (2C), 120.9 (q, *J* = 273.1 Hz), 118.6, 108.3 (q, *J* = 2.4 Hz), 106.9; ^19^F NMR (282 MHz, CDCl_3_): *δ* −67.15; HRMS (ESI) *m*/*z* [M+H]^+^ calcd for **C_17_H_10_F_3_N_4_O**: 343.0801; found: 343.0798.

4-[9-(Trifluoromethyl)pyrido[3′,2′:3,4]pyrazolo[1,5-*a*]pyrimidin-7-yl]benzonitrile **(5p)**.

The purification of the crude product using chromatography on silica gel was carried out using (PE/EtOAc: 8.4/1.6) to afford **5p** as a yellow solid with a yield of 33%; m.p. 201–203 °C; ^1^H NMR (300 MHz,CDCl_3_): *δ* 8.98 (dd, *J* = 3.9, 1.2 Hz, 1H), 8.40 (d, *J* = 8.7 Hz, 2H), 8.31 (dd, *J* = 8.7, 1.2 Hz, 1H), 8.00 (d, *J* = 8.7 Hz, 2H), 7.77 (s, 1H), 7.67 (dd, *J* = 8.7, 3.9 Hz, 1H); ^13^C NMR (75 MHz, CDCl_3_): *δ* 148.7, 145.5, 144.6, 143.8 (q, *J* = 37.4 Hz), 143.2, 134.1, 132.7 (2C), 130.7, 130.4 (2C), 125.5, 125.2, 120.7 (q, *J* = 273.4 Hz), 117.8, 115.6, 108.5 (q, *J* = 2.2 Hz); ^19^F NMR (282 MHz, CDCl_3_): *δ* −66.99; HRMS (ESI) *m*/*z* [M+H]^+^ calcd for **C_17_H_9_F_3_N_5_**: 340.0804; found: 340.0800

7-(4-Nitrophenyl)-9-(trifluoromethyl)pyrido[3′,2′:3,4]pyrazolo[1,5-*a*]pyrimidine **(5q)**.

The purification of the crude product using chromatography on silica gel was carried out using (PE/EtOAc: 8/2) to afford **5q** as a yellow orange solid with a yield of 77%; m.p. 284–286 °C; ^1^H NMR (300 MHz, CDCl_3_): *δ* 8.99 (dd, *J* = 4.2, 1.5 Hz, 1H), 8.56 (d, *J* = 9.0 Hz, 2H), 8.47 (d, *J* = 9.0 Hz, 2H), 8.32 (dd, *J* = 8.7, 1.5 Hz, 1H), 7.80 (s, 1H), 7.69 (dd, *J* = 8.7, 4.2 Hz, 1H); ^13^C NMR (75 MHz, DMSO-*d*_6_): *δ* 149.4, 148.5, 145.3, 145.2, 143.0, 142.4 (q, *J* = 37.8 Hz), 136.4, 132.2 (2C), 130.6, 125.9, 125.5, 124.0 (2C), 121.6 (q, *J* = 273.2 Hz), 116.3, 110.2 (q, *J* = 1.9 Hz); ^19^F NMR (282 MHz, CDCl_3_): *δ* −66.97; HRMS (ESI) *m*/*z* [M+H]^+^calcd for **C_16_H_9_F_3_N_5_O_2_**: 360.0702; found: 360.0698.

### 3.5. General Procedure for the Synthesis of 4-Amino-2-trifluoromethyl Pyrimido[1,2-b]indazole Derivatives ***6a–g***

A mixture of compounds **3f** (1mmol, 1.0 equiv.) and the corresponding amine (1.2 mmol, 1.2 equiv.) was dissolved in absolute EtOH (5 mL), Et_3_N (2 mmol) was then added and the mixture was refluxed for 2–3 h. The progress of the reaction was monitored by TLC analysis (eluent: petroleum ether/ethyl acetate, 8:2). After cooling to room temperature, the solvent was evaporated under reduced pressure and the crude residue was tritured in water and then filtered, dried and recrystallized from EtOH to give the pure products **6a–g**. (^1^H-NMR, ^19^F-NMR and ^13^C-NMR of compounds **6a–g** are shown in Supplementary Materials).

*N*-Propyl-9-(trifluoromethyl)pyrido[3′,2′:3,4]pyrazolo[1,5-*a*]pyrimidin-7-amine **(6a)**.

Compound **6a** was obtained as a light brown solid with a yield of 52%; m.p. 187–189 °C; ^1^H NMR (300 MHz, CDCl_3_): *δ* 8.78 (dd, *J* = 3.9, 1.2 Hz, 1H), 8.14 (dd, *J* = 8.7, 1.2 Hz, 1H), 7.56 (dd, *J* = 8.7, 3.9 Hz, 1H), 7.00 (br s, 1H), 6.74 (s, 1H), 3.59 (q, *J* = 6.9 Hz, 2H), 1.93 (sext, *J* = 7.2 Hz, 2H), 1.16 (t, *J* = 7.2 Hz, 3H); ^13^C NMR (75 MHz, CDCl_3_): *δ* 146.8, 146.3, 146.0 (q, *J* = 35.8 Hz), 144.5, 142.1, 130.6, 124.8, 124.3, 121.2 (q, *J* = 273.6 Hz), 85.9 (q, *J* = 2.6 Hz), 44.3, 22.3, 11.4; ^19^F NMR (282 MHz, CDCl_3_): δ −67.53; HRMS (ESI) *m*/*z* [M+H]^+^ calcd for **C_13_H_13_F_3_N_5_**: 296.1117; found: 296.1113.

*N*-[(4-Methoxyphenyl)methyl]-9-(trifluoromethyl)pyrido[3′,2′:3,4]pyrazolo[1,5-*a*]pyrimidin-7-amine **(6b)**.

Compound **6b** was obtained as a light brown solid with a yield of 62%; m.p. 159–161 °C; ^1^H NMR (300 MHz, CDCl_3_): *δ* 8.80 (dd, *J* = 4.2, 0.9 Hz, 1H), 8.15 (dd, *J* = 8.7, 0.9 Hz, 1H), 7.75 (dd, *J* = 8.7, 4.2 Hz, 1H), 7.37 (d, *J* = 8.4 Hz, 2H), 6.97 (d, *J* = 8.4 Hz, 2H), 6.80 (s, 1H), 4.73 (d, *J* = 5.4 Hz, 2H), 3.85 (s, 3H); ^13^C NMR (75 MHz, CDCl_3_): *δ* 159.8, 146.5, 145.9 (q, *J* = 35.8 Hz), 144.6, 142.2, 132.0, 130.8, 129.0 (2C), 126.9, 124.9, 124.2, 121.1 (q, *J* = 273.6 Hz), 114.6 (2C), 86.3 (q, *J* = 2.4 Hz), 55.4, 46.2; ^19^F NMR (282 MHz, CDCl_3_): *δ* −67.52; HRMS (ESI) *m*/*z* [M+H]^+^ calcd for **C_18_H_15_F_3_N_5_O**: 374.1223; found: 374.1221.

7-(Morpholin-4-yl)-9-(trifluoromethyl)pyrido[3′,2′:3,4]pyrazolo[1,5-*a*]pyrimidine **(6c)**.

Compound **6c** was obtained as a gray solid with a yield of 60%; m.p. 270–272 °C; ^1^H NMR (300 MHz, CDCl_3_): *δ* 8.84 (dd, *J* = 3.9, 1.2 Hz, 1H), 8.22 (dd, *J* = 8.7, 1.2 Hz, 1H), 7.59 (dd, *J* = 8.7, 3.9 Hz, 1H), 6.91 (s, 1H), 4.10–4.08 (m, 4H), 4.03–4.01 (m, 4H); ^13^C NMR (75 MHz, CDCl_3_): *δ* 150.1, 147.1, 145.2 (q, *J* = 36.1 Hz), 144.5, 144.1, 130.1, 125.0, 124.7, 121.0 (q, *J* = 273.7 Hz), 93.3, 66.2 (2C), 48.5 (2C); ^19^F NMR (282 MHz, CDCl_3_): *δ* −67.27; HRMS (ESI) *m*/*z* [M+H]^+^ calcd for **C_14_H_13_F_3_N_5_O**: 324.1066; found: 324.1064.

7-(Pyrrolidin-1-yl)-9-(trifluoromethyl)pyrido[3′,2′:3,4]pyrazolo[1,5-*a*]pyrimidine **(6d)**.

Compound **6d** was obtained as a yellow solid with a yield of 78%; m.p. 271–273 °C; ^1^H NMR (300 MHz, CDCl_3_): *δ* 8.74 (dd, *J* = 3.9, 1.2 Hz, 1H), 8.10 (dd, *J* = 8.7, 1.2 Hz, 1H), 7.50 (dd, *J* = 8.7, 3.9 Hz, 1H), 6.50 (s, 1H), 4.25–4.21 (m, 4H), 2.19–2.15 (m, 4H); ^13^C NMR (75 MHz, CDCl_3_): *δ* 147.7, 146.0 (q, *J* = 35.2 Hz), 145.9, 144.7, 144.1, 129.2, 124.5, 124.4, 121.3 (q, *J* = 273.6 Hz), 89.7 (q, *J* = 2.5 Hz), 51.8 (2C), 25.5 (2C); ^19^F NMR (282 MHz, CDCl_3_): *δ* −67.77; HRMS (ESI) *m*/*z* [M+H]^+^ calcd for **C_14_H_13_F_3_N_5_**: 308.1117; found: 308.1115.

*N*-Phenyl-9-(trifluoromethyl)pyrido[3′,2′:3,4]pyrazolo[1,5-*a*]pyrimidin-7-amine **(6e)**.

Compound **6e** was obtained as a light brown solid with a yield of 48%; m.p. 170–172 °C; ^1^H NMR (300 MHz, CDCl_3_): *δ* 8.85 (dd, *J* = 3.6, 1.2 Hz, 1H), 8.71 (s, 1H), 8.24 (d, *J* = 8.7, 1.2 Hz, 1H), 7.65 (dd, *J* = 8.7, 3.9 Hz, 1H), 7.60 (t, *J* = 7.8 Hz, 2H), 7.50 (d, *J* = 8.1 Hz, 2H), 7.44 (d, *J* = 8.1 Hz, 1H), 7.10 (s, 1H); ^13^C NMR (75 MHz, CDCl_3_): *δ* 146.8, 146.0 (q, *J* = 36.0 Hz), 145.2, 144.7, 142.4, 135.2, 130.8, 130.4 (2C), 127.7, 125.1, 124.4, 124.1 (2C), 121.0 (q, *J* = 273.6 Hz), 87.4 (q, *J* = 2.5 Hz); ^19^F NMR (282 MHz, CDCl_3_): *δ* −67.46; HRMS (ESI) *m*/*z* [M+H]^+^ calcd for **C_16_H_11_F_3_N_5_**: 330.0961; found: 330.0956.

*N*-(4-Methylphenyl)-9-(trifluoromethyl)pyrido[3′,2′:3,4]pyrazolo[1,5-*a*]pyrimidin-7-amine **(6f)**.

Compound **6f** was obtained as a light brown solid with a yield of 49%; m.p. 207–209 °C; ^1^H NMR (300 MHz, CDCl_3_): *δ* 8.83 (dd, *J* = 3.9, 1.2 Hz, 1H), 8.61 (s, 1H), 8.21 (dd, *J* = 9.0, 1.2 Hz, 1H), 7.60 (dd, *J* = 9.0, 3.9 Hz, 1H), 7.34 (br s, 4H), 7.02 (s, 1H), 2.47 (s, 3H); ^13^C NMR (75 MHz, CDCl_3_): *δ* 146.7, 146.0 (q, *J* = 36.0 Hz), 145.5, 144.7, 142.4, 137.9, 132.4, 130.9 (2C), 130.8, 125.0, 124.3 (2C), 124.2, 121.1 (q, *J* = 273.6 Hz), 87.3 (q, *J* = 2.5 Hz), 21.1; ^19^F NMR (282 MHz, CDCl_3_): δ −67.47; HRMS (ESI) *m*/*z* [M+H]^+^ calcd for **C_17_H_13_F_3_N_5_**: 344.1117; found: 344.1113.

*N*-(4-Bromophenyl)-9-(trifluoromethyl)pyrido[3′,2′:3,4]pyrazolo[1,5-*a*]pyrimidin-7-amine **(6g)**.

Compound **6g** was obtained as a light brown solid with a yield of 54%; m.p. 249–251 °C; ^1^H NMR (300 MHz, CDCl_3_): *δ* 8.84 (dd, *J* = 4.2, 1.2 Hz, 1H), 8.63 (s, 1H), 8.20 (dd, *J* = 8.7, 1.2 Hz, 1H), 7.72 (d, *J* = 8.7 Hz, 2H), 7.61 (dd, *J* = 8.7, 4.2 Hz, 1H), 7.39 (d, *J* = 8.7 Hz, 2H), 7.05 (s, 1H); ^13^C NMR (75 MHz, DMSO-*d*_6_): δ 146.8, 146.4, 144.6, 144.4 (q, *J* = 34.8 Hz), 142.8, 136.4, 133.1 (2C), 130.4, 127.2 (2C), 125.6, 124.6, 121.7 (q, *J* = 273.3 Hz), 119.5, 88.3 (q, *J* = 2.3 Hz); ^19^F NMR (282 MHz, CDCl_3_): δ −67.44; HRMS (ESI) *m*/*z* [M+H]^+^ calcd for **C_16_H_10_BrF_3_N_5_**: 408.0066; found: 408.0066.

### 3.6. General Procedure for the Synthesis of 4-(Alkylthio or Arylthiol)-2-(trifluoromethyl)pyrido[2′,3′:3,4]pyrazolo[1,5-a]pyrimidine ***7a–c***

A mixture of compound **3g** (1 mmol, 1.0 equiv.) and the corresponding thiol (1.1 mmol, 1.1 equiv.) was dissolved in absolute EtOH (5 mL), Et_3_N (2 mmol) was then added and the mixture was stirred at room temperature for 30 min. Then, the solvent was evaporated under reduced pressure and the crude residue was filtered, washed with water, dried, and recrystallized from EtOH to give the pure products **7a–c**. (^1^H-NMR, ^19^F-NMR and ^13^C-NMR of compounds **7a–c** are shown in Supplementary Materials).

Ethyl{[2-(trifluoromethyl)pyrido[2′,3′:3,4]pyrazolo[1,5-*a*]pyrimidin-4-yl]sulfanyl}acetate **(7a)**.

Compound **7a** was obtained as a yellow solid with a yield of 67%; m.p. 176–178 °C; ^1^H NMR (300 MHz, DMSO-*d*_6_): *δ* 9.03 (dd, *J* = 4.2, 1.2 Hz, 1H), 8.85 (dd, *J* = 8.1, 1.2 Hz, 1H), 7.99 (s, 1H), 7.78 (dd, *J* = 8.1, 4.2 Hz, 1H), 4.65 (s, 2H), 4.21 (q, *J* = 7.2 Hz, 2H), 1.23 (t, *J* = 7.2 Hz, 3H); ^13^C NMR (75 MHz, DMSO-*d*_6_): *δ* 168.3, 160.5, 155.7, 150.8, 141.7 (q, *J* = 35.8 Hz), 141.2, 131.4, 121.4 (q, *J* = 273.3 Hz), 118.6, 106.5, 105.7 (q, *J* = 2.1 Hz), 62.3, 32.9, 14.4; ^19^F NMR (282 MHz, DMSO-*d*_6_): *δ* −66.63; HRMS (ESI) *m*/*z* [M+H]^+^ calcd for **C_14_H_12_F_3_N_4_O_2_S**: 357.0627; found: 357.0622.

4-[(4-Methoxyphenyl)sulfanyl]-2-(trifluoromethyl)pyrido[2′,3′:3,4]pyrazolo[1,5-*a*]pyrimidine **(7b)**.

Compound **7b** was obtained as a beige solid with a yield of 79%; m.p. 247–249 °C; ^1^H NMR (300 MHz, CDCl_3_): *δ* 9.08 (dd, *J* = 4.2, 1.5 Hz, 1H), 8.75 (dd, *J* = 8.1, 1.5 Hz, 1H), 7.67 (d, *J* = 8.7 Hz, 2H), 7.39 (dd, *J* = 8.1, 4.2 Hz, 1H), 7.15 (d, *J* = 8.7 Hz, 2H), 6.82 (s, 1H), 3.96 (s, 3H); ^13^C NMR (75 MHz, CDCl_3_): *δ* 162.3, 161.0, 155.4, 154.0, 142.7 (q, *J* = 36.4 Hz), 141.4, 137.7 (2C), 131.0, 120.8 (q, *J* = 273.4 Hz), 118.0, 116.5 (2C), 115.0, 106.6, 103.7 (q, *J* = 2.5 Hz), 55.6; ^19^F NMR (282 MHz, CDCl_3_): *δ* −67.23; HRMS (ESI) *m*/*z* [M+H]^+^ calcd for **C_17_H_12_F_3_N_4_OS**: 377.0678; found: 377.0676.

4-[(4-Fluorophenyl)sulfanyl]-2-(trifluoromethyl)pyrido[2′,3′:3,4]pyrazolo[1,5-*a*]pyrimidine **(7c)**.

Compound **7c** was obtained as a yellow solid with a yield of 56%; m.p. 221–223 °C; ^1^H NMR (300 MHz, DMSO-*d*_6_): *δ* 9.06 (dd, *J* = 4.2, 1.5 Hz, 1H), 8.87 (dd, *J* = 8.1, 1.5 Hz, 1H), 7.96 (dd, *J* = 8.7, 5.4 Hz, 2H), 7.59 (t, *J* = 8.7 Hz, 2H), 7.50 (dd, *J* = 8.1, 4.2 Hz, 1H), 6.81 (s, 1H); ^13^C NMR (75 MHz, DMSO-*d*_6_): *δ* 164.6 (d, *J* = 249.3 Hz), 160.7, 155.9, 153.0, 152.9, 141.8 (q, *J* = 35.8 Hz), 141.4, 139.1 (d, *J* = 9.1 Hz), 131.5, 121.3 (q, *J* = 271.3 Hz), 120.8 (d, *J* = 3.1 Hz), 118.9, 118.8, 118.6, 106.5, 104.4 (q, *J* = 2.5 Hz); ^19^F NMR (282 MHz, DMSO-*d*_6_): *δ* −66.0.1, −107.87; HRMS (ESI) *m*/*z* [M+H]^+^ calcd for **C_16_H_9_F_4_N_4_S**: 365.0478; found: 365.0477.

### 3.7. General Procedure for the Synthesis of 7-Alkoxy (or 7-Phenoxy)-2-trifluoromethyl Pyrimido[1,2-b]indazole Derivatives ***8a–c***

A solution of the corresponding alcohol or phenol (1.1 equiv.) in the mixture of acetonitrile was degassed through argon bubbling. Potassium carbonate (1.3 equiv.) was then added and the mixture was stirred at room temperature for 15 min. Then, compound **3f** (or **3h**) (1 equivalent) was added and the reaction mixture was refluxed for 2–3 h. After the completion of the reaction monitored by TLC analysis (eluent: petroleum ether/ethyl acetate, 8:2), the solvent was evaporated under reduced pressure, and the crude residue was purified using silica gel column chromatography to give the desired products **8a–c**. (^1^H-NMR, ^19^F-NMR and ^13^C-NMR of compounds **8a–c** are shown in Supplementary Materials).

7-Ethoxy-9-(trifluoromethyl)pyrido[3′,2′:3,4]pyrazolo[1,5-*a*]pyrimidine **(8a)**.

The purification of the crude product by chromatography on silica gel was carried out using (PE/EtOAc: 8/2) to afford **8a** as a white crystal with a yield of 69%; m.p. 189–191 °C; ^1^H NMR (300 MHz, CDCl_3_): *δ* 8.87 (dd, *J* = 3.9, 1.2 Hz, 1H), 8.32 (dd, *J* = 8.7, 1.2 Hz, 1H), 7.61 (dd, *J* = 8.7, 3.9 Hz, 1H), 7.01 (s, 1H), 4.75 (q, *J* = 7.2 Hz, 2H), 1.79 (t, *J* = 7.2 Hz, 3H); ^13^C NMR (75 MHz, CDCl_3_): *δ* 155.3, 147.9, 146.0 (q, *J* = 37.3 Hz), 145.6, 143.6, 130.3, 125.3, 125.2, 120.8 (q, *J* = 272.0 Hz), 88.3 (q, *J* = 2.1 Hz), 68.2, 14.2; ^19^F NMR (282 MHz, CDCl_3_): *δ* −67.24; HRMS (ESI) *m*/*z* [M+H]^+^ calcd for **C_12_H_10_F_3_N_4_O**: 283.0801; found: 283.0799.

7-Methoxy-9-(trifluoromethyl)pyrazino[2′,3′:3,4]pyrazolo[1,5-*a*]pyrimidine **(8b)**.

The purification of the crude product by chromatography on silica gel was carried out using (PE/EtOAc: 8/2) to afford **8b** as a brown solid with a yield of 60%; m.p. 145–147 °C; ^1^H NMR (300 MHz, CDCl_3_): *δ* 8.99 (d, *J* = 2.1 Hz, 1H), 8.84 (d, *J* = 2.1 Hz, 1H), 7.16 (s, 1H), 4.52 (s, 3H); ^13^C NMR (75 MHz, CDCl_3_): *δ* 156.4, 154.8, 149.6, 147.5 (q, *J* = 37.2 Hz), 143.0, 142.9, 123.4, 120.5 (q, *J* = 274.0 Hz), 89.2 (q, *J* = 2.2 Hz), 58.5; ^19^F NMR (282 MHz, CDCl_3_): *δ* −67.47; HRMS (ESI) *m*/*z* [M+H]^+^ calcd for **C_10_H_7_F_3_N_5_O**: 270.0597; found: 270.0595.

7-Phenoxy-9-(trifluoromethyl)pyrazino[2′,3′:3,4]pyrazolo[1,5-*a*]pyrimidine **(8c)**.

The purification of the crude product using chromatography on silica gel was carried out using (PE/EtOAc: 9/1) to afford **8c** as a light brown solid with a yield of 72%; m.p. 183–185 °C; ^1^H NMR (300 MHz, CDCl_3_): *δ* 9.02 (d, *J* = 1.8 Hz, 1H), 8.87 (d, *J* = 1.8 Hz, 1H), 7.64 (t, *J* = 8.1 Hz, 2H), 7.53 (t, *J* = 7.5 Hz, 1H), 7.41 (d, *J* = 7.5 Hz, 2H), 6.85 (s, 1H); ^13^C NMR (75 MHz, CDCl_3_): *δ* 156.1, 155.0, 151.4, 149.8, 147.2 (q, *J* = 37.3 Hz), 143.4, 143.1, 131.2 (2C), 128.3, 123.5, 120.8 (2C), 120.3 (q, *J* = 273.8 Hz), 91.8 (q, *J* = 2.3 Hz); ^19^F NMR (282 MHz, CDCl_3_): *δ* −67.43; HRMS (ESI) *m*/*z* [M+H]^+^ calcd for **C_15_H_9_F_3_N_5_O**: 332.0753; found: 332.0750

## 4. Conclusions

We have developed a simple and general strategy for the regioselective synthesis of trifluoromethylated pyrimido[1,2-*b*]indazole derivatives starting from commercially available compounds. This strategy was based first on the condensation of the 3-amino indazole derivatives with ethyl 4,4,4-trifluoro-3-oxobutanoate providing trifluoromethylated pyrimido[1,2-*b*]indazol-4(1*H*)-one derivatives with good yields. The functionalization of the corresponding chlorinated derivatives, using the Suzuki–Miyaura coupling reaction and aromatic nucleophilic substitution, provided access to a new library of trifluoromethylated pyrimido[1,2-*b*]indazole derivatives.

The process shows promise as a valuable tool for constructing complex fluorinated heterocycles. This versatile strategy is applied to the production of a wide range of compounds currently under biological evaluation, in particular as novel monoamine oxidase (MAO) inhibitors.

## Data Availability

Data are contained within the article and Supplementary Materials.

## Supplementary Materials

The following supporting information can be downloaded at: https://www.mdpi.com/article/10.3390/molecules29010044/s1, ^1^H-NMR, ^19^F-NMR and ^13^C-NMR of compounds **2a–h**, **3a–h**, **4a–h**, **5a–q**, **6a–g, 7a–c** and **8a–c**.
