# Supplementary material for: Synthesis of Trifluoromethylated Pyrimido[1,2-b]indazole Derivatives through the Cyclocondensation of 3-Aminoindazoles with Ketoester and Their Functionalization via Suzuki-Miyaura Cross-Coupling and SNAr Reactions
Source: Molecules. 2023 Dec 20;29(1):44. doi: 10.3390/molecules29010044 (PMC10779788; doi:10.3390/molecules29010044)

Copie of ^{1}H, ^{13}C and ^{19}F NMR of products **2a-h**, **3a-h**, **4a-h**, **5a-q**, **6a-g**, **7a-c**, **8a-c** ……

2-Trifluoromethyl-1*H*-pyrimido[1,2-*b*]indazol-4-one (2a)

^{1}H NMR (300 MHz, DMSO-d$_6$)

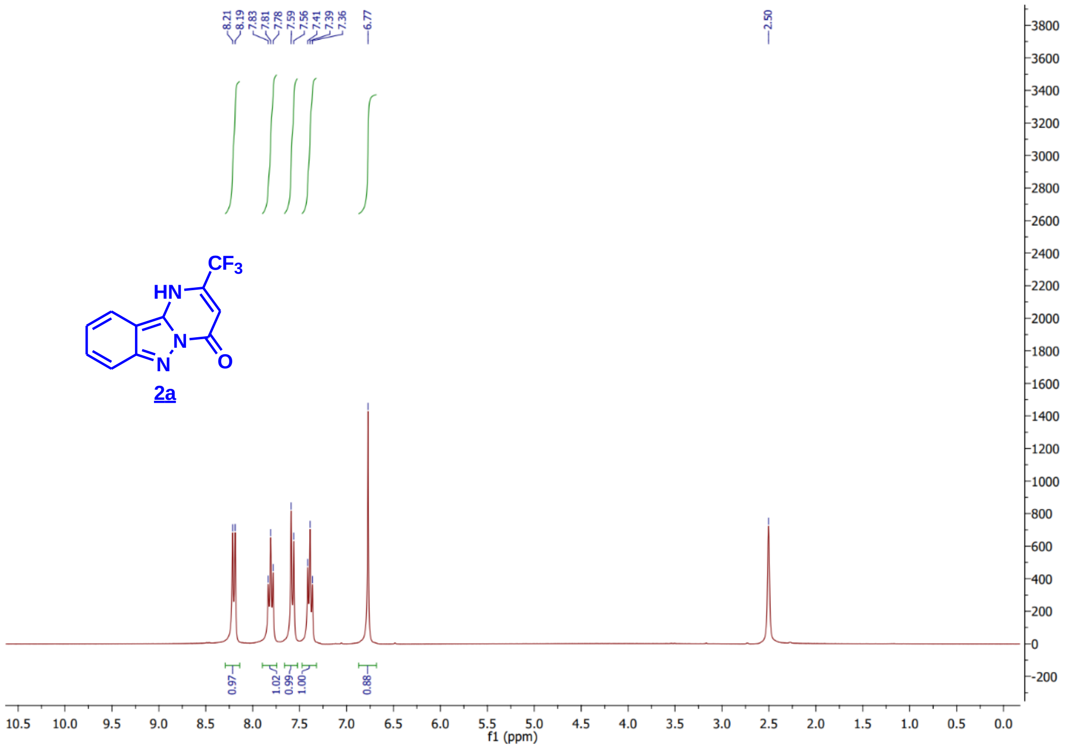

^{19}F NMR (282 MHz, DMSO-d$_6$)

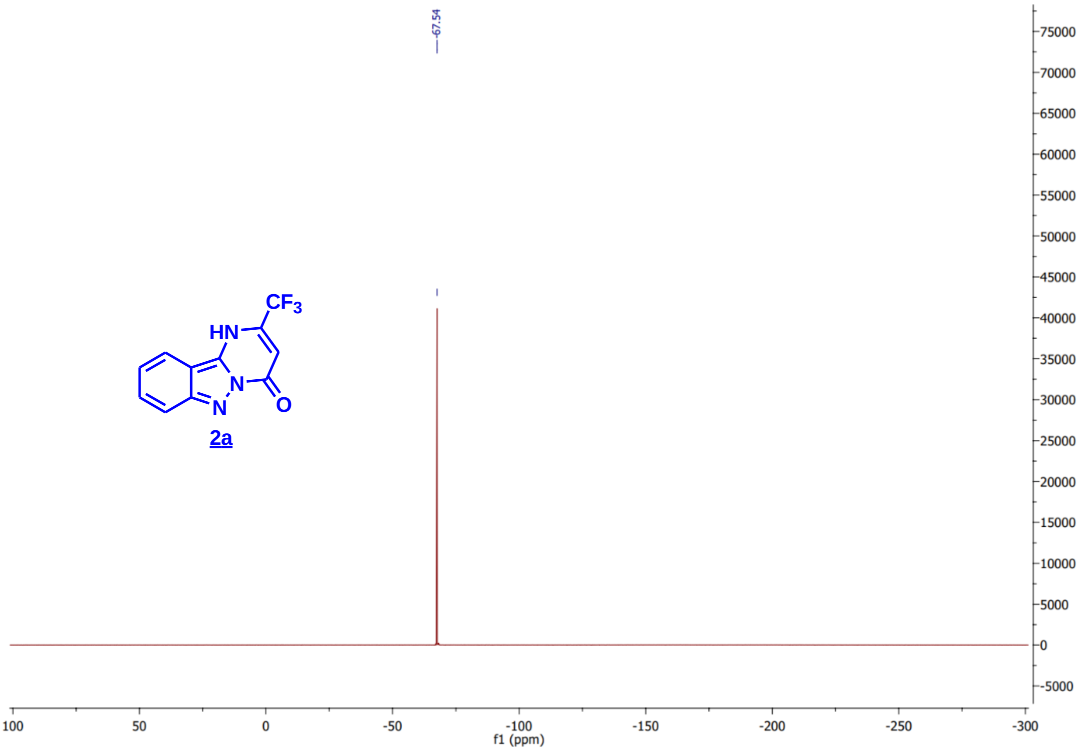

^{13}C NMR (75 MHz, DMSO-d6)

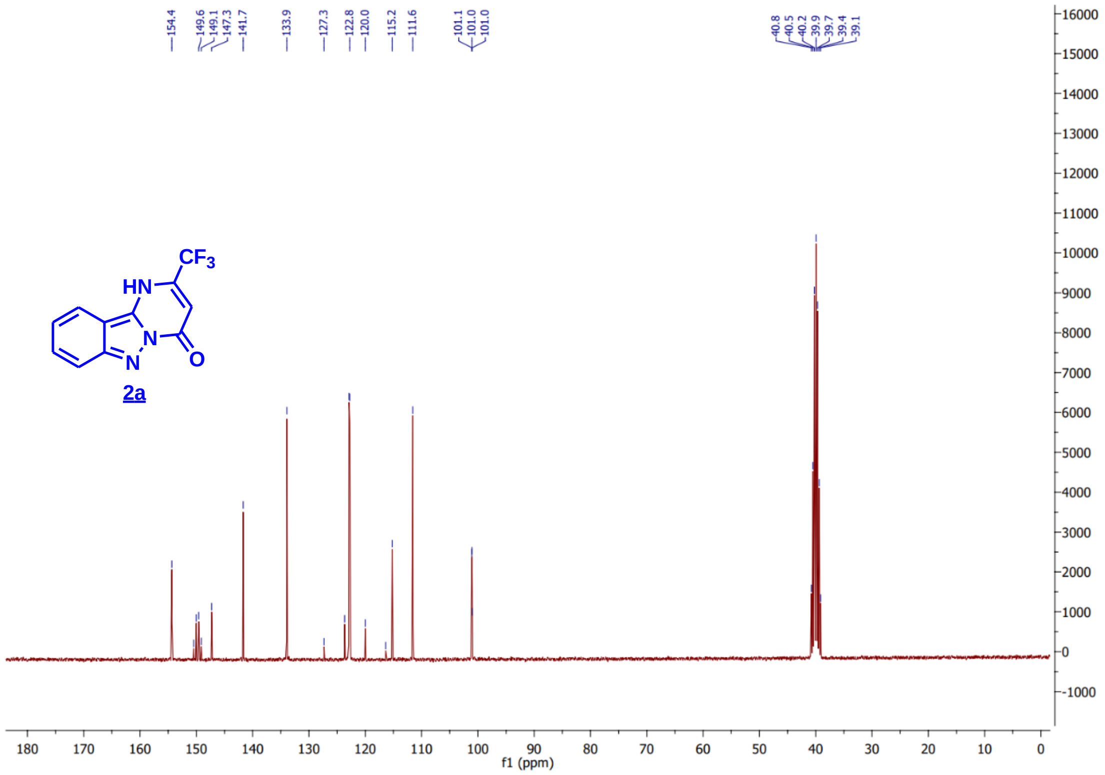

10-Chloro-2-trifluoromethyl-1*H*-pyrimido[1,2-*b*]indazol-4-one (2b)

^{1}H NMR (300 MHz, DMSO-d6)

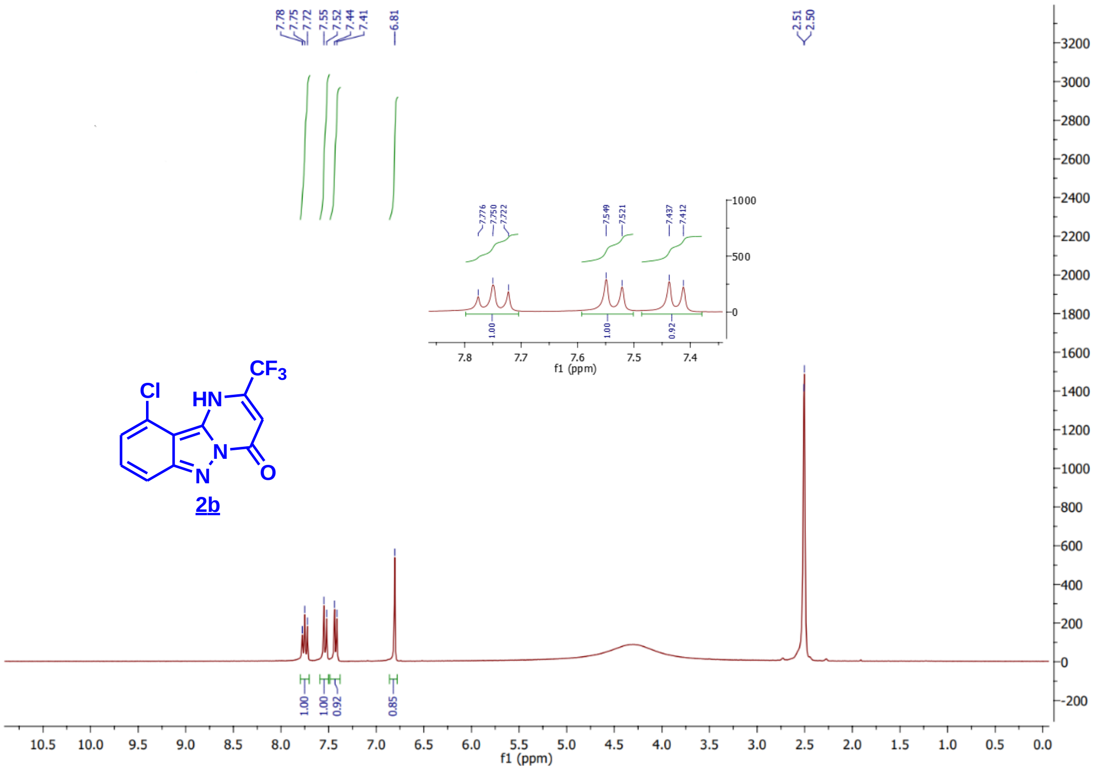

^{19}F NMR (282 MHz, DMSO-d6)

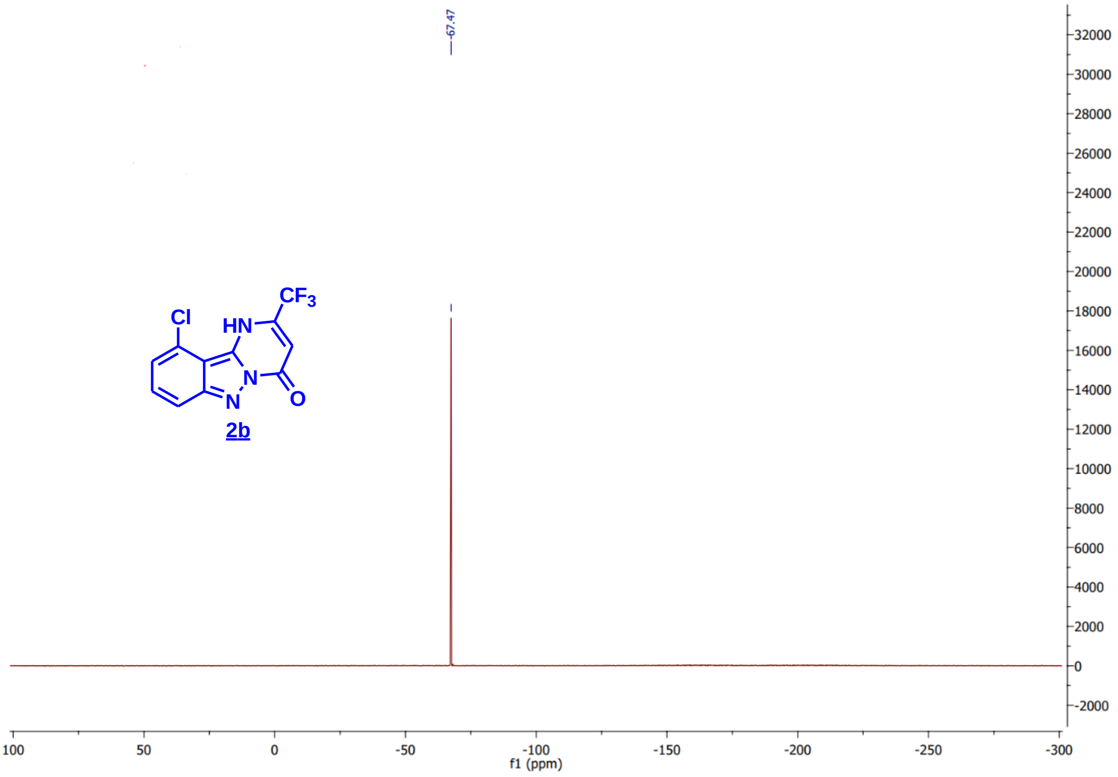

^{13}C NMR (75 MHz, DMSO-d6)

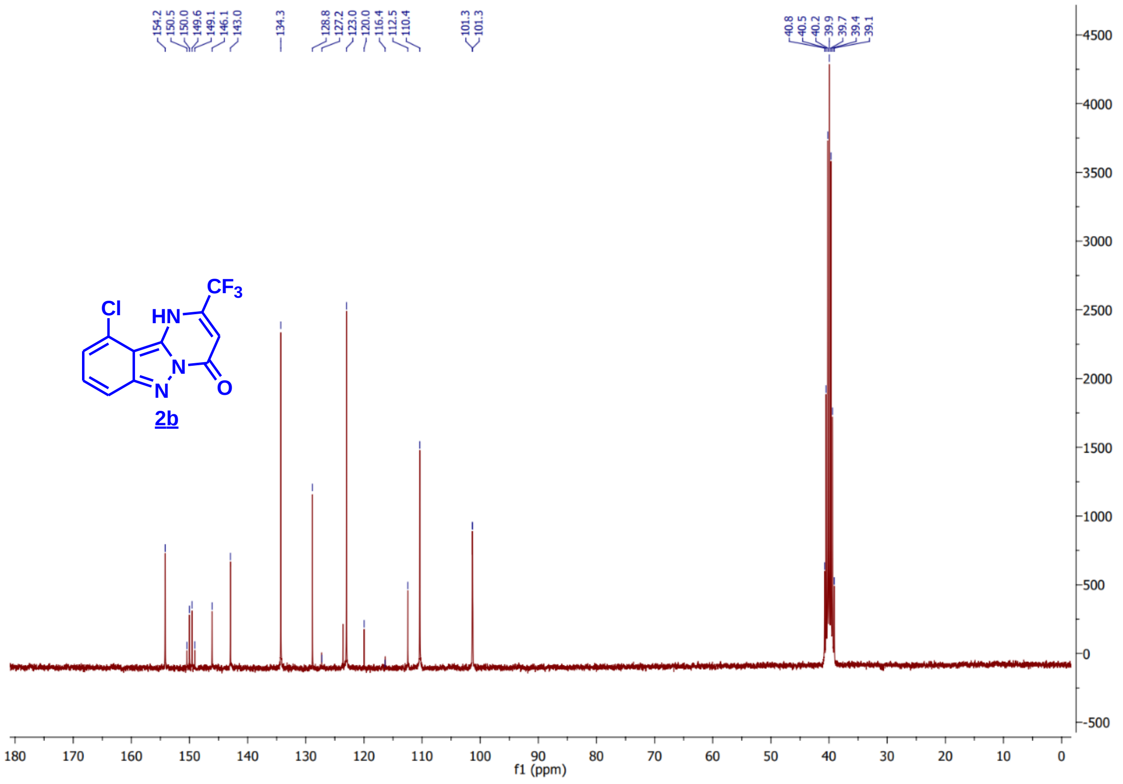

10-Fluoro-2-trifluoromethyl-1*H*-pyrimido[1,2-*b*]indazol-4-one (2c)

^{1}H NMR (300 MHz, DMSO-d$_6$)

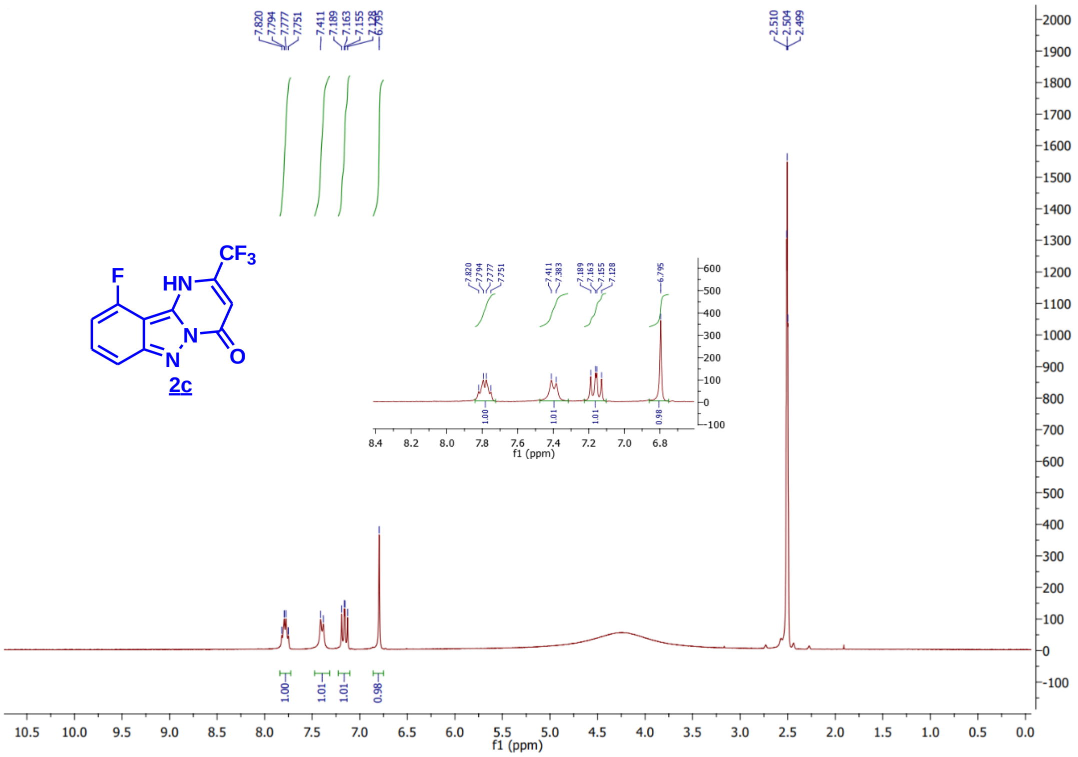

^{19}F NMR (282 MHz, DMSO-d$_6$)

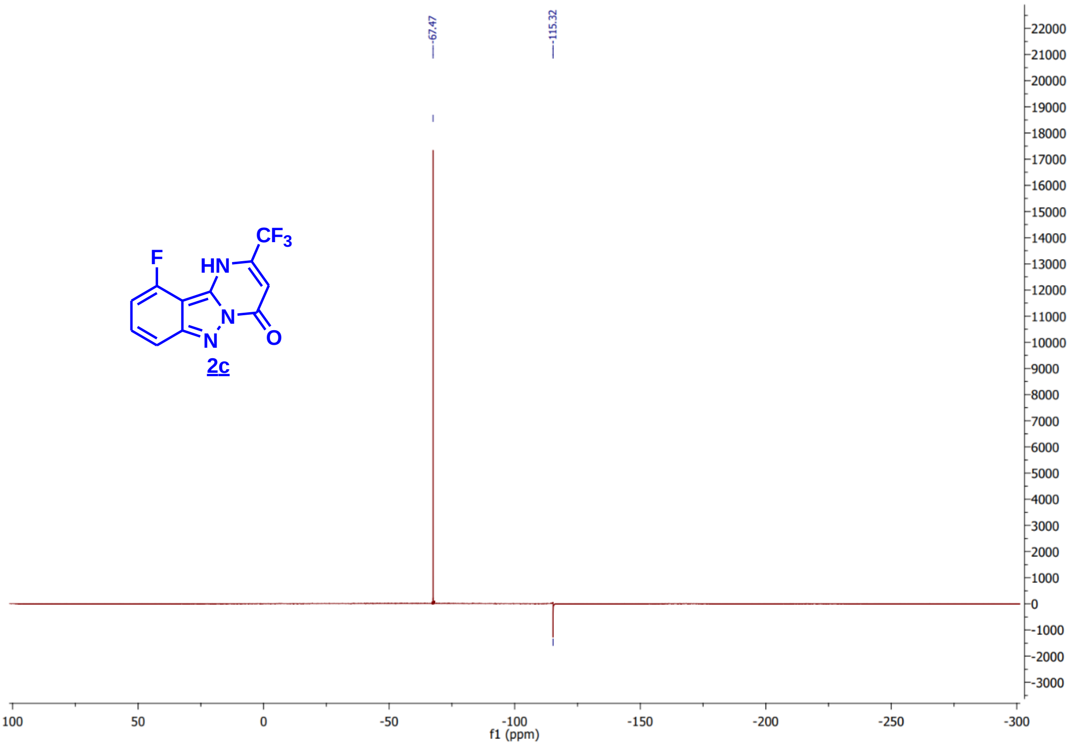

^{13}C NMR (75 MHz, **THF-d$_8$**)

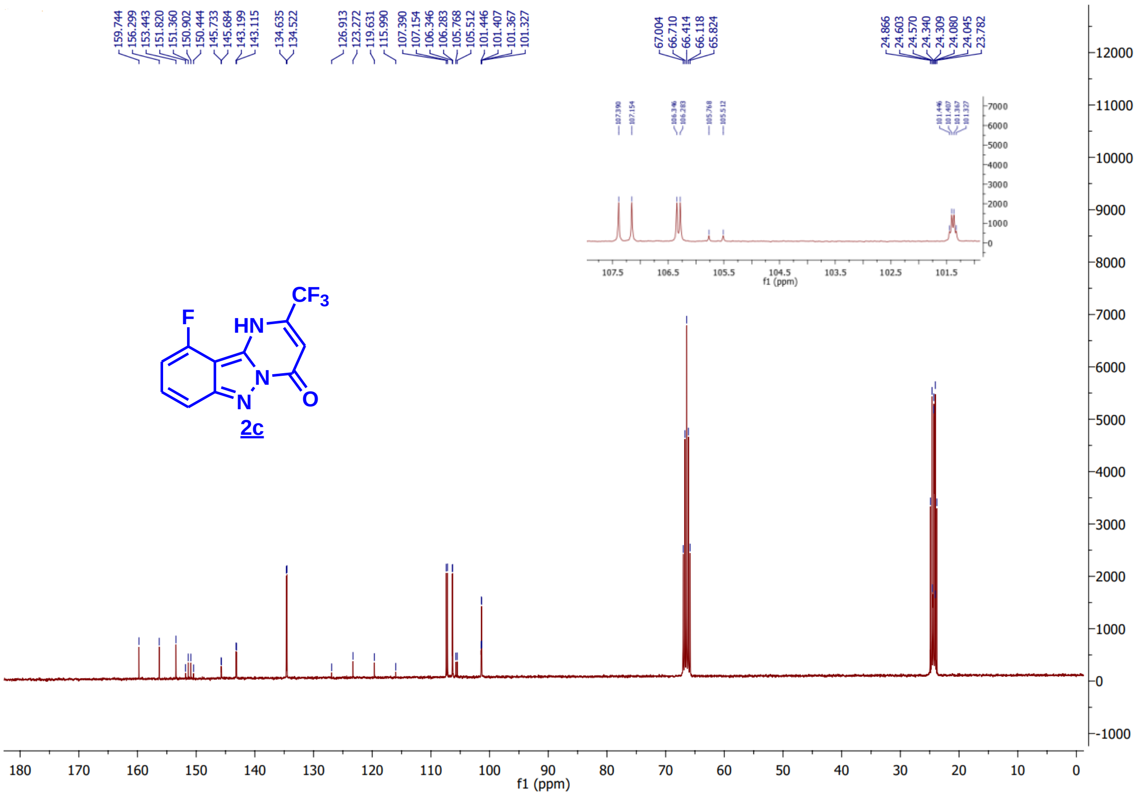

2,8-Bis-trifluoromethyl-1H-pyrimido[1,2-b]indazol-4-one (2d)

^{1}H NMR (300 MHz, DMSO-d$_6$)

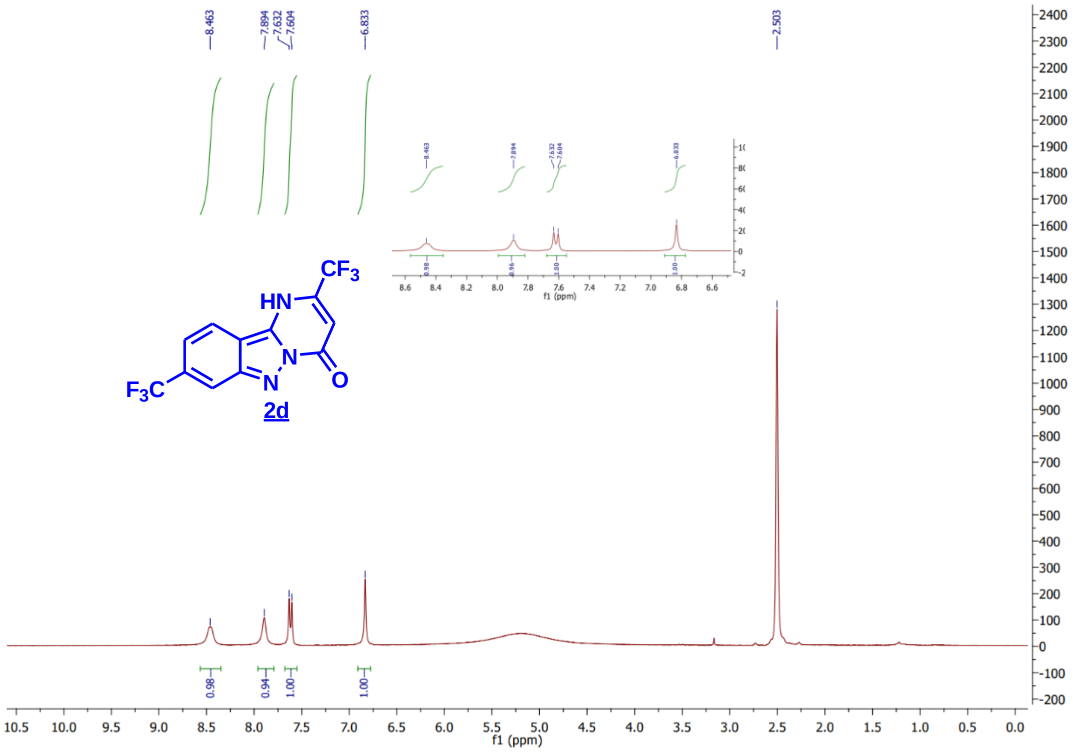

^{19}F NMR (282 MHz, DMSO-d$_6$)

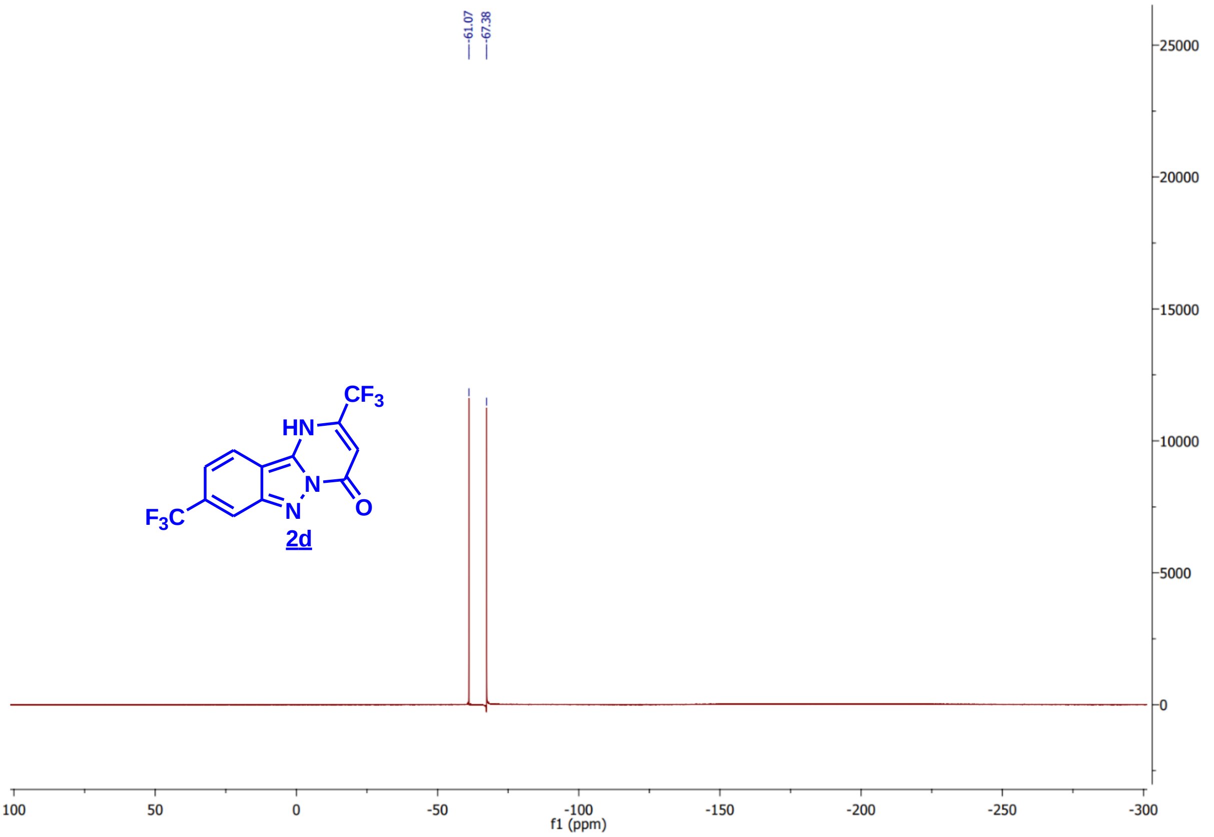

^{13}C NMR (75 MHz, THF-d$_8$)

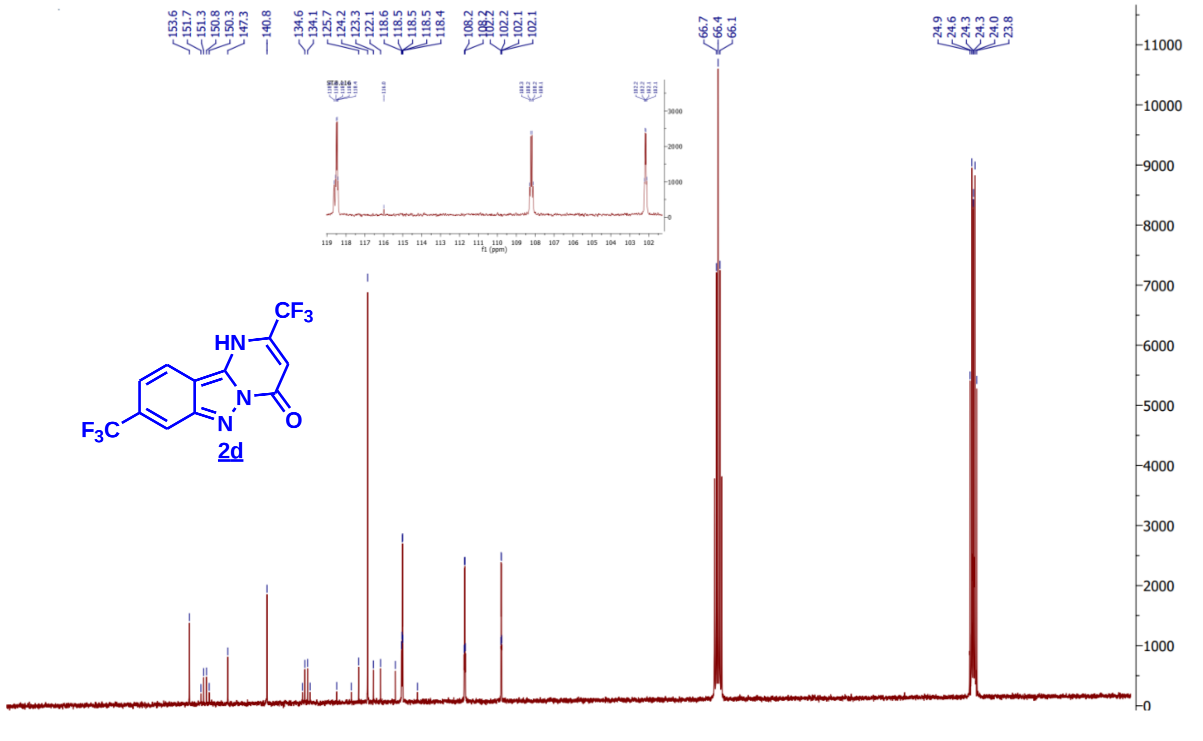

9-Nitro-2-trifluoromethyl-1H-pyrimido[1,2-b]indazol-4-one (2e)

^{1}H NMR (300 MHz, DMSO-d_6)

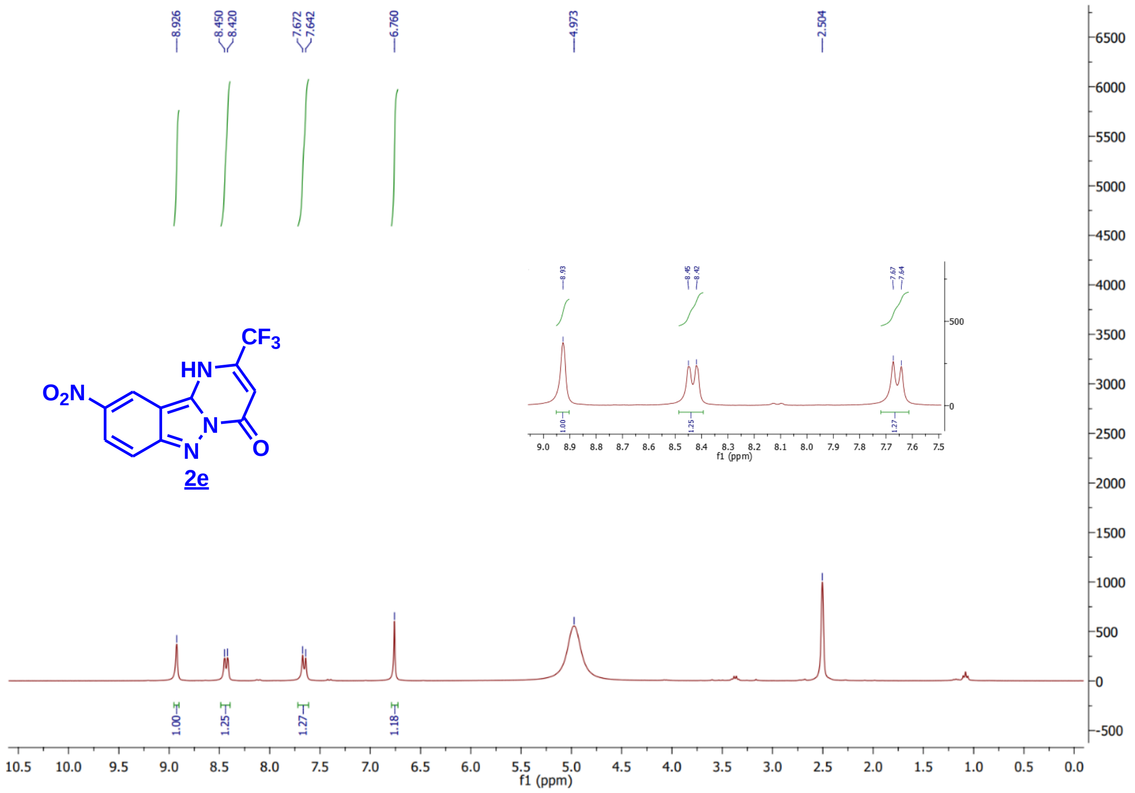

^{19}F NMR (282 MHz, DMSO-d_6)

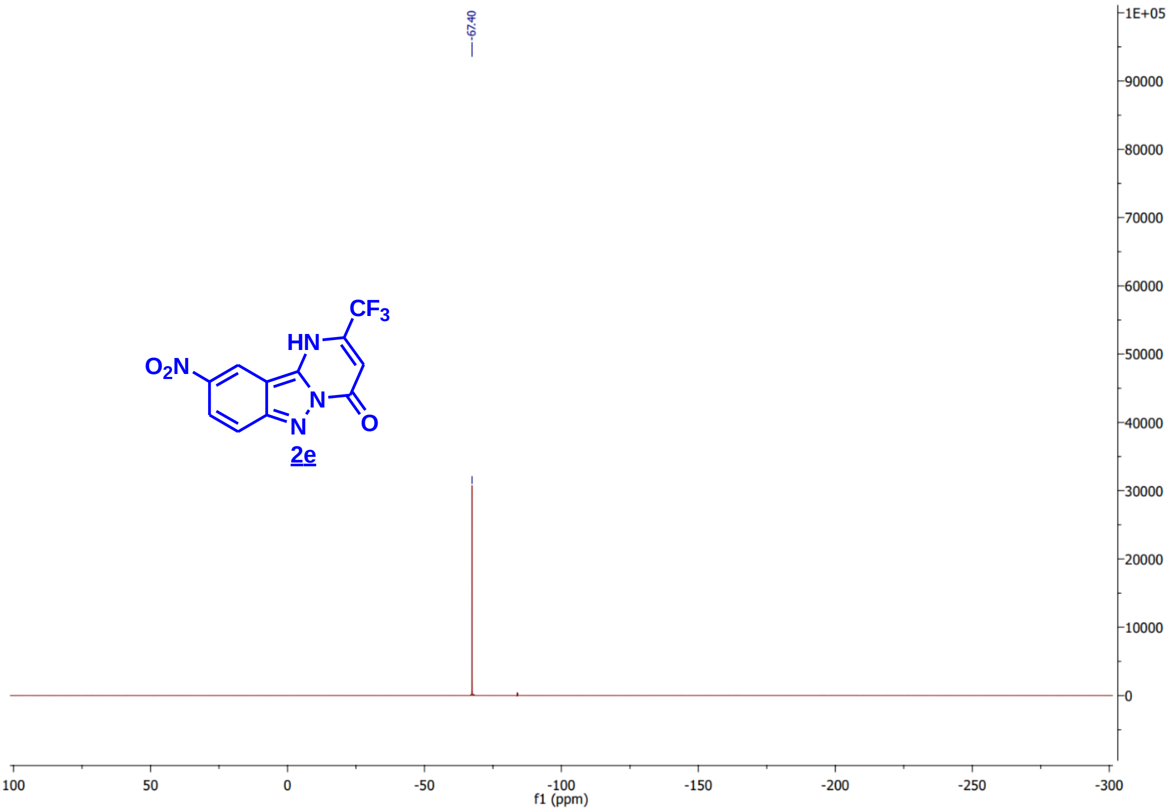

^{13}C NMR (75 MHz, THF-d$_8$)

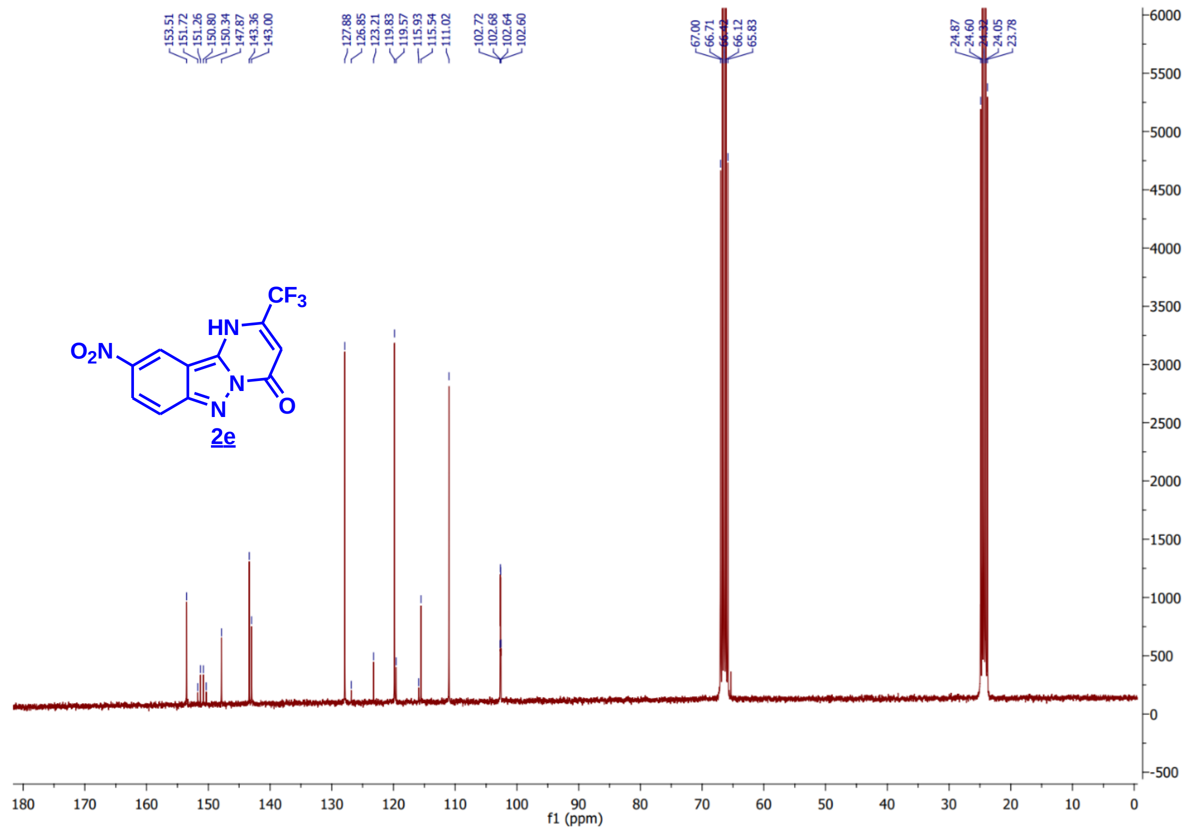

9-(trifluoromethyl)pyrido[3',2':3,4]pyrazolo[1,5-*a*]pyrimidin-7(10*H*)-one (2f)

^{1}H NMR (300 MHz, DMSO-d$_6$)

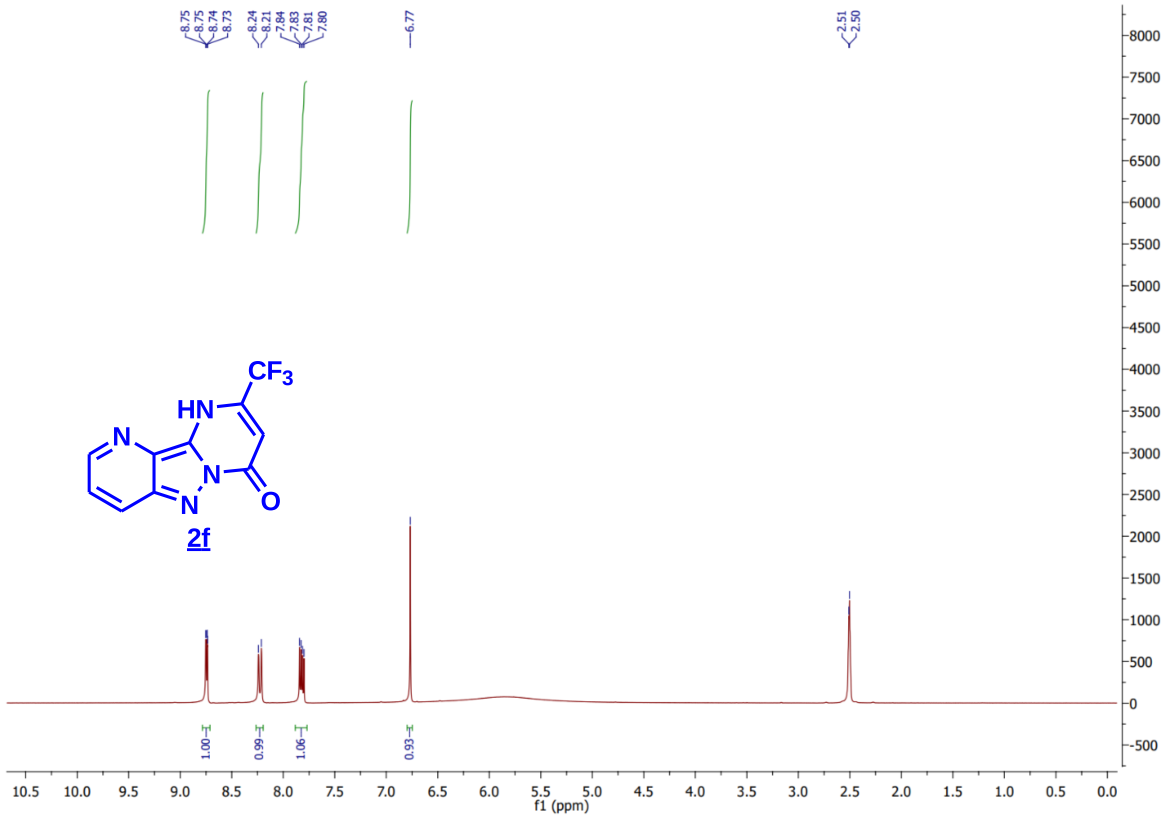

^{19}F NMR (282 MHz, DMSO-d6)

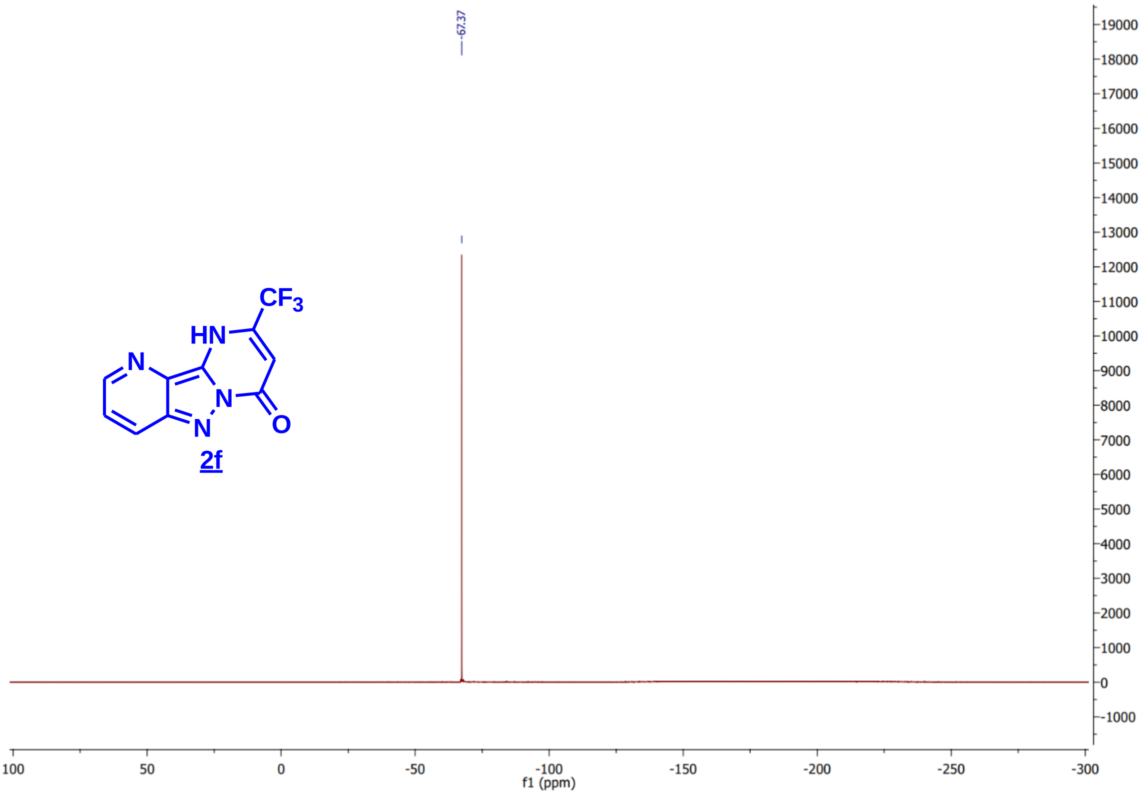

^{13}C NMR (75 MHz, DMSO-d6)

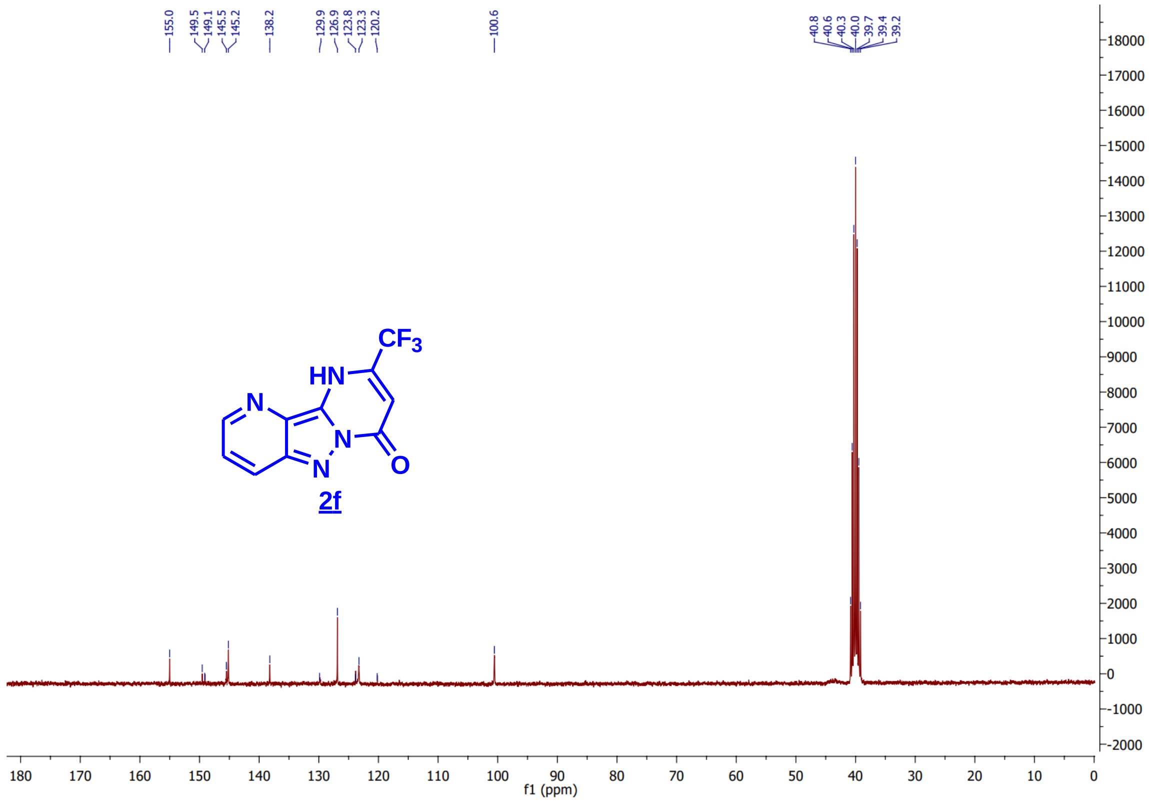

2-(trifluoromethyl)pyrido[2',3':3,4]pyrazolo[1,5-*a*]pyrimidin-4(1*H*)-one (2g)

^{1}H NMR (300 MHz, DMSO-d$_6$)

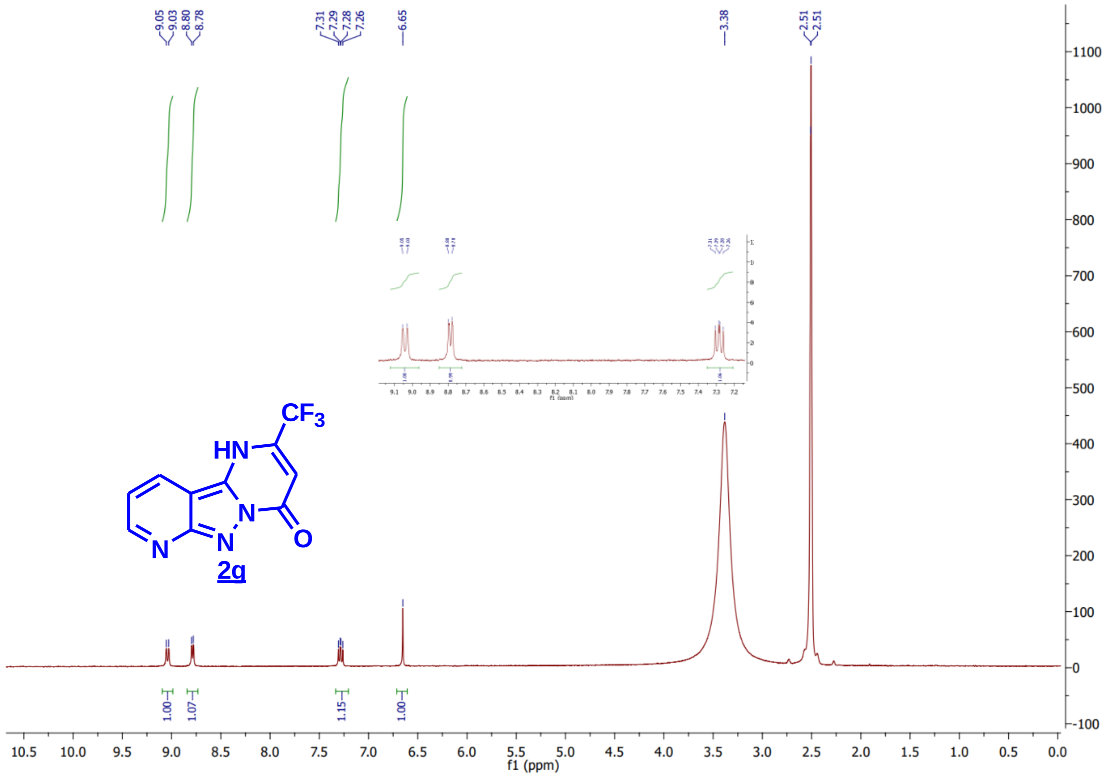

^{19}F NMR (282 MHz, DMSO-d$_6$)

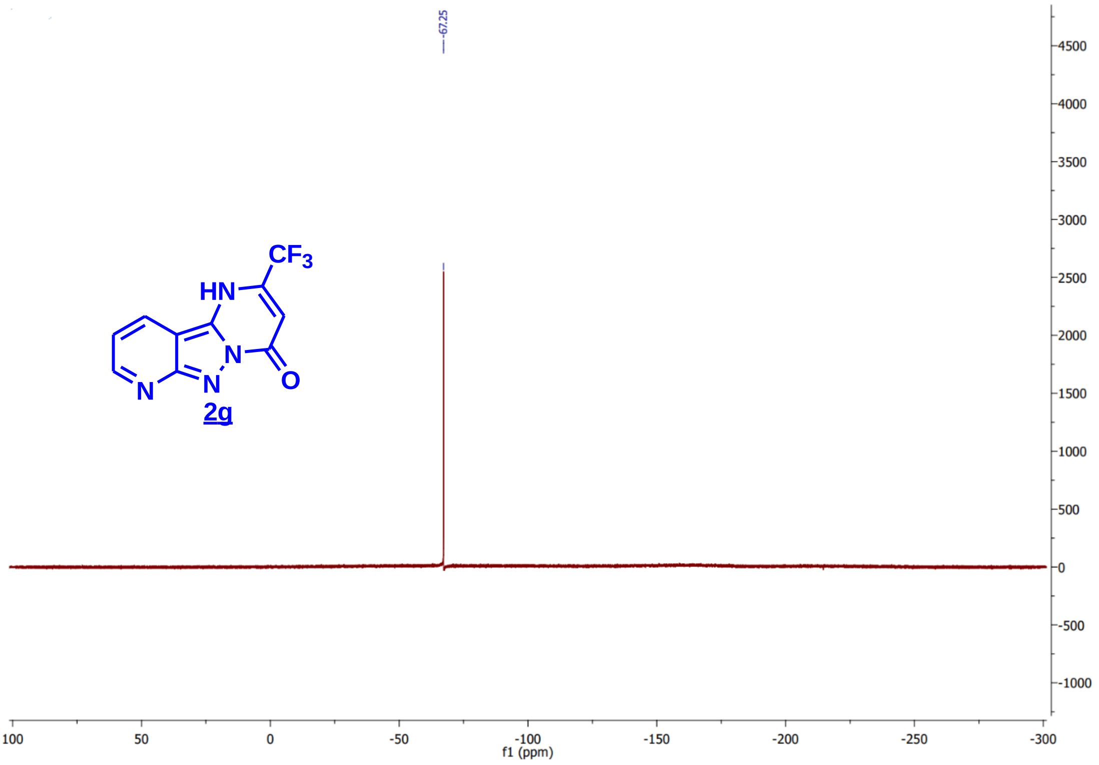

^{13}C NMR (75 MHz, $CDCl_3$ / TFA-d_1)

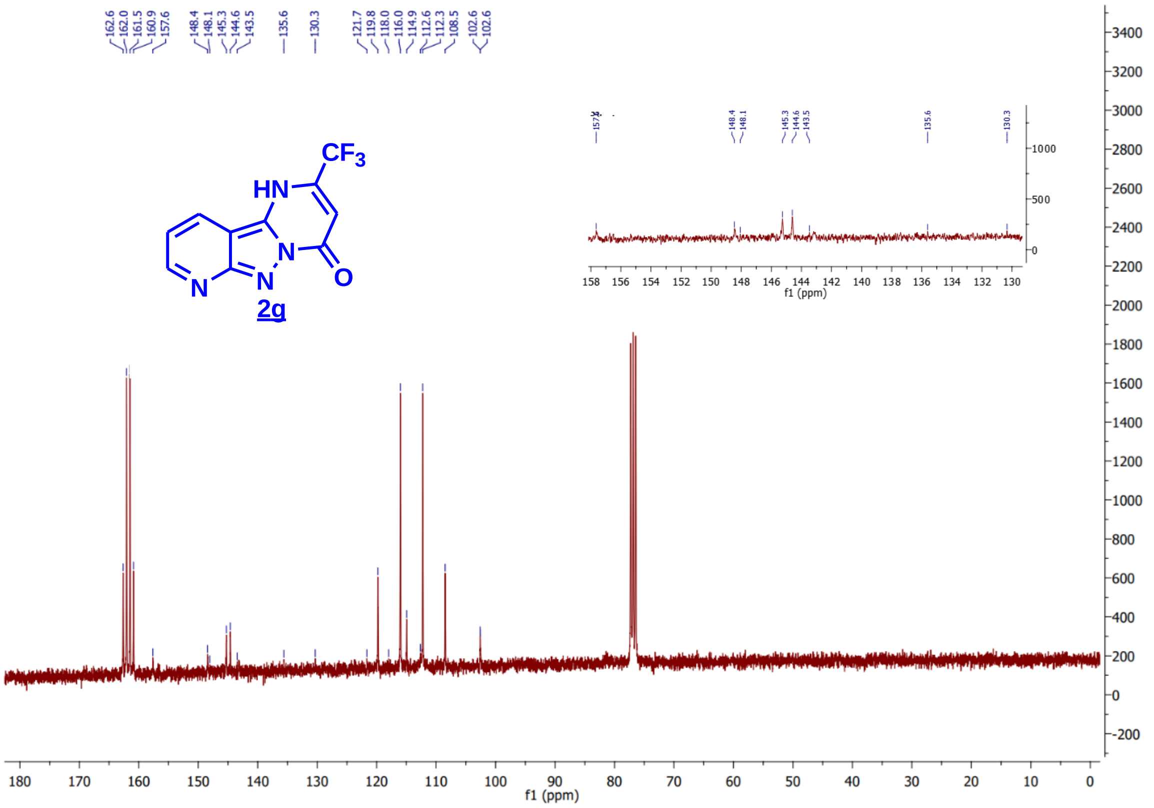

9-(trifluoromethyl)pyrazino[2',3':3,4]pyrazolo[1,5-*a*]pyrimidin-7(10*H*)-one (2h)

^{1}H NMR (300 MHz, DMSO-d_6)

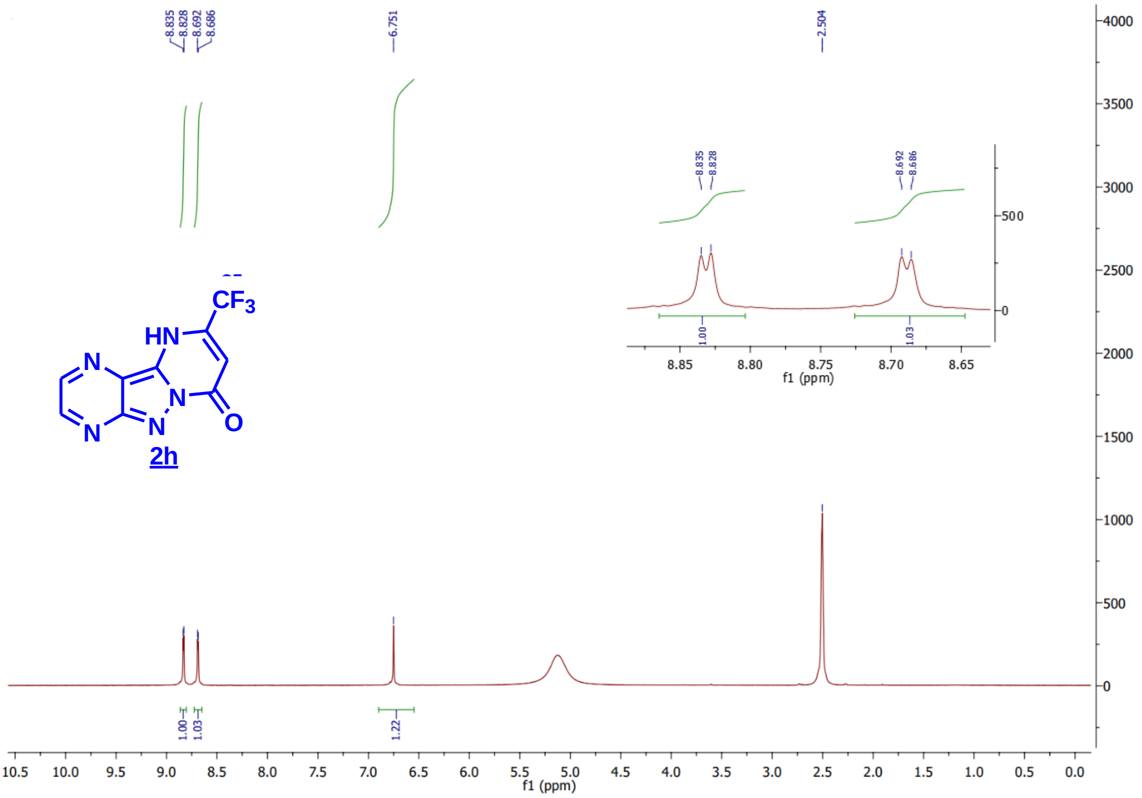

^{19}F NMR (282 MHz, DMSO-d6)

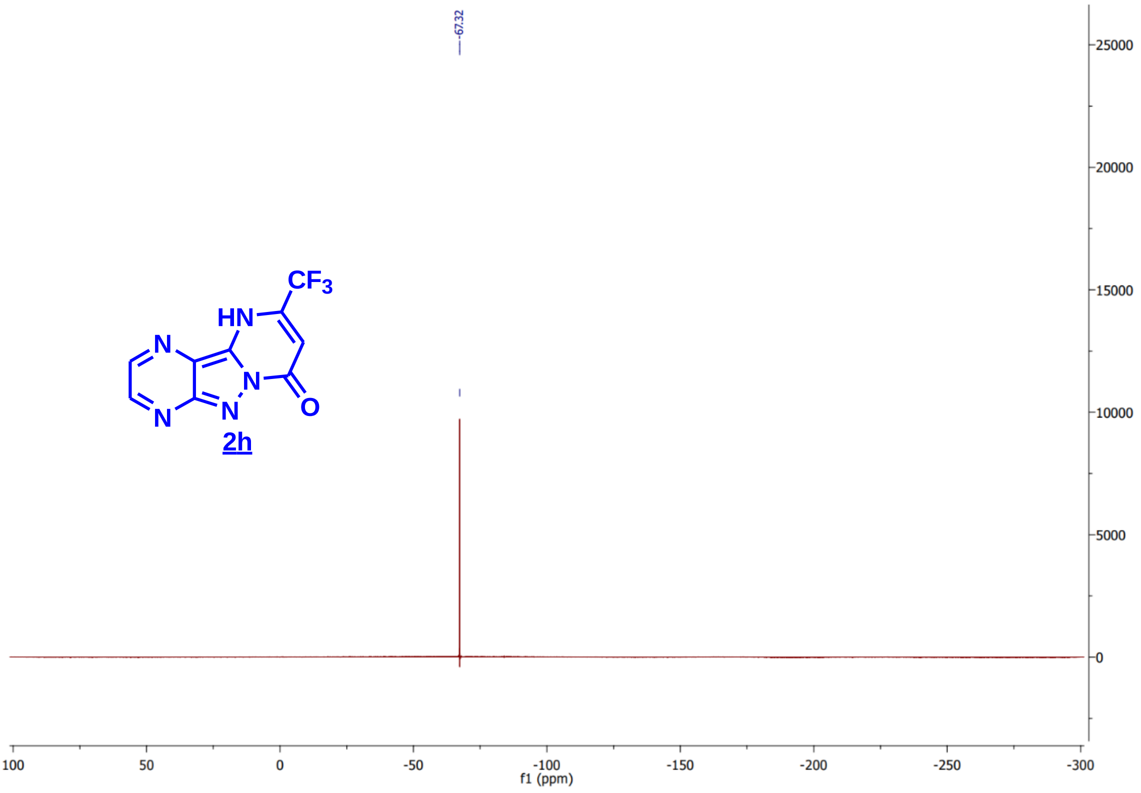

^{13}C NMR (75 MHz, THF-d8)

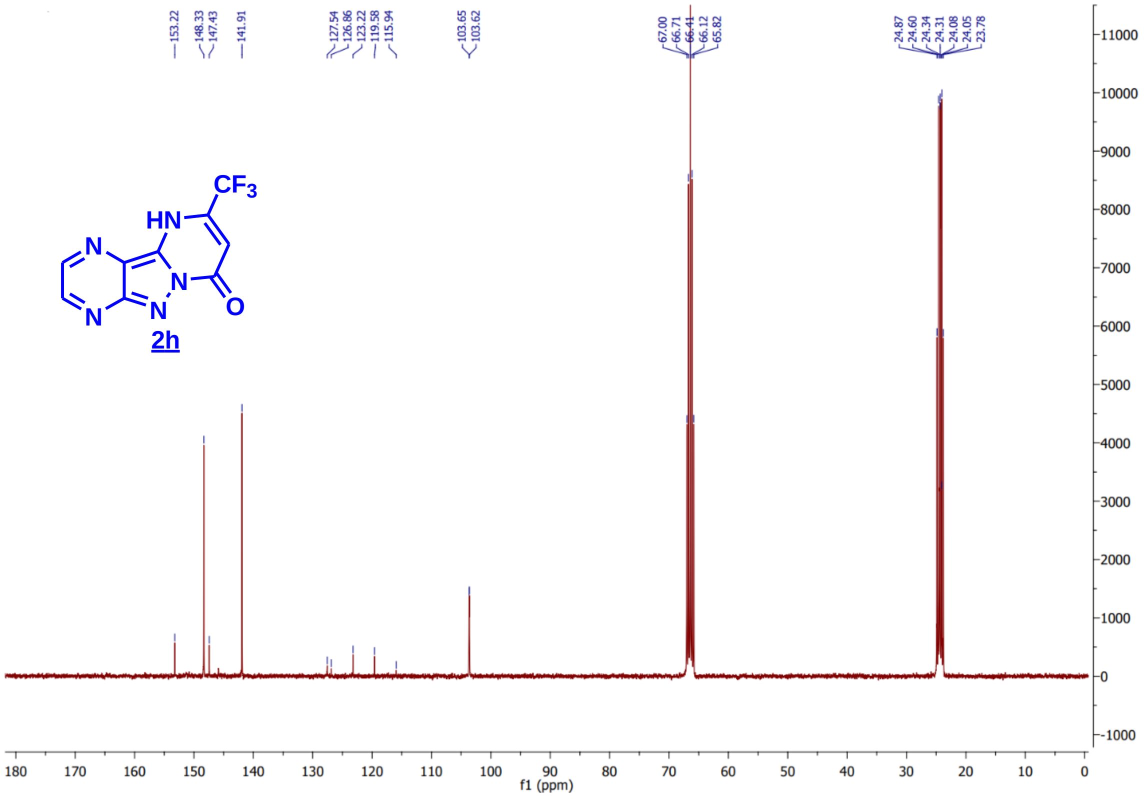

4-Chloro-2-trifluoromethyl-pyrimido[1,2-b]indazole (3a)

^{1}H NMR (300 MHz, $CDCl_3$)

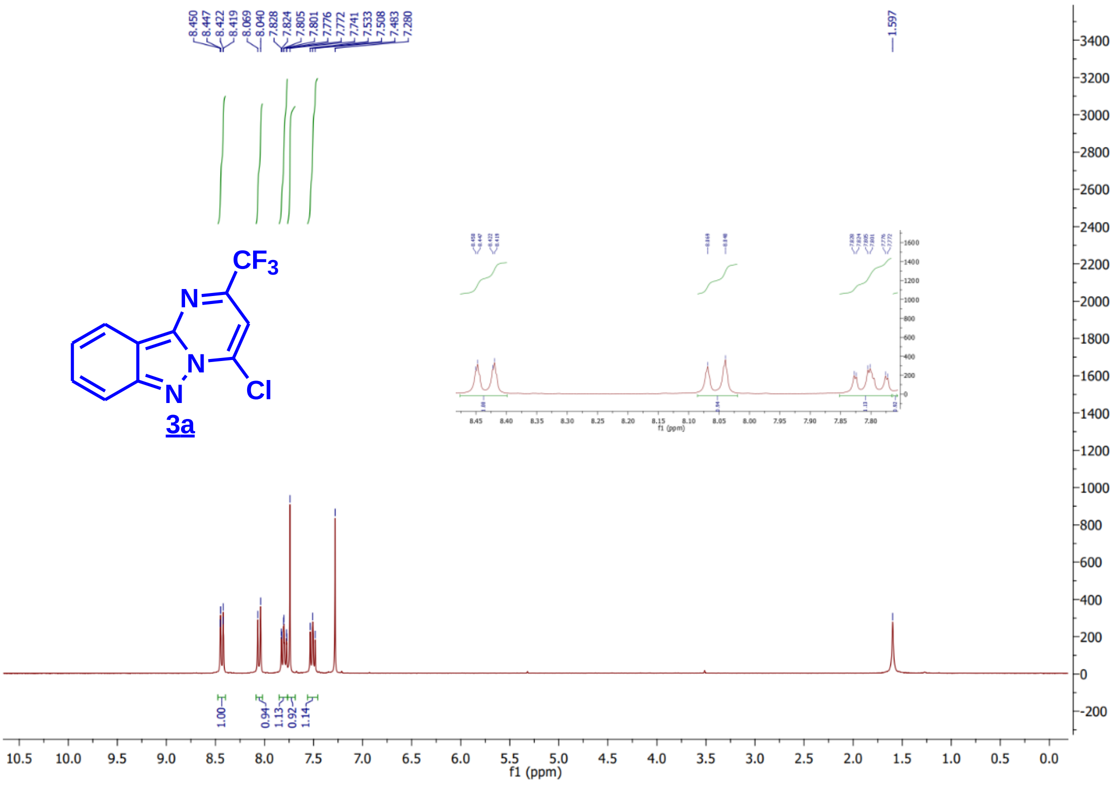

^{19}F NMR (282 MHz, $CDCl_3$)

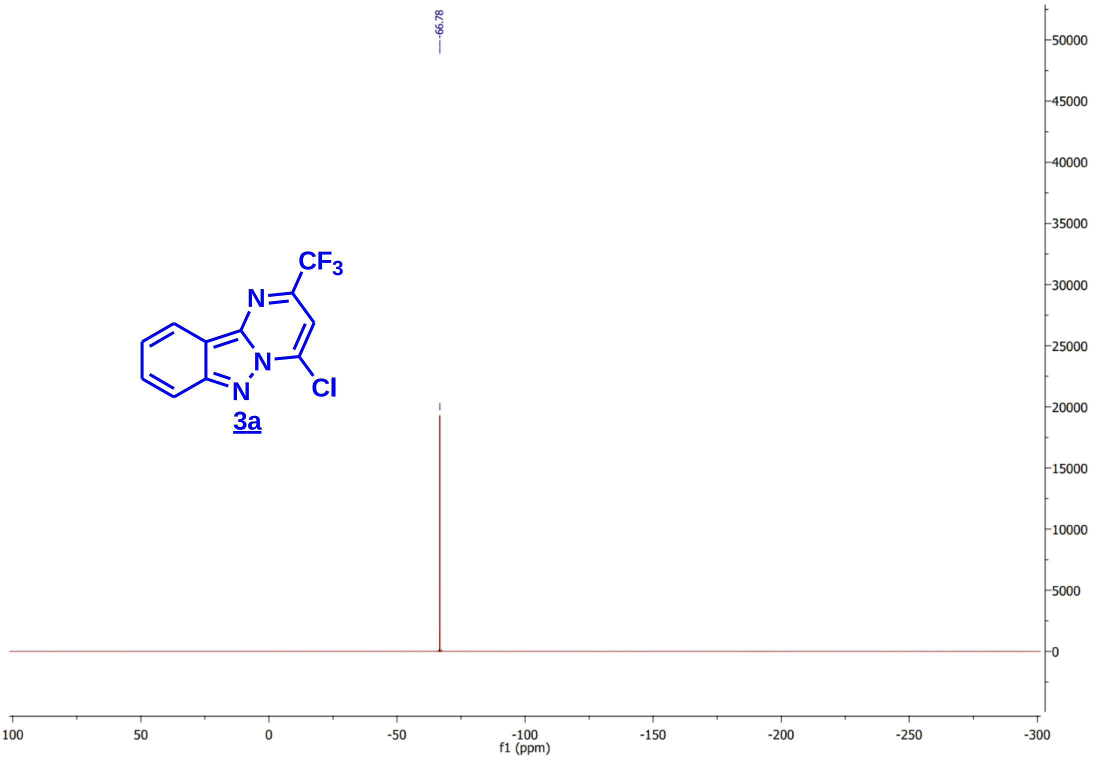

^{13}C NMR (75 MHz, $CDCl_3$)

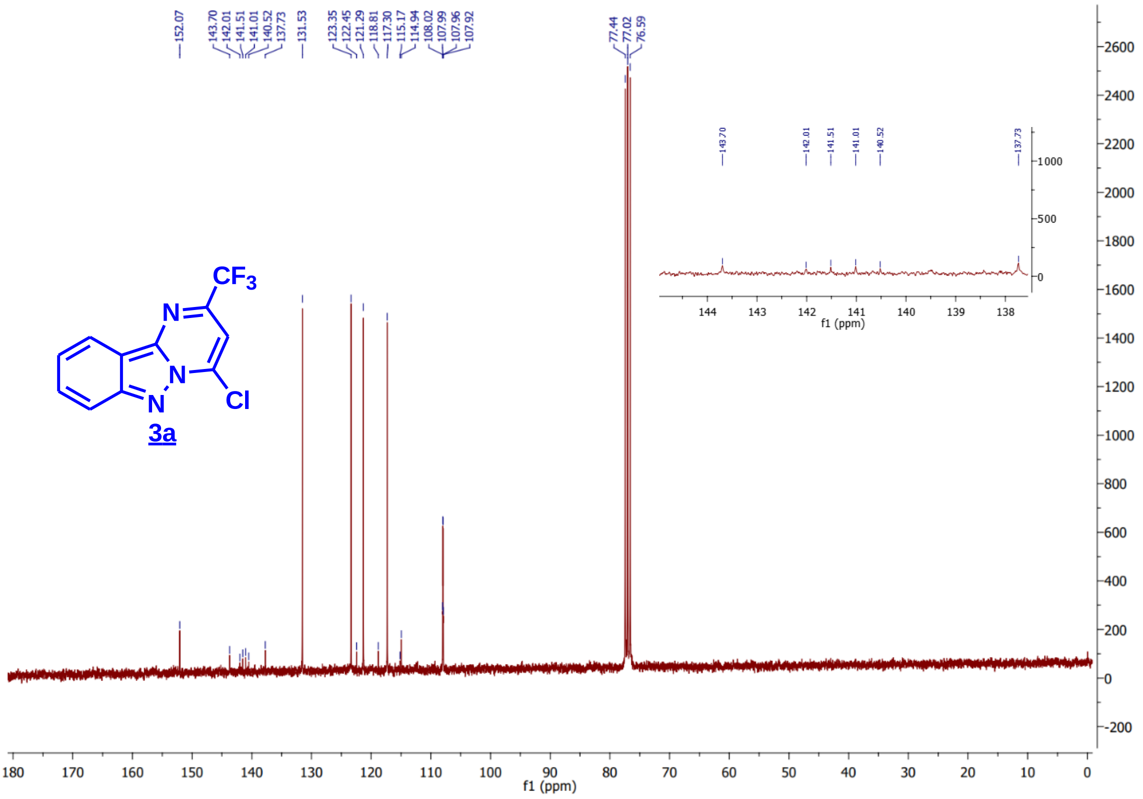

4,10-Dichloro-2-trifluoromethyl-pyrimido[1,2-b]indazole (3b)

^{1}H NMR (300 MHz, $CDCl_3$)

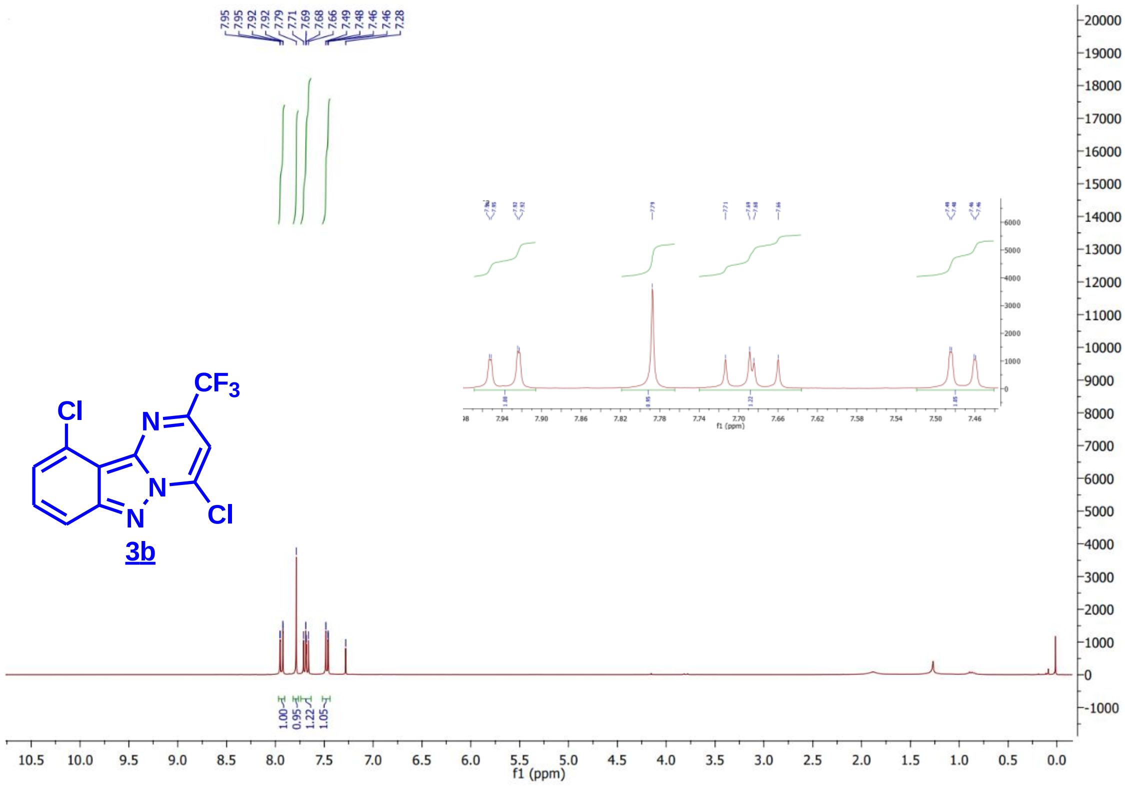

^{19}F NMR (282 MHz, $CDCl_3$)

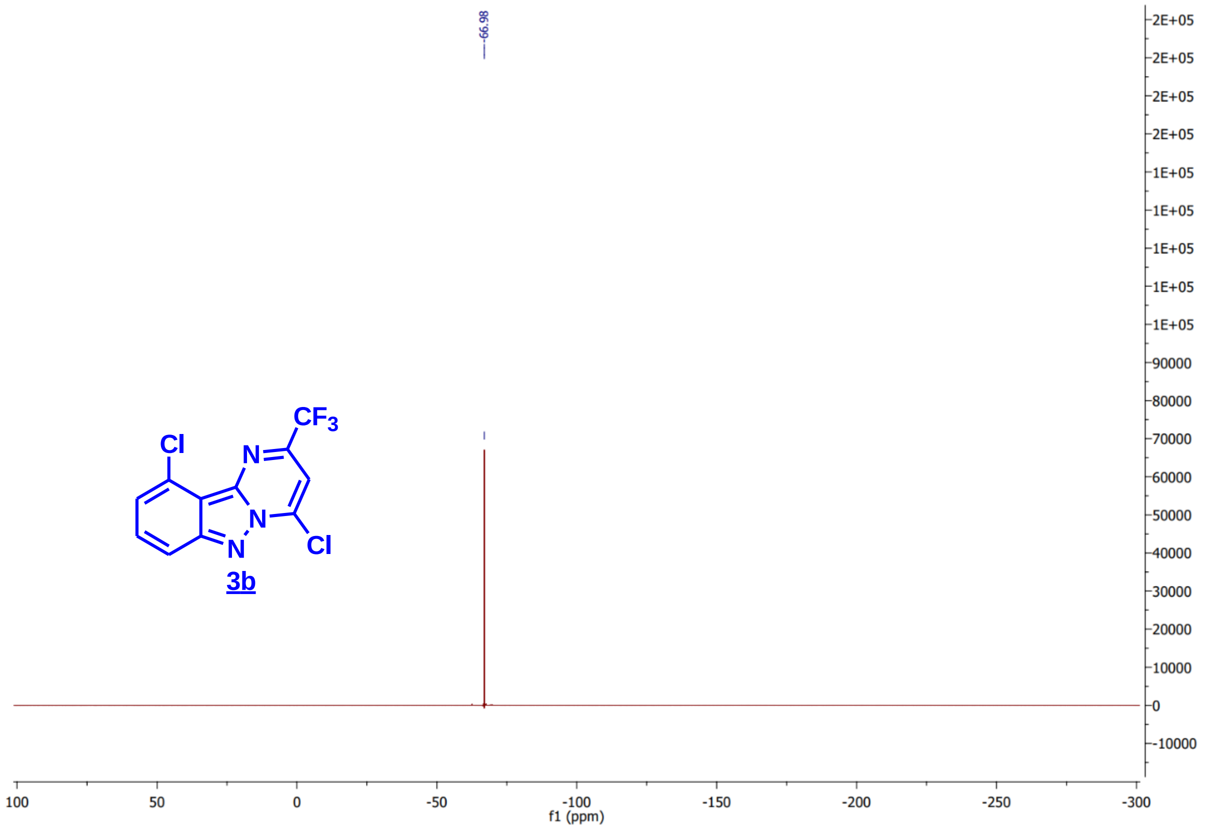

^{13}C NMR (75 MHz, $CDCl_3$)

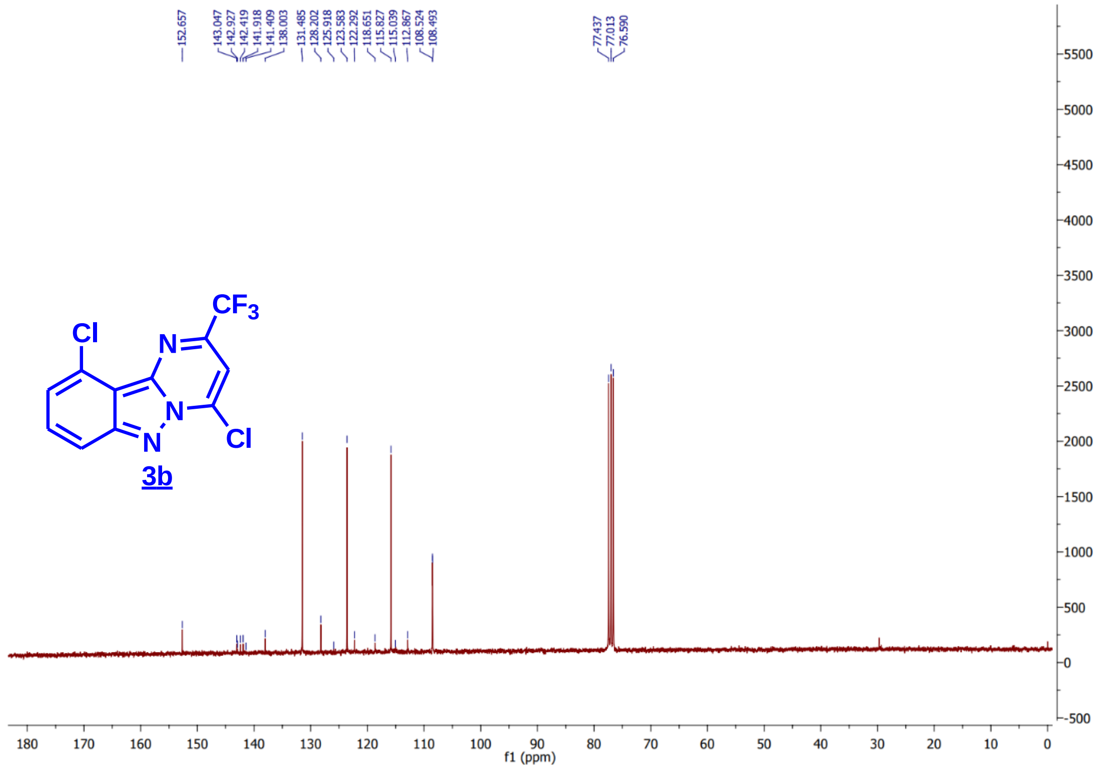

4-Chloro-10-fluoro-2-trifluoromethyl-pyrimido[1,2-b]indazole (3c)

^{1}H NMR (300 MHz, $CDCl_3$)

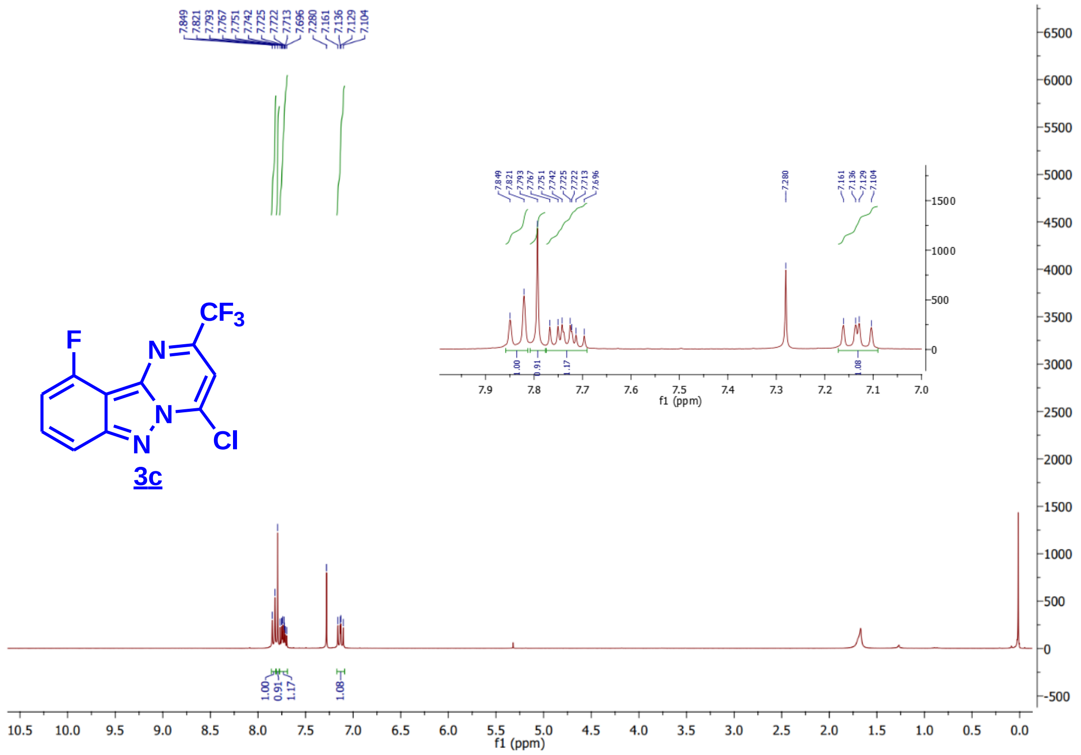

^{19}F NMR (282 MHz, $CDCl_3$)

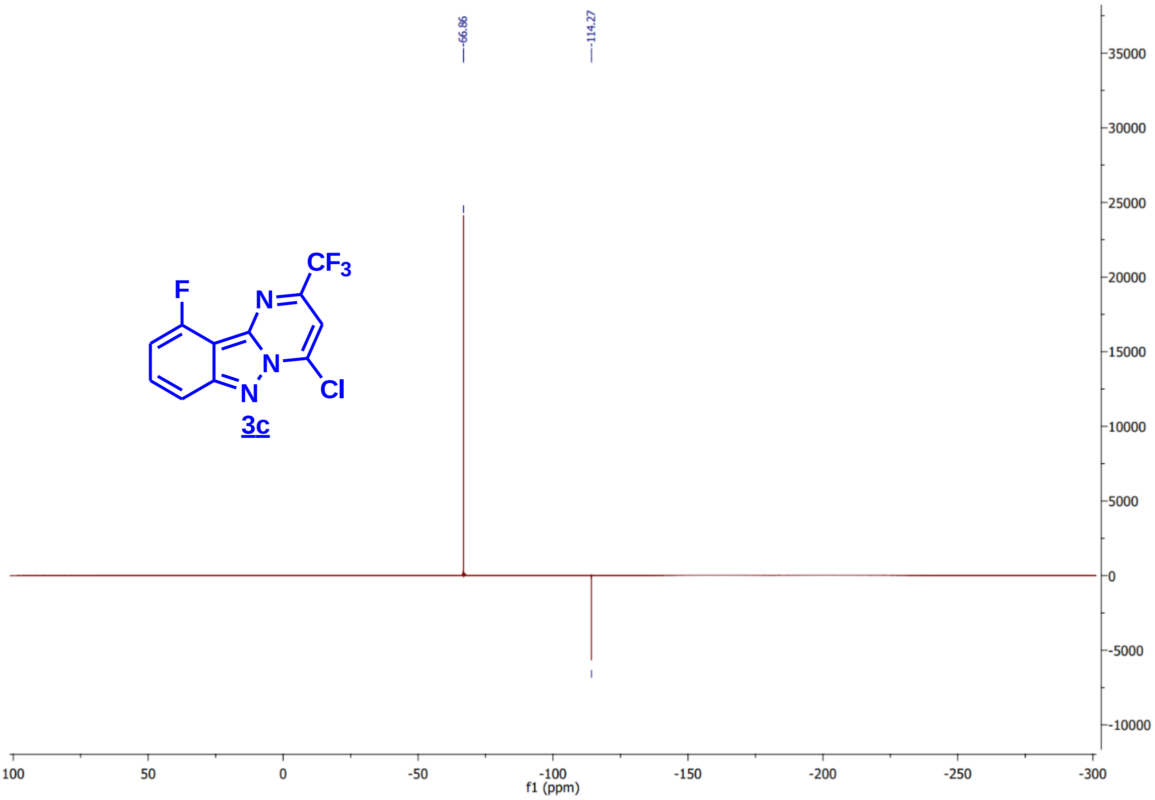

^{13}C NMR (75 MHz, $CDCl_3$)

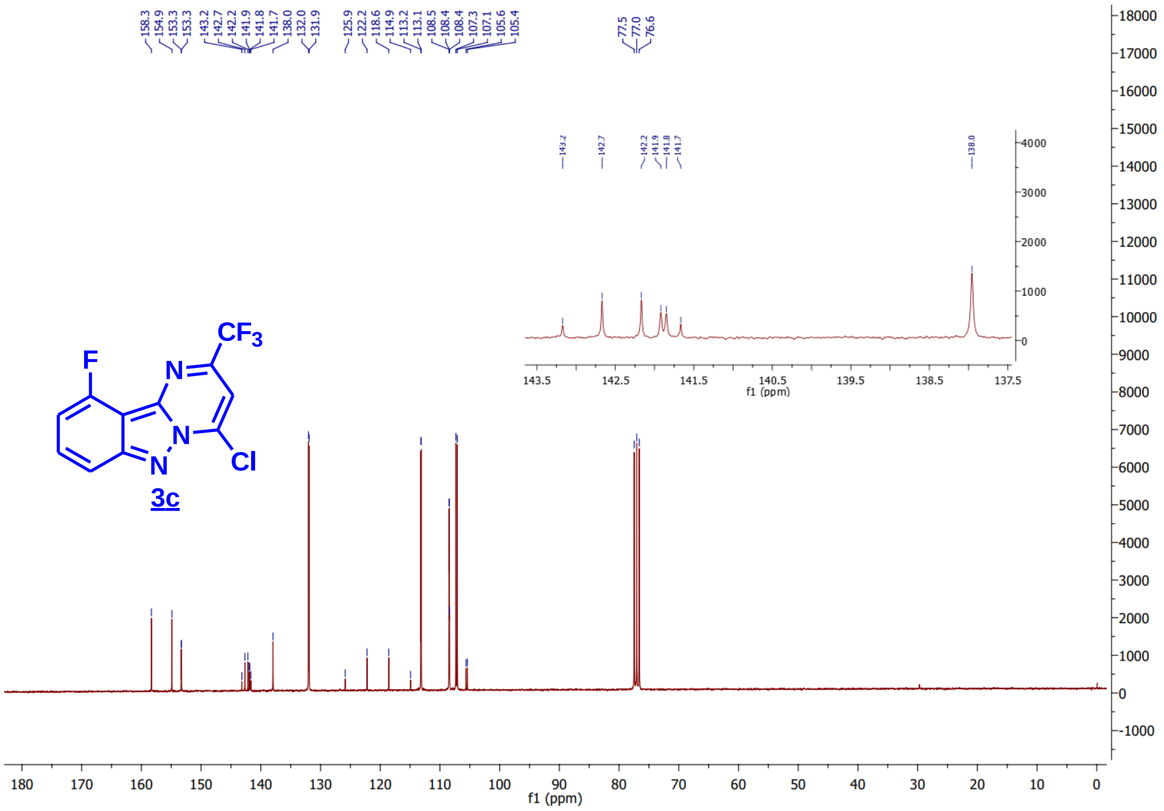

4.4-Chloro-2,8-bis-trifluoromethyl-pyrimido[1,2-b]indazole (3d)

^{1}H NMR (300 MHz, $CDCl_3$)

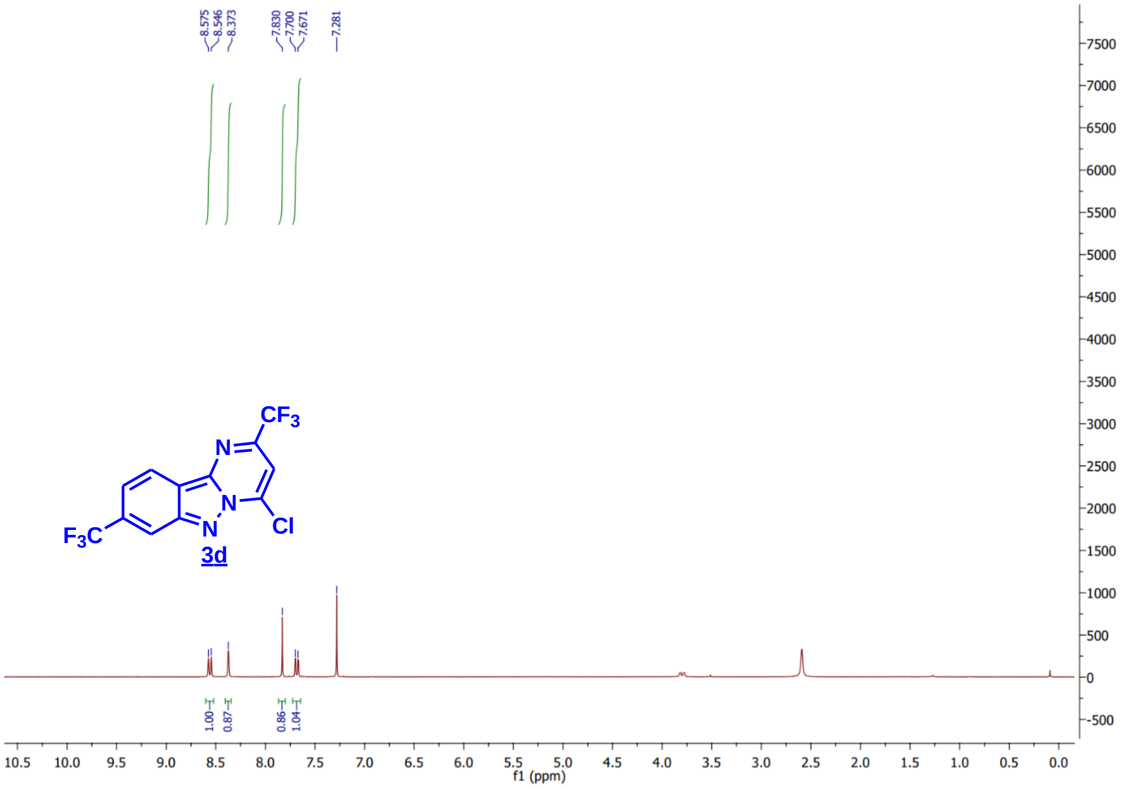

^{19}F NMR (282 MHz, $CDCl_3$)

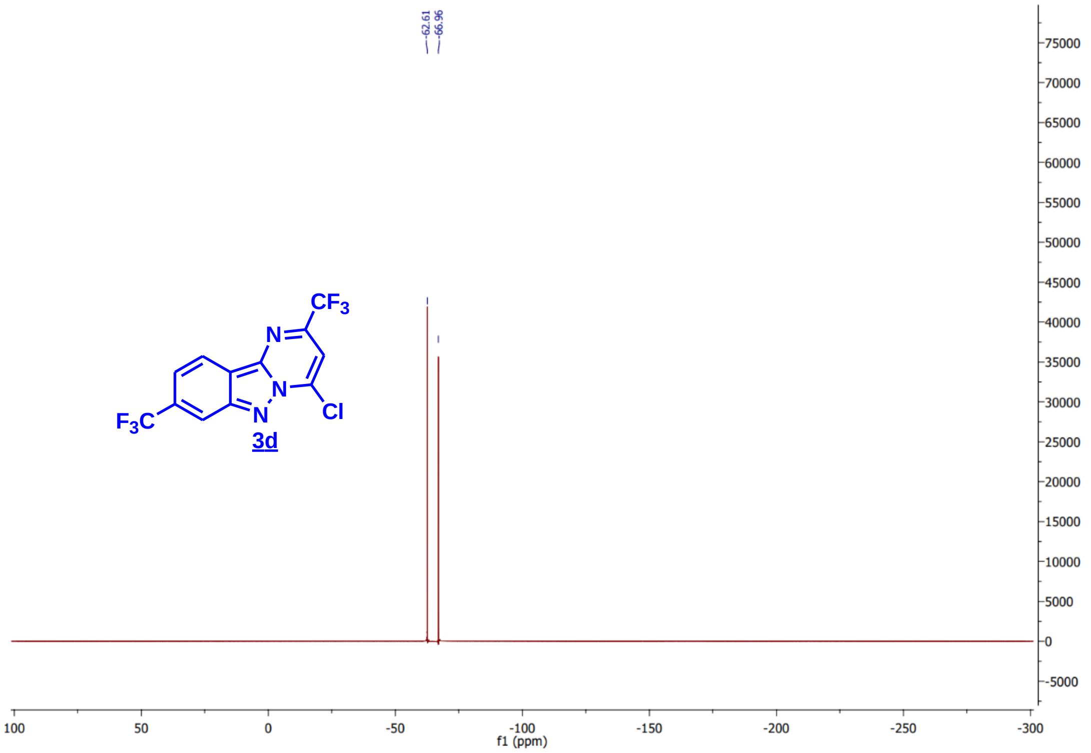

^{13}C NMR (75 MHz, $CDCl_3$)

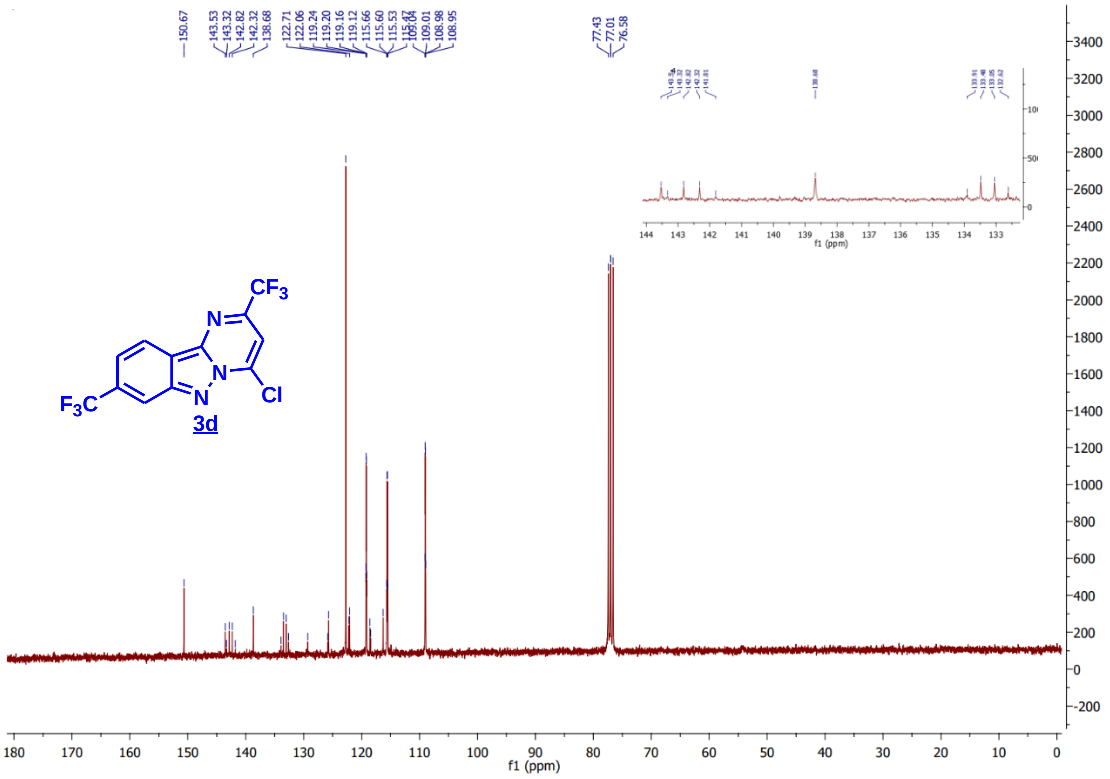

4-Chloro-9-nitro-2-trifluoromethyl-pyrimido[1,2-b]indazole (3e)

^{1}H NMR (300 MHz, $CDCl_3$)

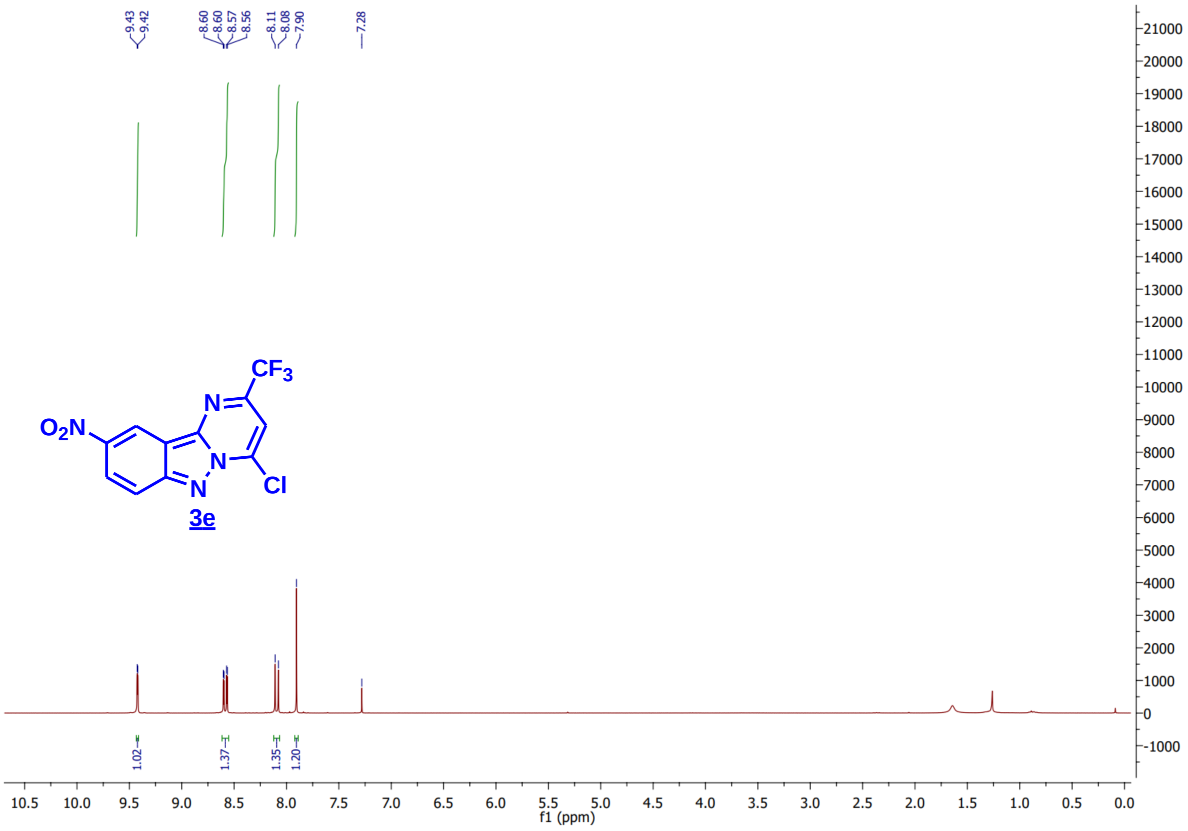

^{19}F NMR (282 MHz, $CDCl_3$)

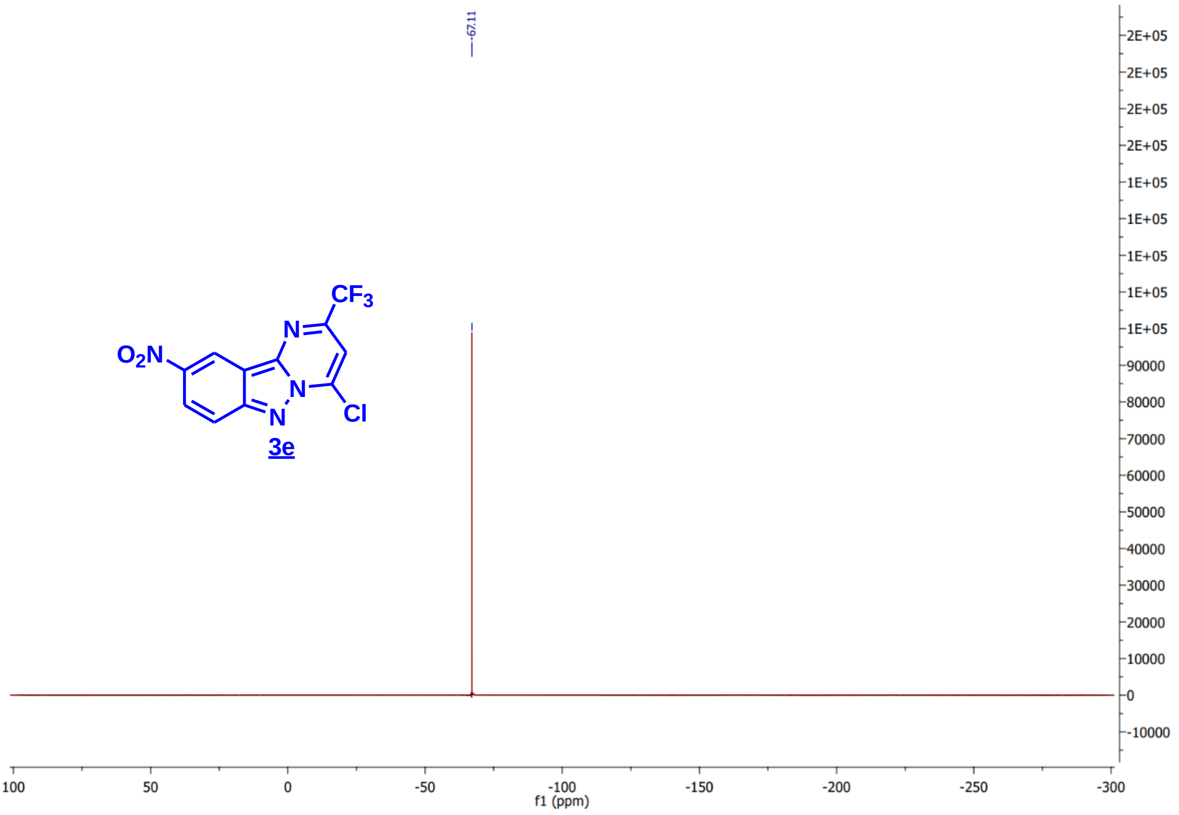

^{13}C NMR (75 MHz, $CDCl_3$)

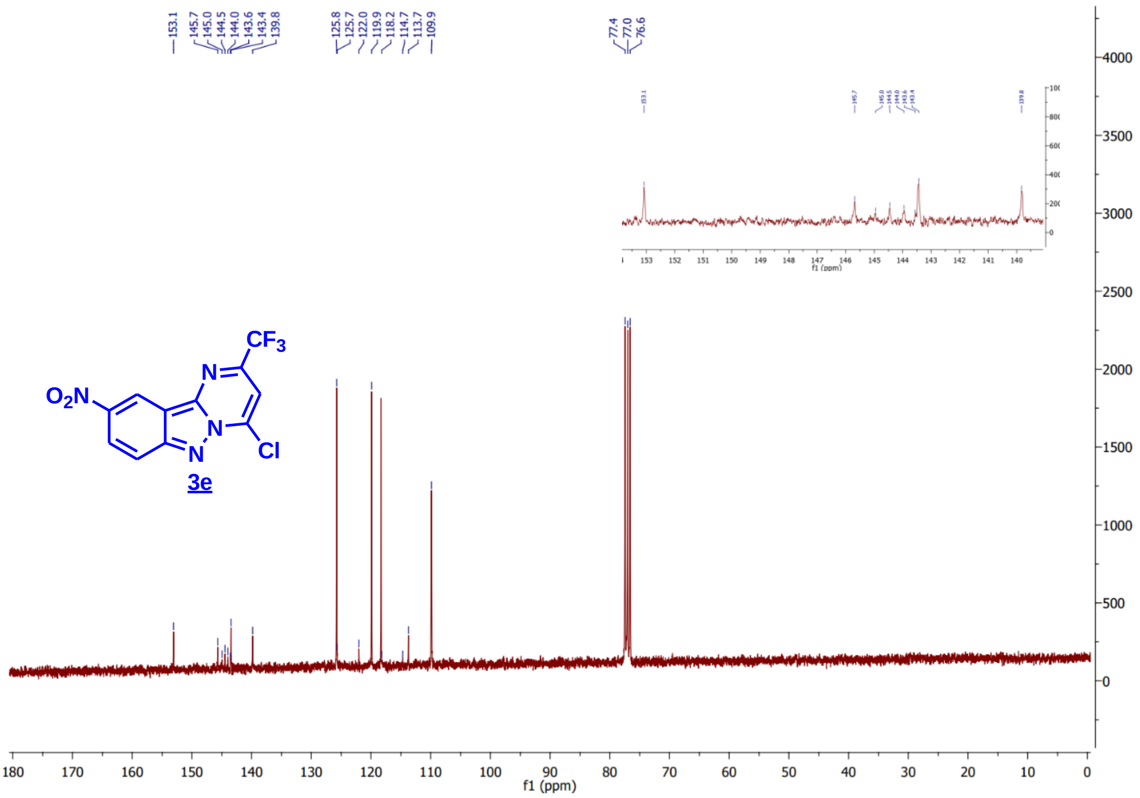

7-chloro-9-(trifluoromethyl)pyrido[3',2':3,4]pyrazolo[1,5-*a*]pyrimidine (3f)

^{1}H NMR (300 MHz, DMSO-d_6)

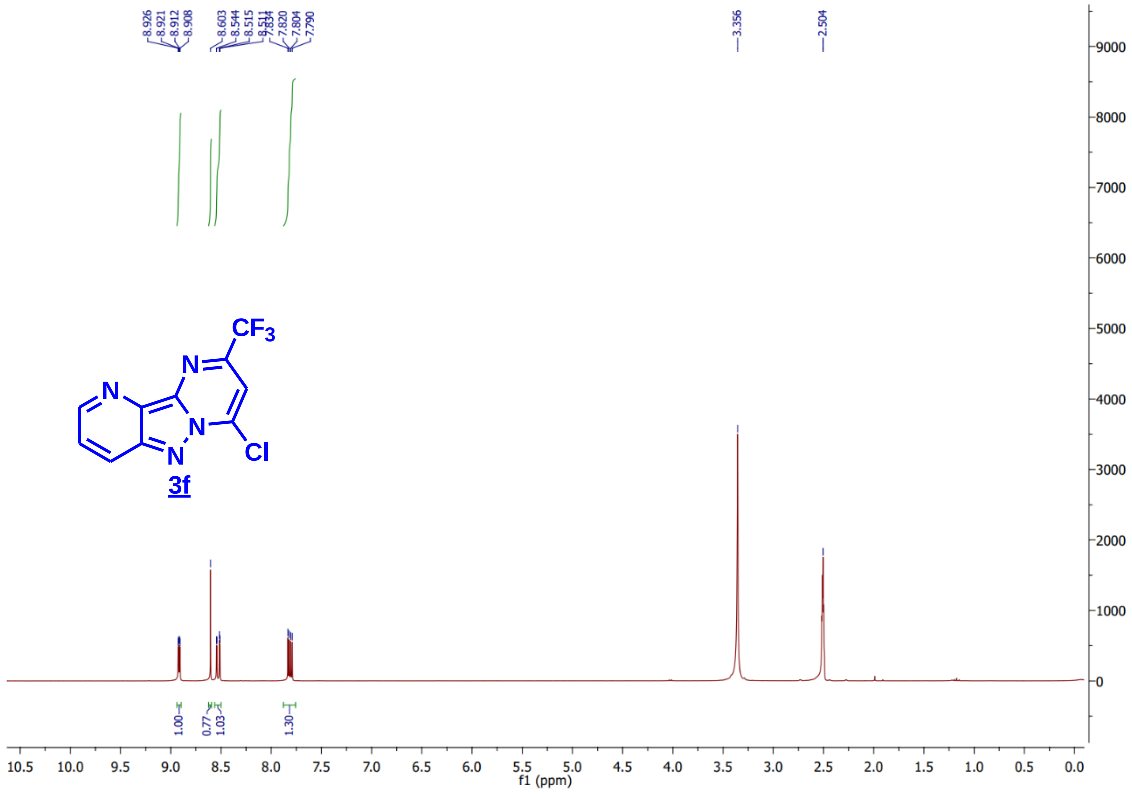

^{19}F NMR (282 MHz, DMSO-d$_6$)

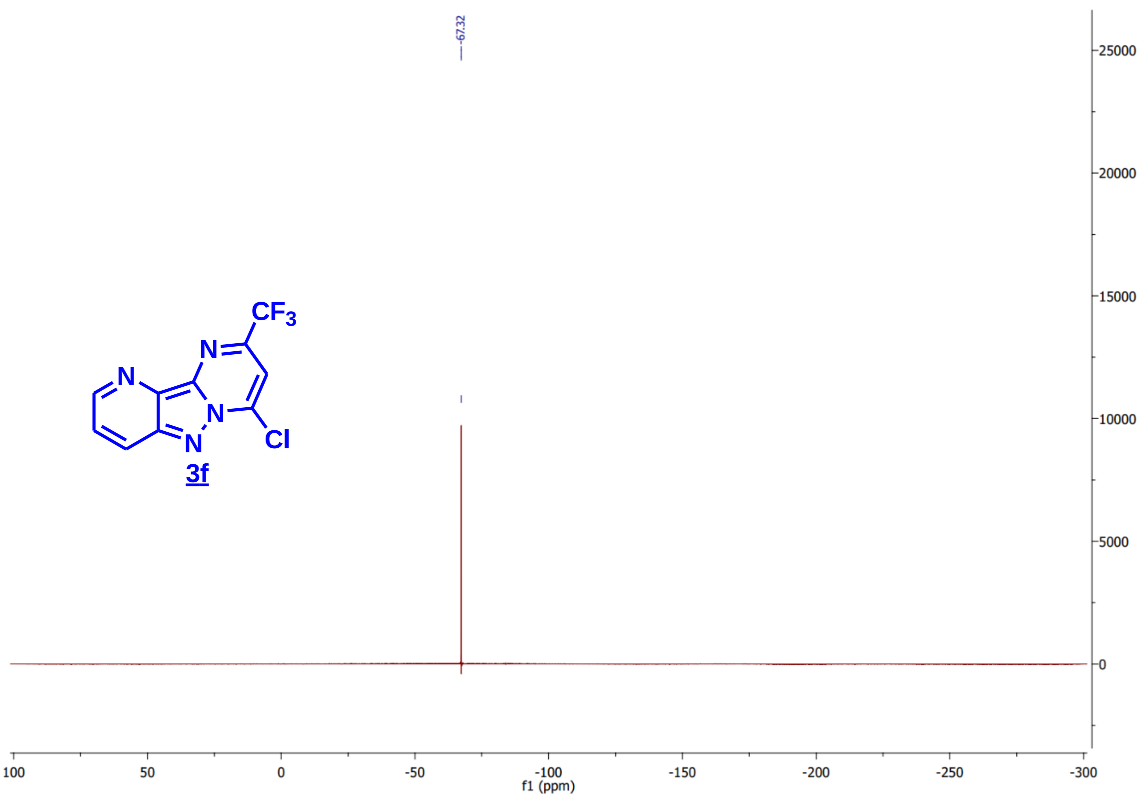

^{13}C NMR (75 MHz, CDCl$_3$)

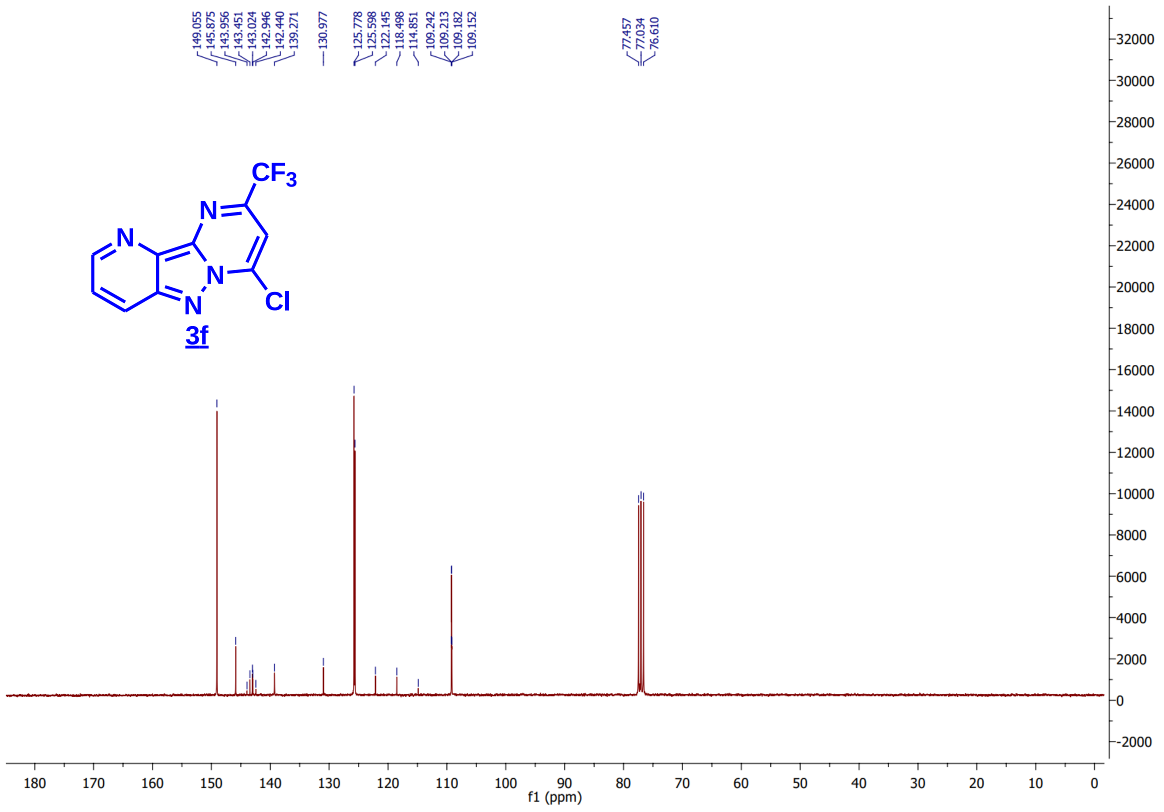

4-chloro-2-(trifluoromethyl)pyrido[2',3':3,4]pyrazolo[1,5-*a*]pyrimidine (3g)

^{1}H NMR (300 MHz, DMSO-d$_6$)

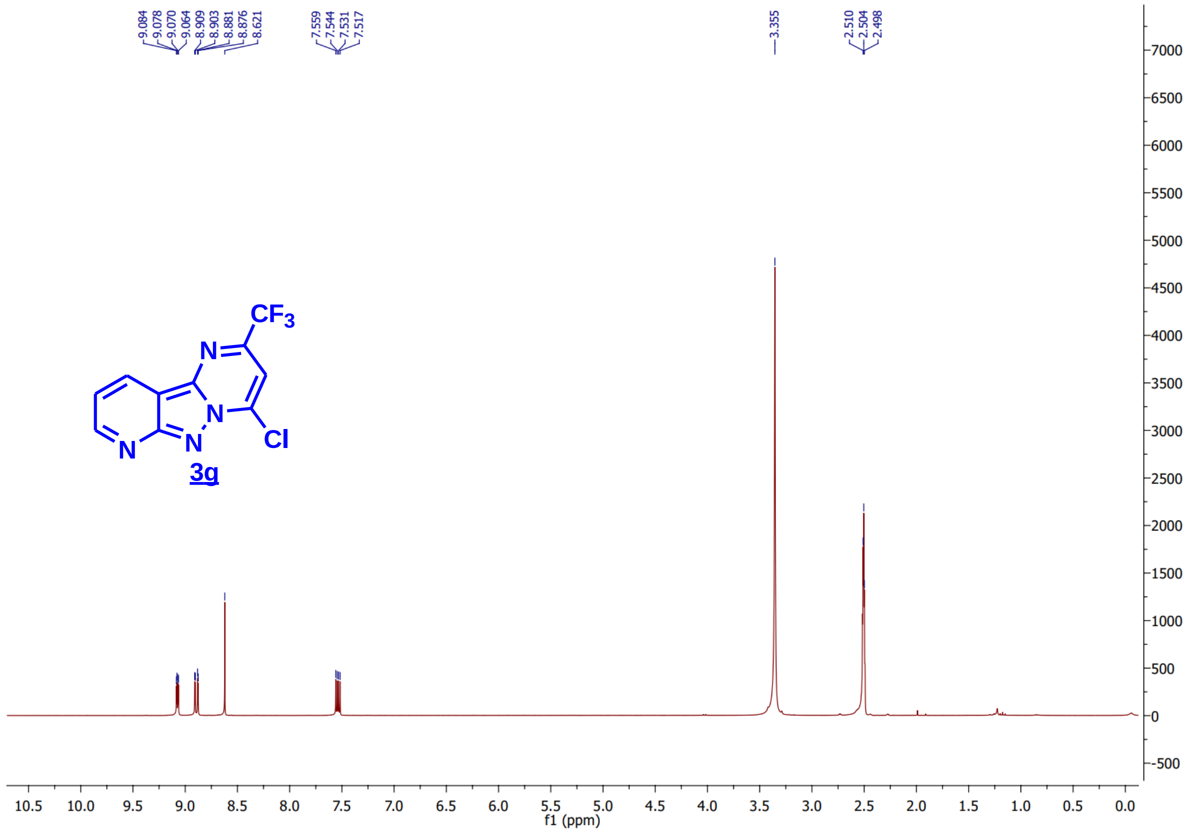

^{19}F NMR (282 MHz, DMSO-d$_6$)

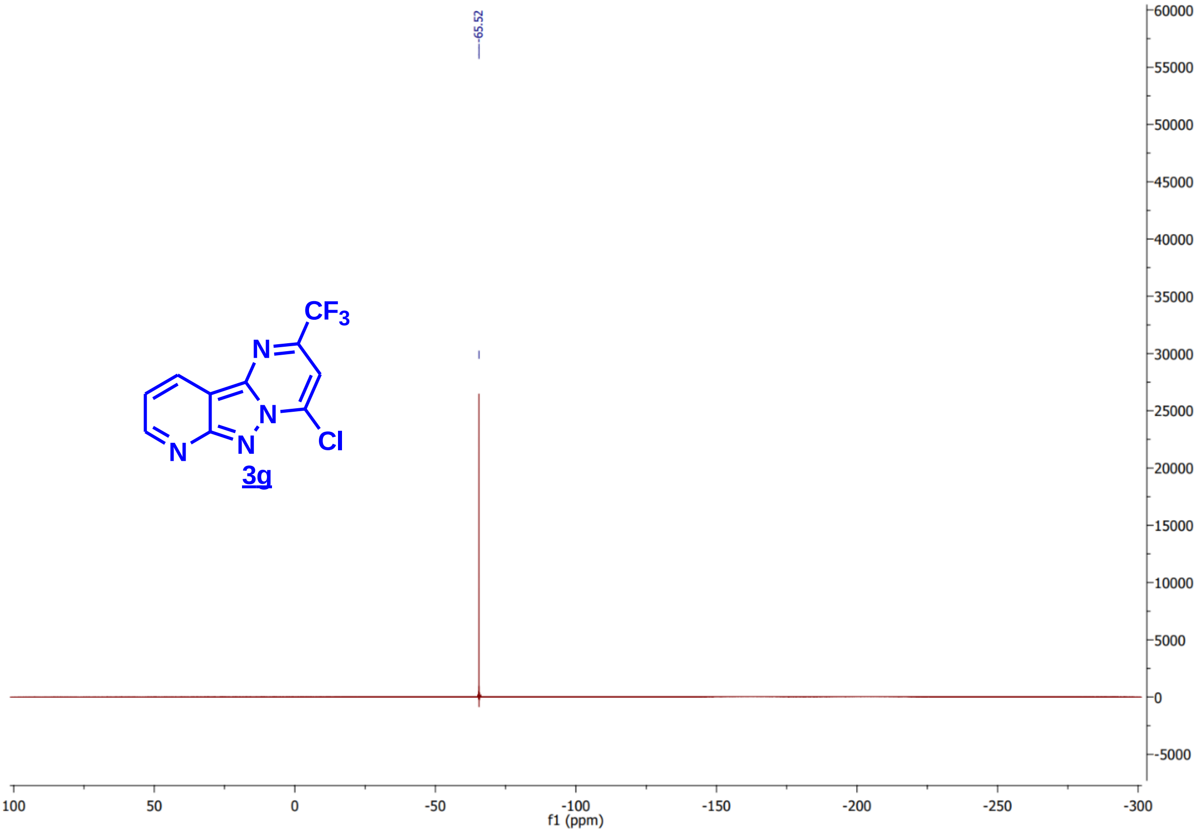

^{13}C NMR (75 MHz, $CDCl_3$)

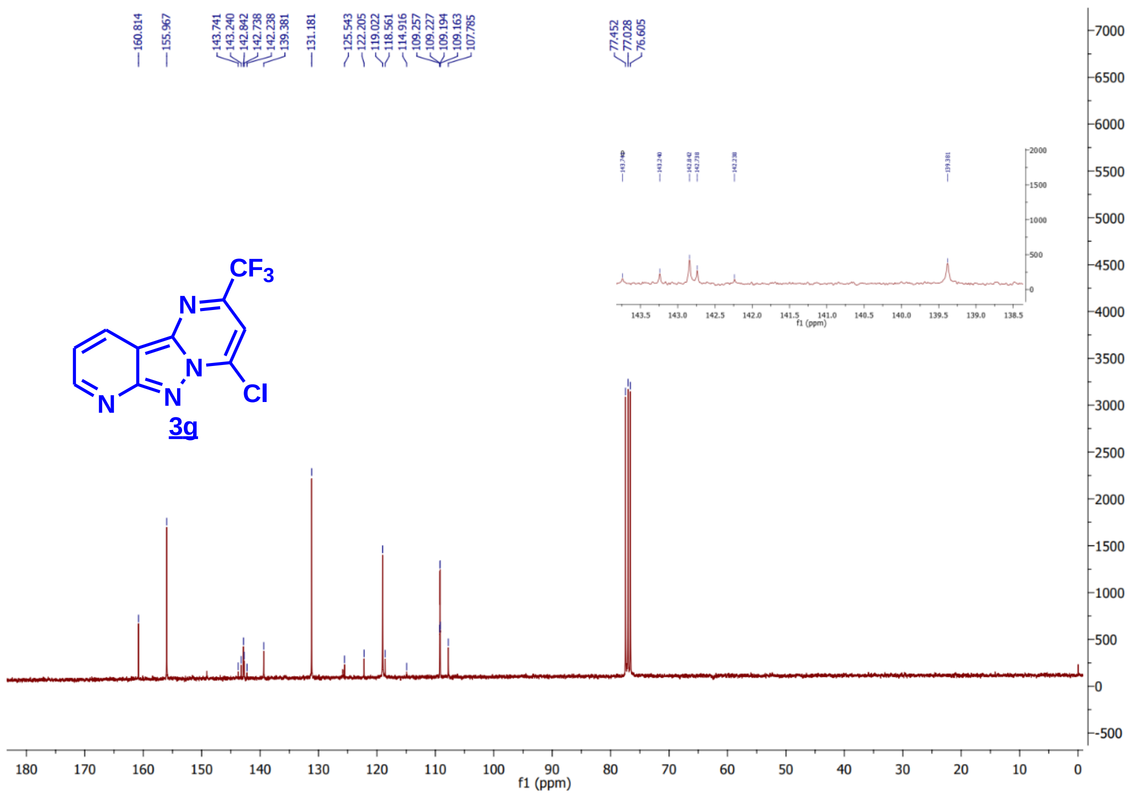

7-chloro-9-(trifluoromethyl)pyrazino[2',3':3,4]pyrazolo[1,5-*a*]pyrimidine (3h)

^{1}H NMR (300 MHz, DMSO-d_6)

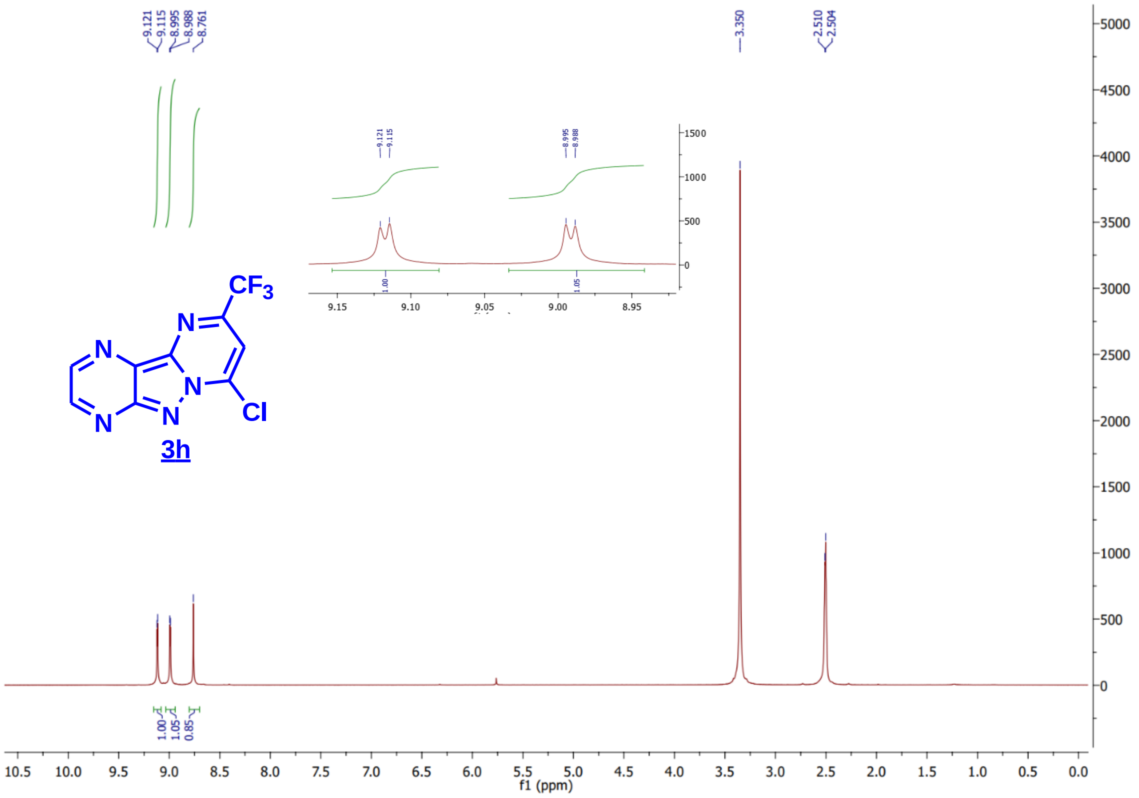

^{19}F NMR (282 MHz, DMSO-d$_6$)

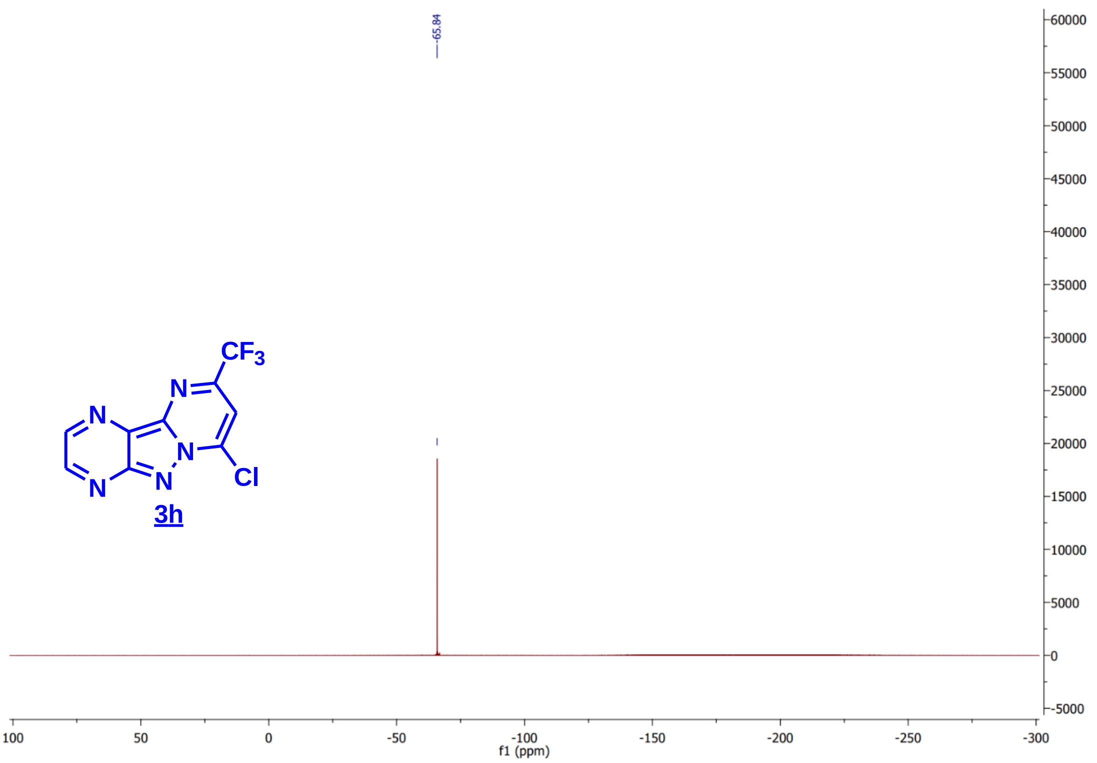

^{13}C NMR (75 MHz, CDCl$_3$)

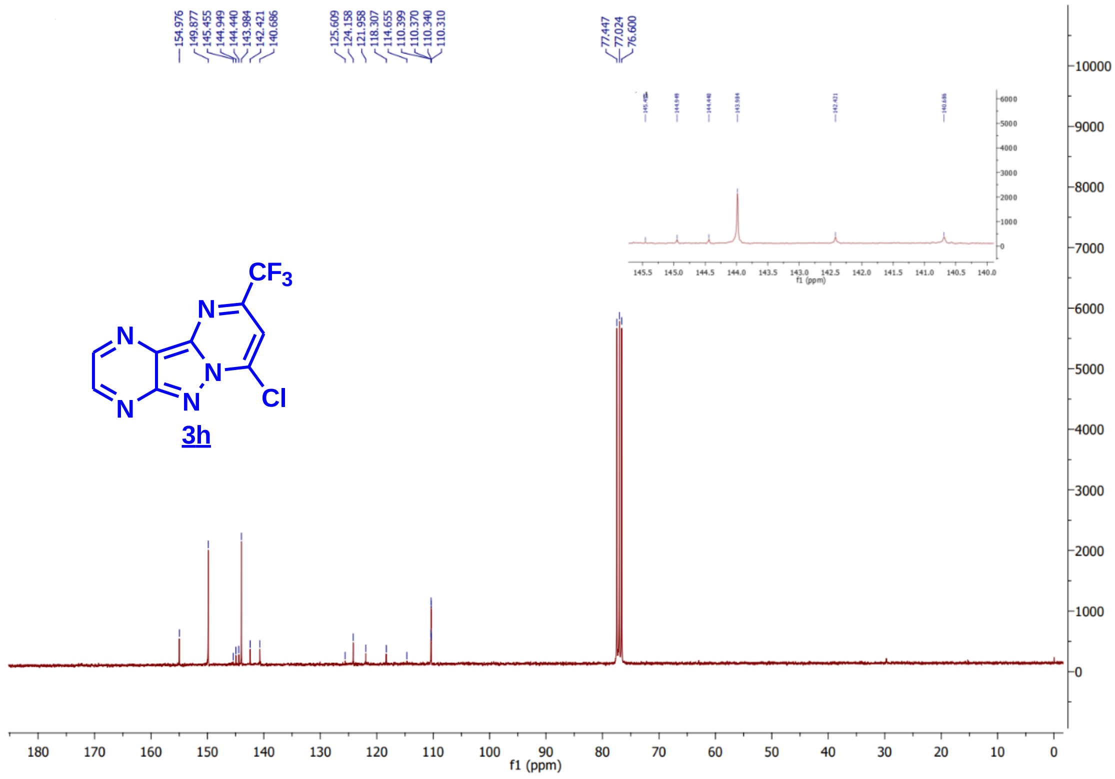

4-(4-Methoxy-phenyl)-2-trifluoromethyl-pyrimido[1,2-b]indazole (4a)

^{1}H NMR (300 MHz, $CDCl_3$)

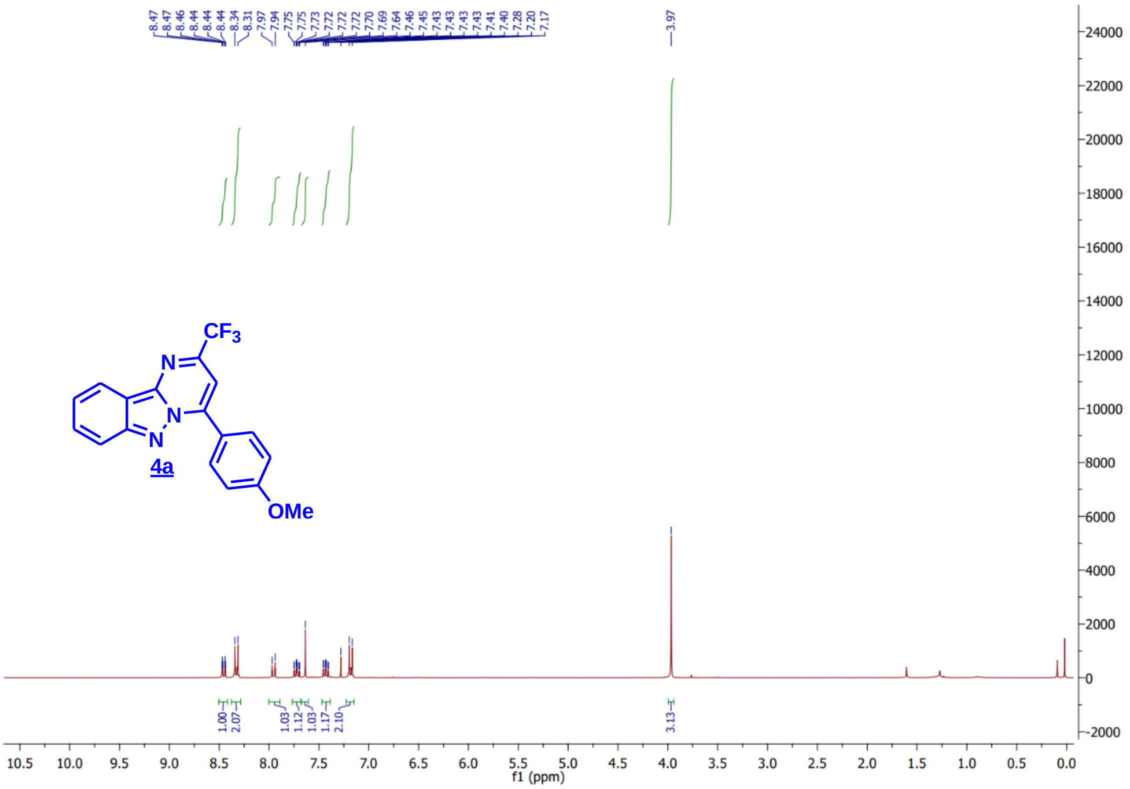

^{19}F NMR (282 MHz, $CDCl_3$)

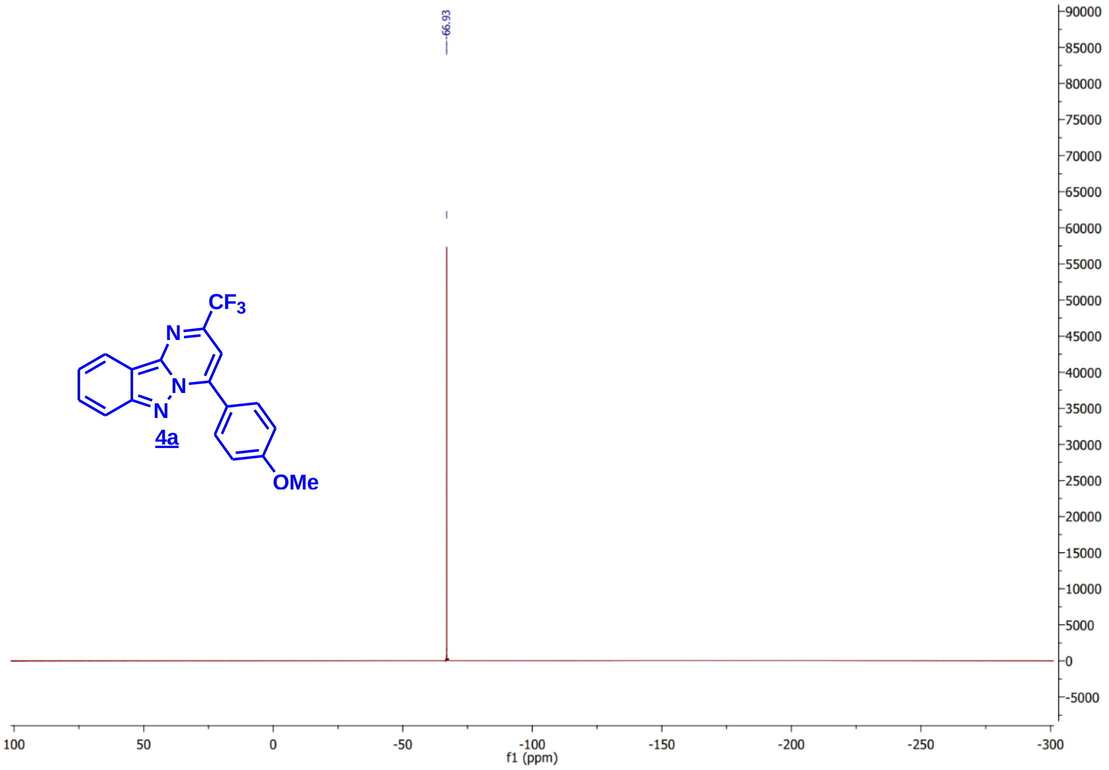

^{13}C NMR (75 MHz, $CDCl_3$)

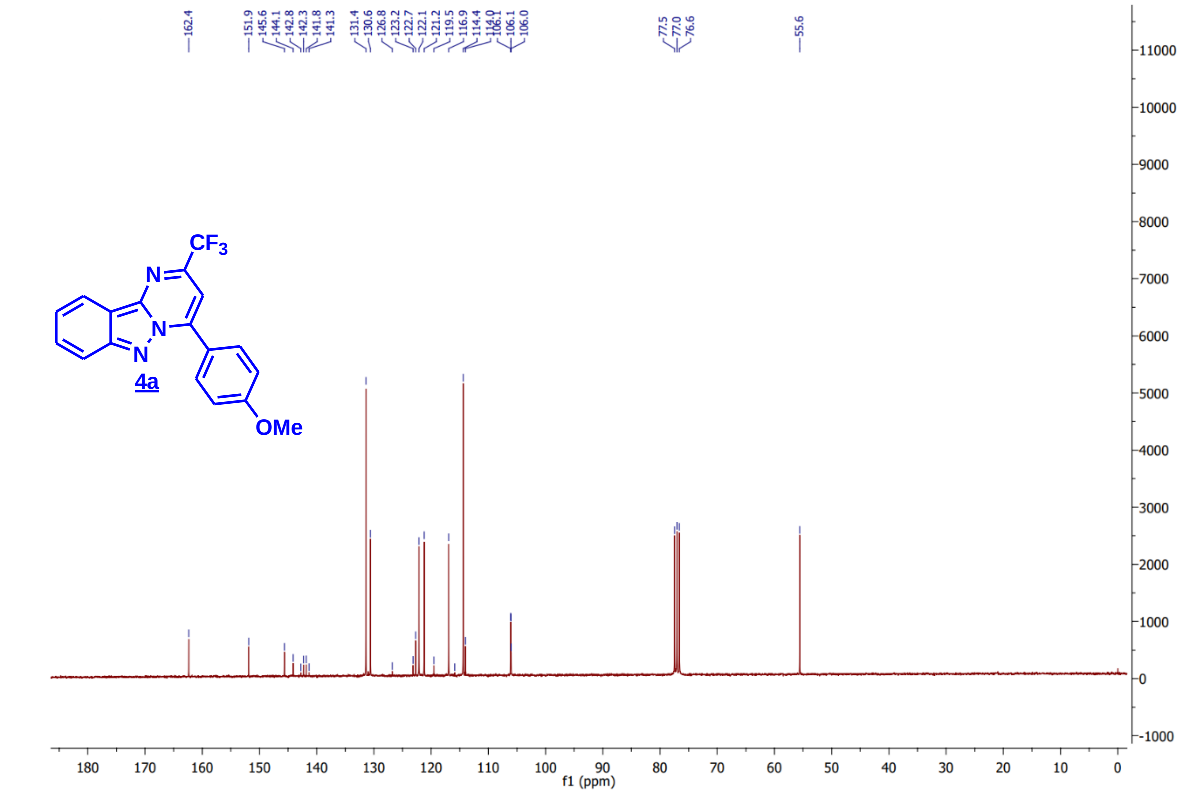

10-Chloro-4-(4-methoxy-phenyl)-2-trifluoromethyl-pyrimido[1,2-b]indazole (4b)

^{1}H NMR (300 MHz, $CDCl_3$)

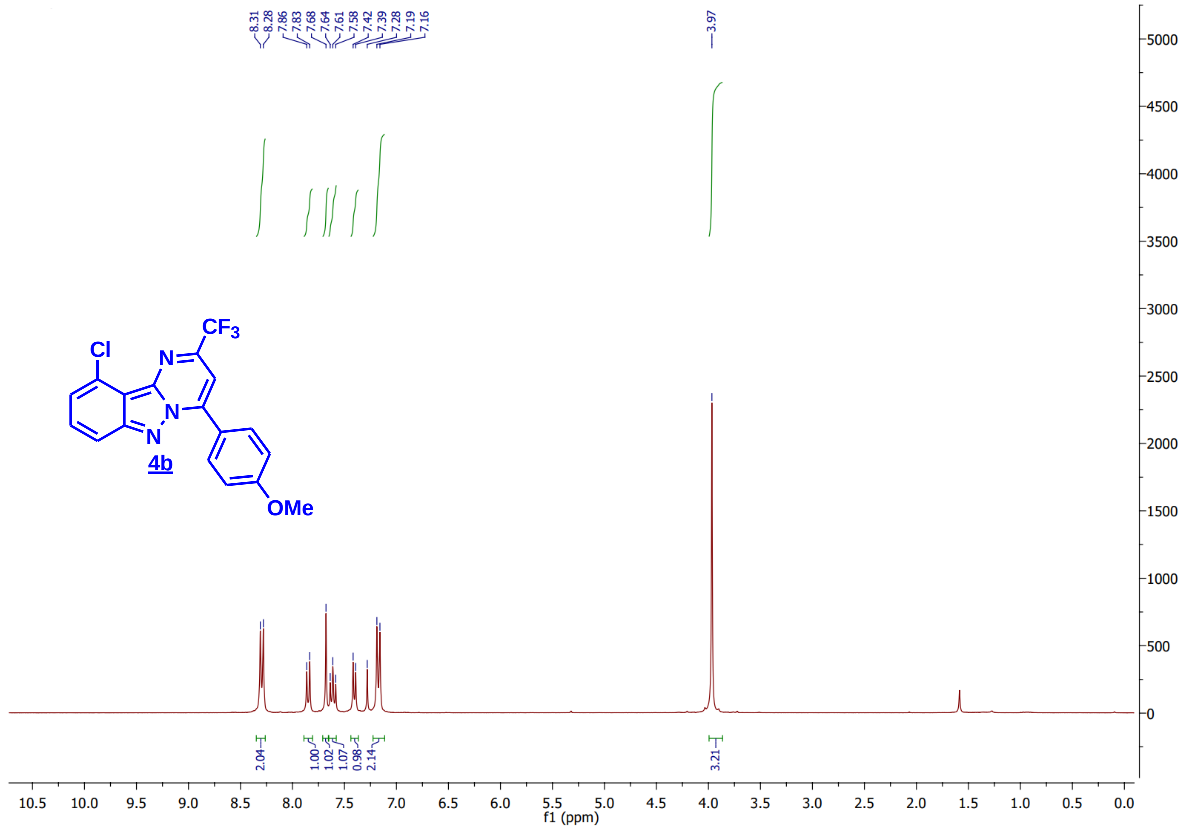

^{19}F NMR (282 MHz,CDCl3)

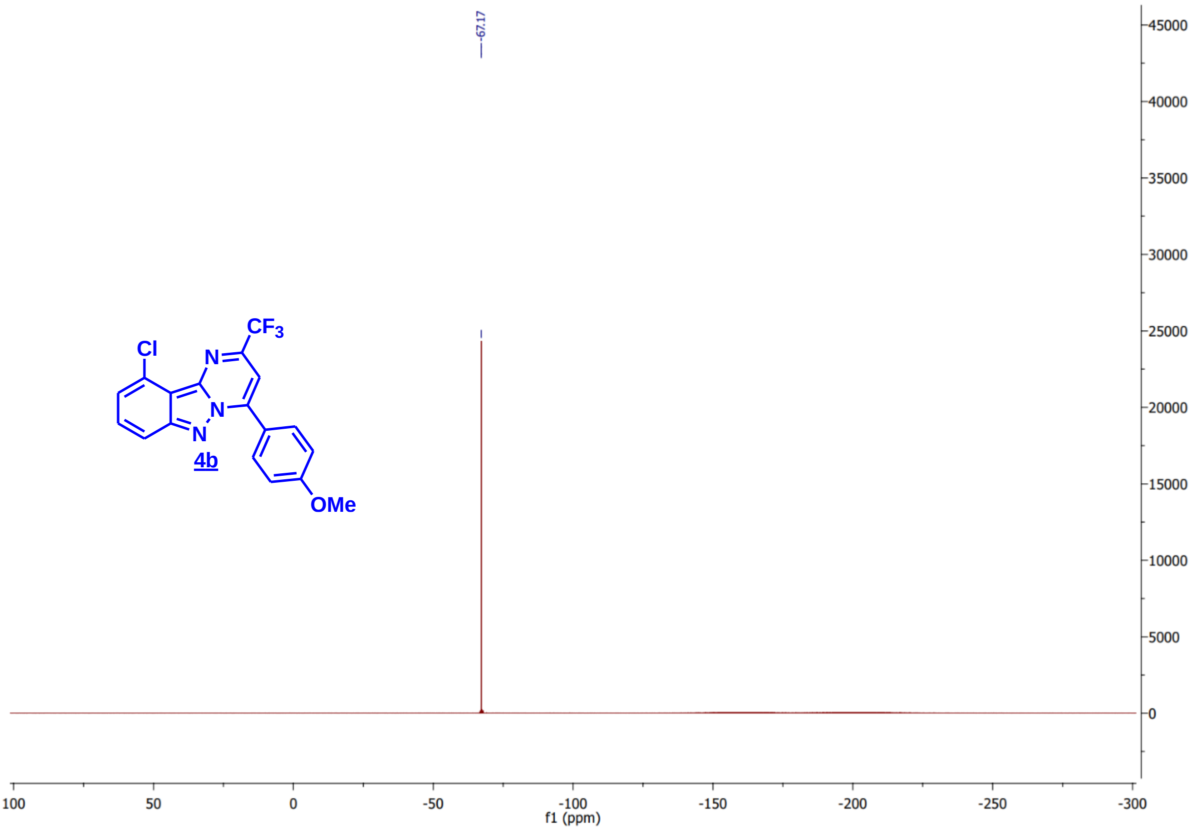

^{13}C NMR (75 MHz, CDCl3)

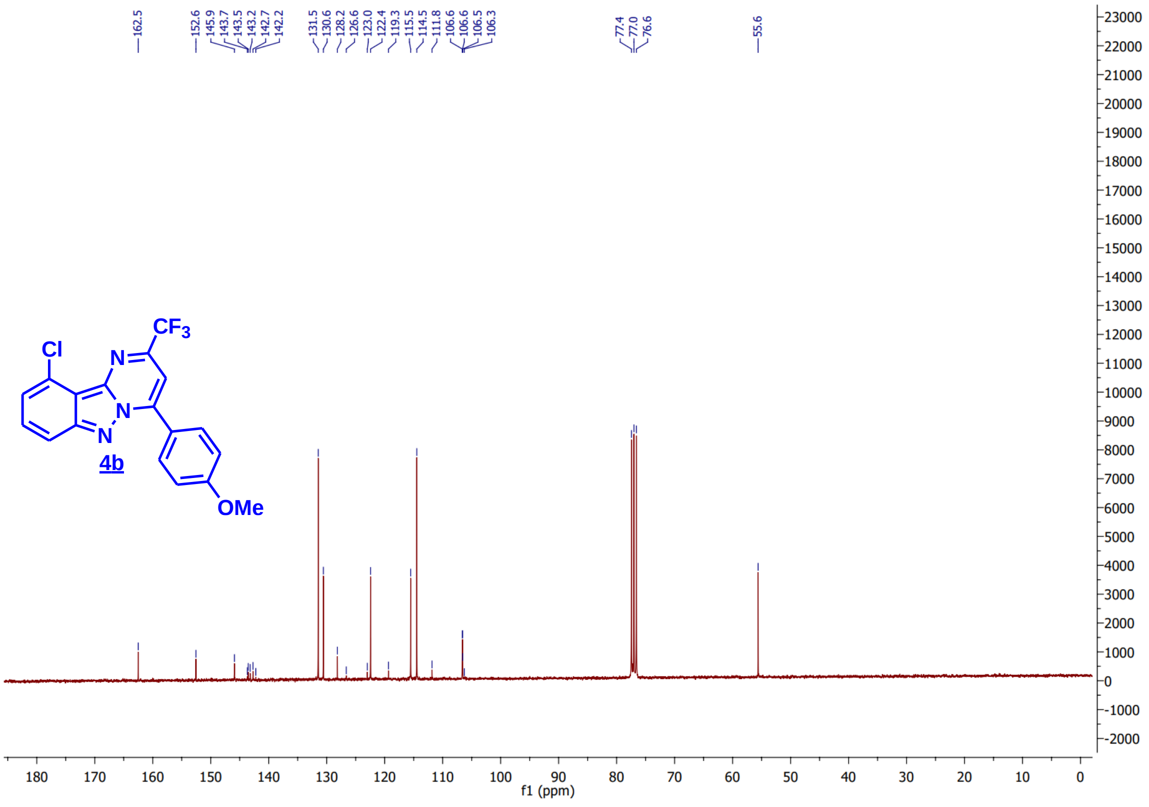

10-Fluoro-4-(4-methoxy-phenyl)-2-trifluoromethyl-pyrimido[1,2-b]indazole (4c)

^{1}H NMR (300 MHz, $CDCl_3$)

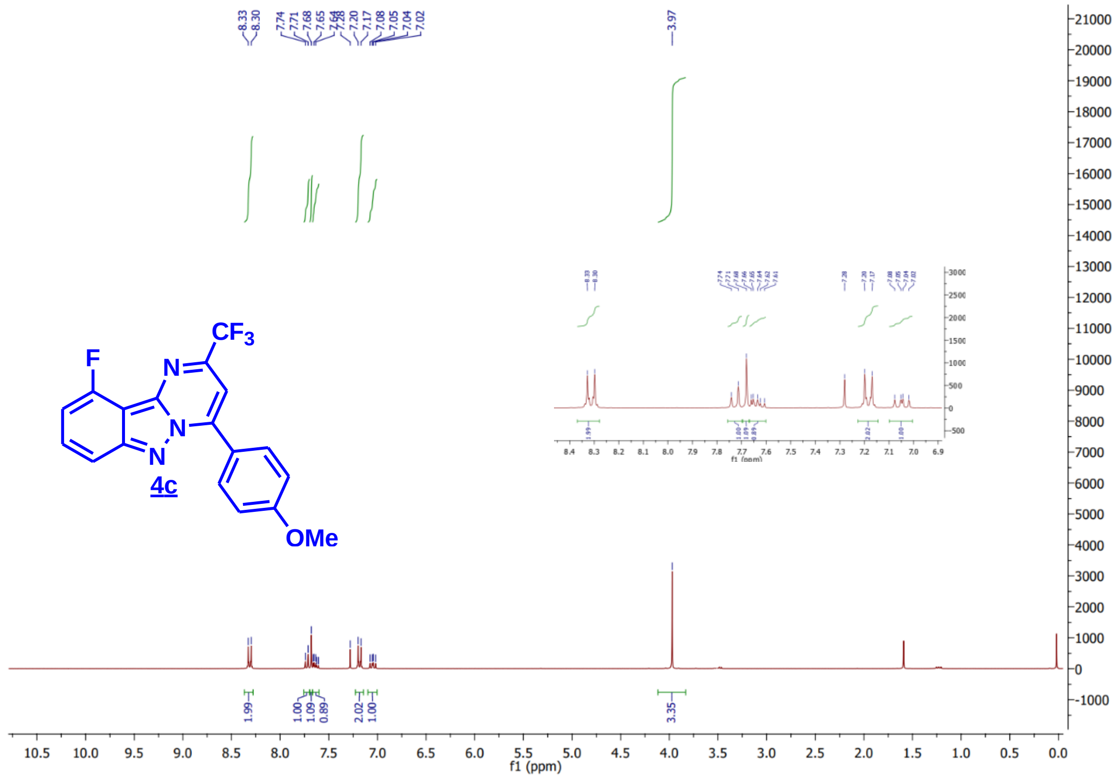

^{19}F NMR (282 MHz,$CDCl_3$)

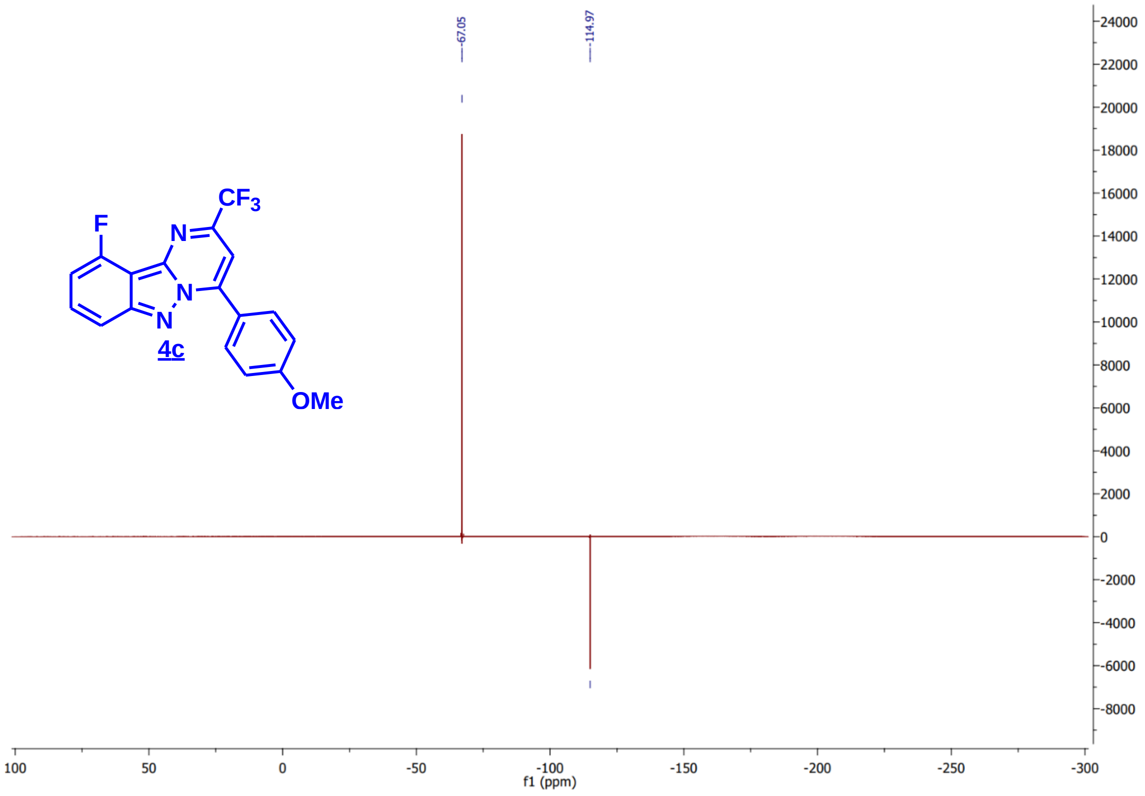

^{13}C NMR (75 MHz, $CDCl_3$)

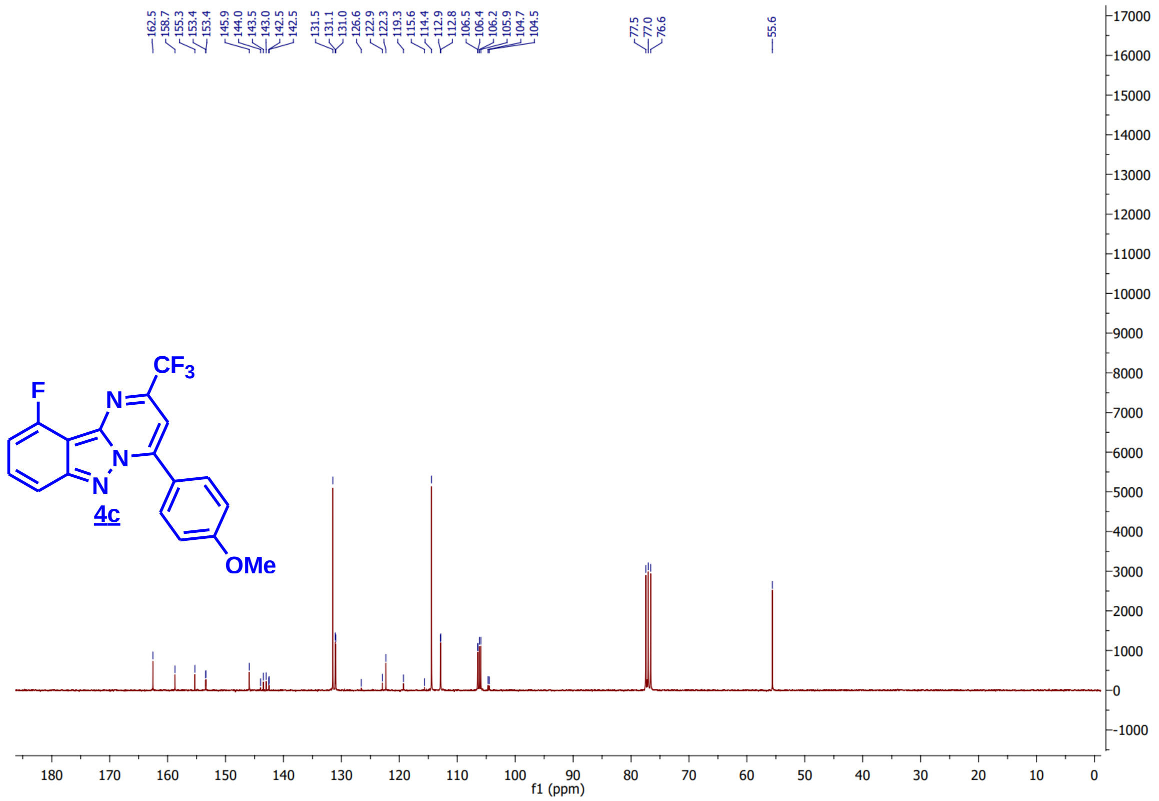

4.4-(4-Methoxy-phenyl)-2,8-bis-trifluoromethyl-pyrimido[1,2-b]indazole (4d)

^{1}H NMR (300 MHz, $CDCl_3$)

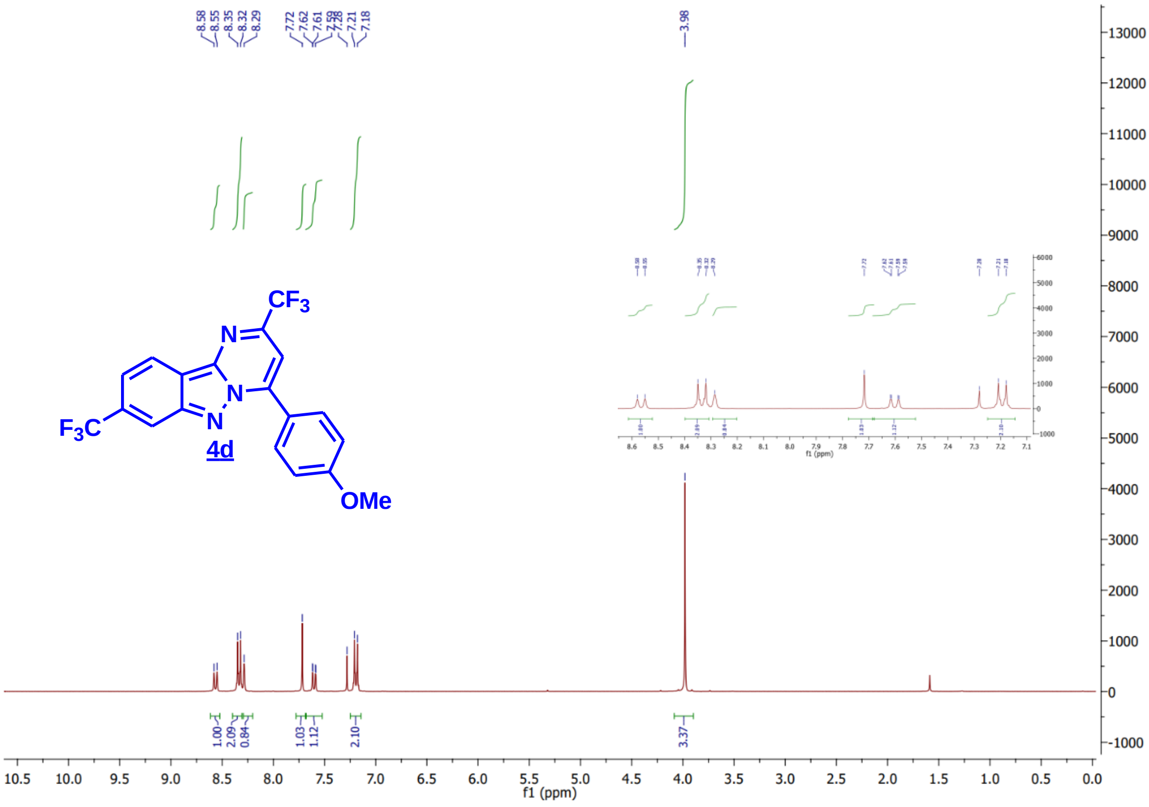

^{19}F NMR (282 MHz,CDCl$_3$)

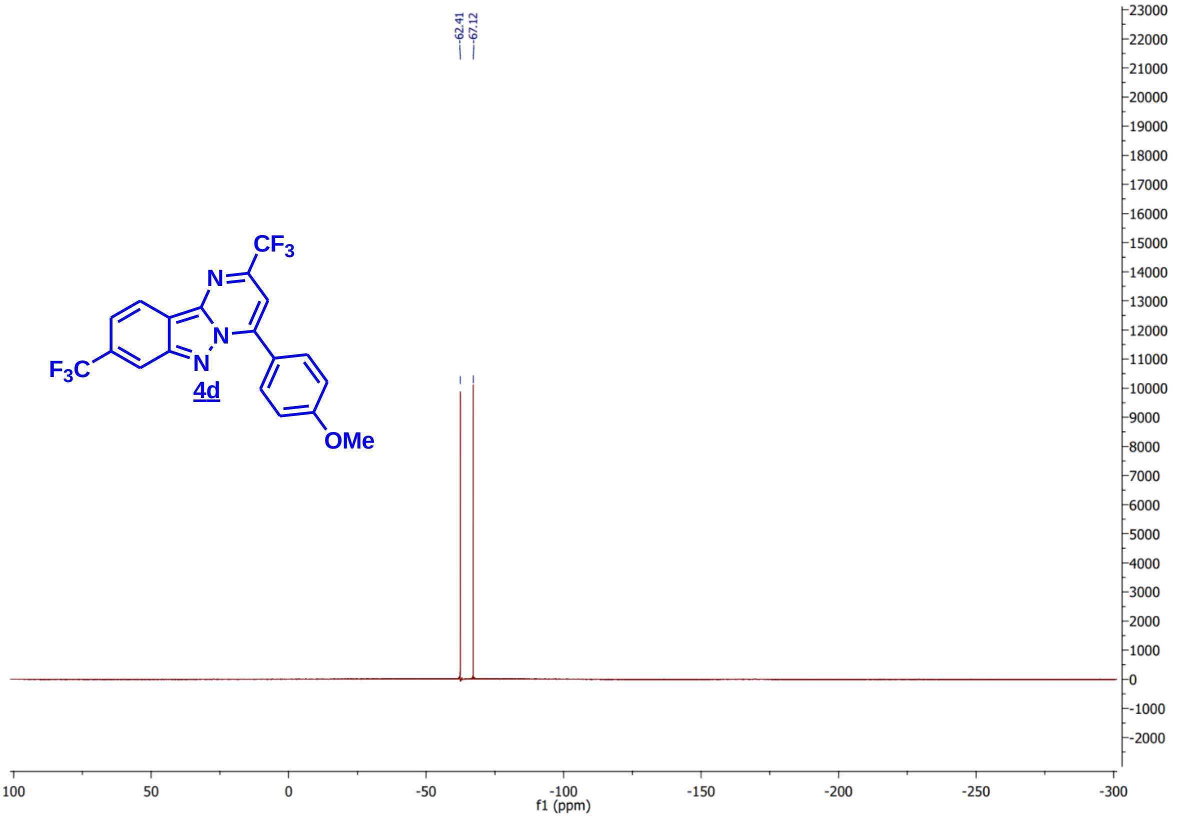

^{13}C NMR (75 MHz, CDCl$_3$)

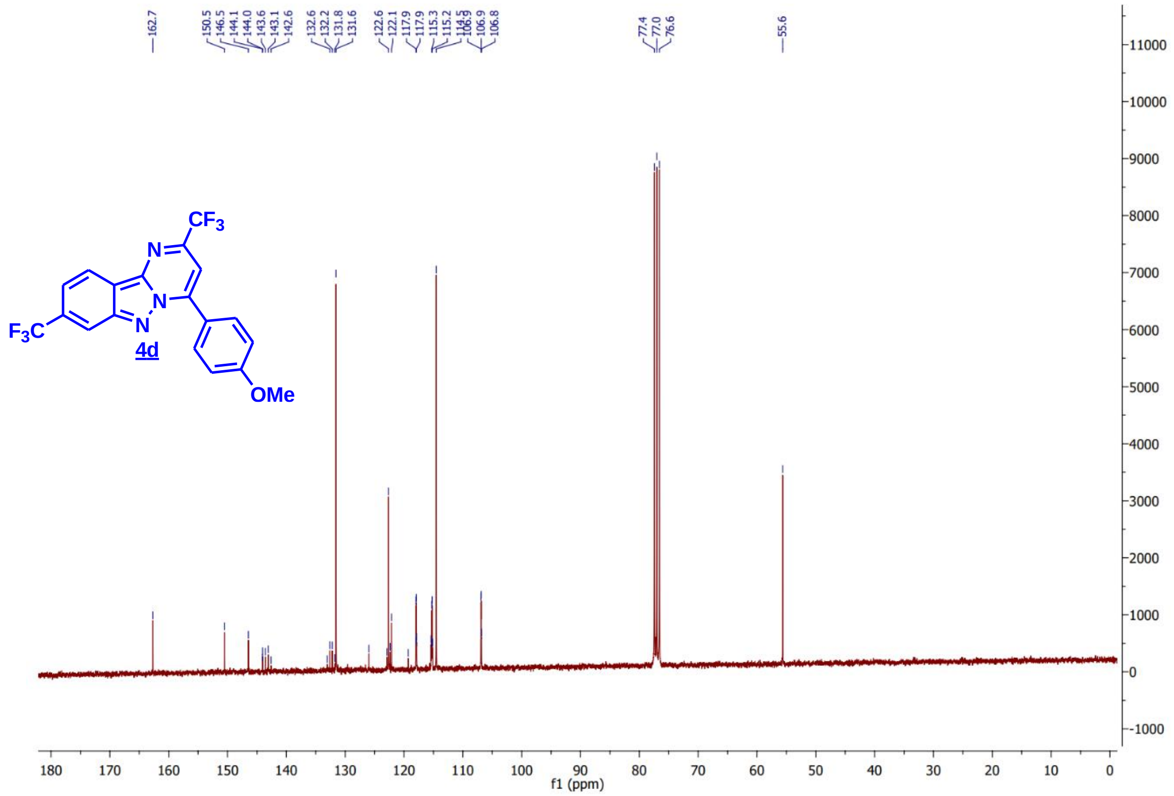

4-(4-Methoxy-phenyl)-9-nitro-2-trifluoromethyl-pyrimido[1,2-b]indazole (4e)

^{1}H NMR (300 MHz, $CDCl_3$)

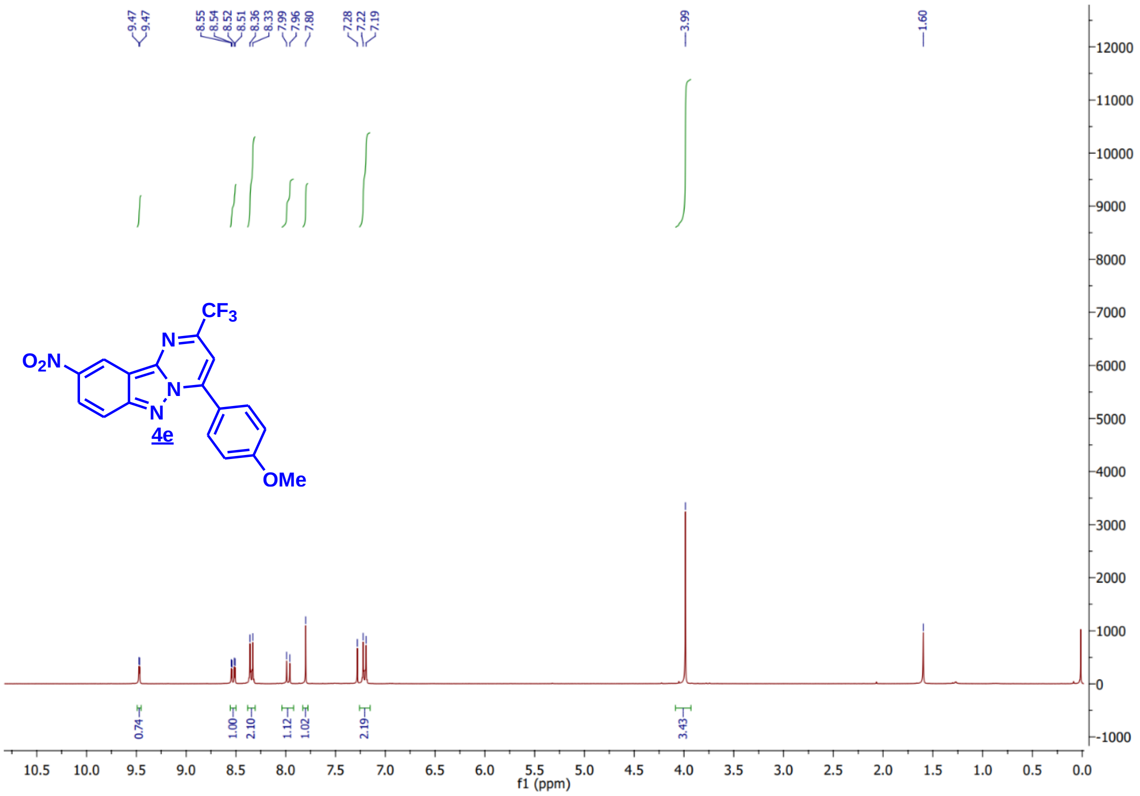

^{19}F NMR (282 MHz,$CDCl_3$)

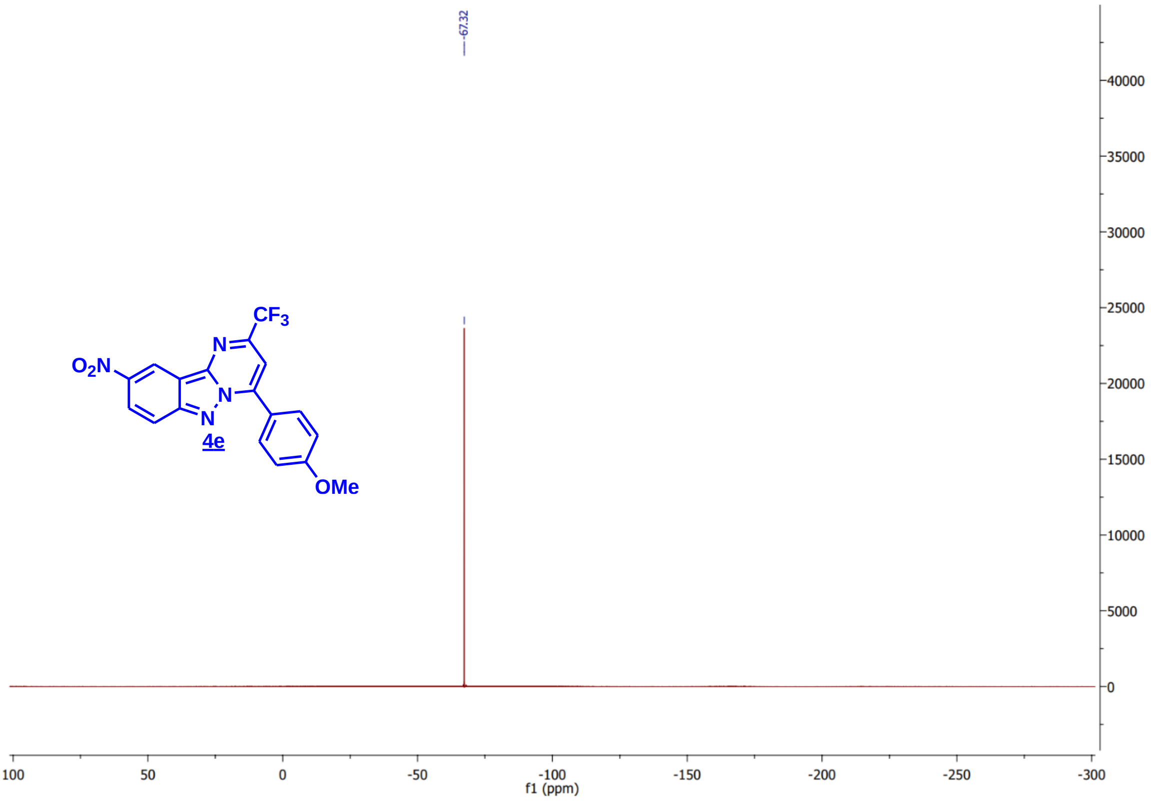

^{13}C NMR (75 MHz, $CDCl_3$)

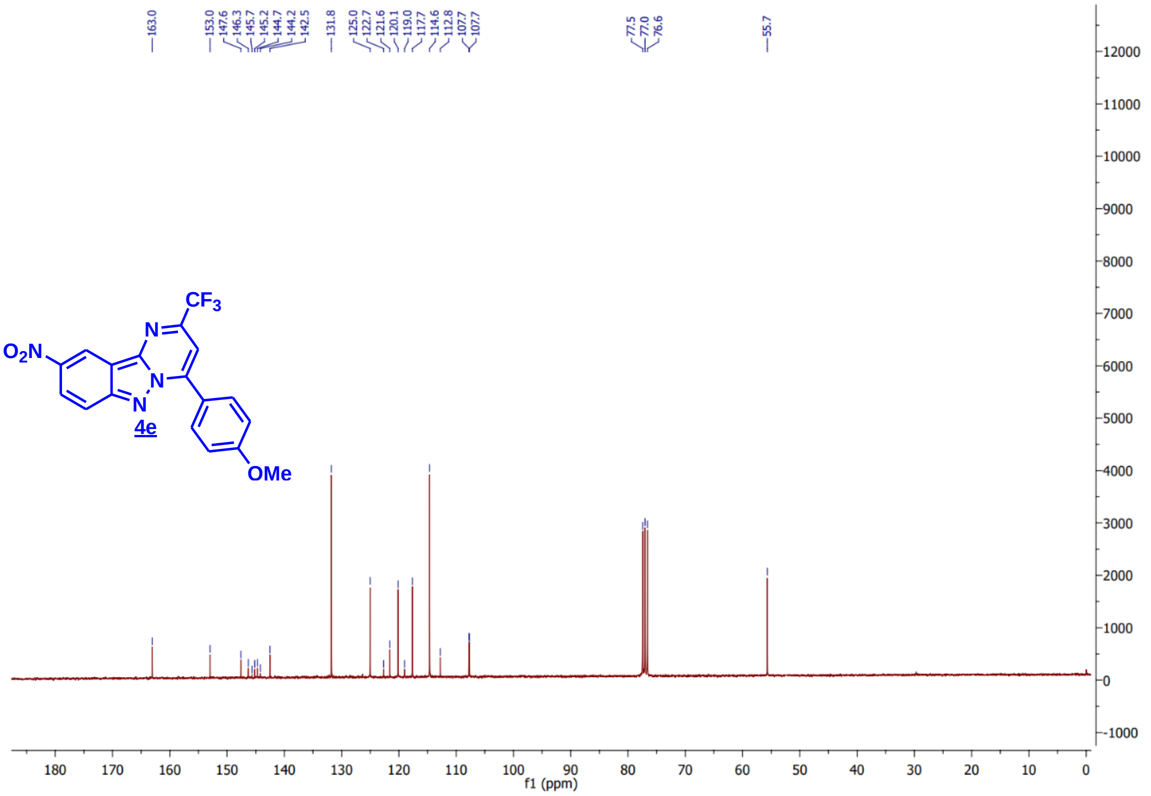

7-(4-methoxyphenyl)-9-(trifluoromethyl)pyrido[3',2':3,4]pyrazolo[1,5-*a*]pyrimidine (4f)

^{1}H NMR (300 MHz, $CDCl_3$)

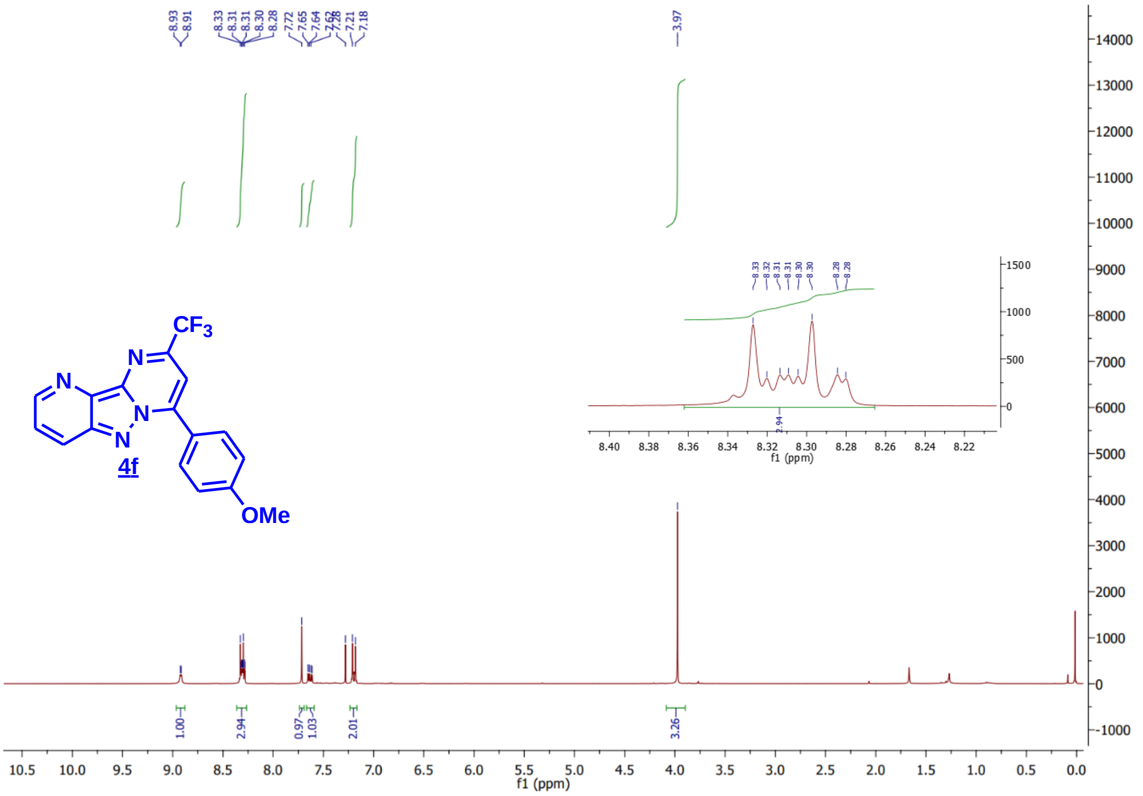

^{19}F NMR (282 MHz,CDCl$_3$)

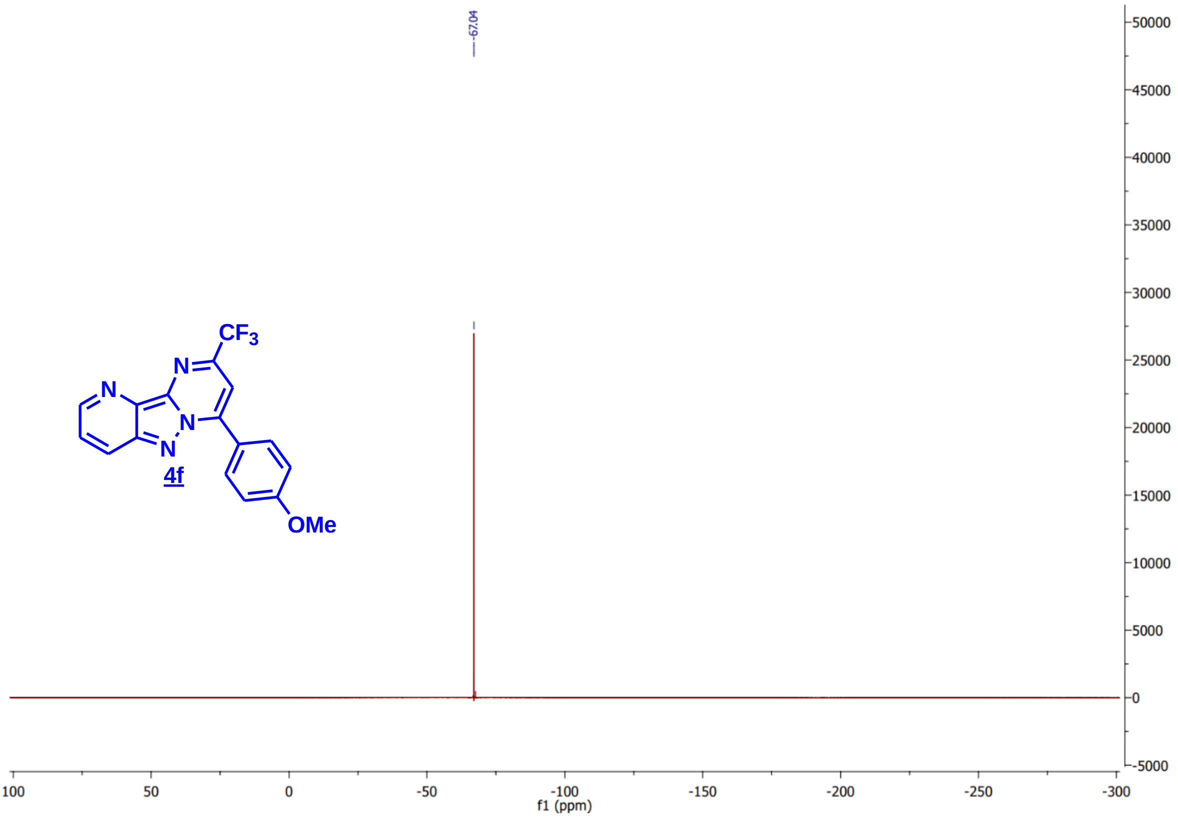

^{13}C NMR (75 MHz, CDCl$_3$)

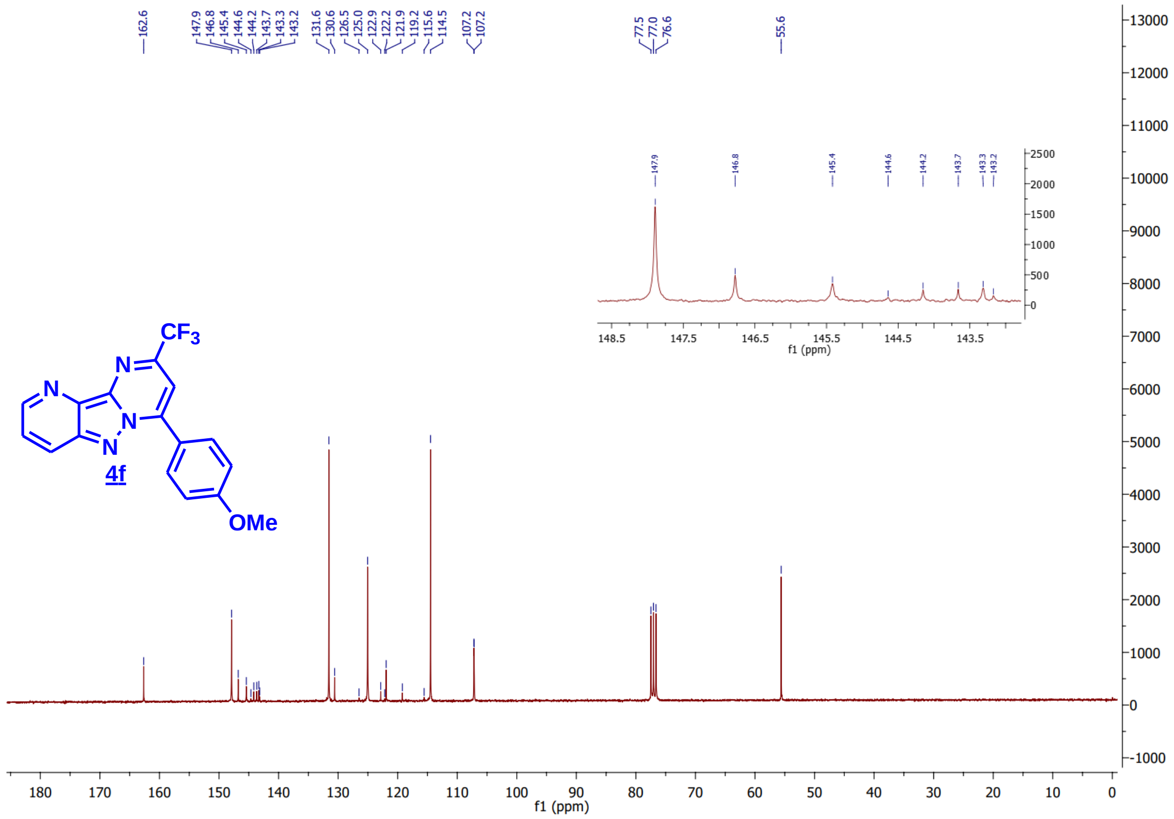

4-(4-methoxyphenyl)-2-(trifluoromethyl)pyrido[2',3':3,4]pyrazolo[1,5-*a*]pyrimidine (4g)

^{1}H NMR (300 MHz, $CDCl_3$)

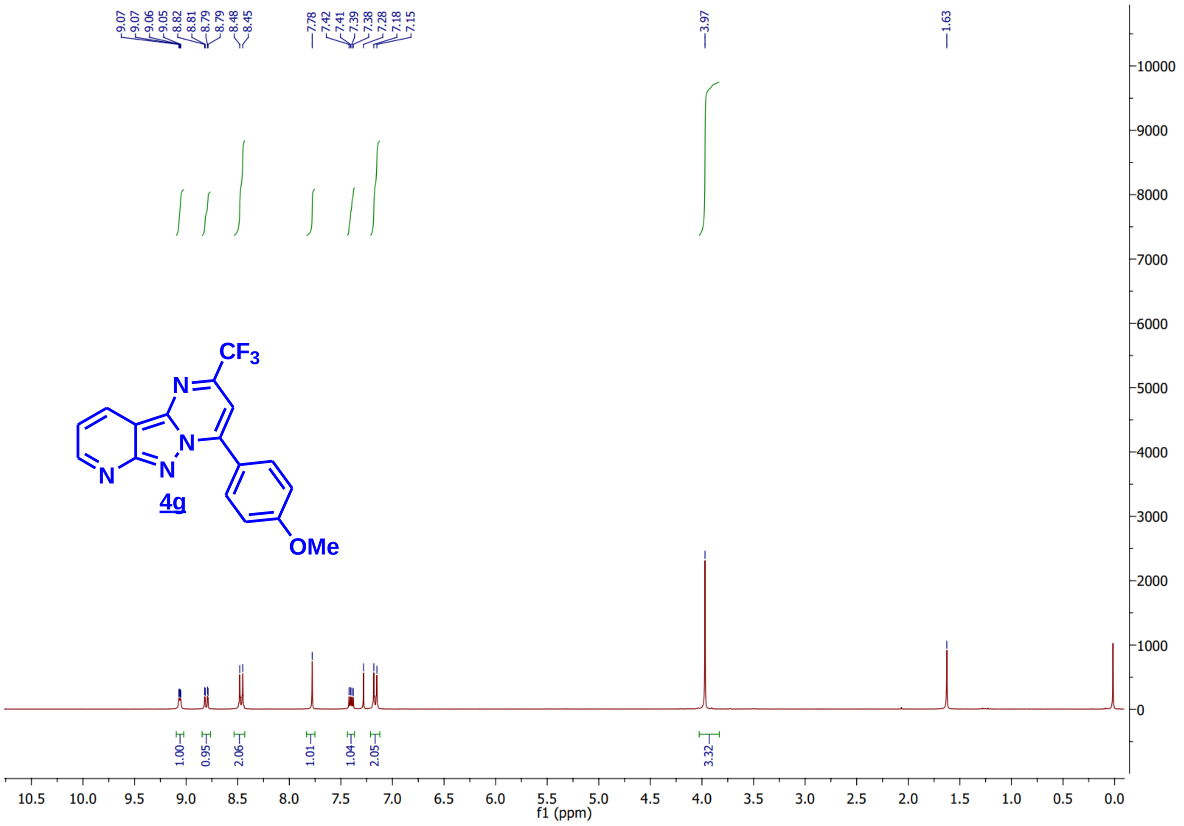

^{19}F NMR (282 MHz, $CDCl_3$)

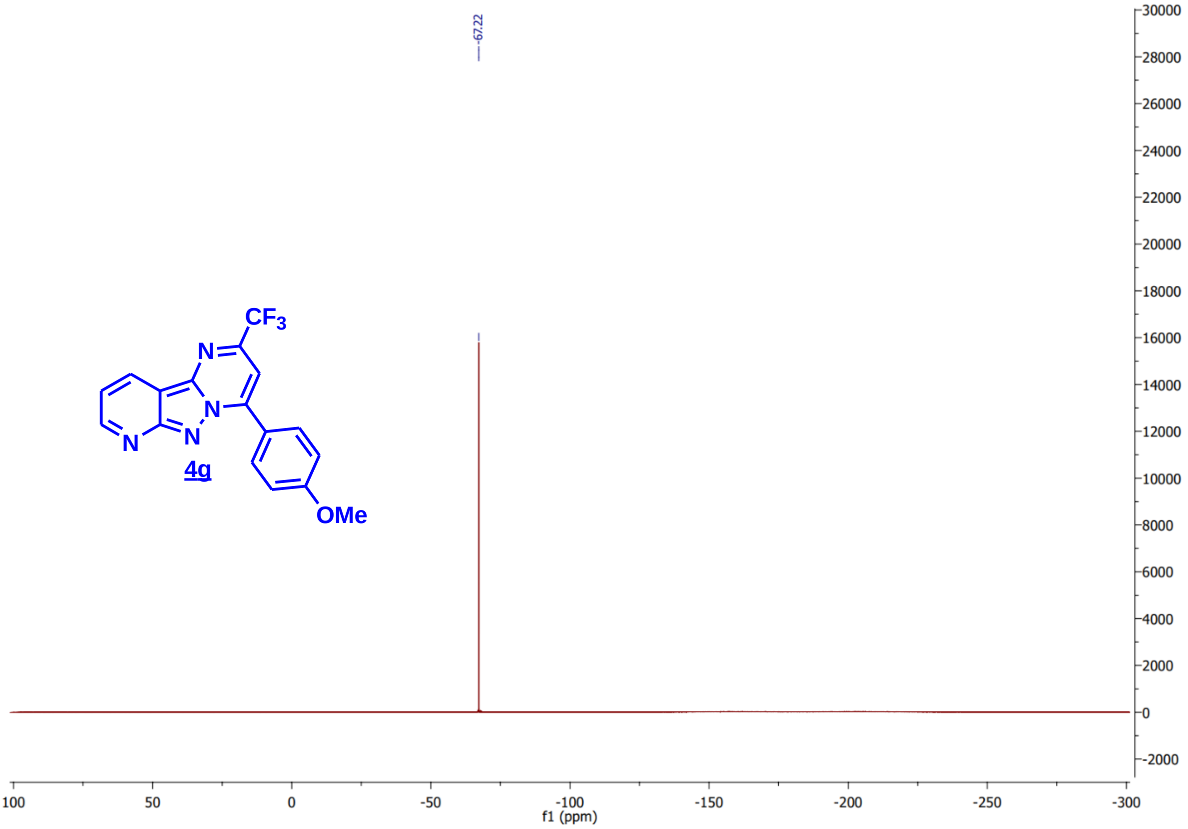

^{13}C NMR (75 MHz, CDCl3)

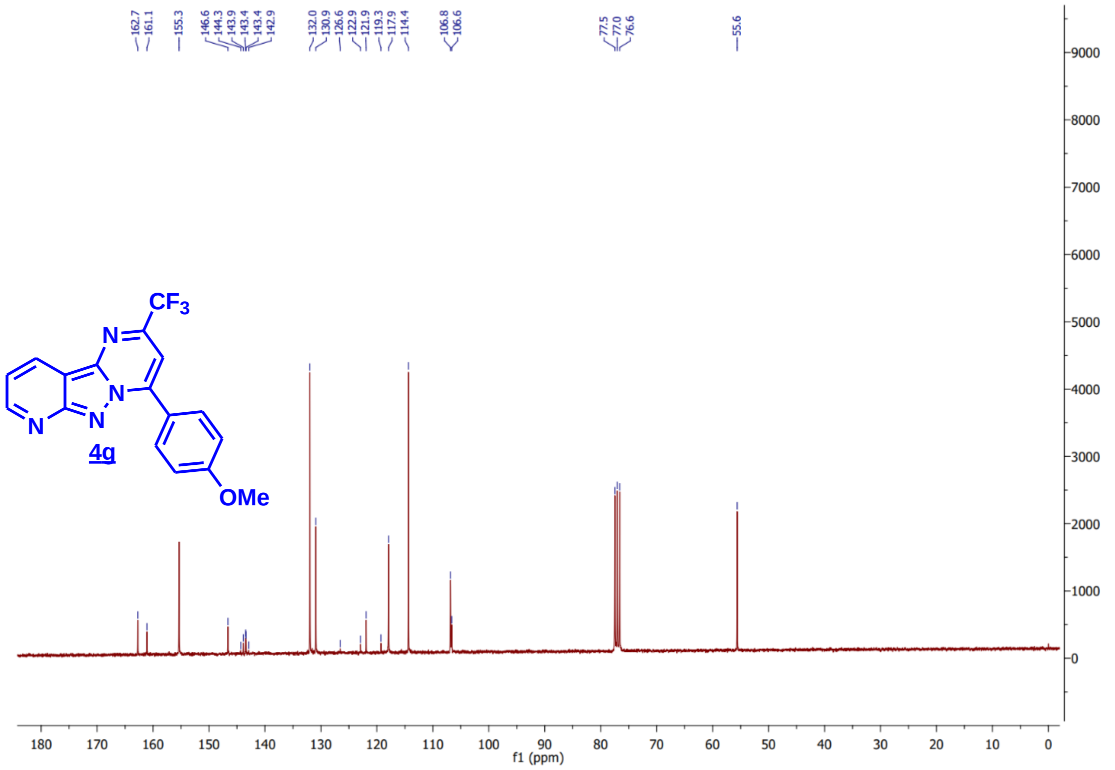

7-(4-methoxyphenyl)-9-(trifluoromethyl)pyrazino[2',3':3,4]pyrazolo[1,5-*a*]pyrimidine (4h)

^{1}H NMR (300 MHz, CDCl3)

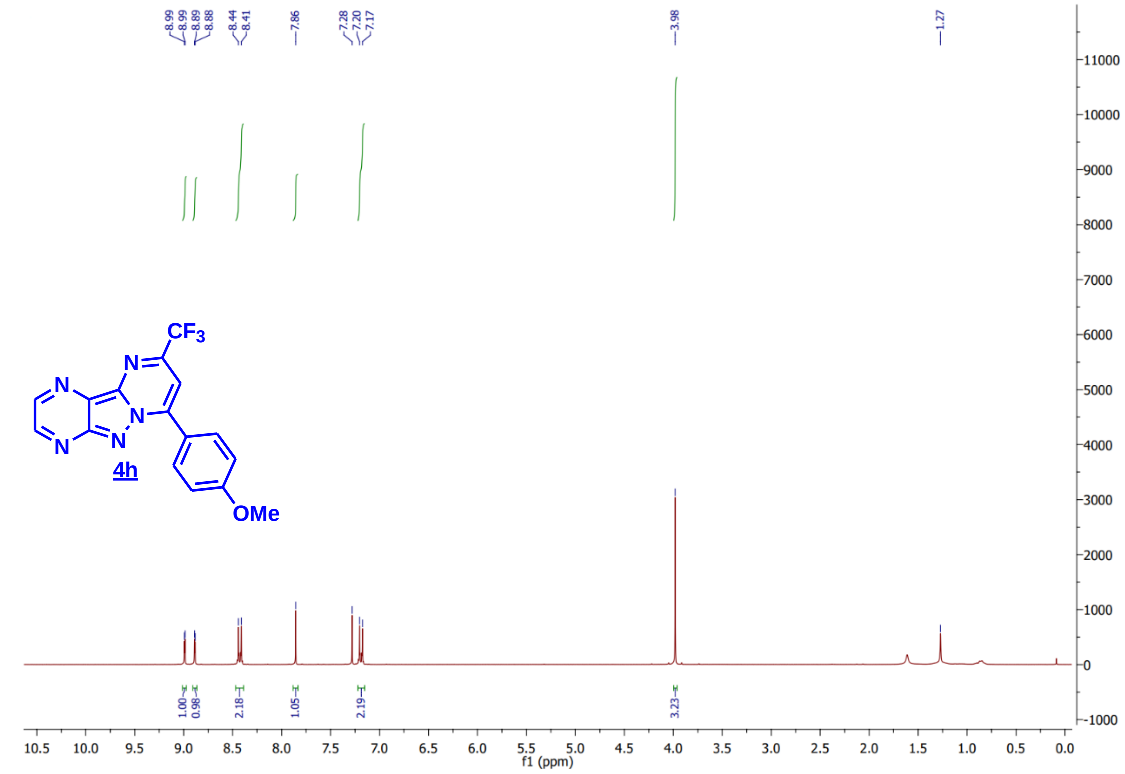

^{19}F NMR (282 MHz,CDCl3)

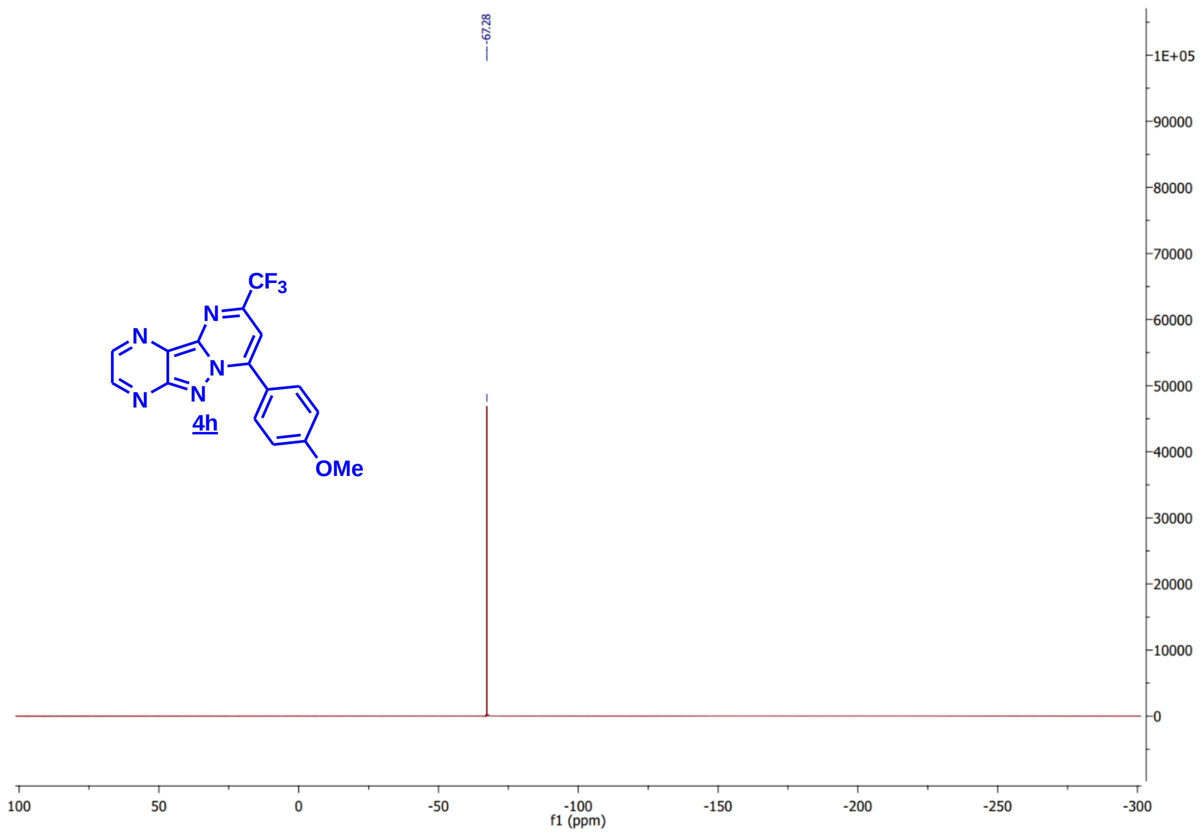

^{13}C NMR (75 MHz, CDCl3)

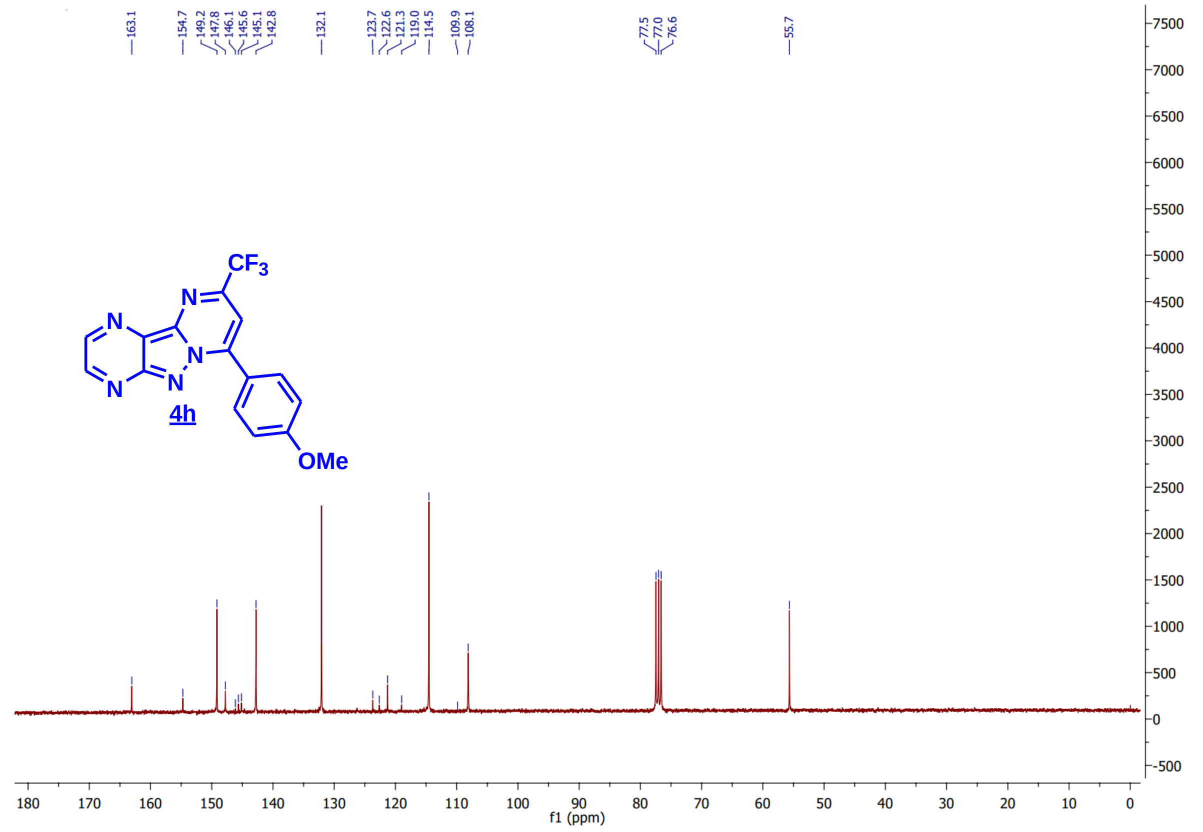

4-*p*-Tolyl-2-trifluoromethyl-pyrimido[1,2-b]indazole (5a)

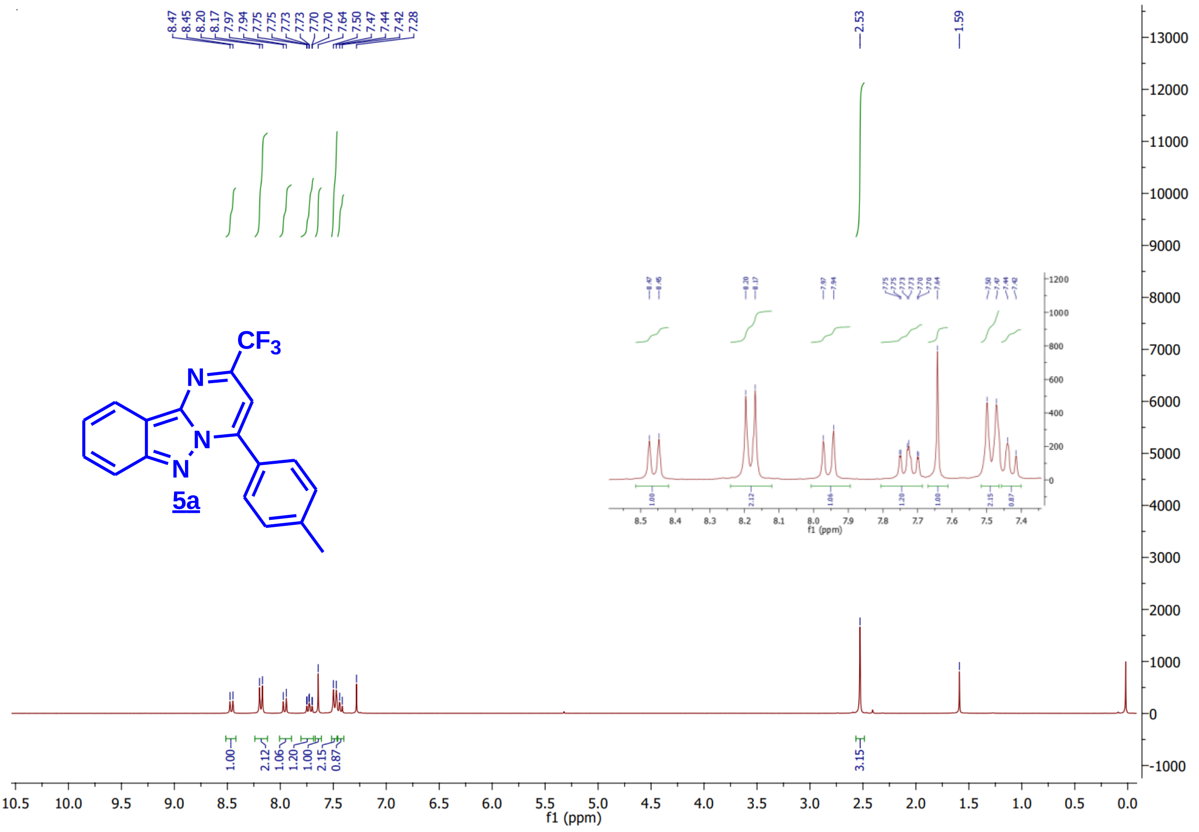

^{19}F NMR (282 MHz,$CDCl_3$)

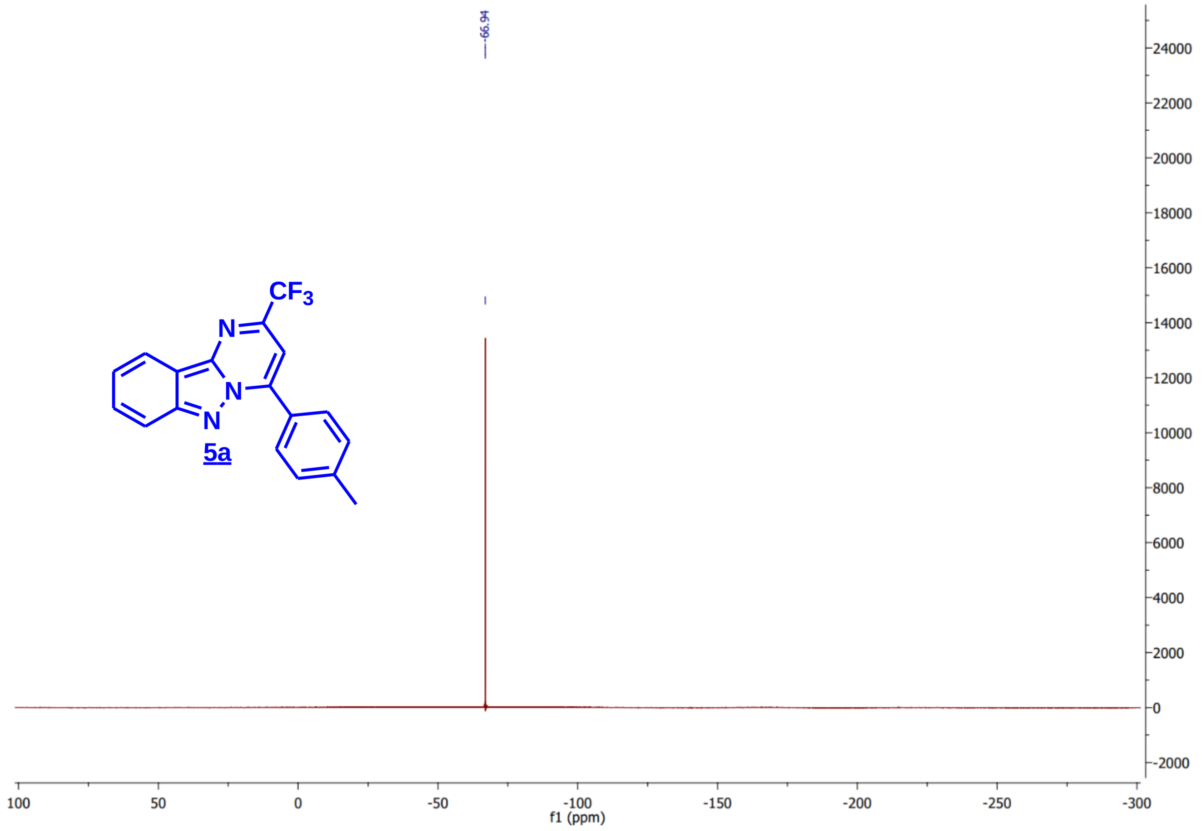

^{13}C NMR (75 MHz, $CDCl_3$)

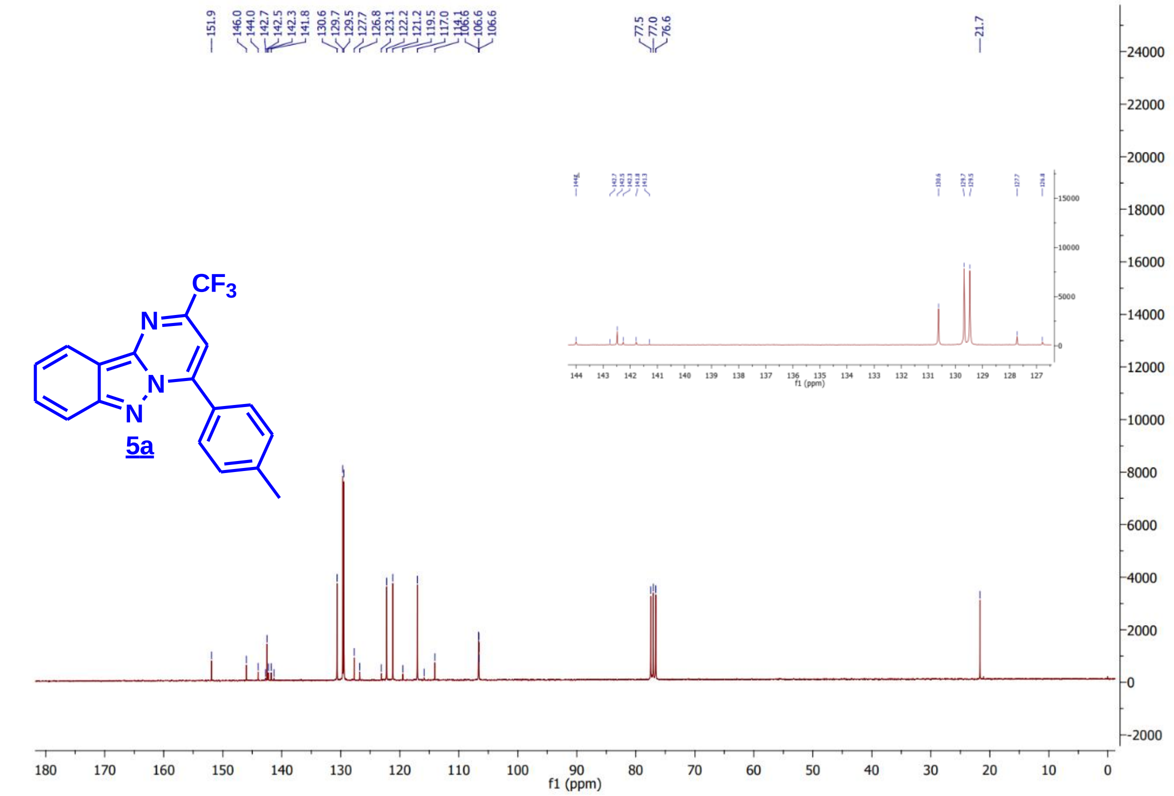

7-(3-methylphenyl)-9-(trifluoromethyl)pyrido[3',2':3,4]pyrazolo[1,5-*a*]pyrimidine (5b)

^{1}H NMR (300 MHz, $CDCl_3$)

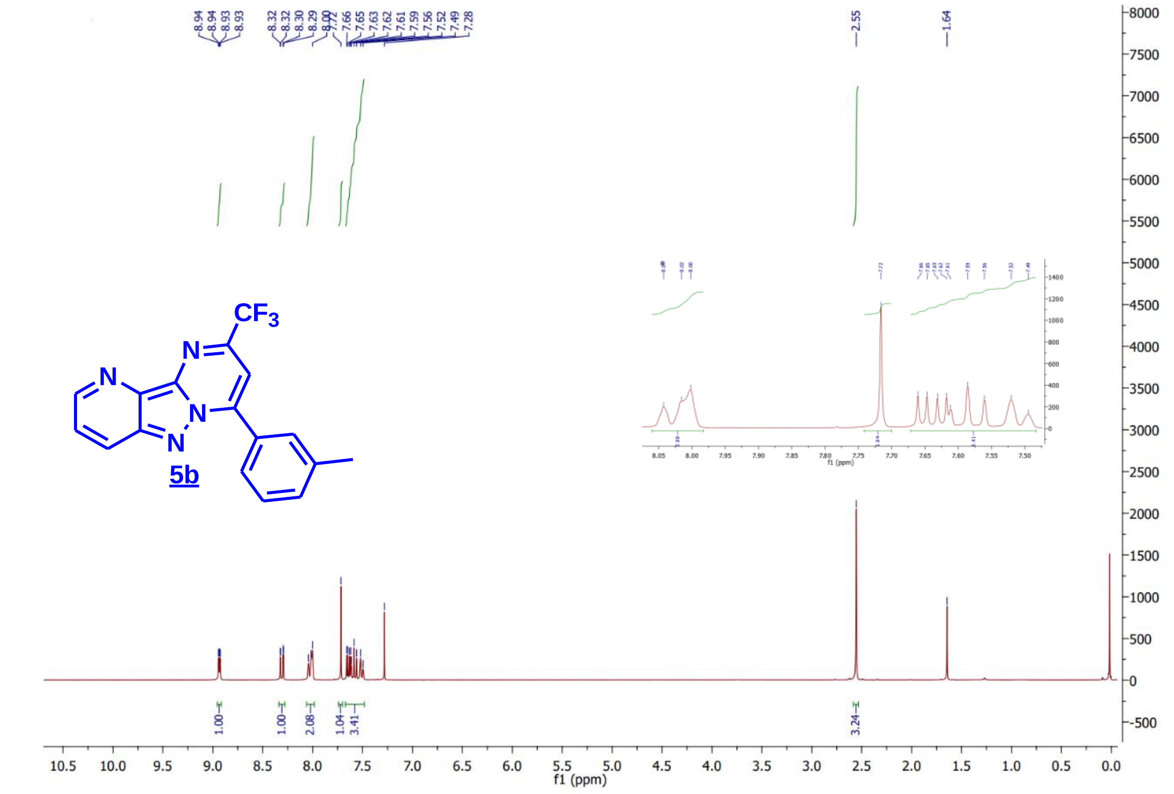

^{19}F NMR (282 MHz,CDCl$_3$)

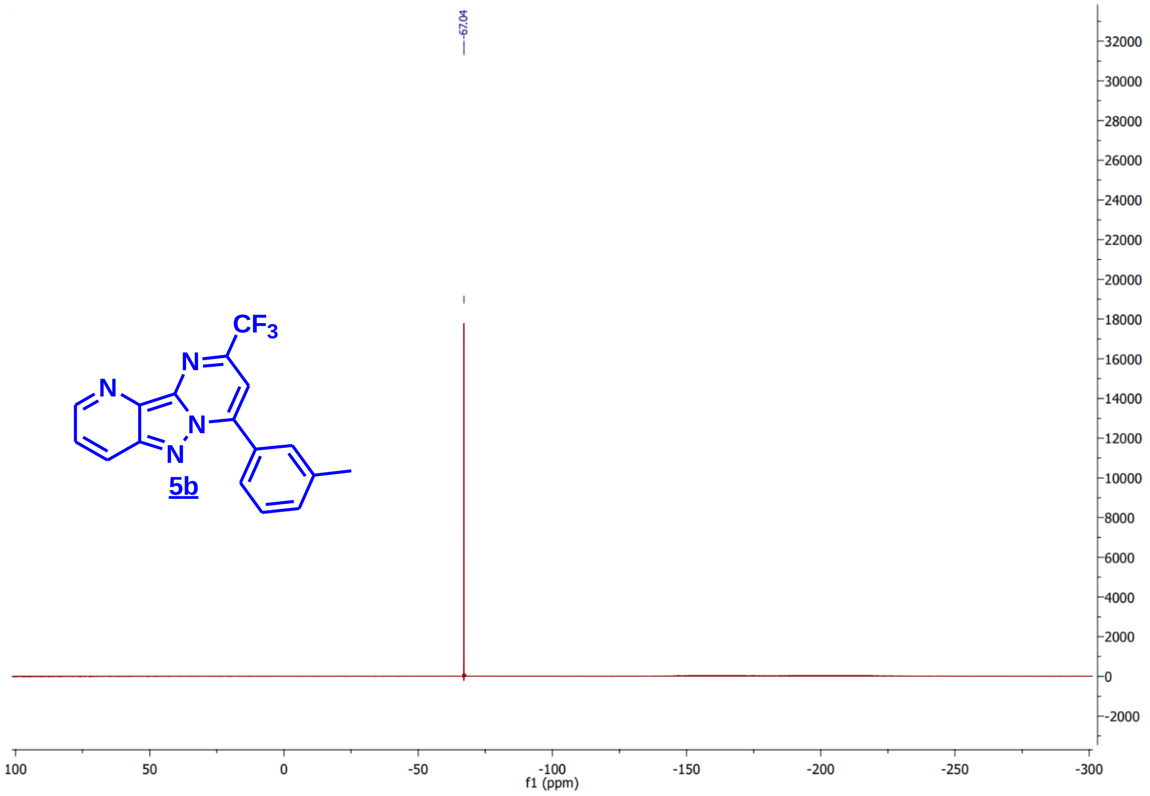

^{13}C NMR (75 MHz, CDCl$_3$)

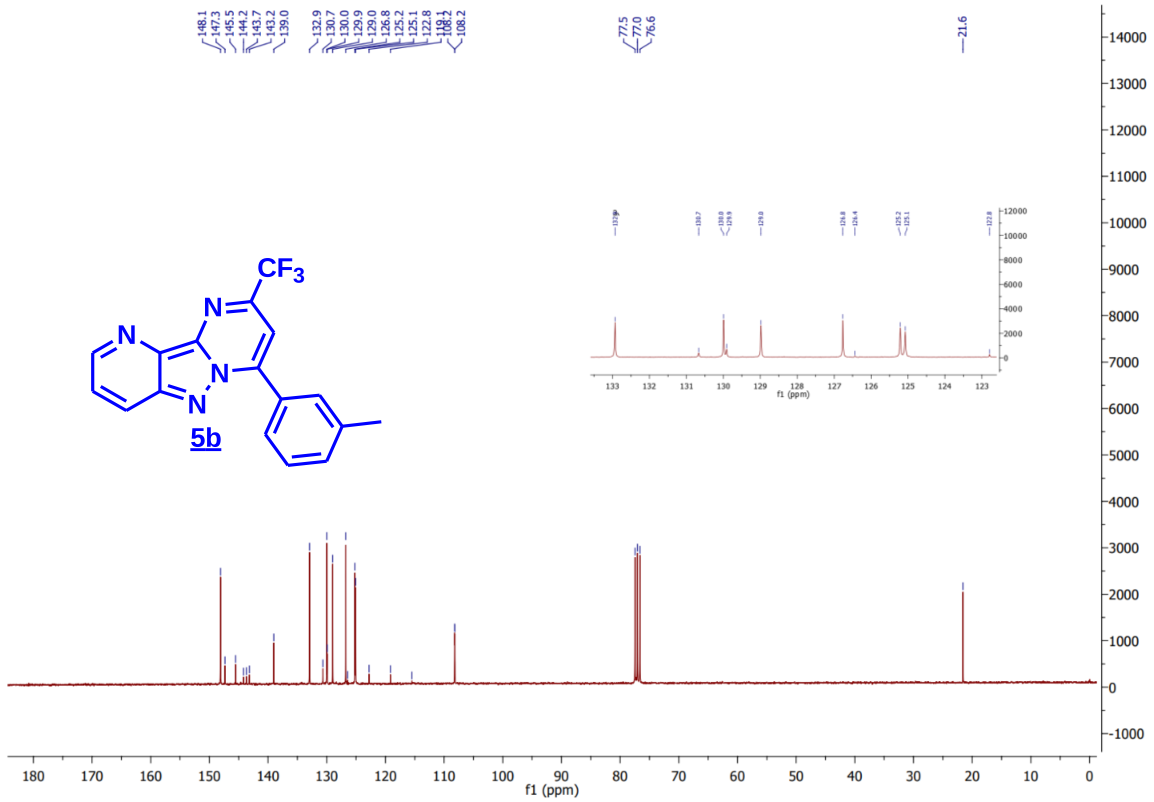

4-(4-tert-Butyl-phenyl)-2,8-bis-trifluoromethyl-pyrimido[1,2-b]indazole (5c)

^{1}H NMR (300 MHz, $CDCl_3$)

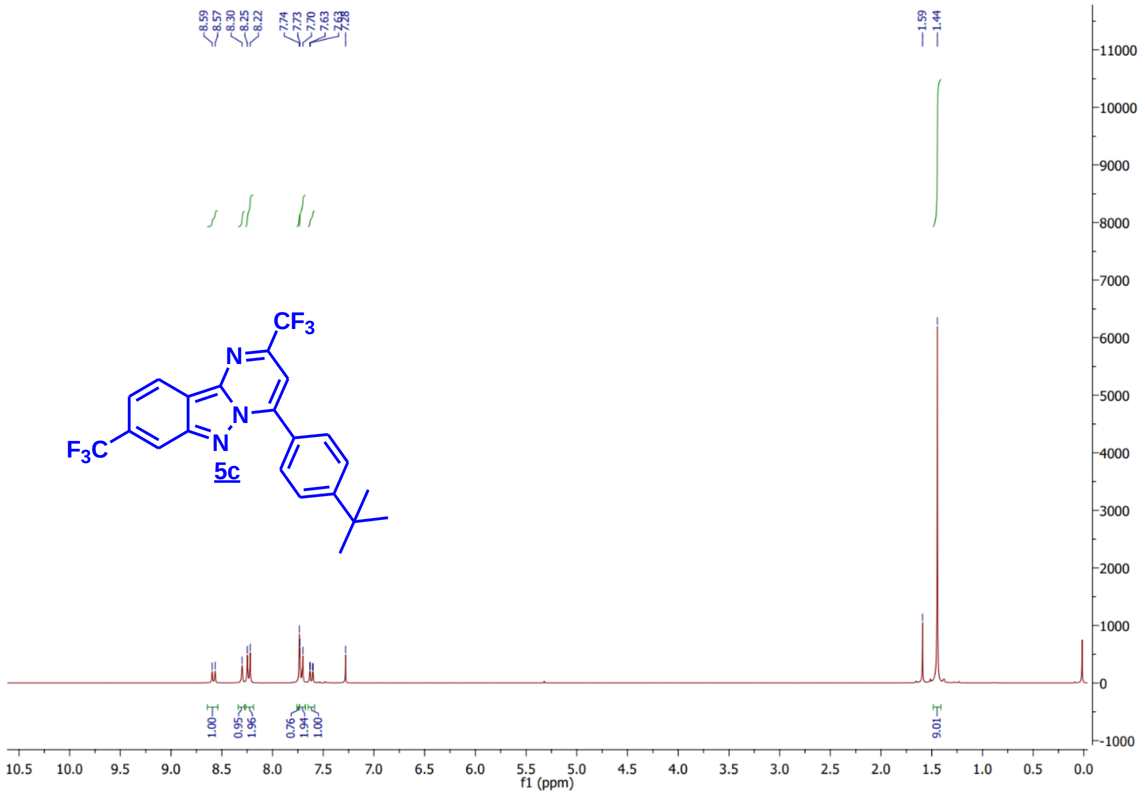

^{19}F NMR (282 MHz,$CDCl_3$)

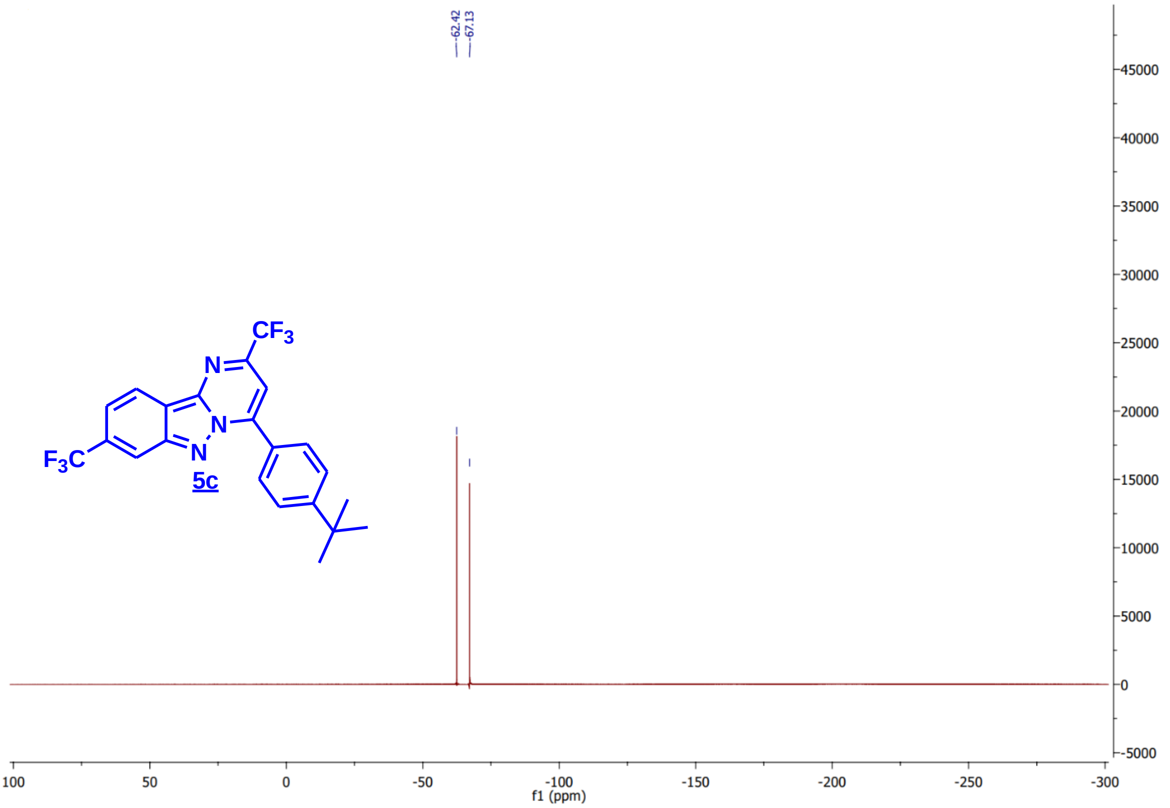

^{13}C NMR (75 MHz, $CDCl_3$)

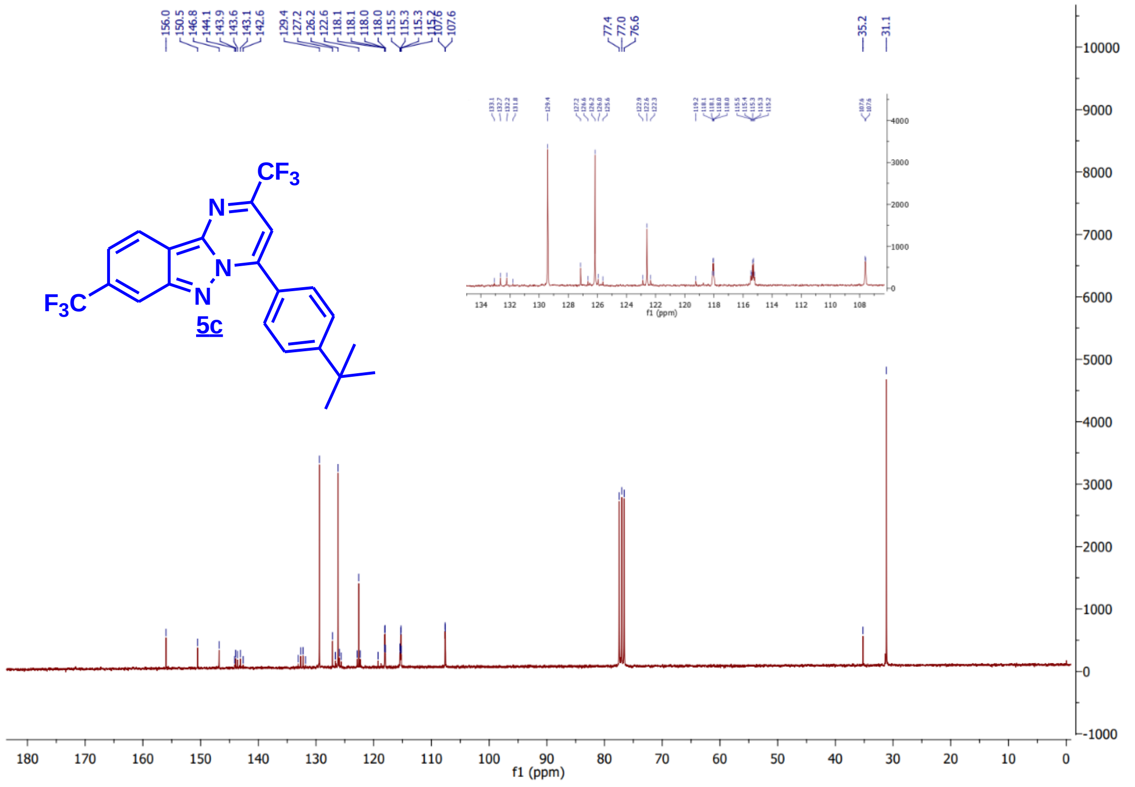

7-(3-methoxyphenyl)-9-(trifluoromethyl)pyrido[3',2':3,4]pyrazolo[1,5-*a*]pyrimidine (5d)

^{1}H NMR (300 MHz, $CDCl_3$)

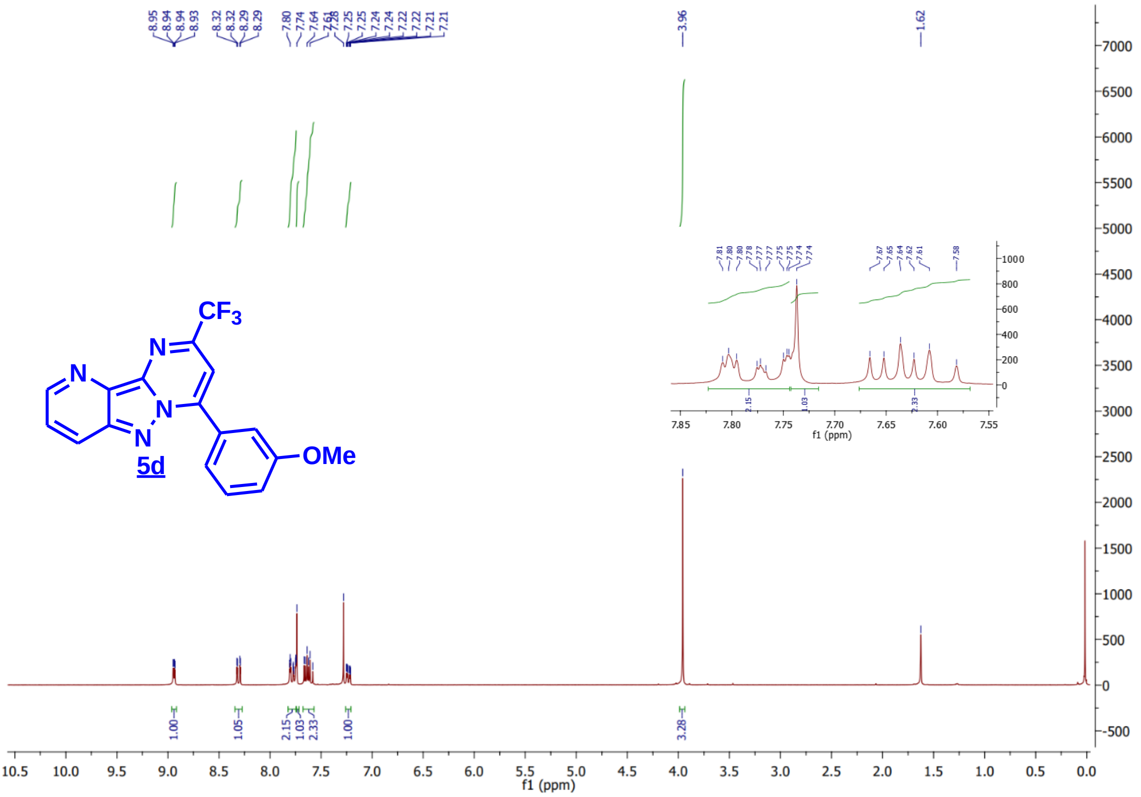

^{19}F NMR (282 MHz,CDCl$_3$)

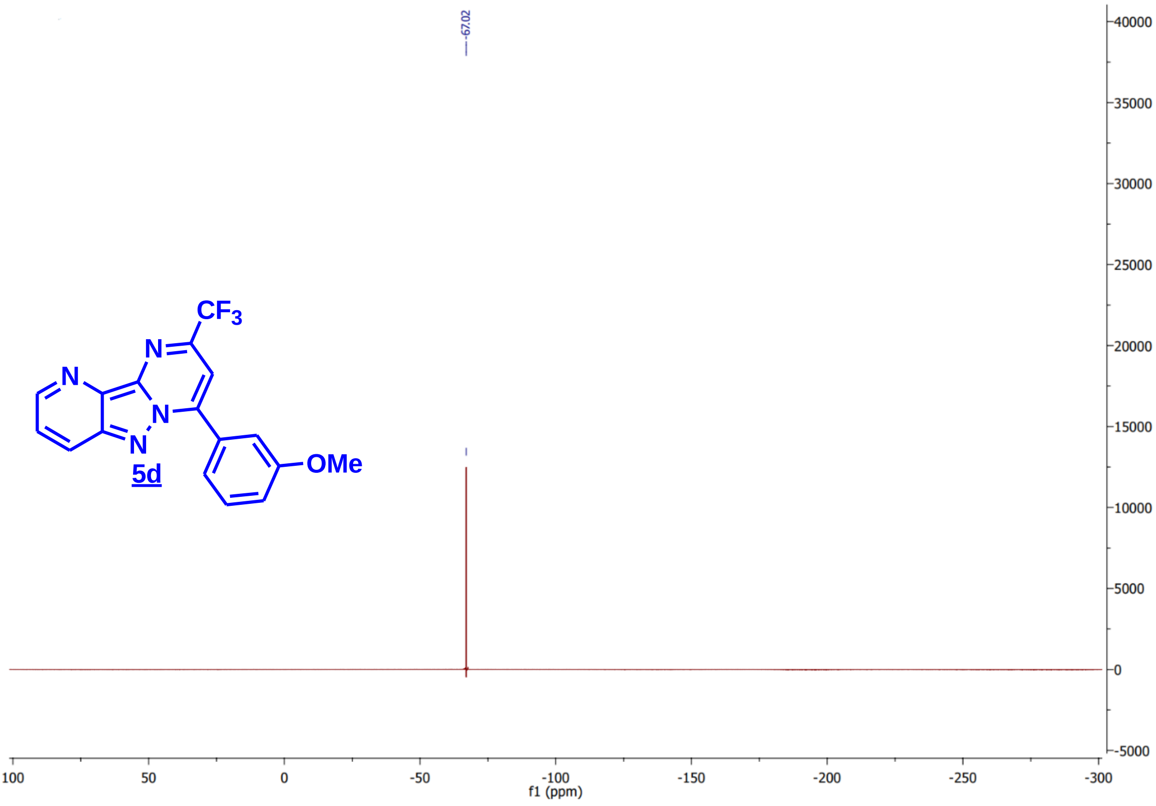

^{13}C NMR (75 MHz, CDCl$_3$)

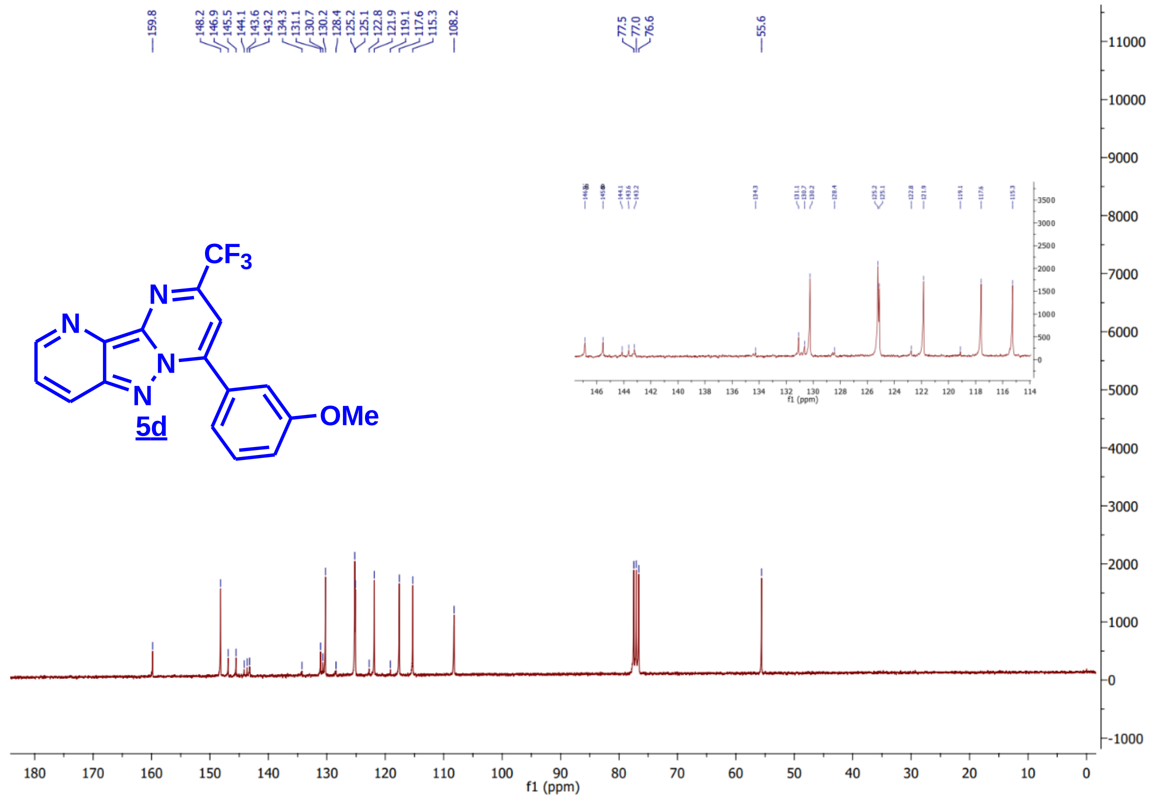

7-(3,4-dimethoxyphenyl)-9-(trifluoromethyl)pyrazino[2',3':3,4]pyrazolo[1,5-*a*]pyrimidine (5e)

^{1}H NMR (300 MHz, $CDCl_3$)

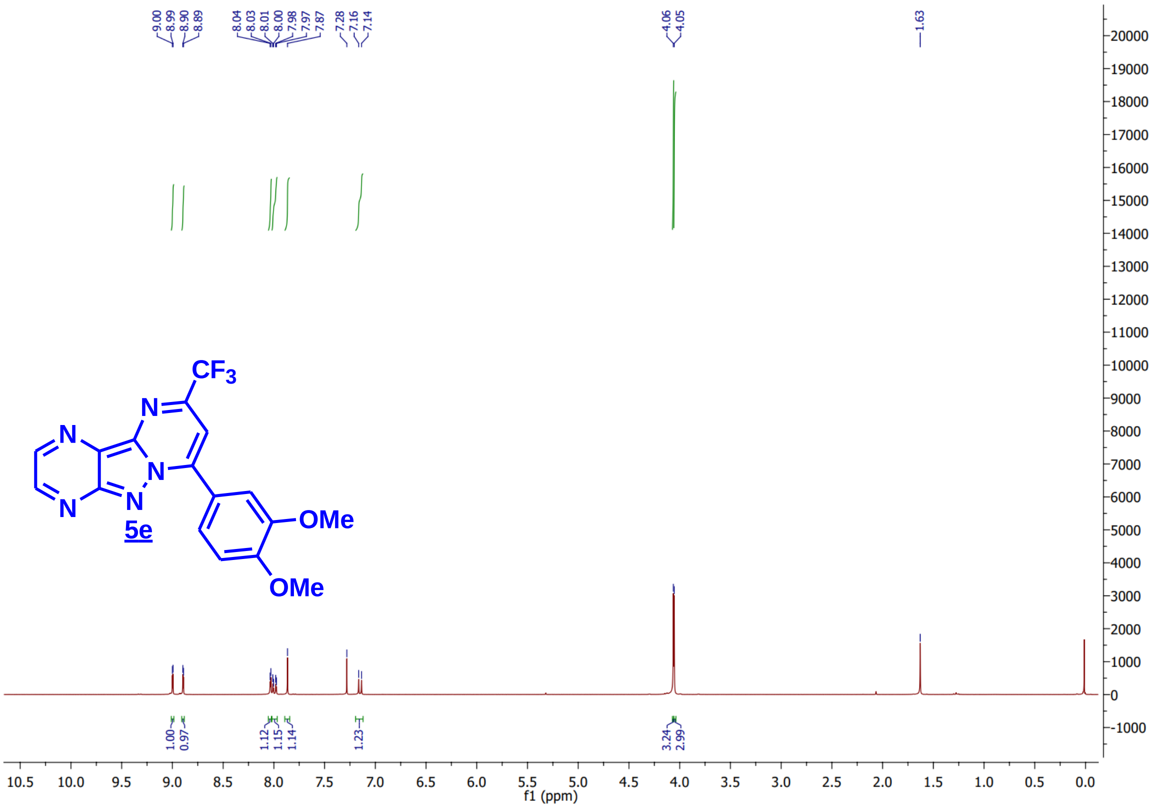

^{19}F NMR (282 MHz,$CDCl_3$)

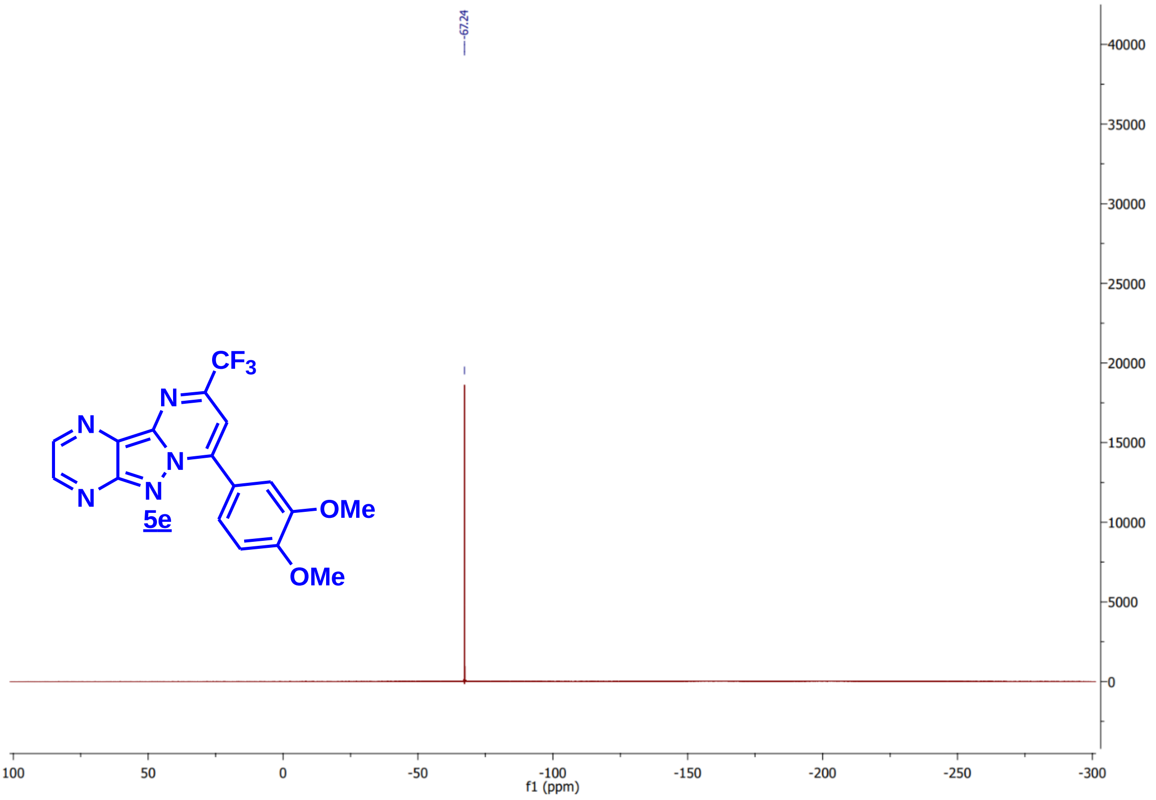

^{13}C NMR (75 MHz, $CDCl_3$)

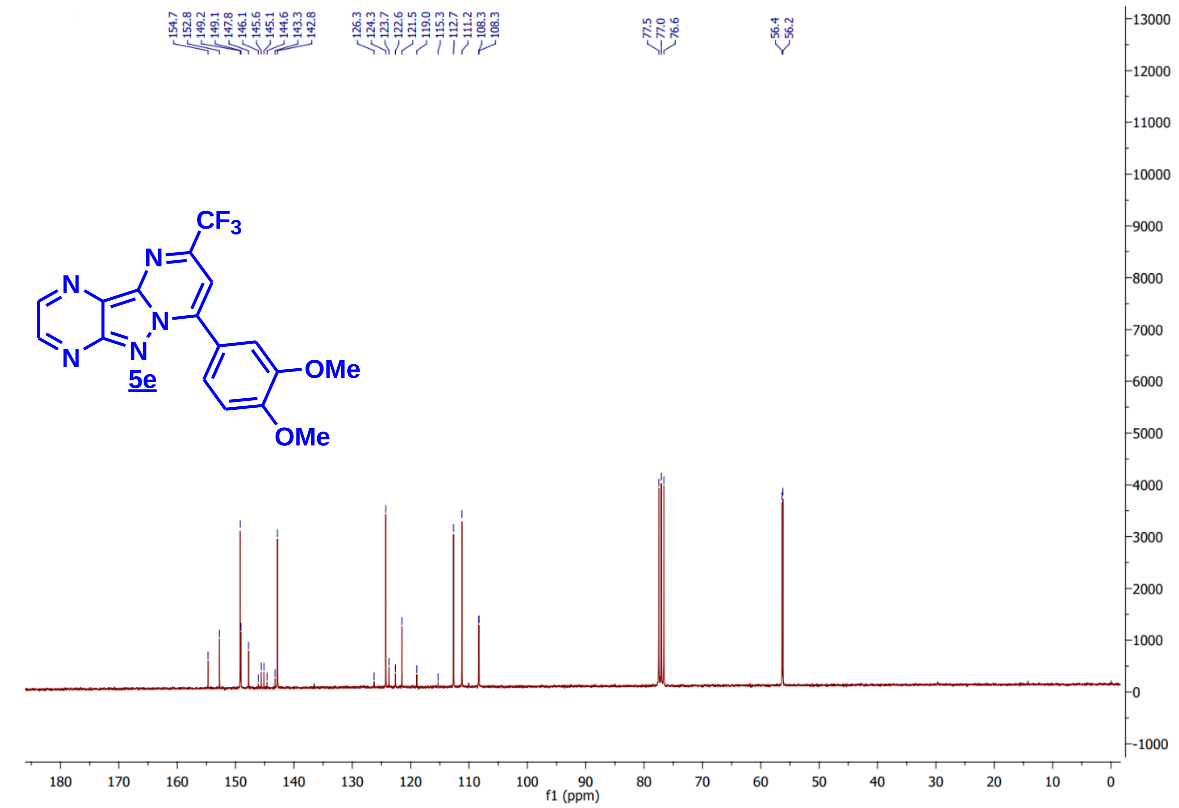

7-(2,5-dimethoxyphenyl)-9-(trifluoromethyl)pyrazino[2',3':3,4]pyrazolo[1,5-*a*]pyrimidine (5f)

^{1}H NMR (300 MHz, $CDCl_3$)

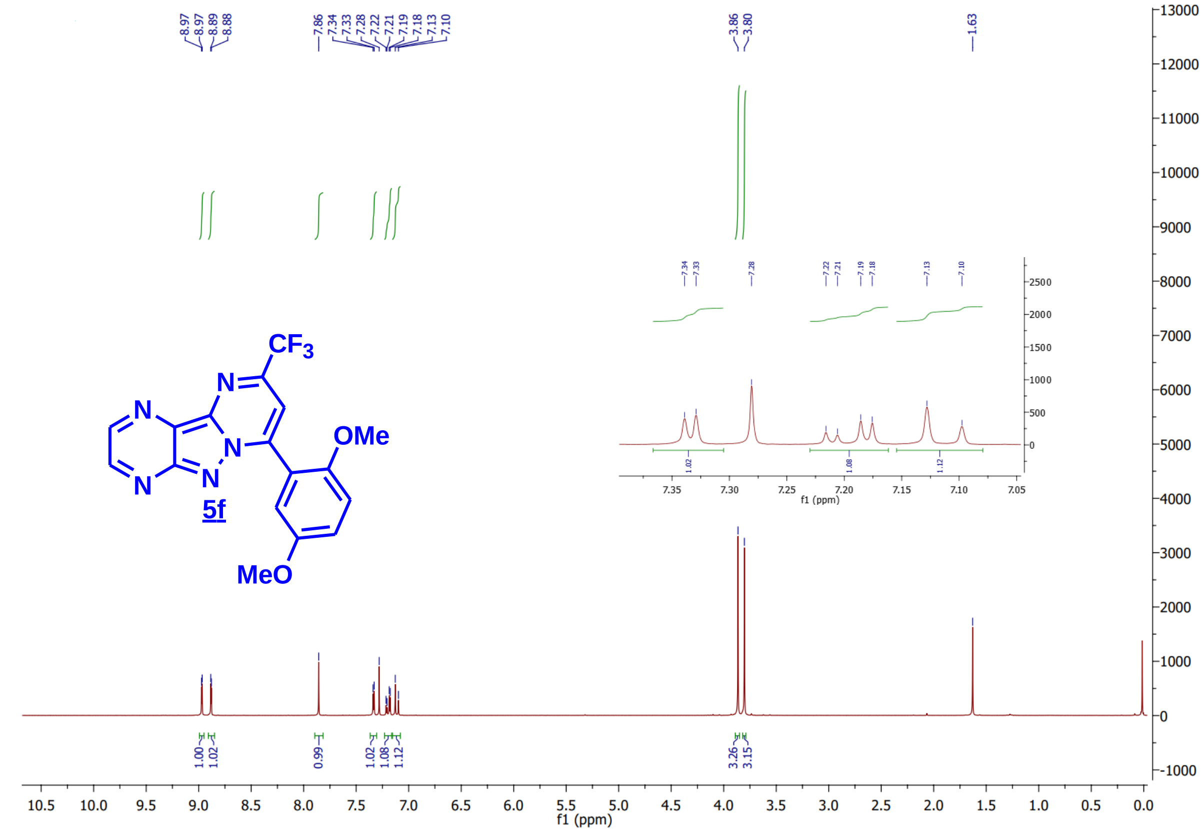

^{19}F NMR (282 MHz,CDCl3)

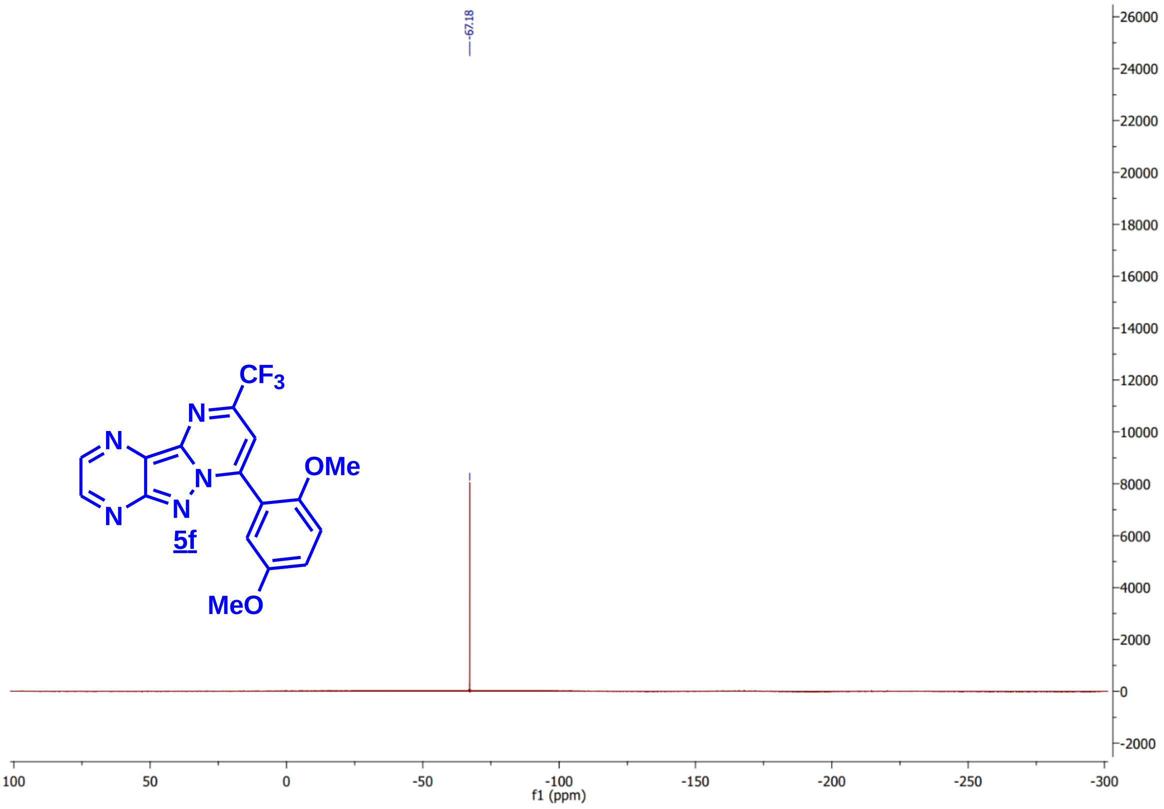

^{13}C NMR (75 MHz, CDCl3)

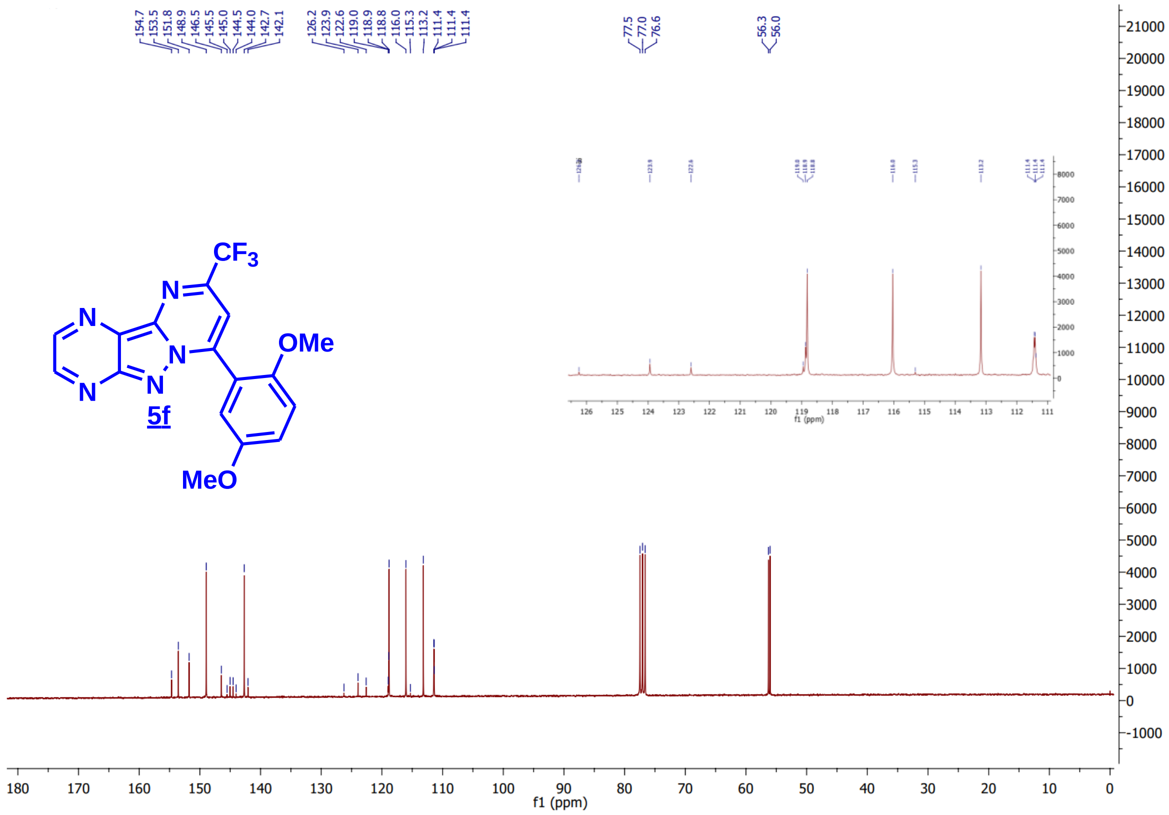

7-(2,4-dimethoxyphenyl)-9-(trifluoromethyl)pyrazino[2',3':3,4]pyrazolo[1,5-*a*]pyrimidine (5g)

^{1}H NMR (300 MHz, $CDCl_3$)

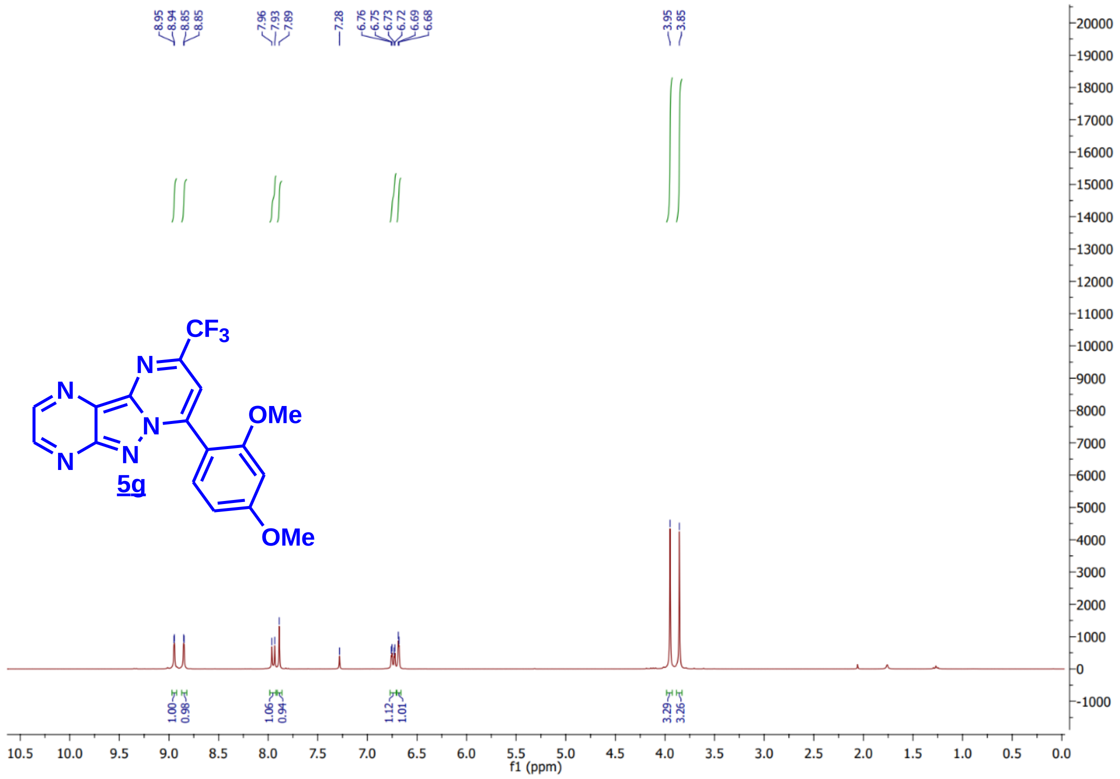

^{19}F NMR (282 MHz,$CDCl_3$)

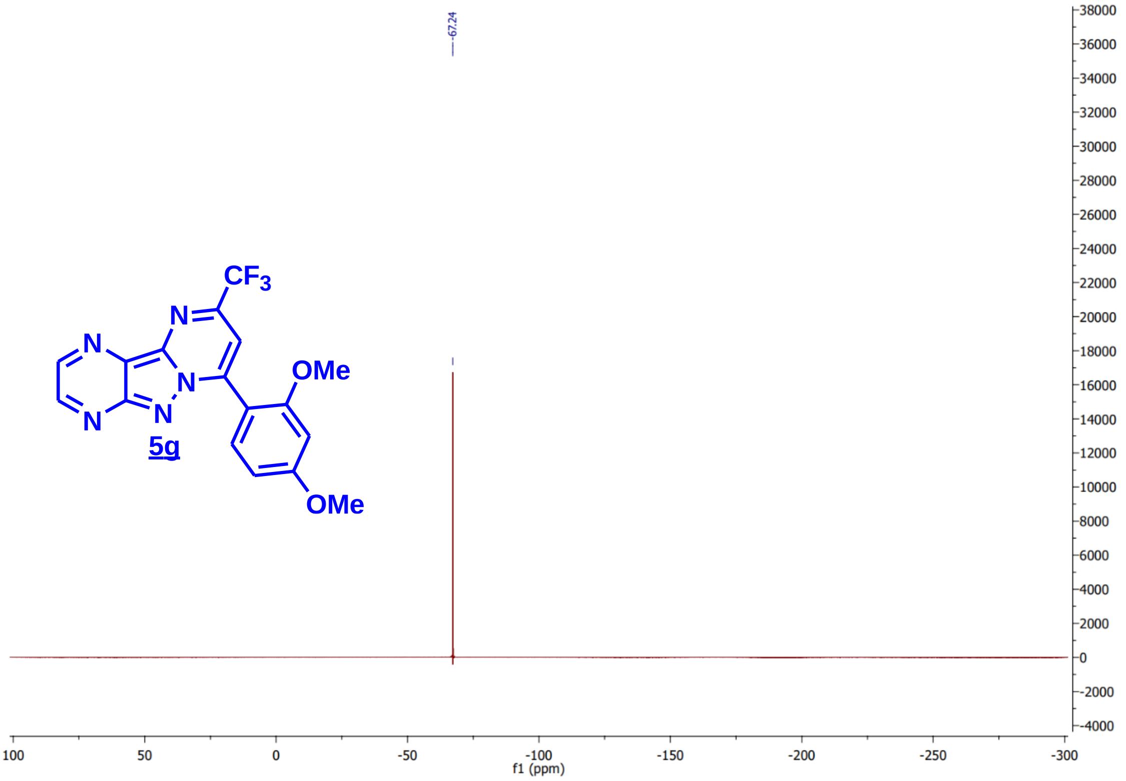

^{13}C NMR (75 MHz, $CDCl_3$)

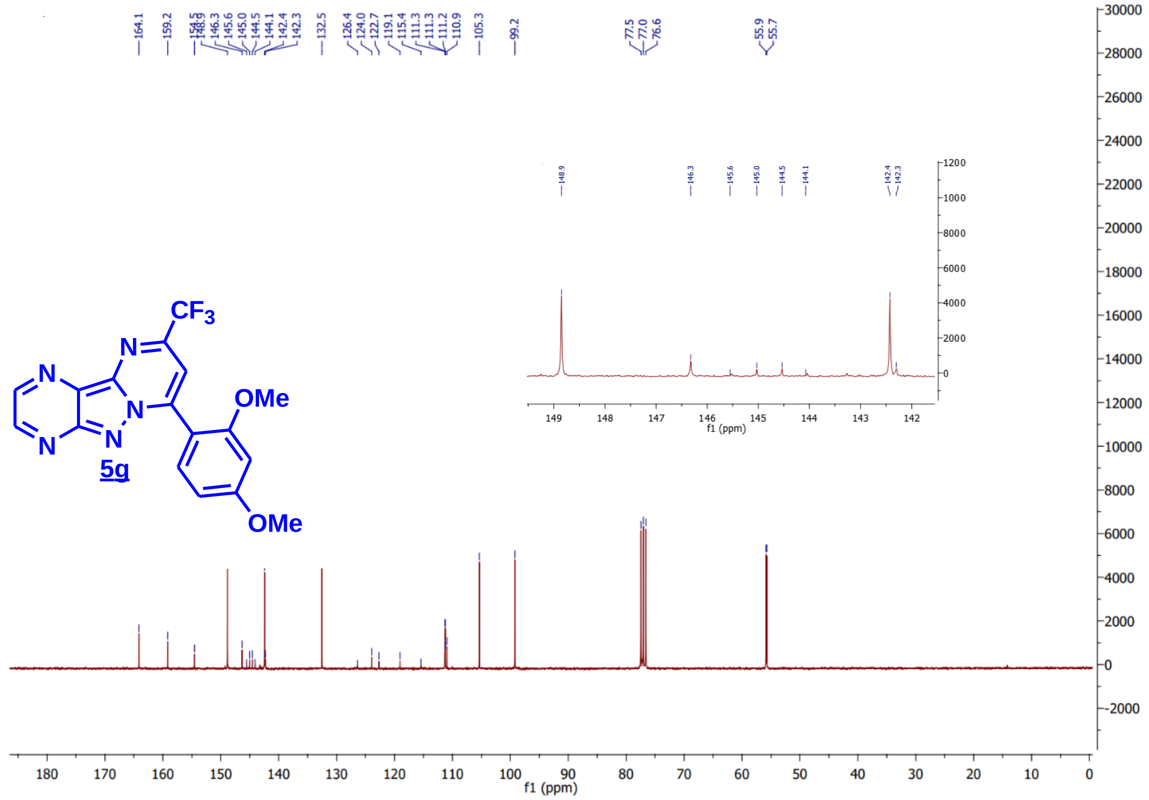

7-[4-(methylsulfanyl)phenyl]-9-(trifluoromethyl)pyrido[3',2':3,4]pyrazolo[1,5-*a*]pyrimidine (5h)

^{1}H NMR (300 MHz, $CDCl_3$)

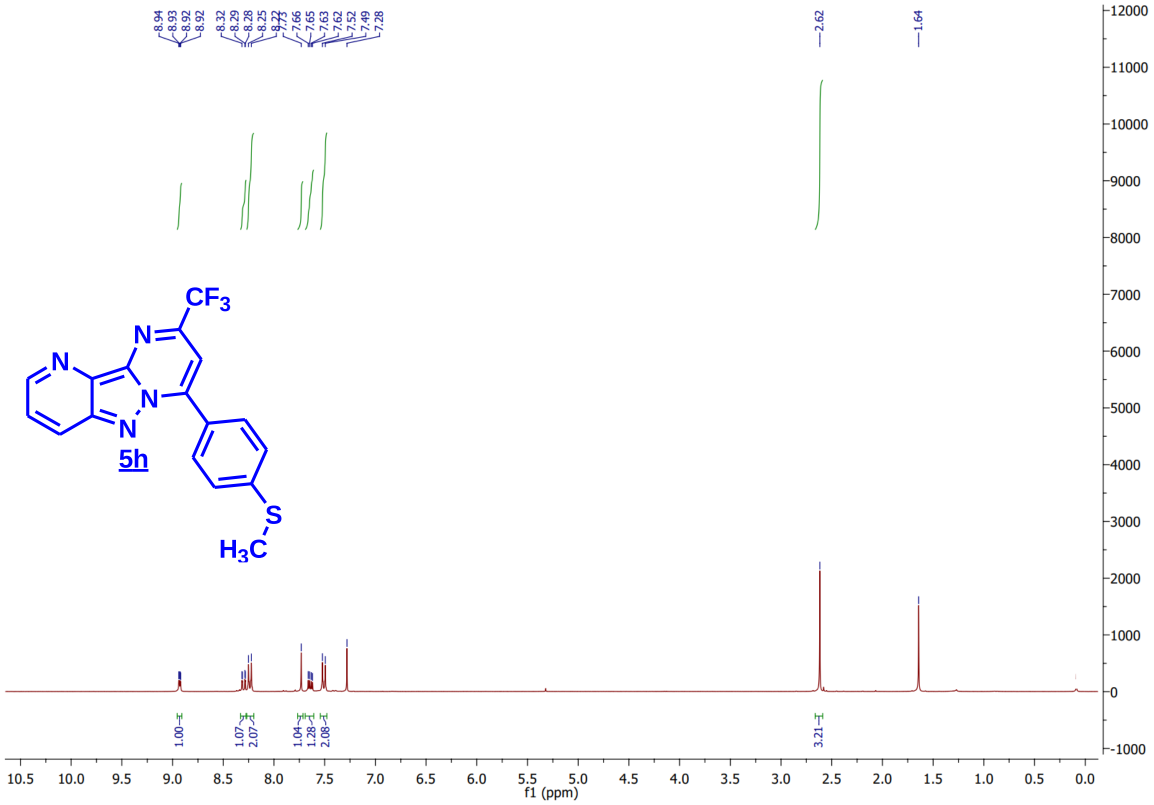

^{19}F NMR (282 MHz,$CDCl_3$)

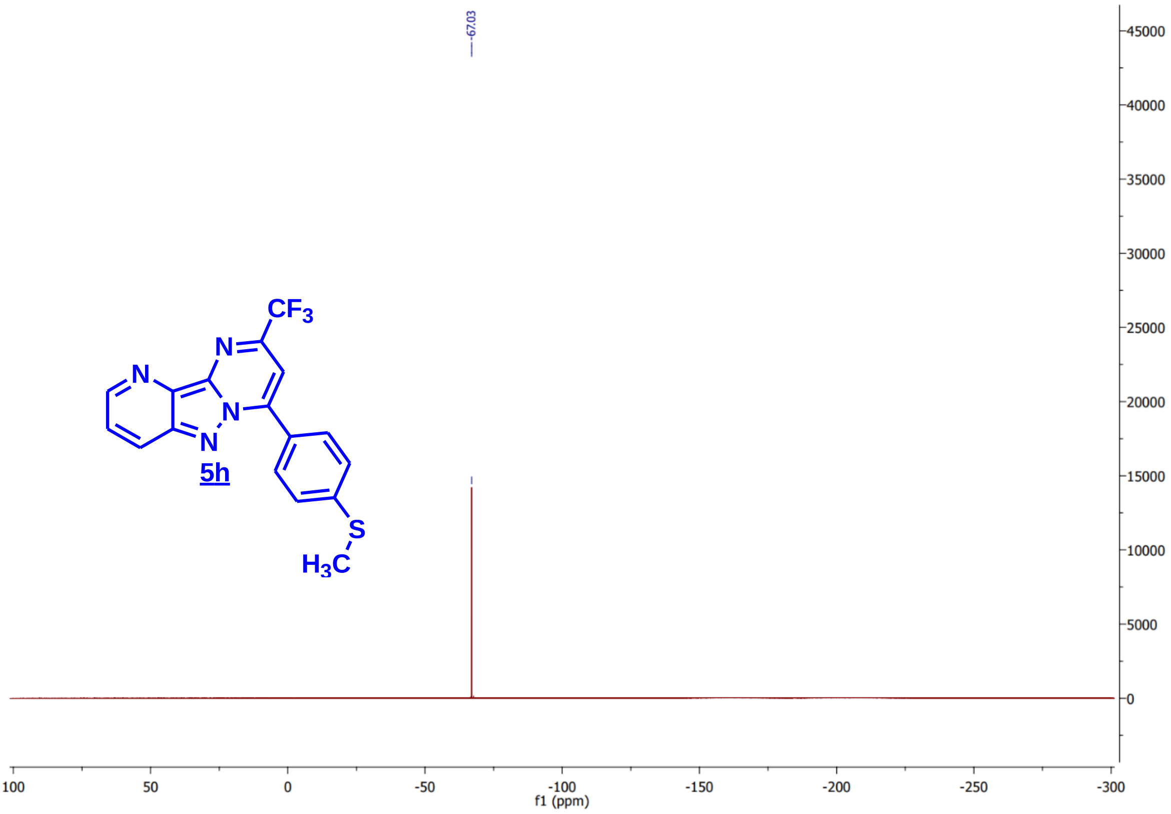

^{13}C NMR (75 MHz, $CDCl_3$)

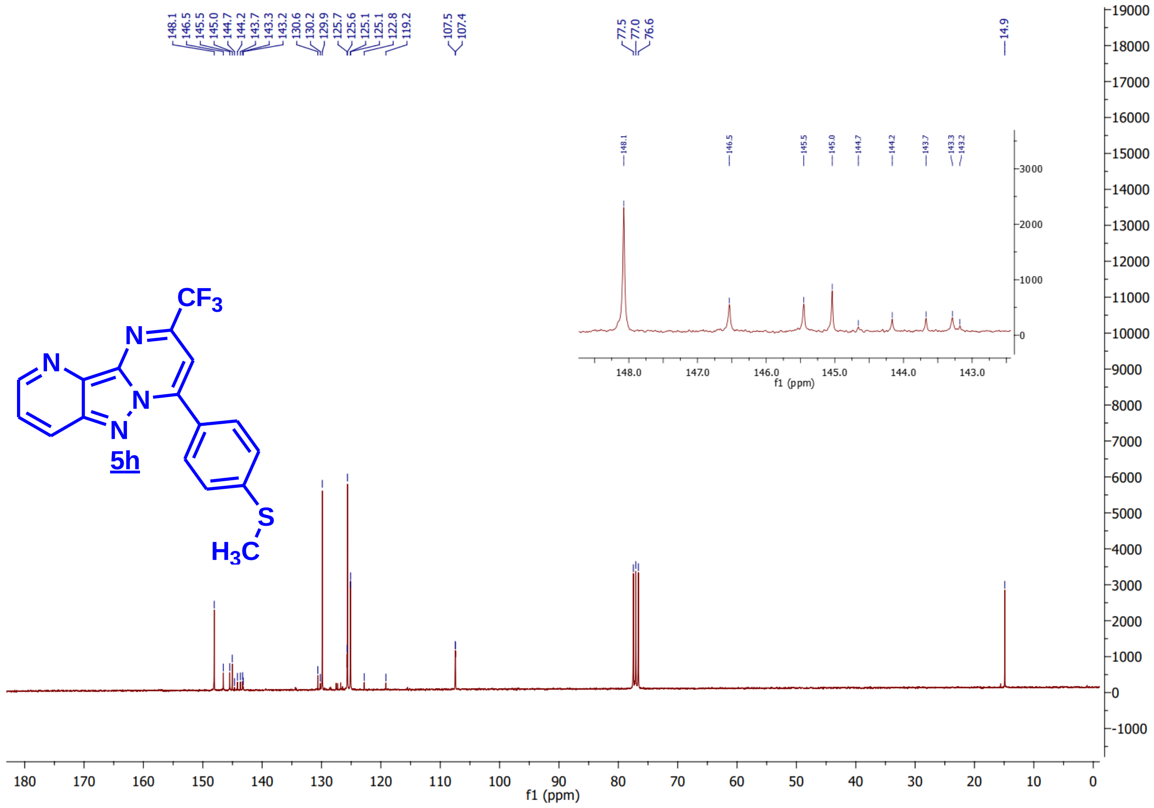

4-(2*H*-1,3-benzodioxol-4-yl)-2-(trifluoromethyl)pyrido[2',3':3,4]pyrazolo[1,5-*a*]pyrimidine (11p)

^{1}H NMR (300 MHz, CDCl$_3$)

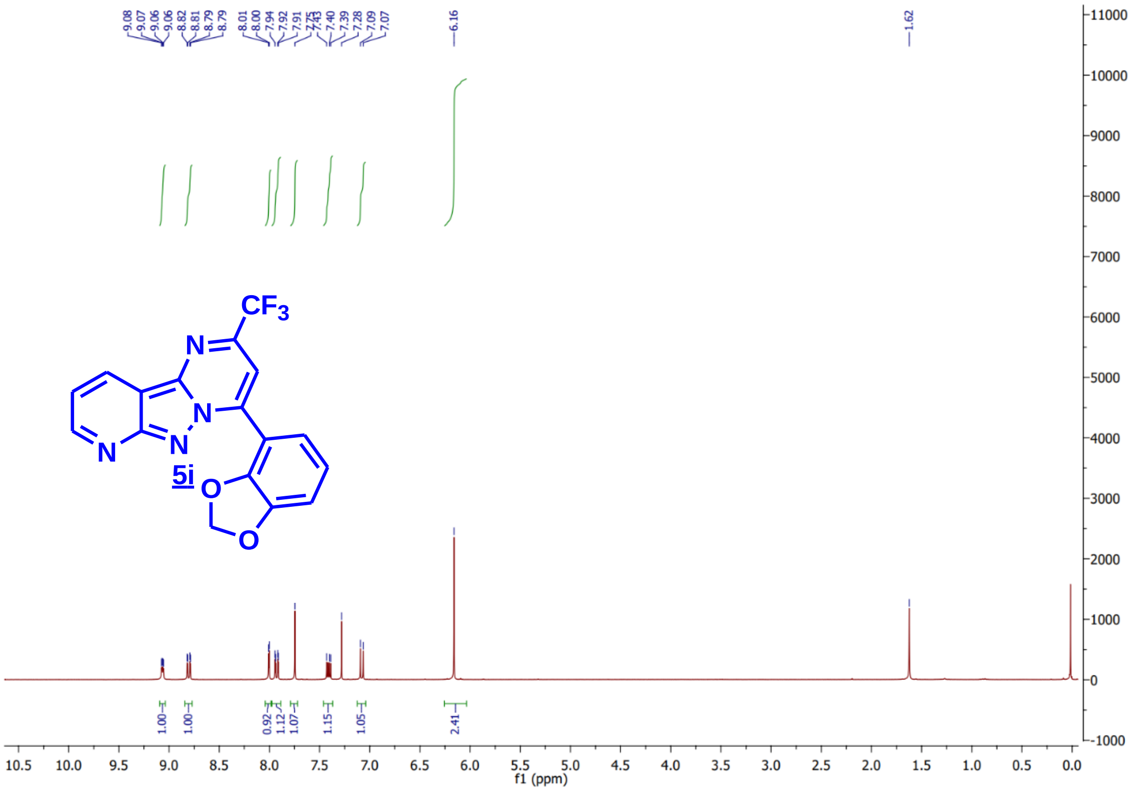

^{19}F NMR (282 MHz,CDCl$_3$)

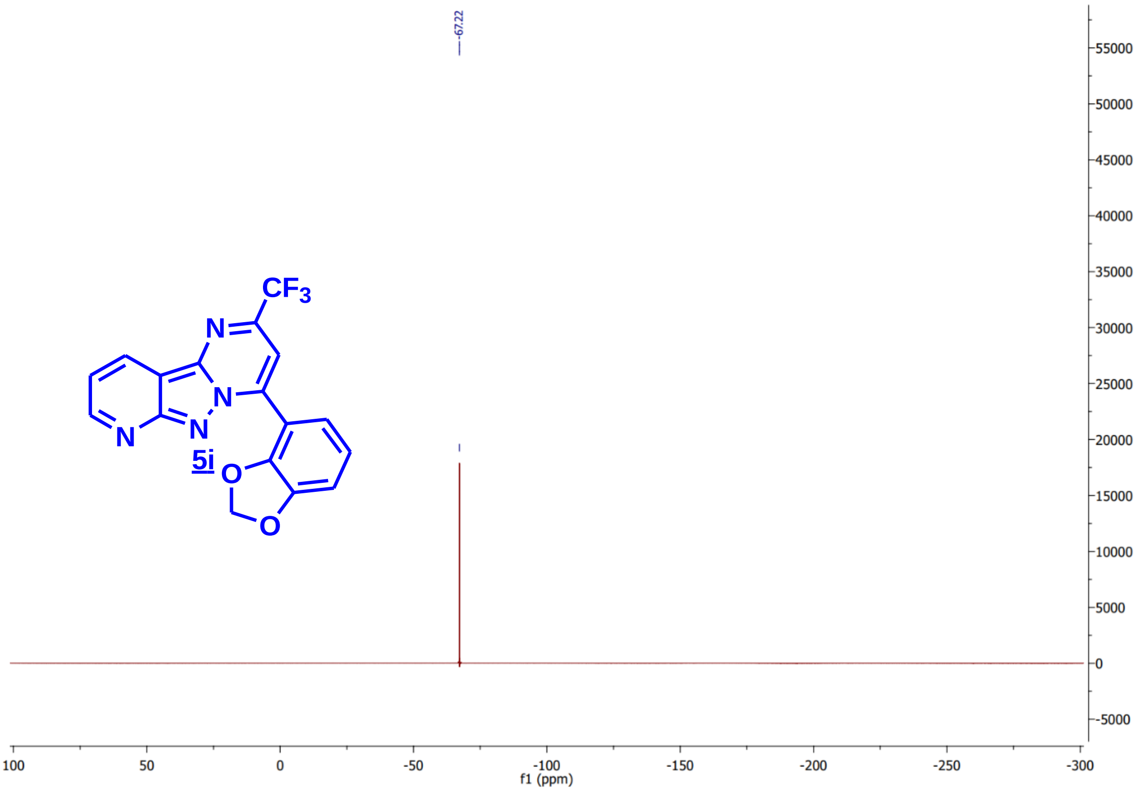

^{13}C NMR (75 MHz, $CDCl_3$)

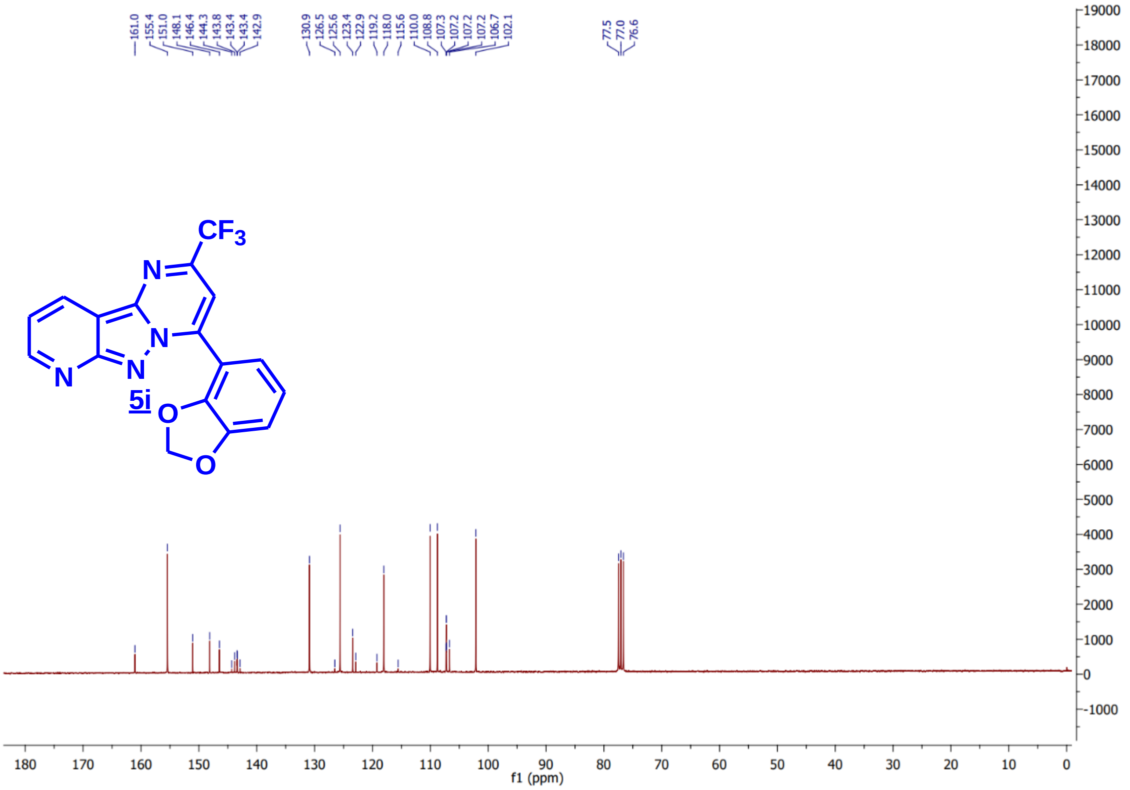

4-(4-Chloro-phenyl)-2,8-bis-trifluoromethyl-pyrimido[1,2-b]indazole (5j)

^1H NMR (300 MHz, $CDCl_3$)

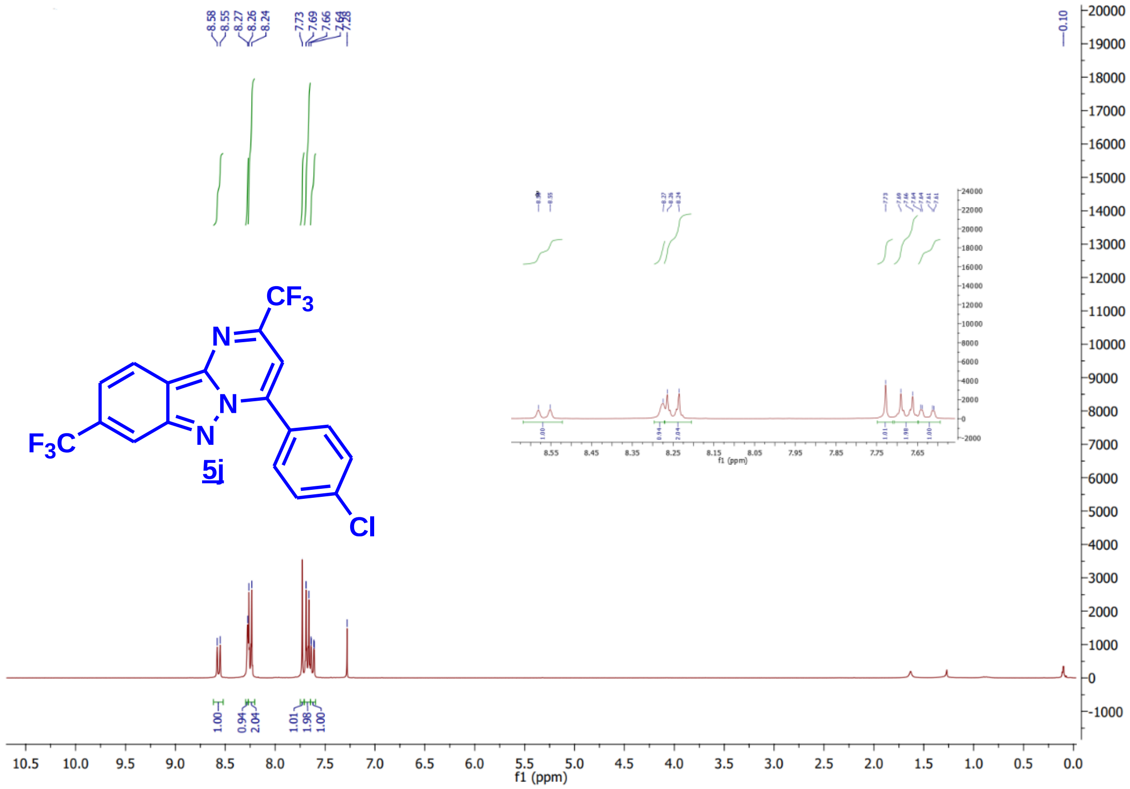

^{19}F NMR (282 MHz,CDCl$_3$)

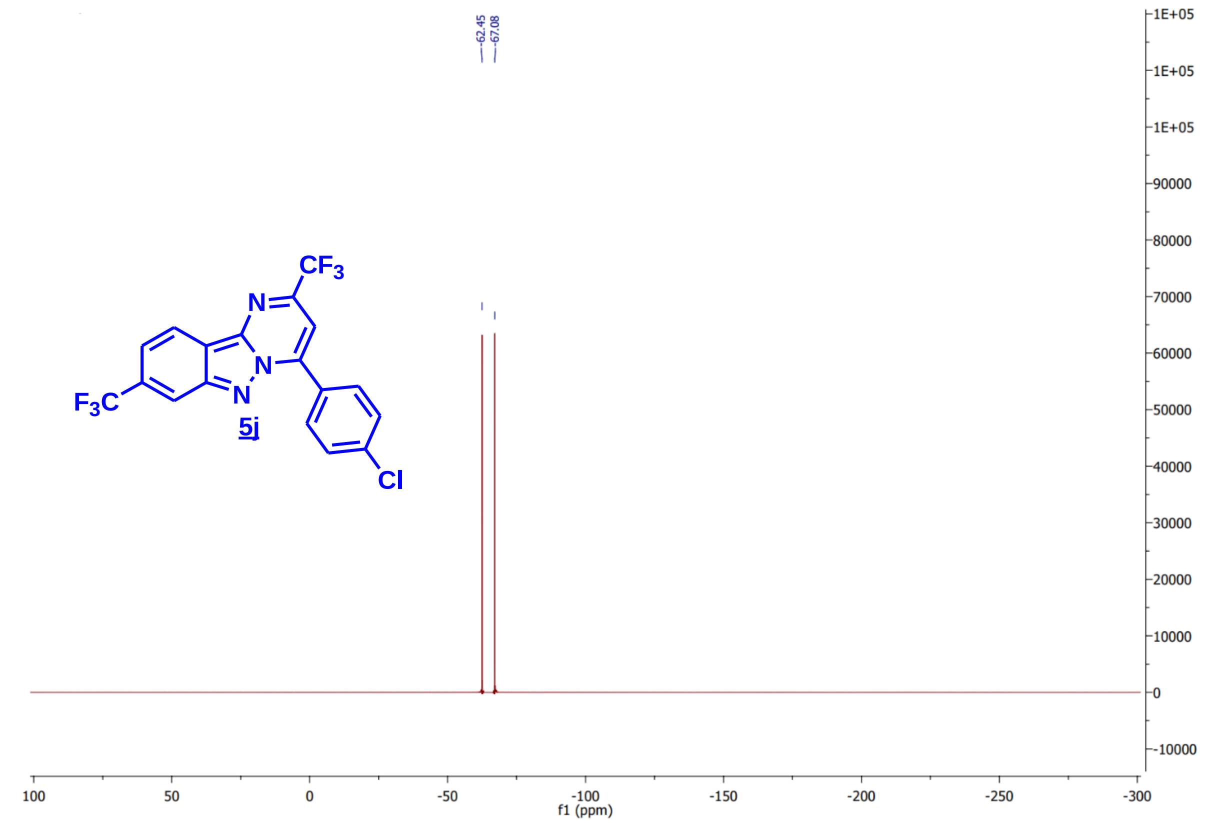

^{13}C NMR (75 MHz, CDCl$_3$)

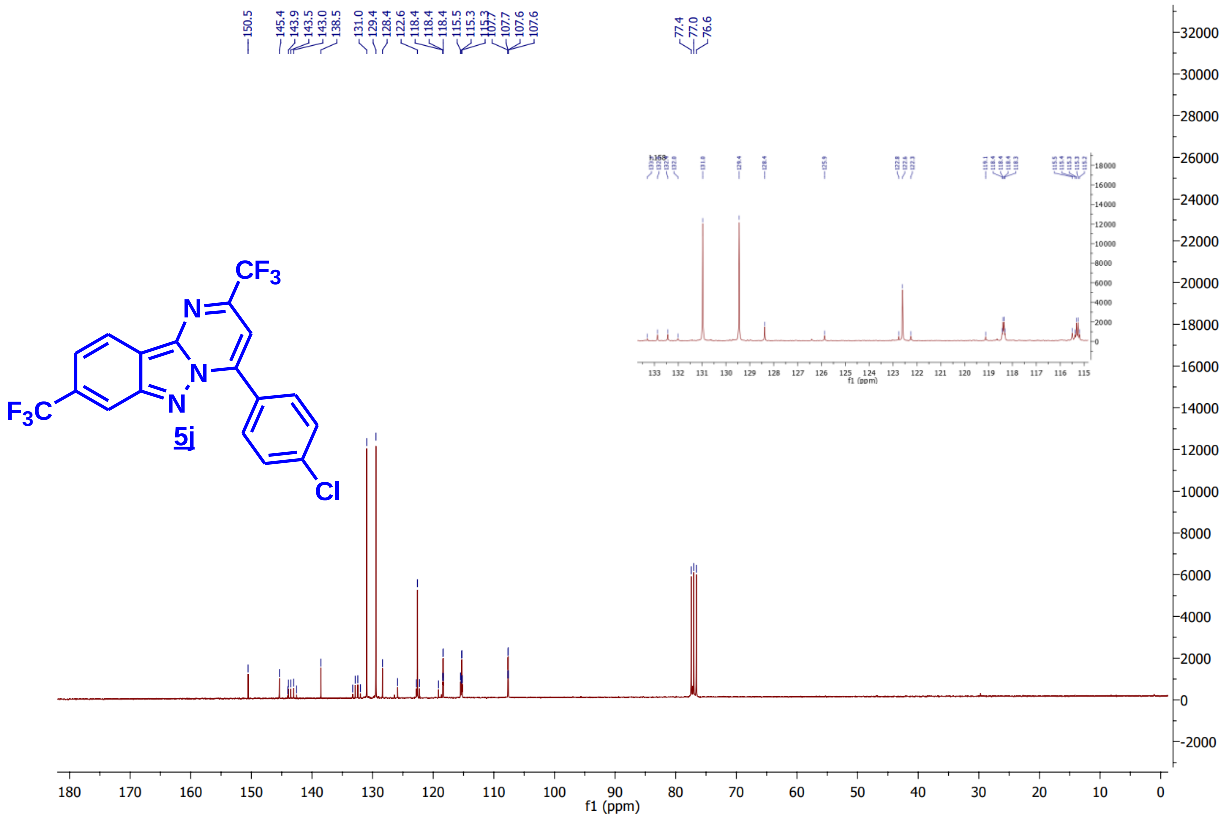

4-(4-Fluoro-phenyl)-2-trifluoromethyl-pyrimido[1,2-b]indazole (5k)

^{1}H NMR (300 MHz, $CDCl_3$)

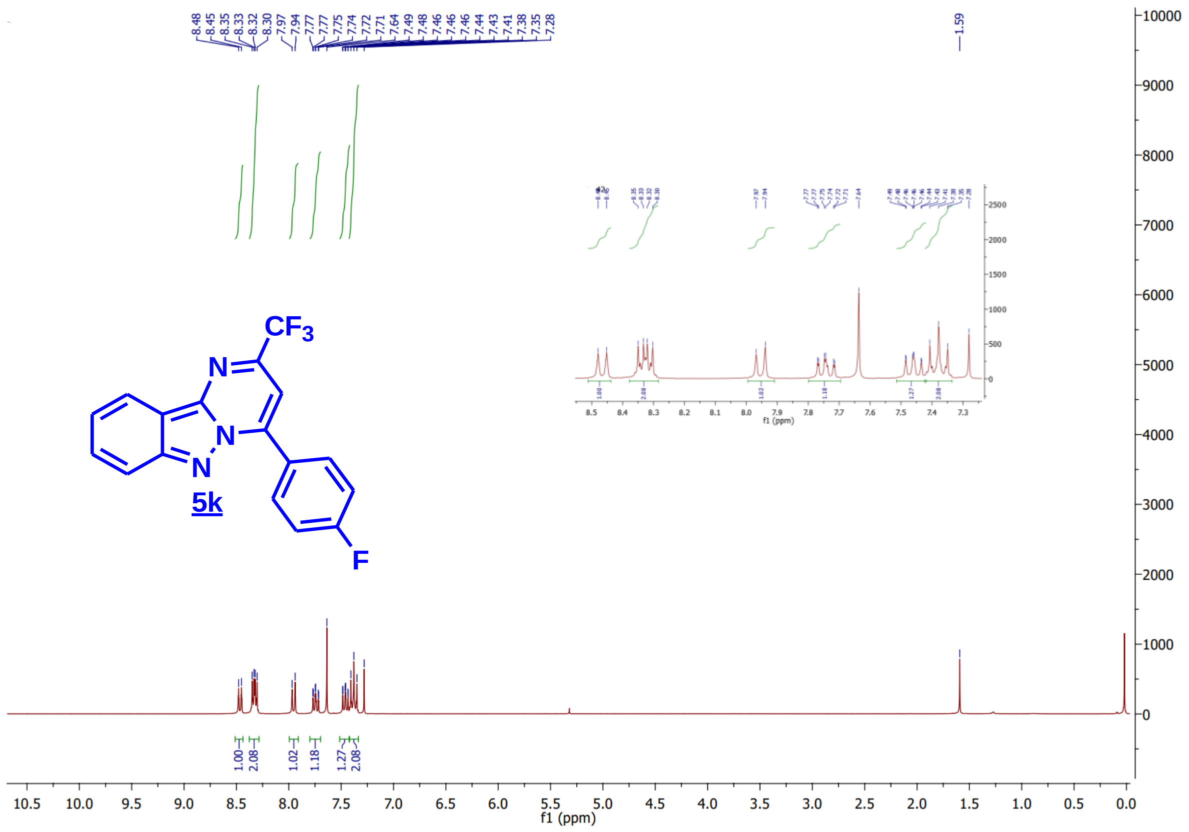

^{19}F NMR (282 MHz,$CDCl_3$)

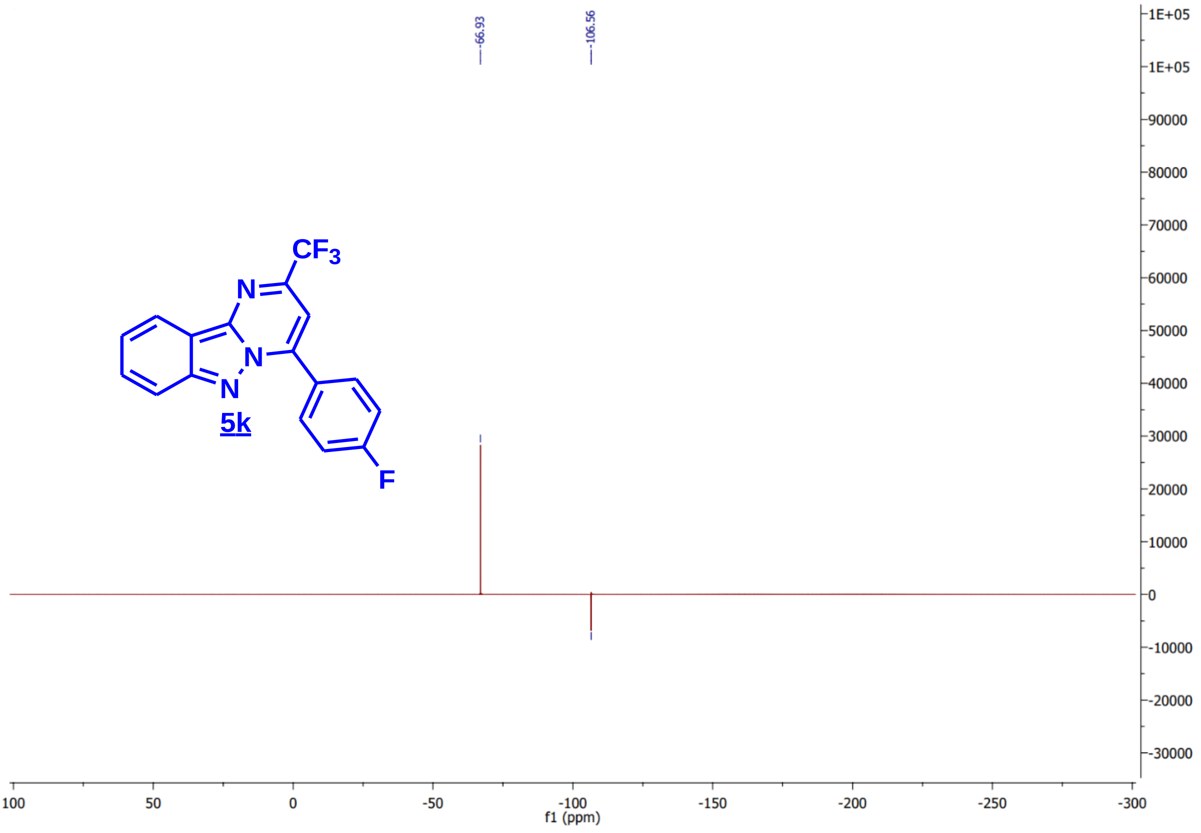

^{13}C NMR (75 MHz, $CDCl_3$)

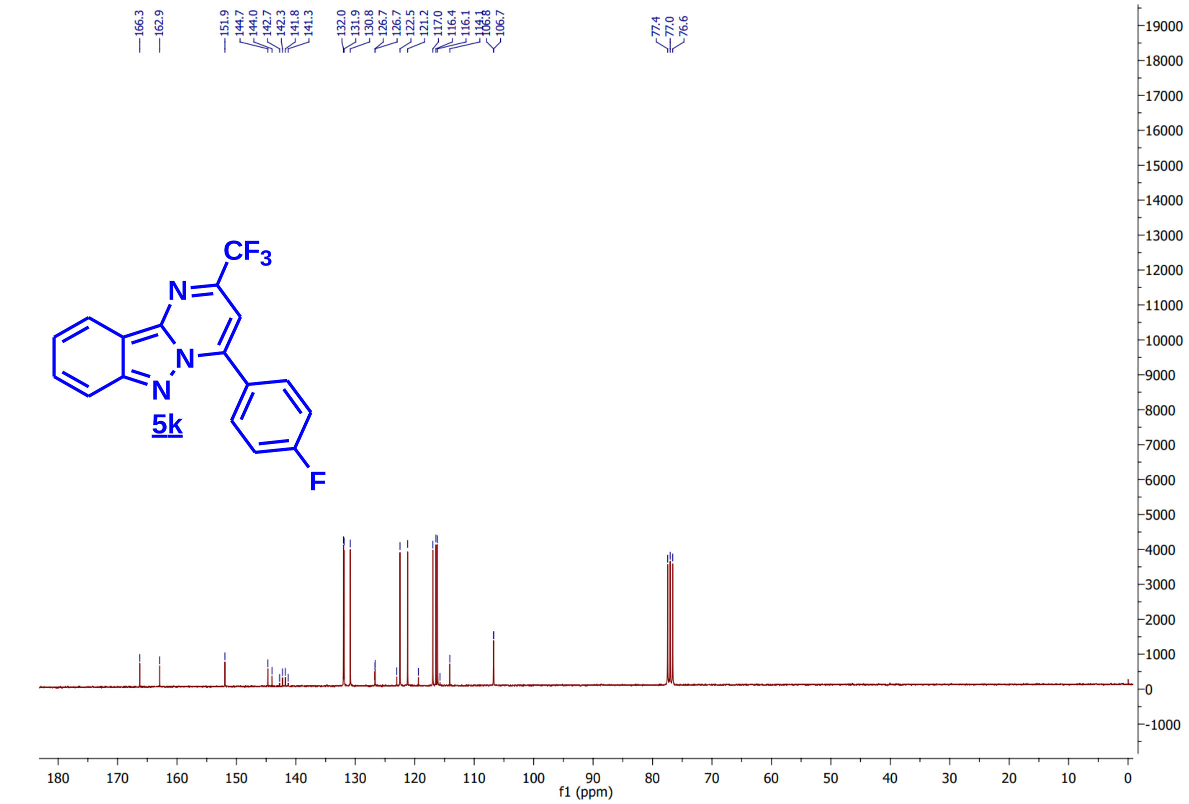

2-Trifluoromethyl-4-(4-trifluoromethyl-phenyl)-pyrimido[1,2-b]indazole (5l)

^{1}H NMR (300 MHz, $CDCl_3$)

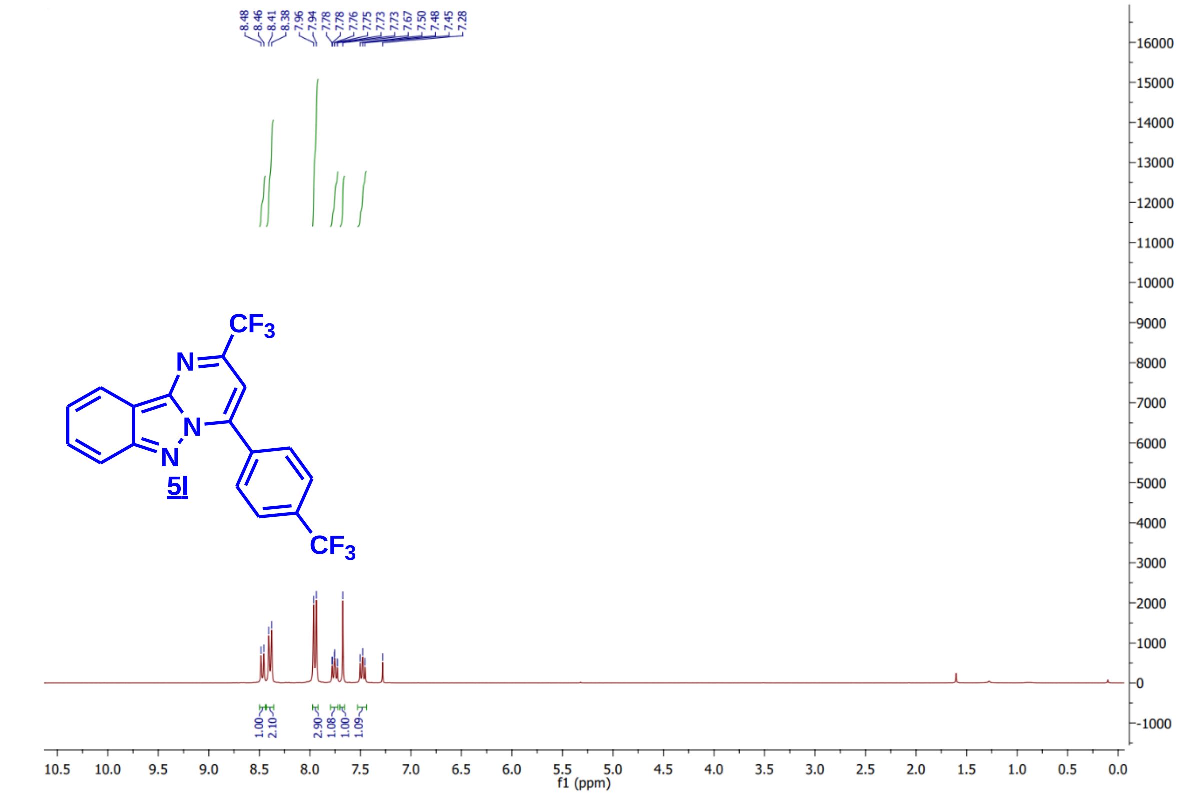

^{19}F NMR (282 MHz,CDCl$_3$)

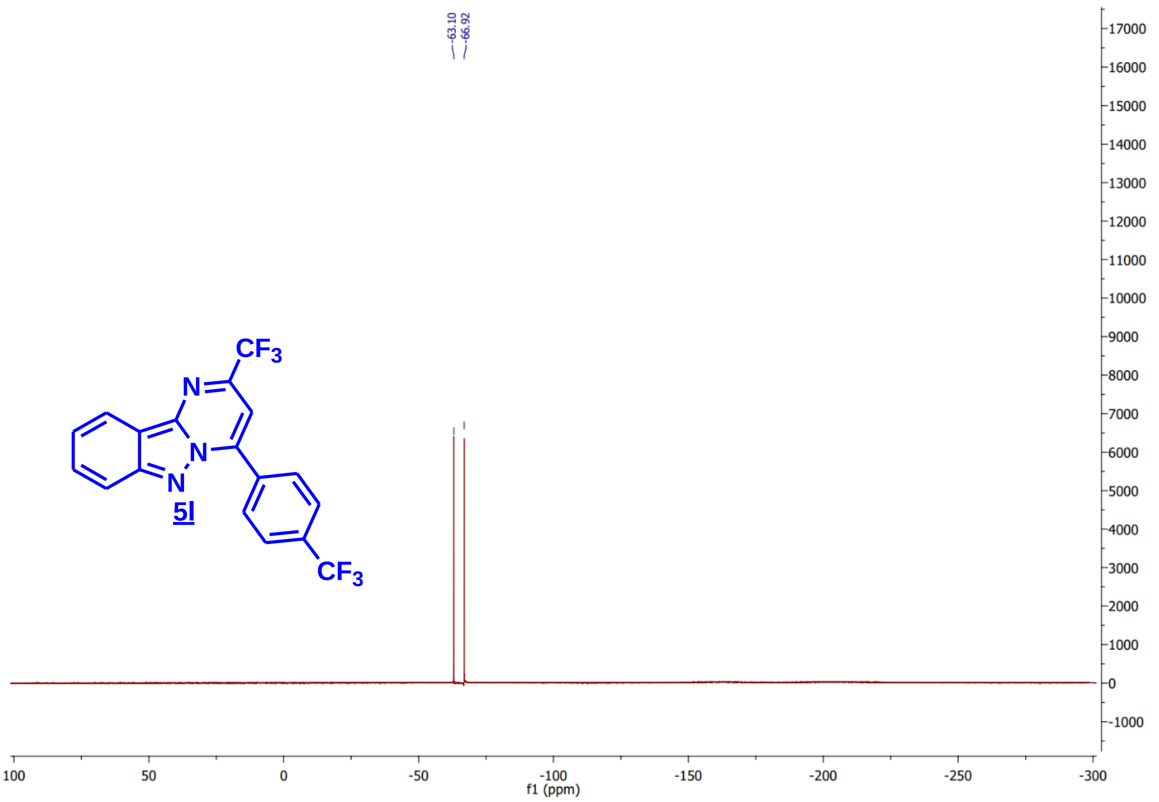

^{13}C NMR (75 MHz, CDCl$_3$)

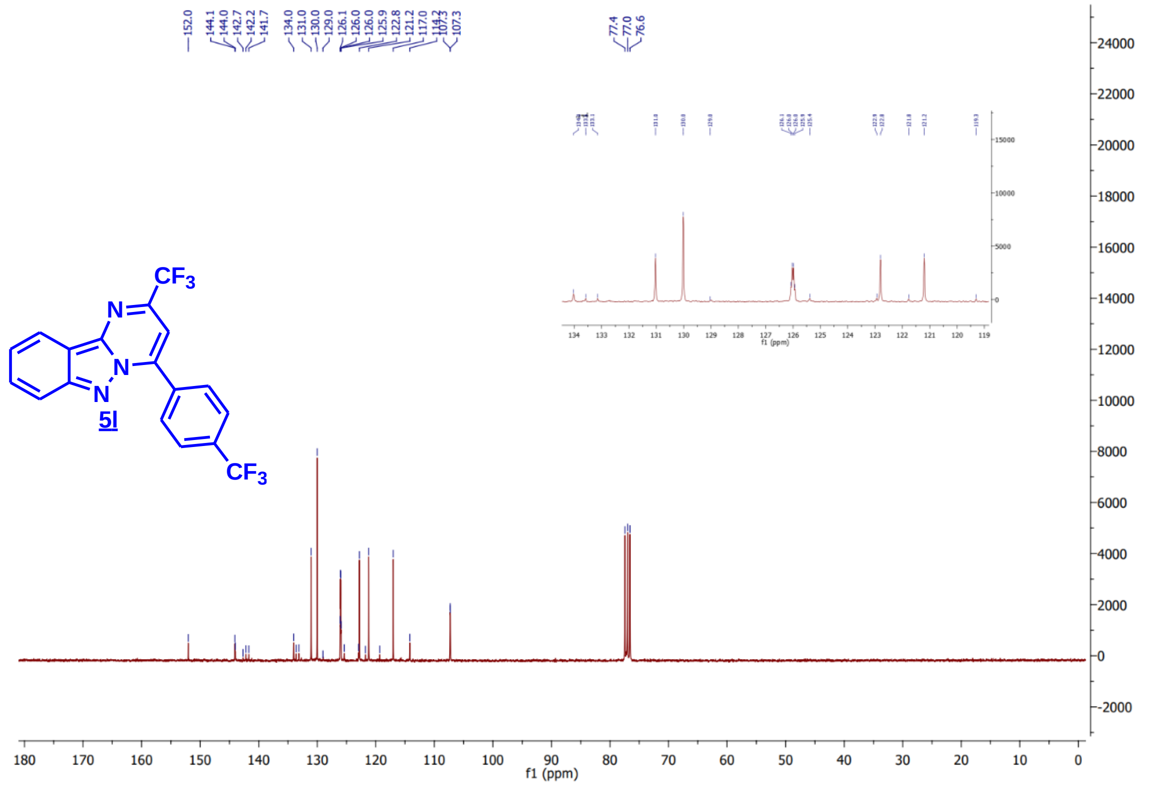

4-(4-Trifluoromethoxy-phenyl)-2-trifluoromethyl-pyrimido[1,2-b]indazole (5m)

^{1}H NMR (300 MHz, $CDCl_3$)

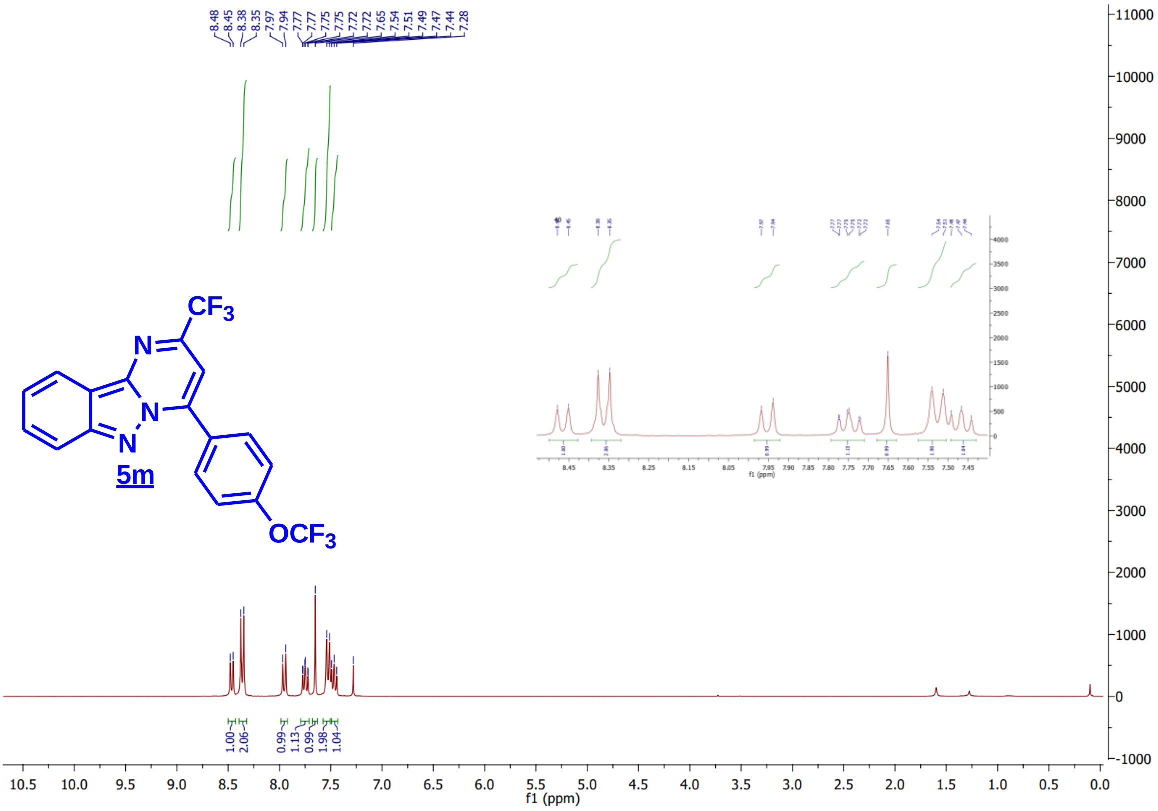

^{19}F NMR (282 MHz, $CDCl_3$)

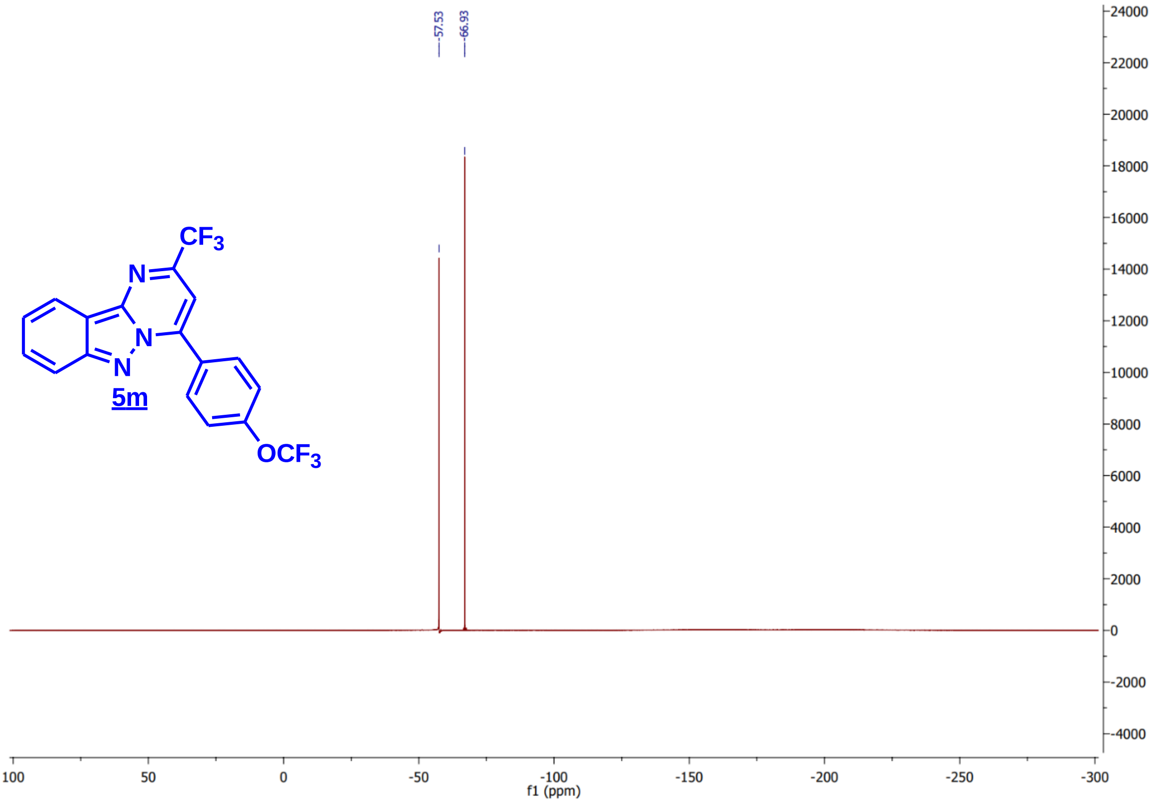

^{13}C NMR (75 MHz, $CDCl_3$)

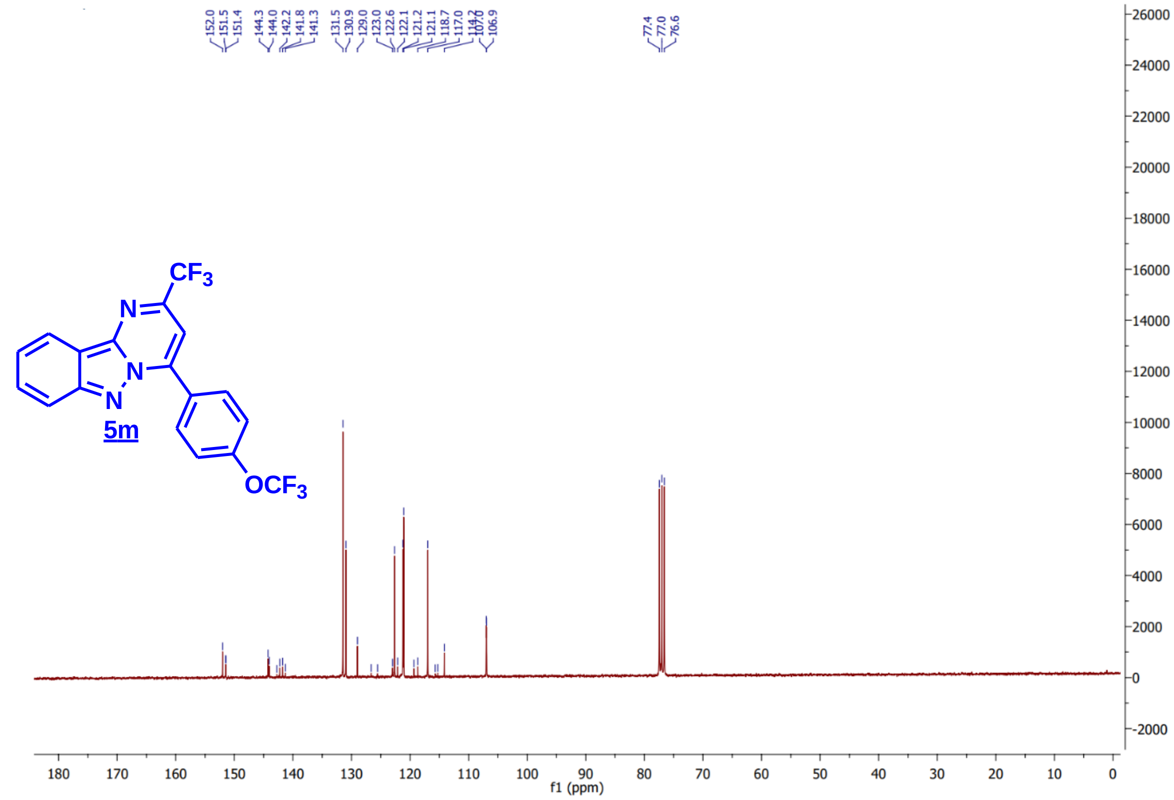

ethyl4-[9-(trifluoromethyl)pyrazino[2',3':3,4]pyrazolo[1,5-*a*]pyrimidin-7-yl]benzoate (5n)

^{1}H NMR (300 MHz, $CDCl_3$)

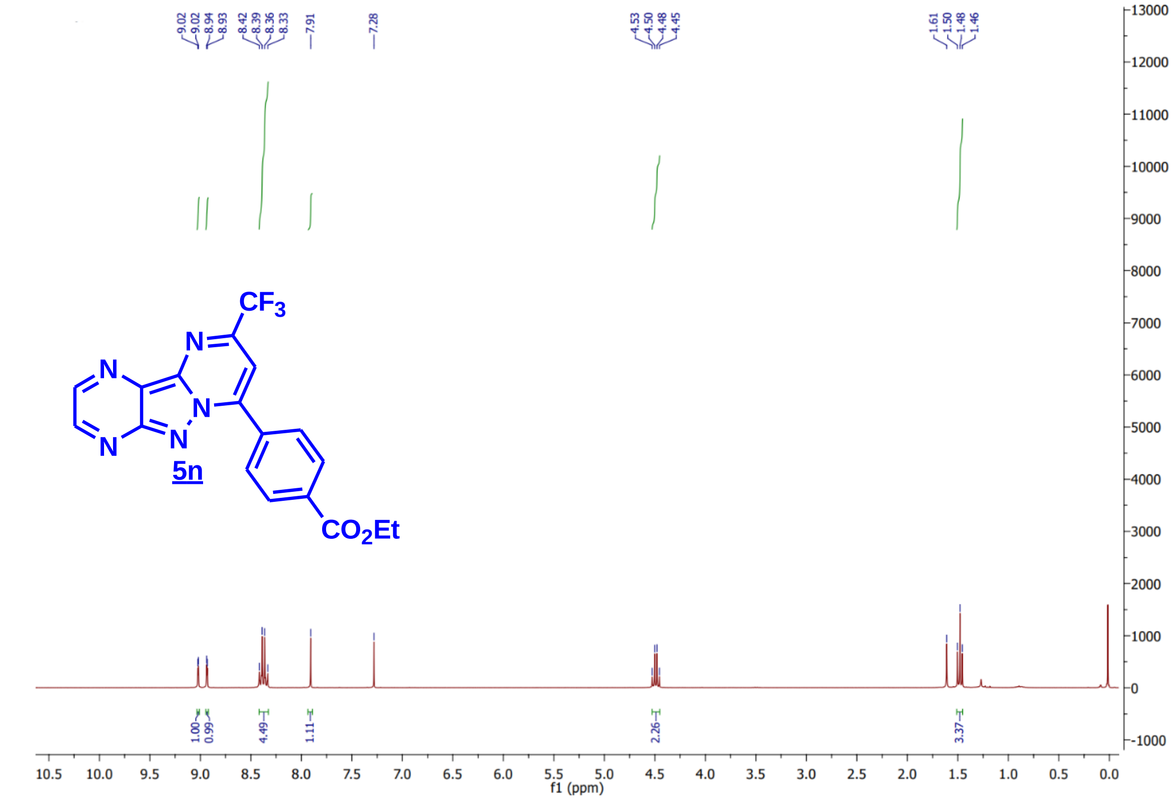

^{19}F NMR (282 MHz,CDCl3)

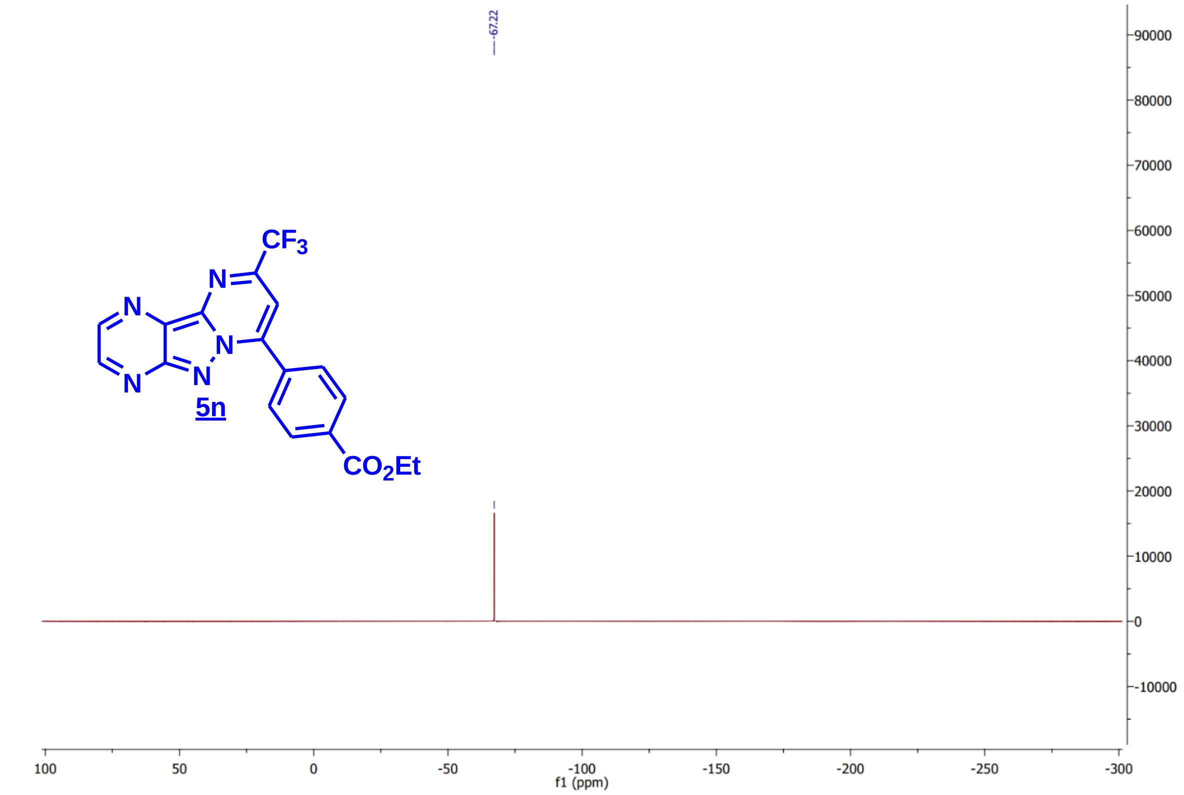

^{13}C NMR (75 MHz, CDCl3)

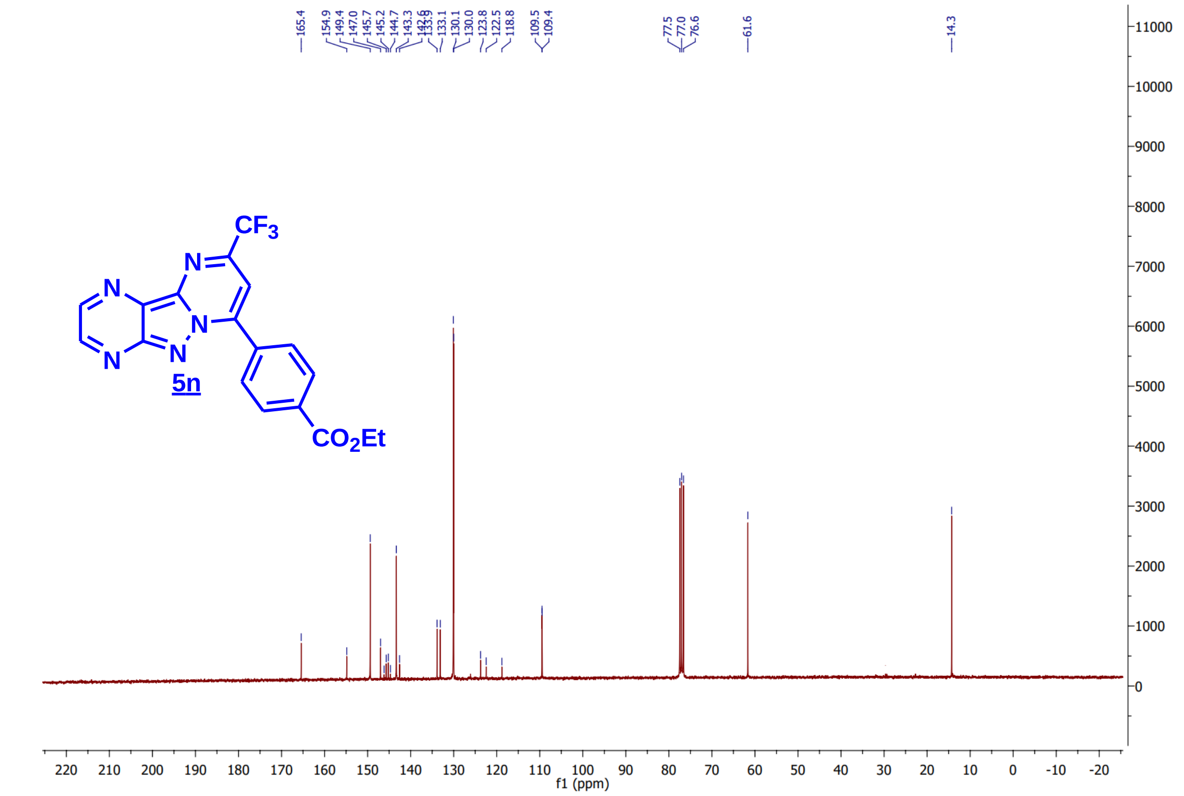

4-[2-(trifluoromethyl)pyrido[2',3':3,4]pyrazolo[1,5-*a*]pyrimidin-4-yl]benzaldehyde (5o)

^{1}H NMR (300 MHz, $CDCl_3$

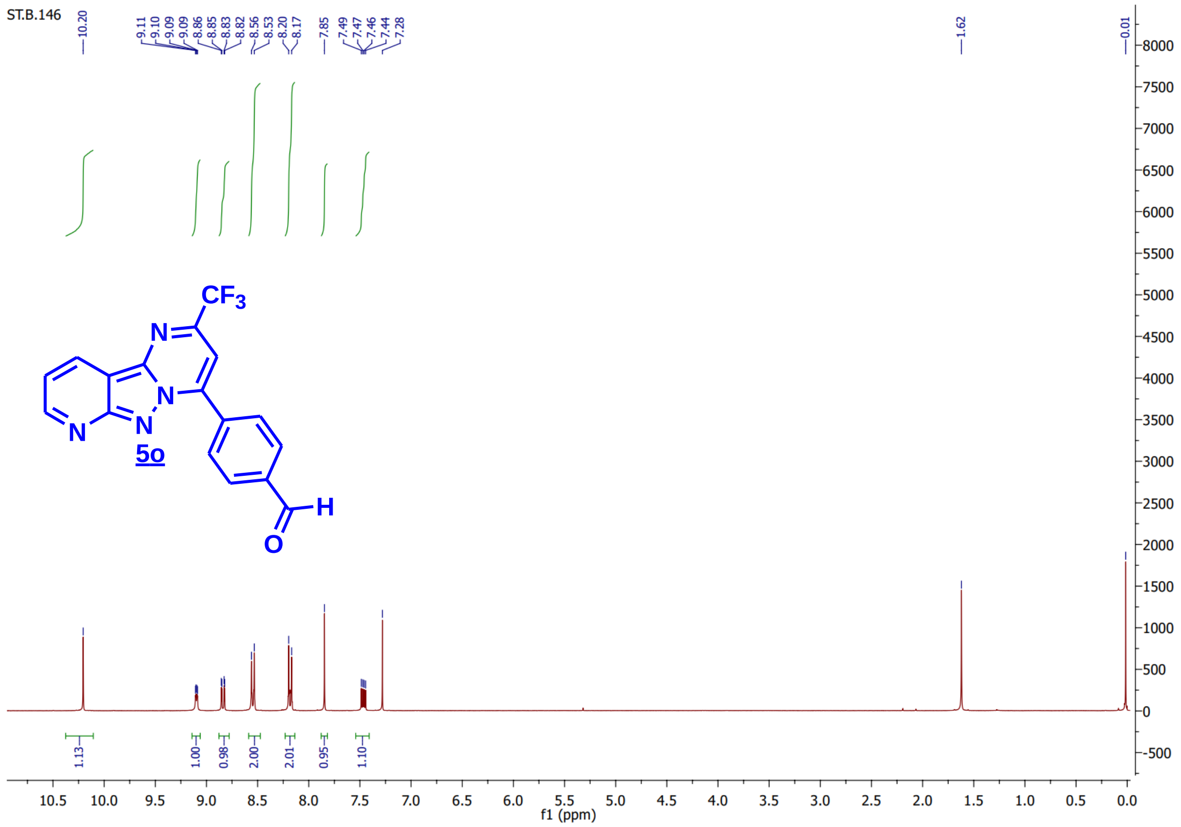

^{19}F NMR (282 MHz,$CDCl_3$)

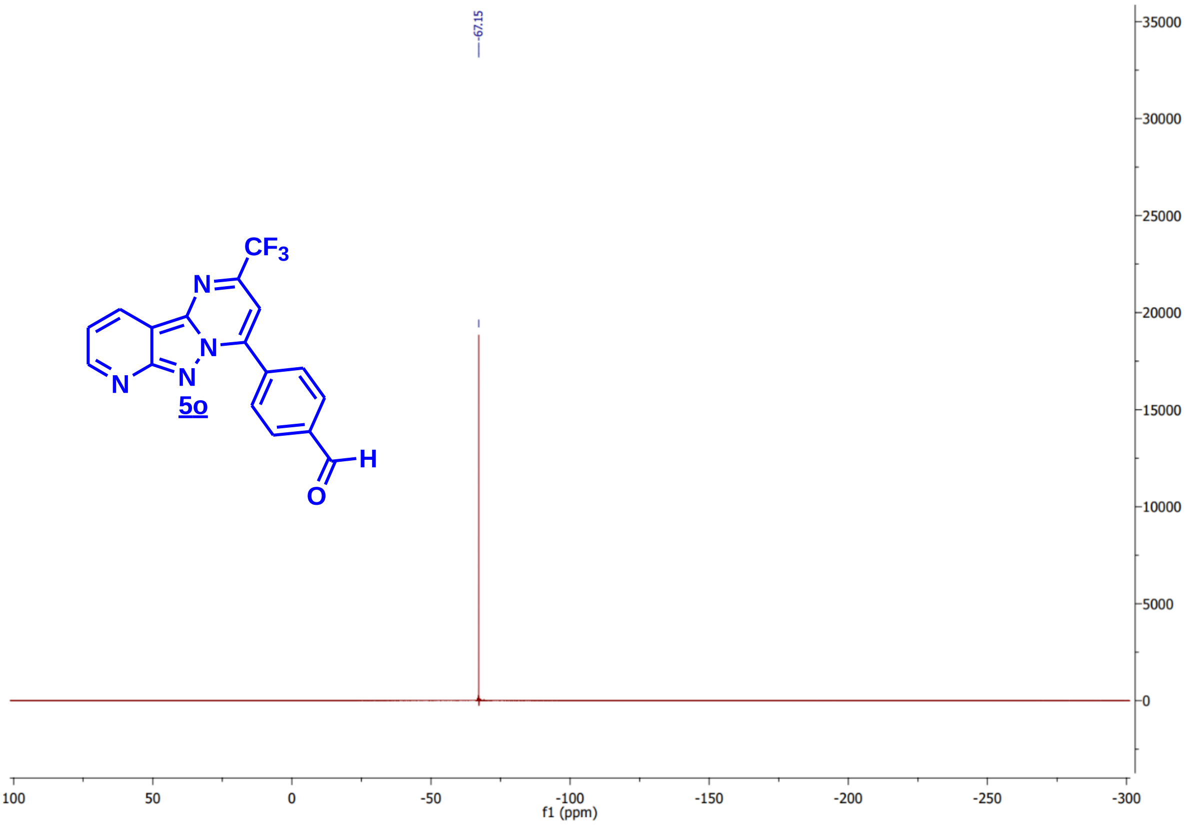

^{13}C NMR (75 MHz, $CDCl_3$)

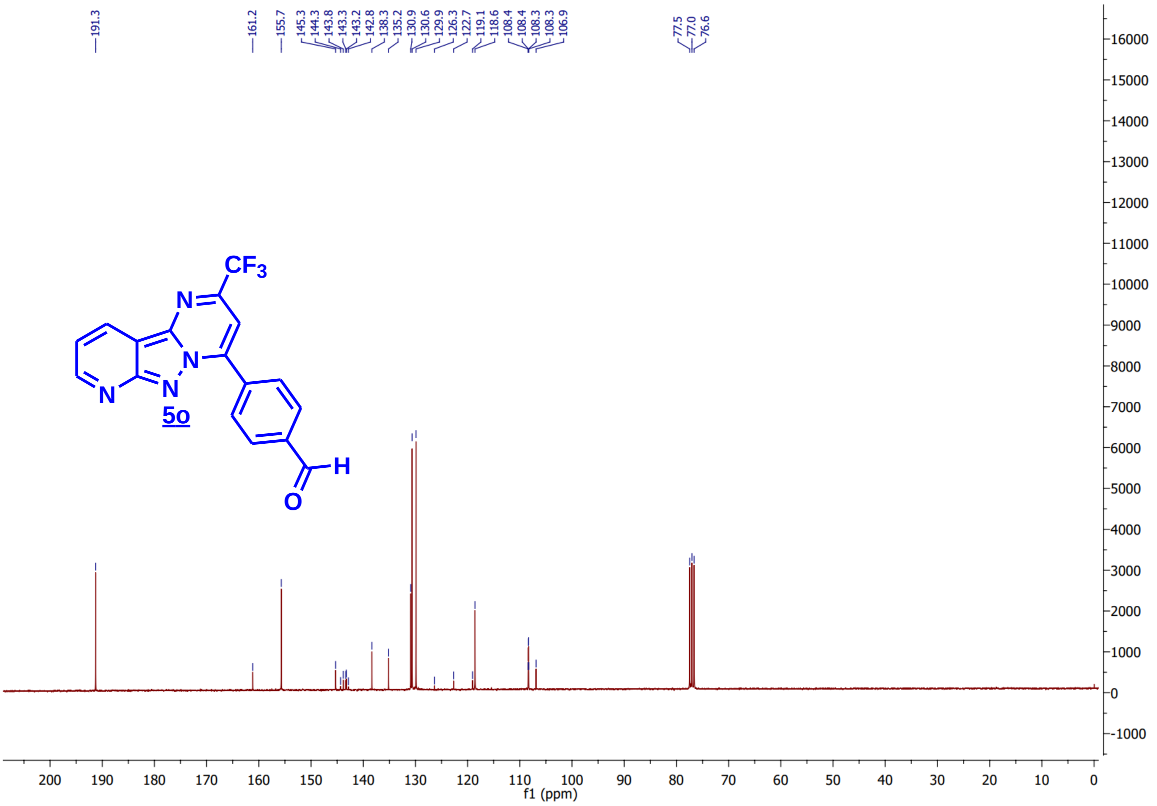

4-[9-(trifluoromethyl)pyrido[3',2':3,4]pyrazolo[1,5-*a*]pyrimidin-7-yl]benzonitrile (5p)

^{1}H NMR (300 MHz, $CDCl_3$)

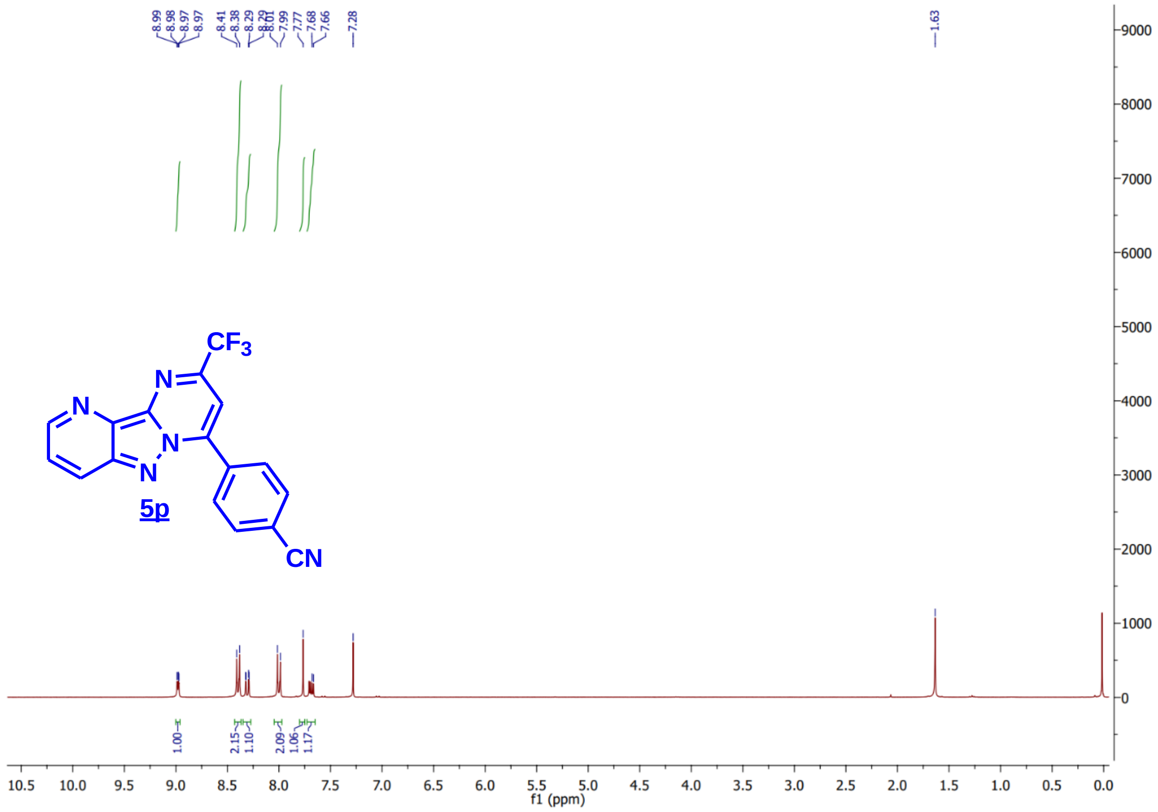

^{19}F NMR (282 MHz,$CDCl_3$)

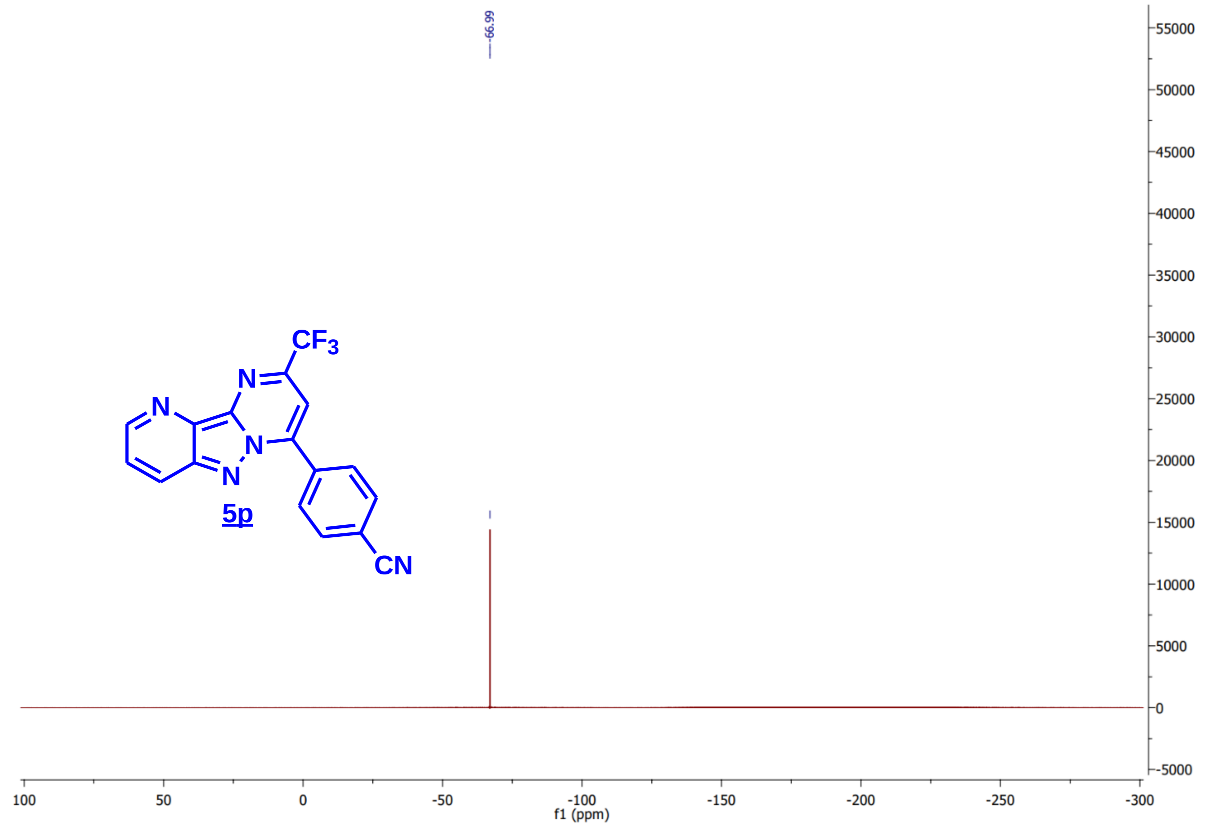

^{13}C NMR (75 MHz, $CDCl_3$)

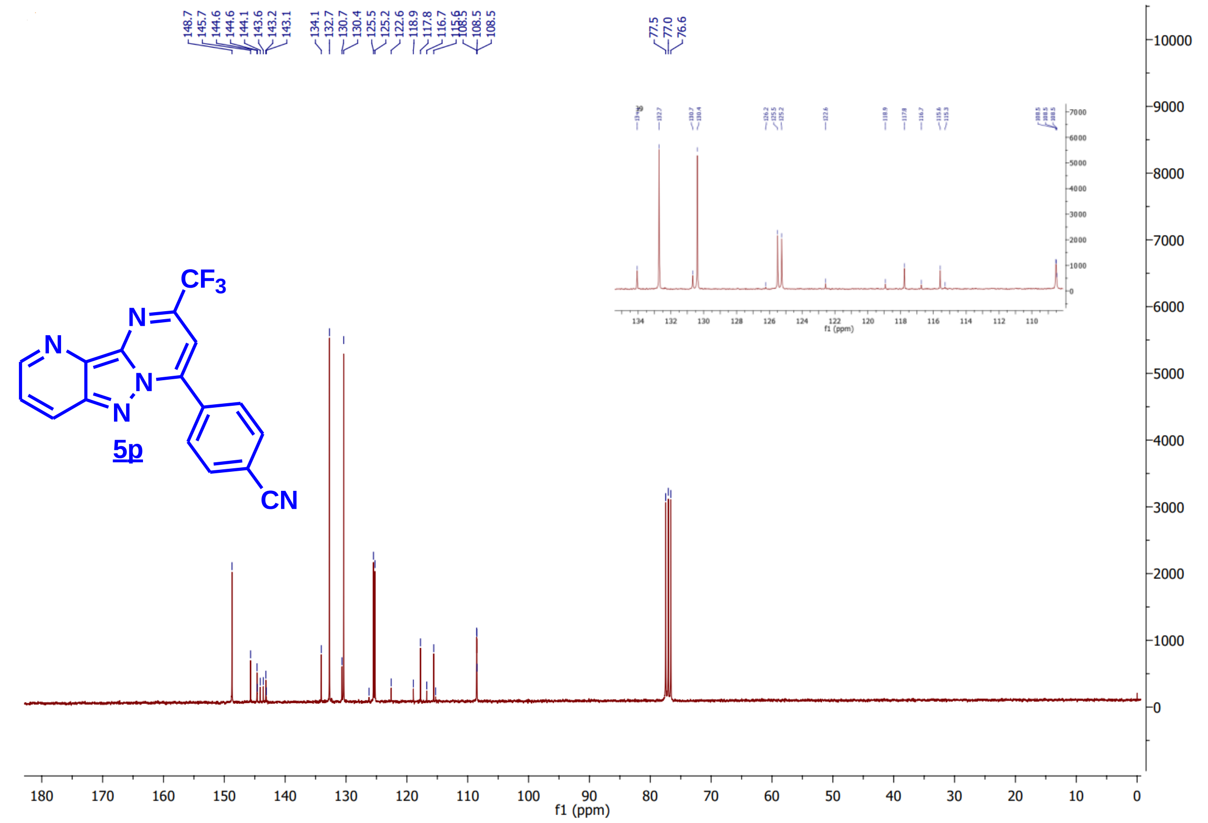

7-(4-nitrophenyl)-9-(trifluoromethyl)pyrido[3',2':3,4]pyrazolo[1,5-*a*]pyrimidine (5q)

^{1}H NMR (300 MHz, $CDCl_3$)

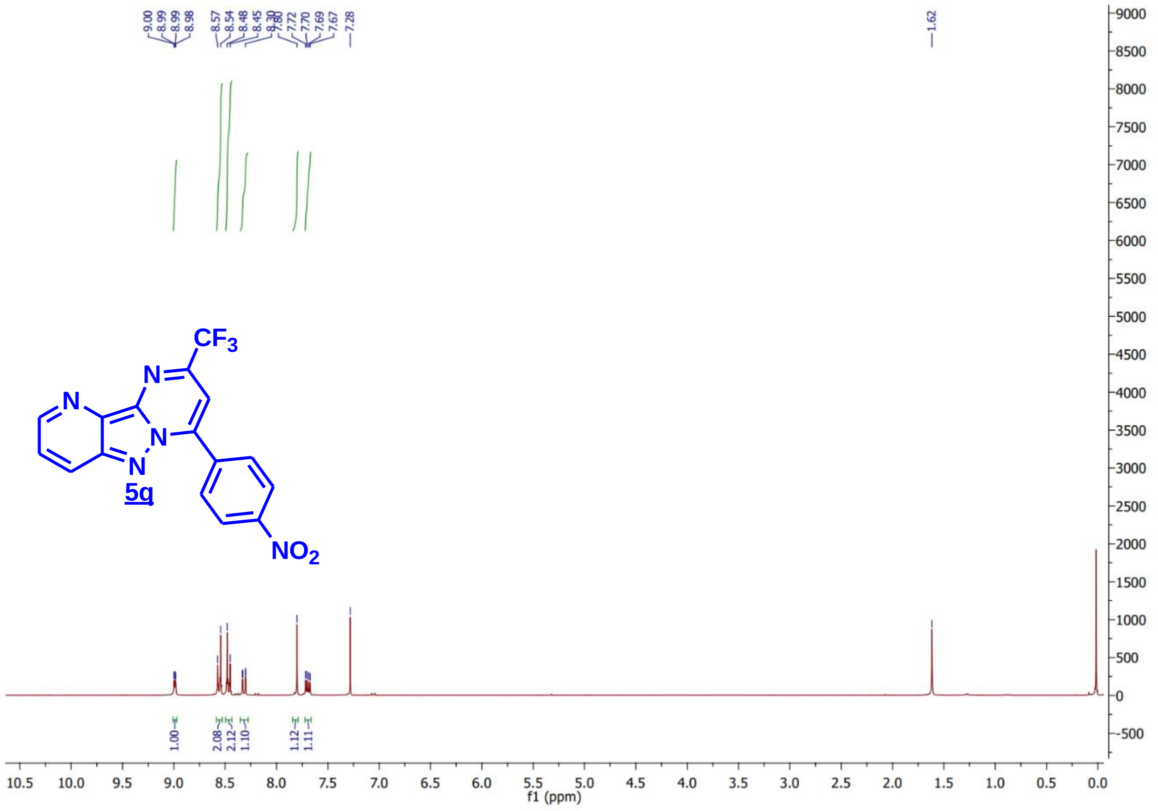

^{19}F NMR (282 MHz, $CDCl_3$)

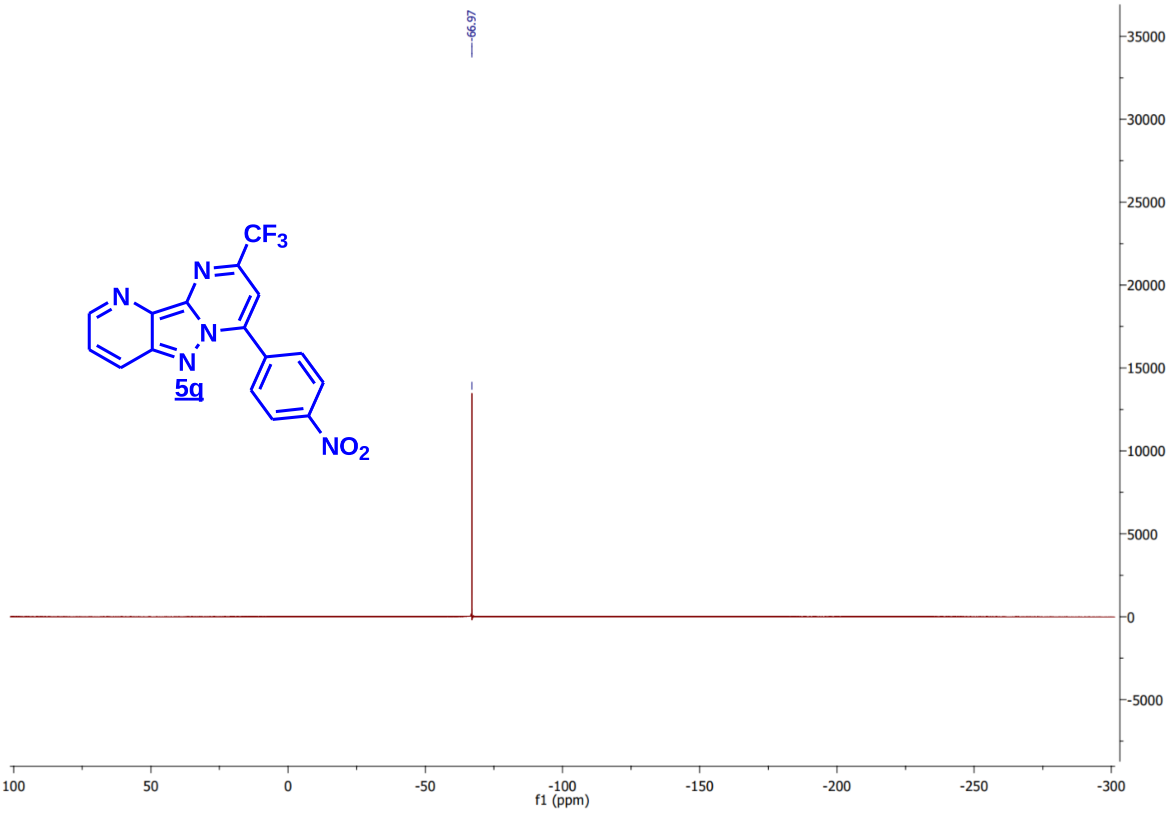

^{13}C NMR (75 MHz, DMSO-d$_6$)

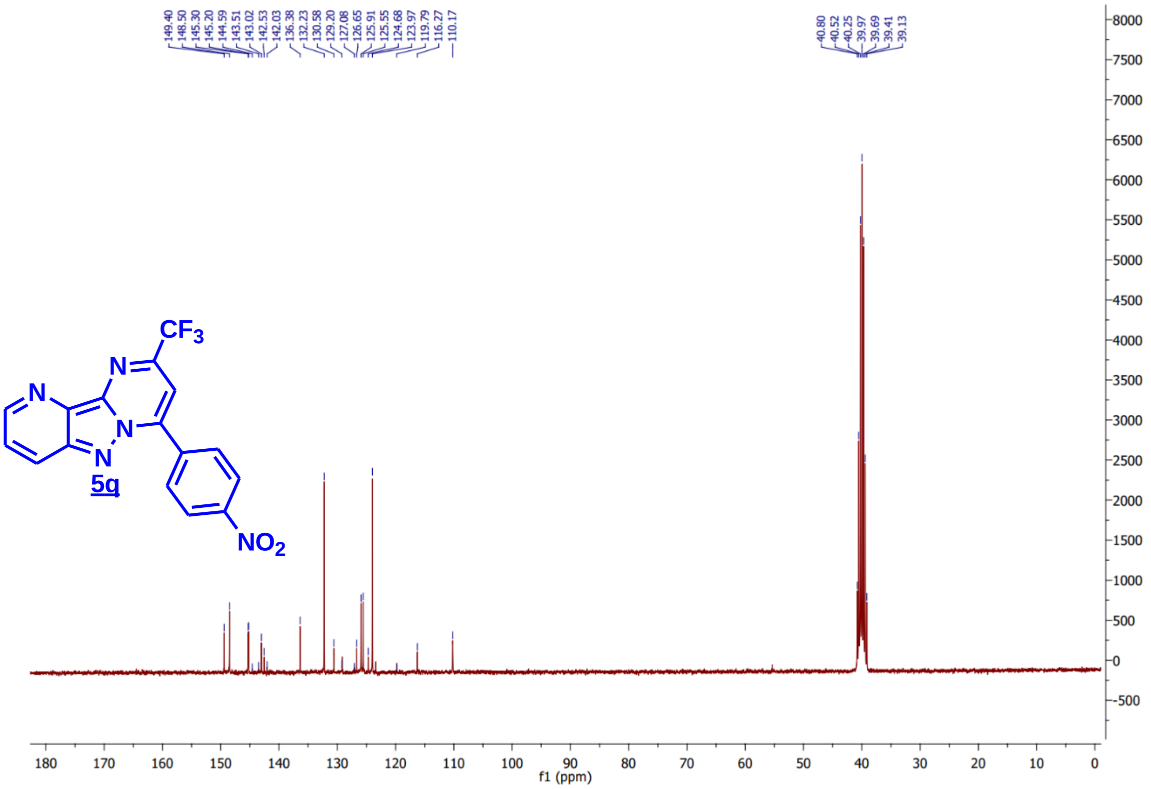

N-propyl-9-(trifluoromethyl)pyrido[3',2':3,4]pyrazolo[1,5-*a*]pyrimidin-7-amine (6a)

^{1}H NMR (300 MHz, $CDCl_3$)

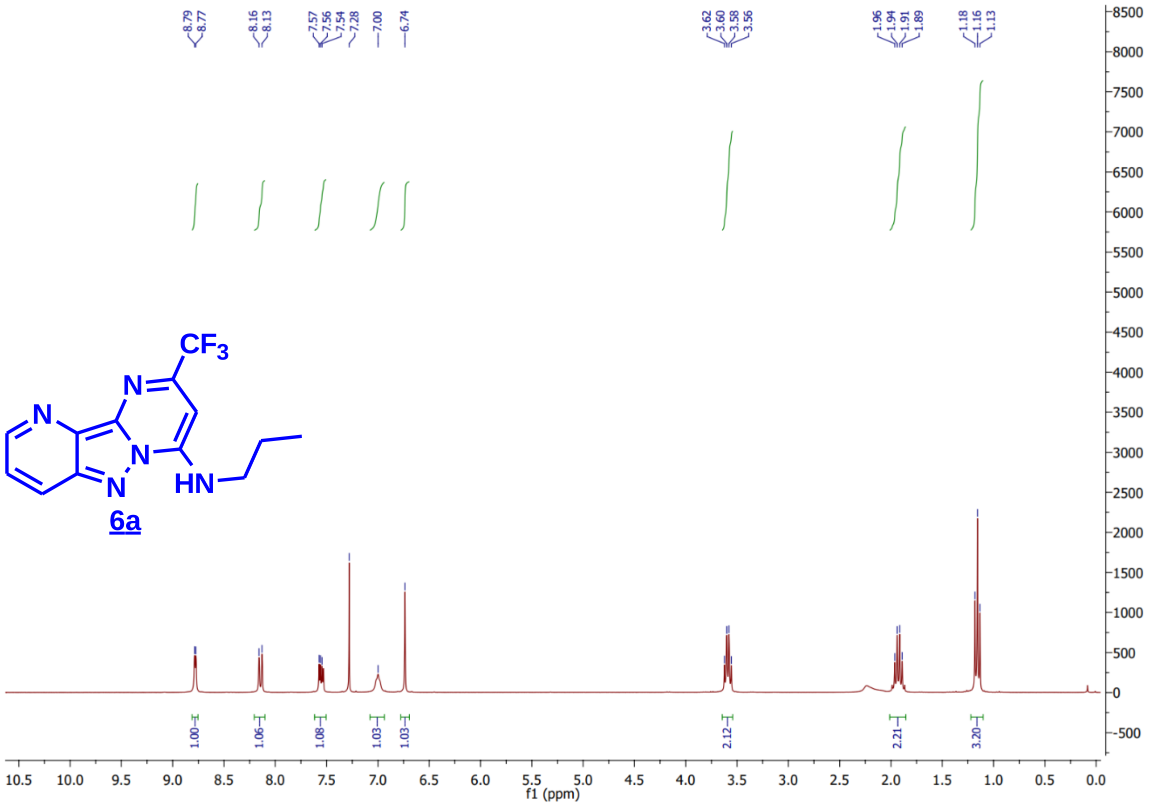

^{19}F NMR (282 MHz,$CDCl_3$)

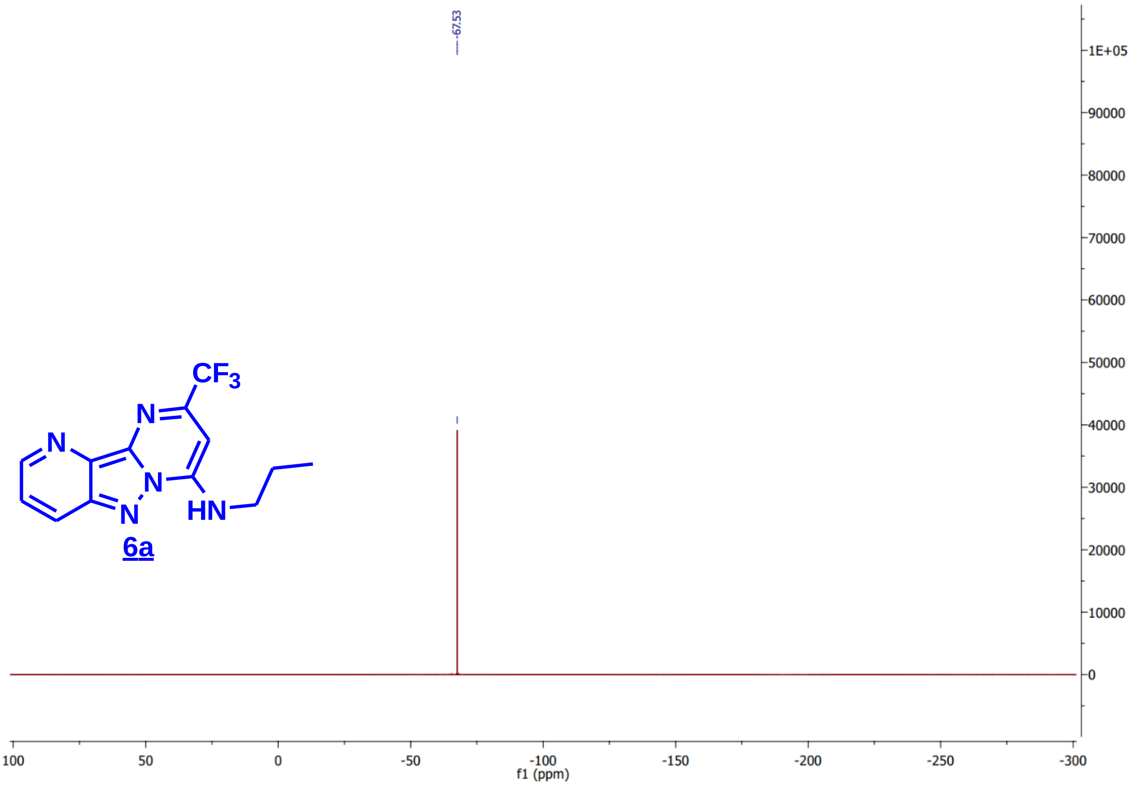

^{13}C NMR (75 MHz, $CDCl_3$)

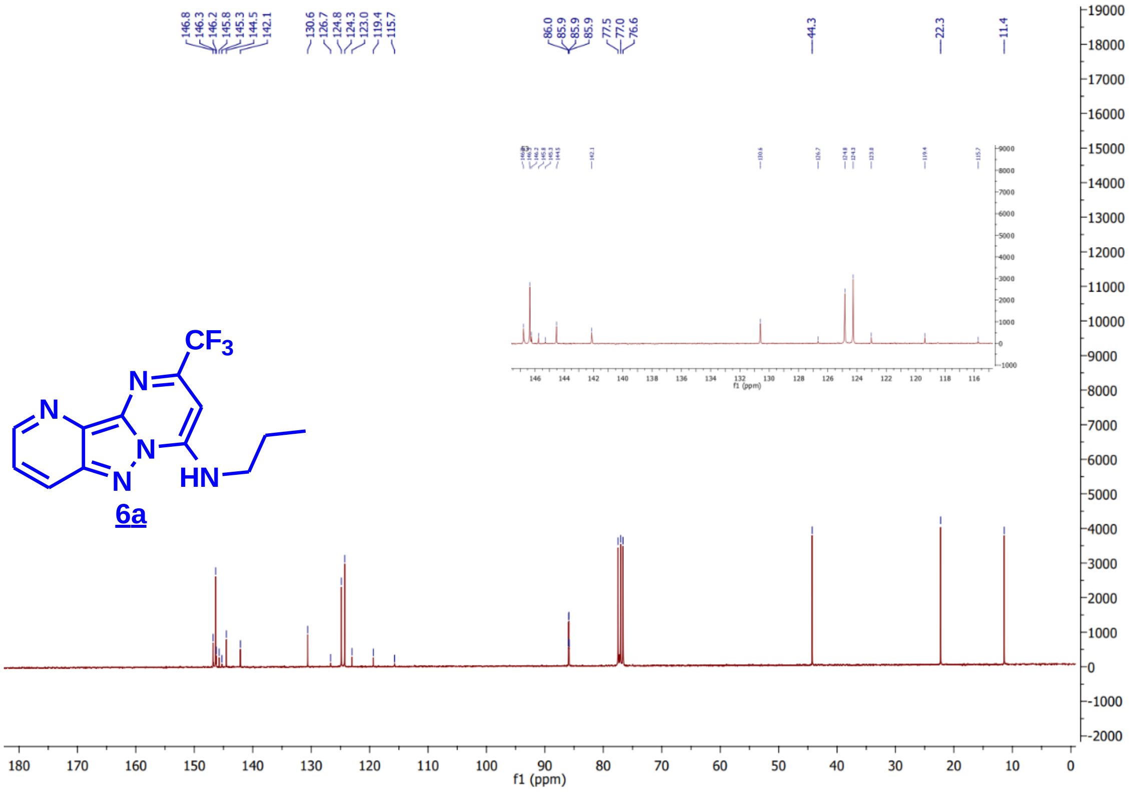

_N_-[(4-methoxyphenyl)methyl]-9-(trifluoromethyl)pyrido[3',2':3,4]pyrazolo[1,5-_a_]pyrimidin-7-amine (6b)

^{1}H NMR (300 MHz, $CDCl_3$)

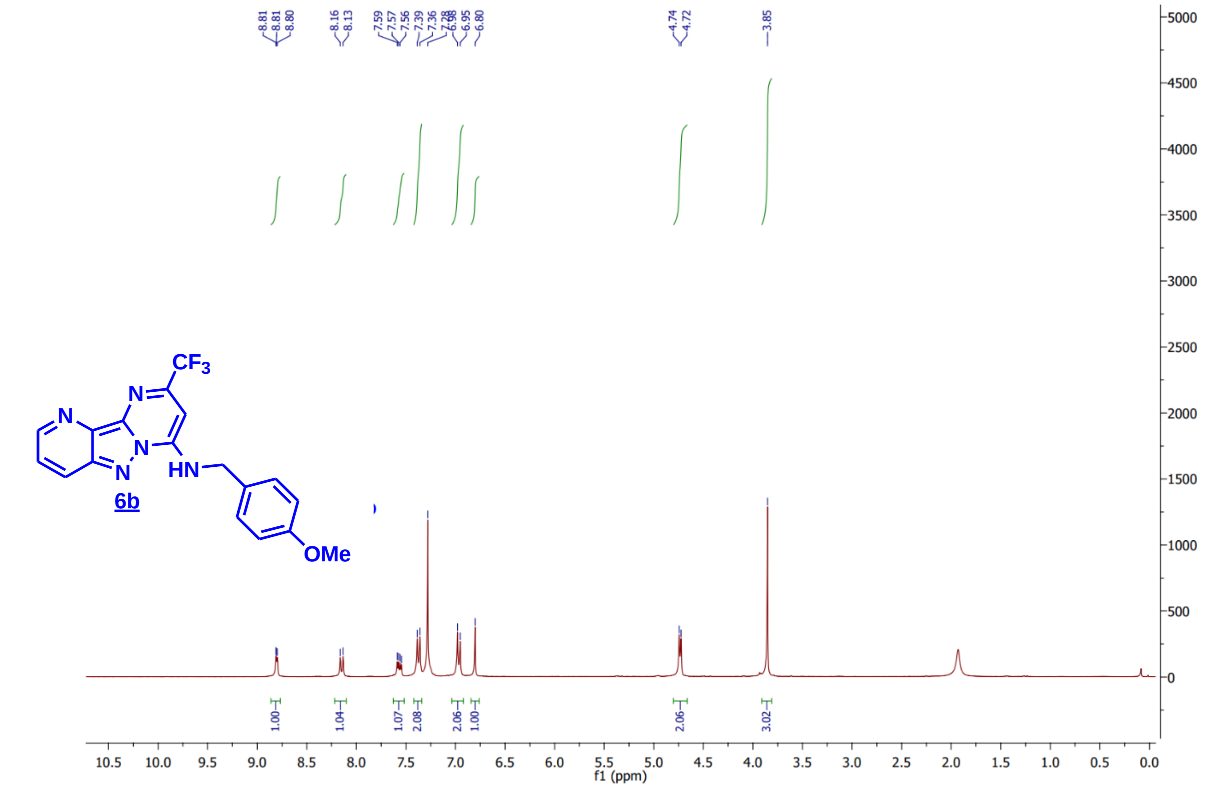

^{19}F NMR (282 MHz,$CDCl_3$)

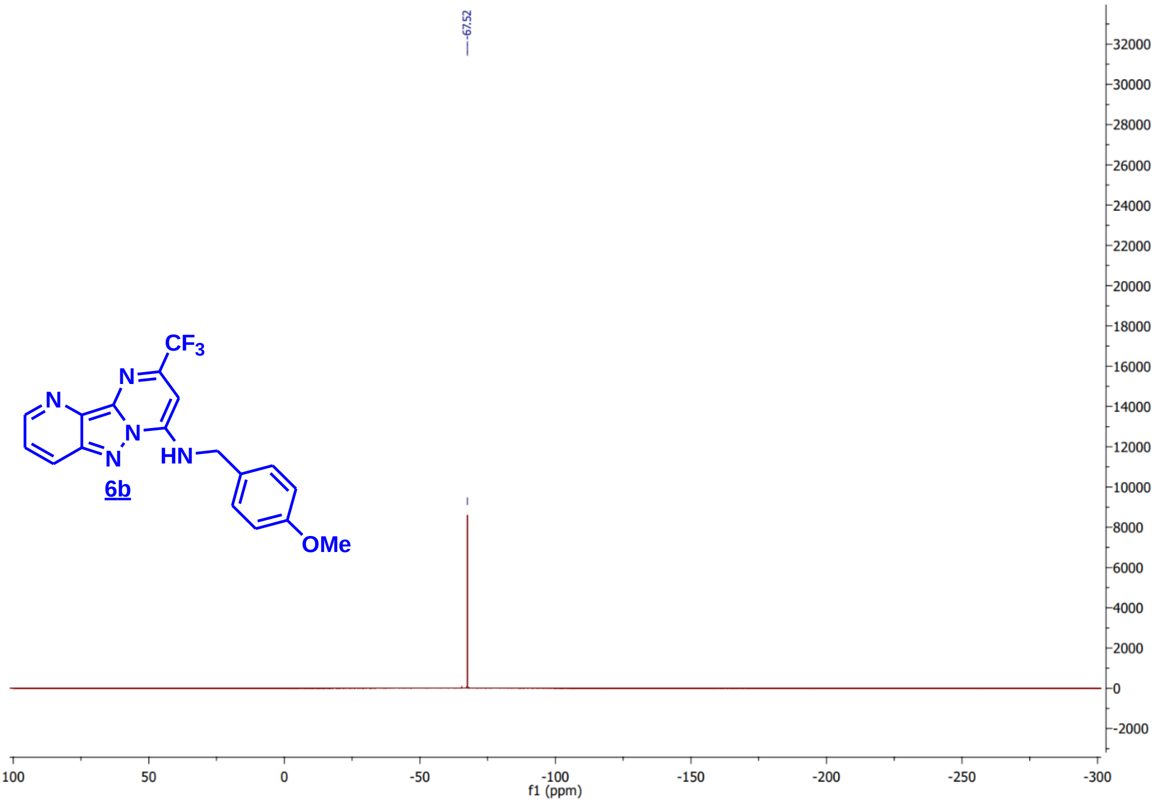

^{13}C NMR (75 MHz, $CDCl_3$)

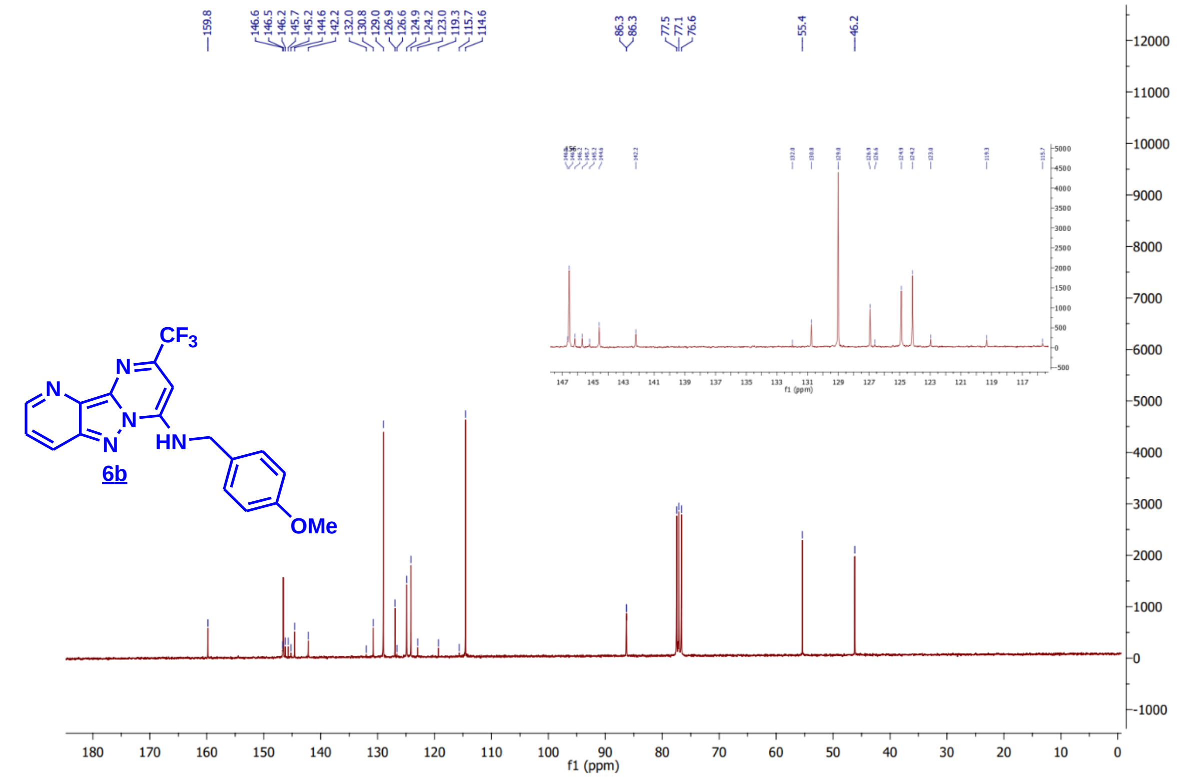

7-(morpholin-4-yl)-9-(trifluoromethyl)pyrido[3',2':3,4]pyrazolo[1,5-*a*]pyrimidine (6c)

^{1}H NMR (300 MHz, $CDCl_3$)

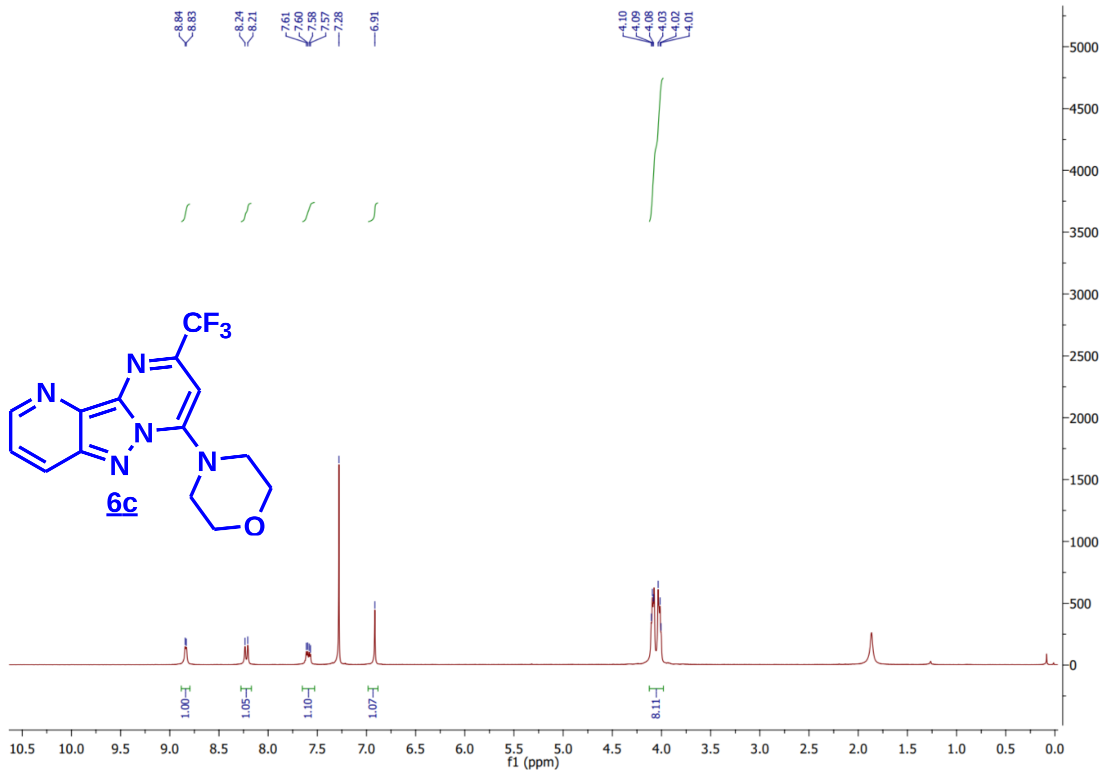

^{19}F NMR (282 MHz,CDCl3)

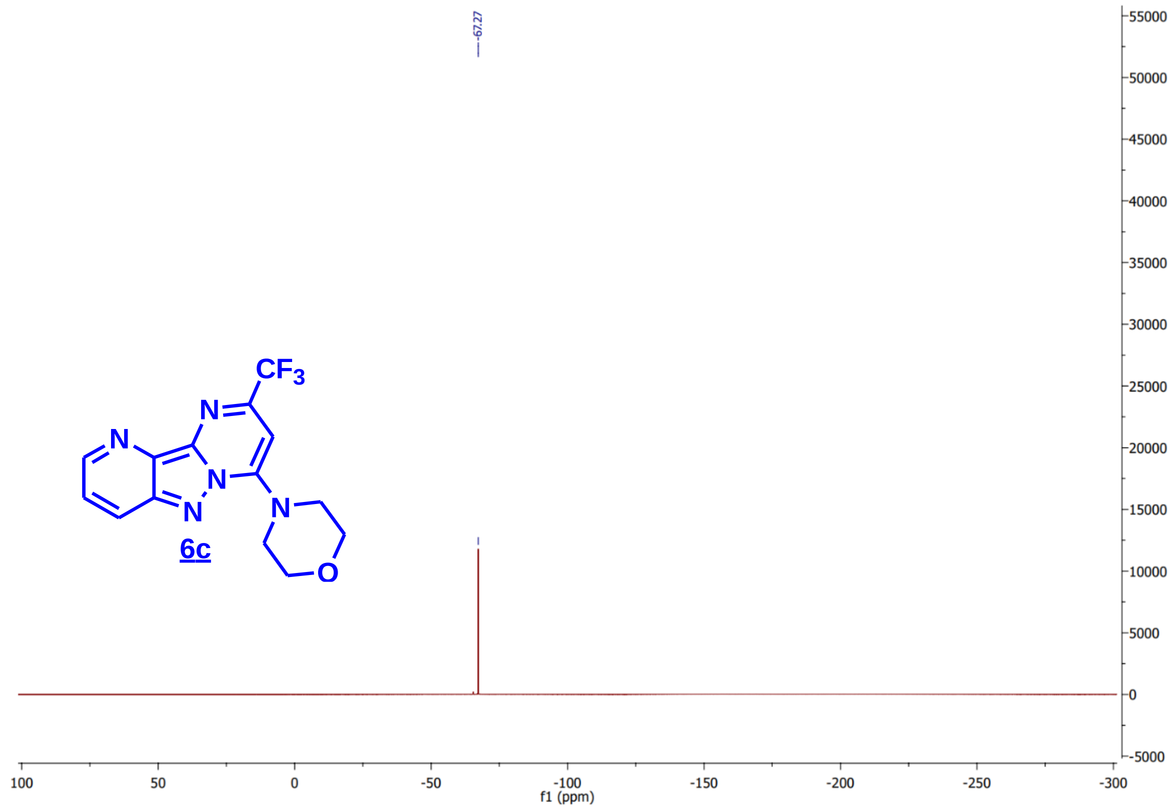

^{13}C NMR (75 MHz, CDCl3)

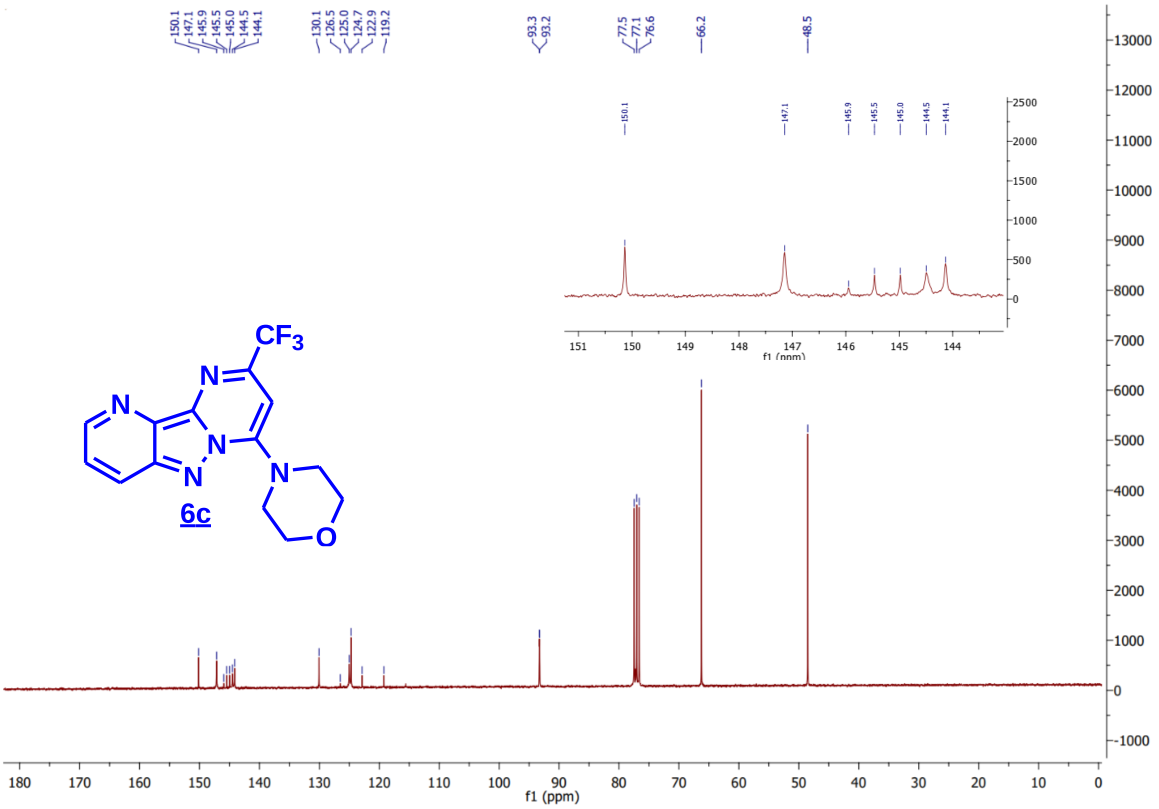

7-(pyrrolidin-1-yl)-9-(trifluoromethyl)pyrido[3',2':3,4]pyrazolo[1,5-*a*]pyrimidine (6d)

^{1}H NMR (300 MHz, CDCl$_3$)

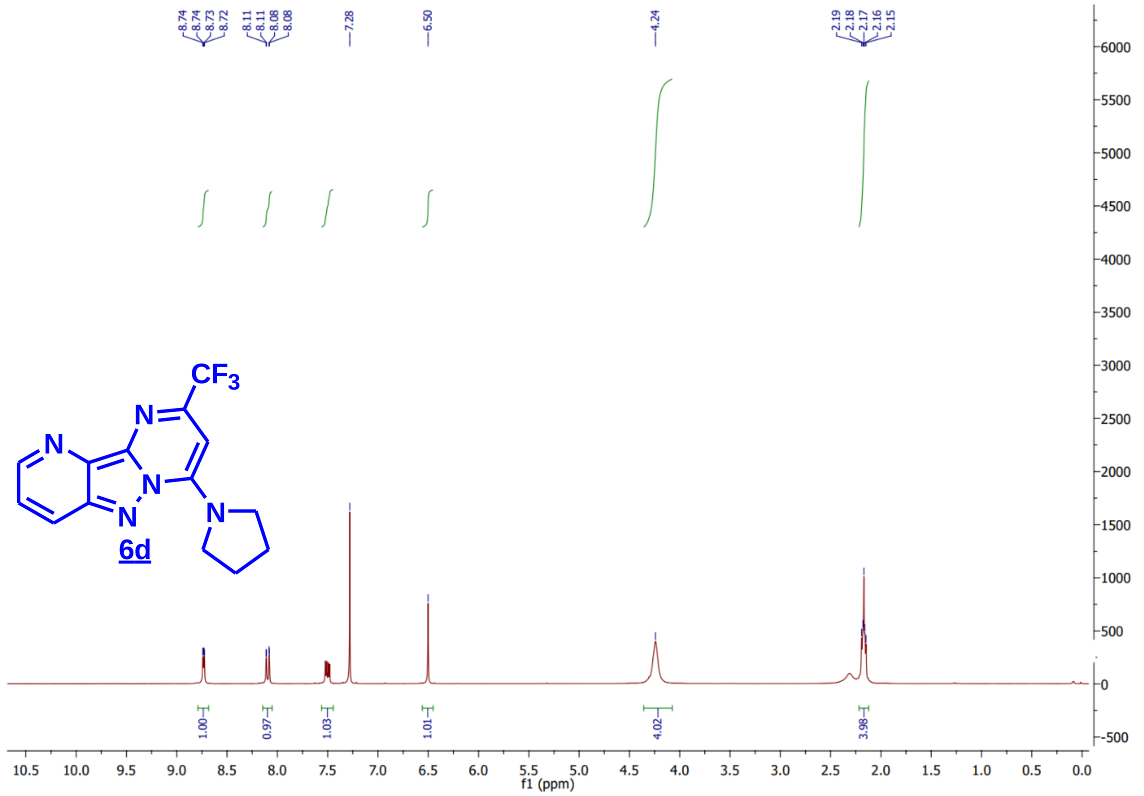

^{19}F NMR (282 MHz,CDCl$_3$)

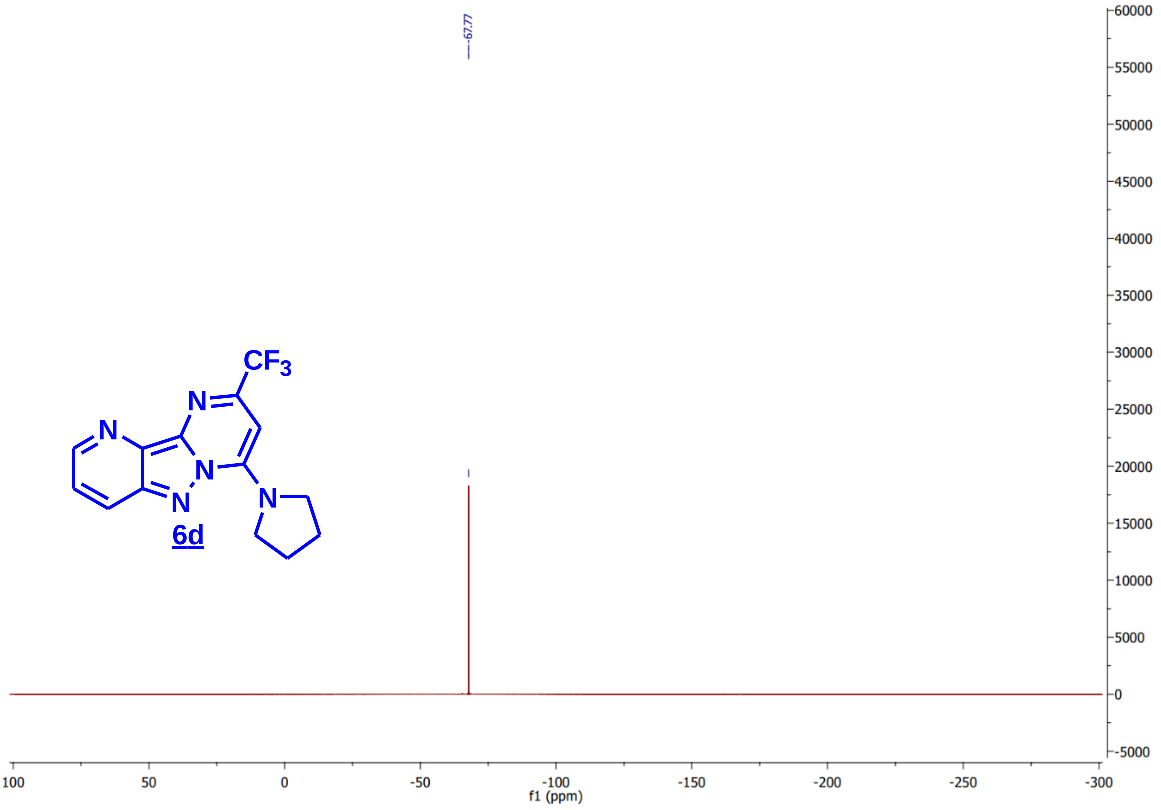

^{13}C NMR (75 MHz, $CDCl_3$)

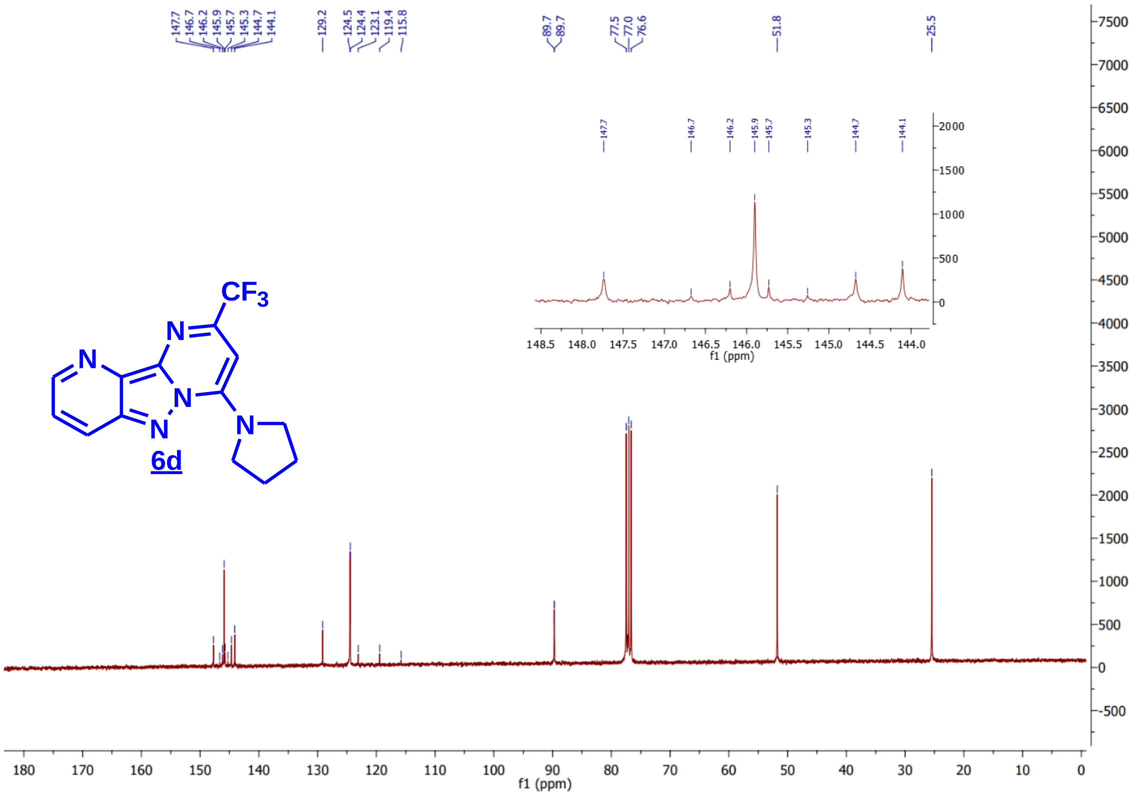

***N*-phenyl-9-(trifluoromethyl)pyrido[3',2':3,4]pyrazolo[1,5-*a*]pyrimidin-7-amine (6e)**

^{1}H NMR (300 MHz, $CDCl_3$)

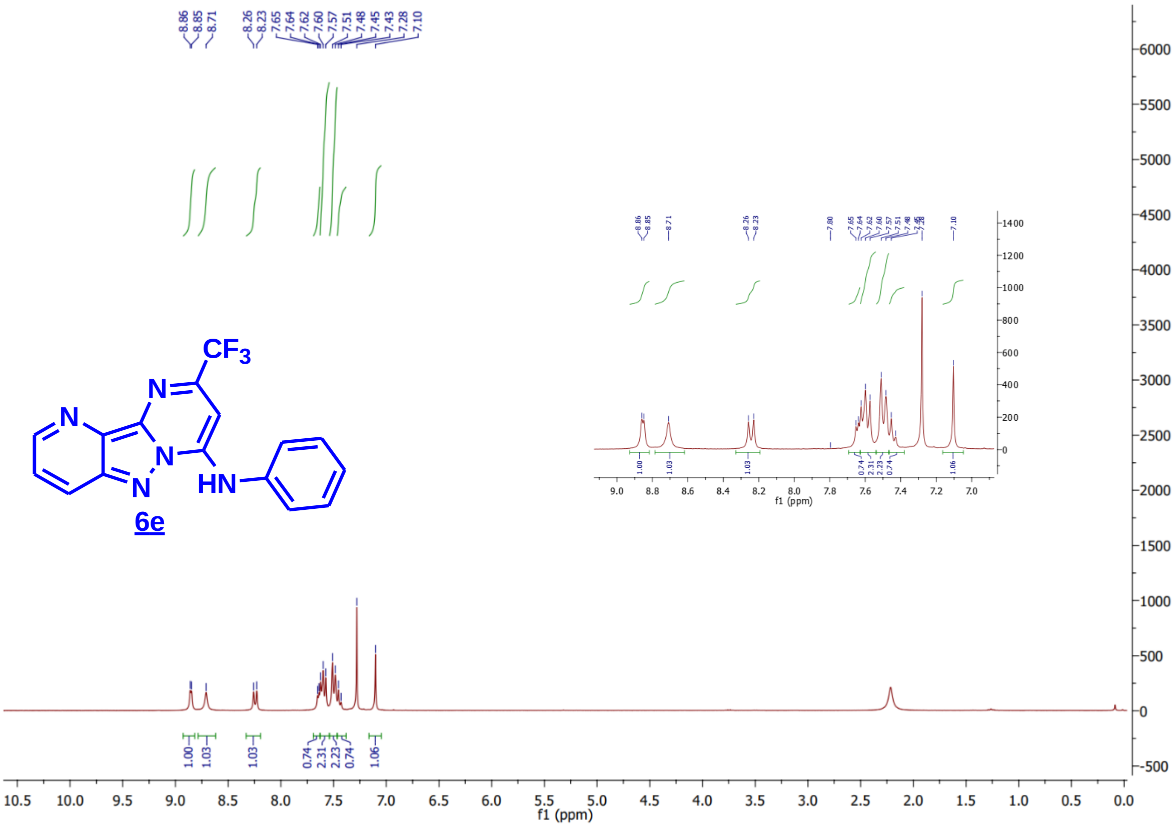

^{19}F NMR (282 MHz,CDCl$_3$)

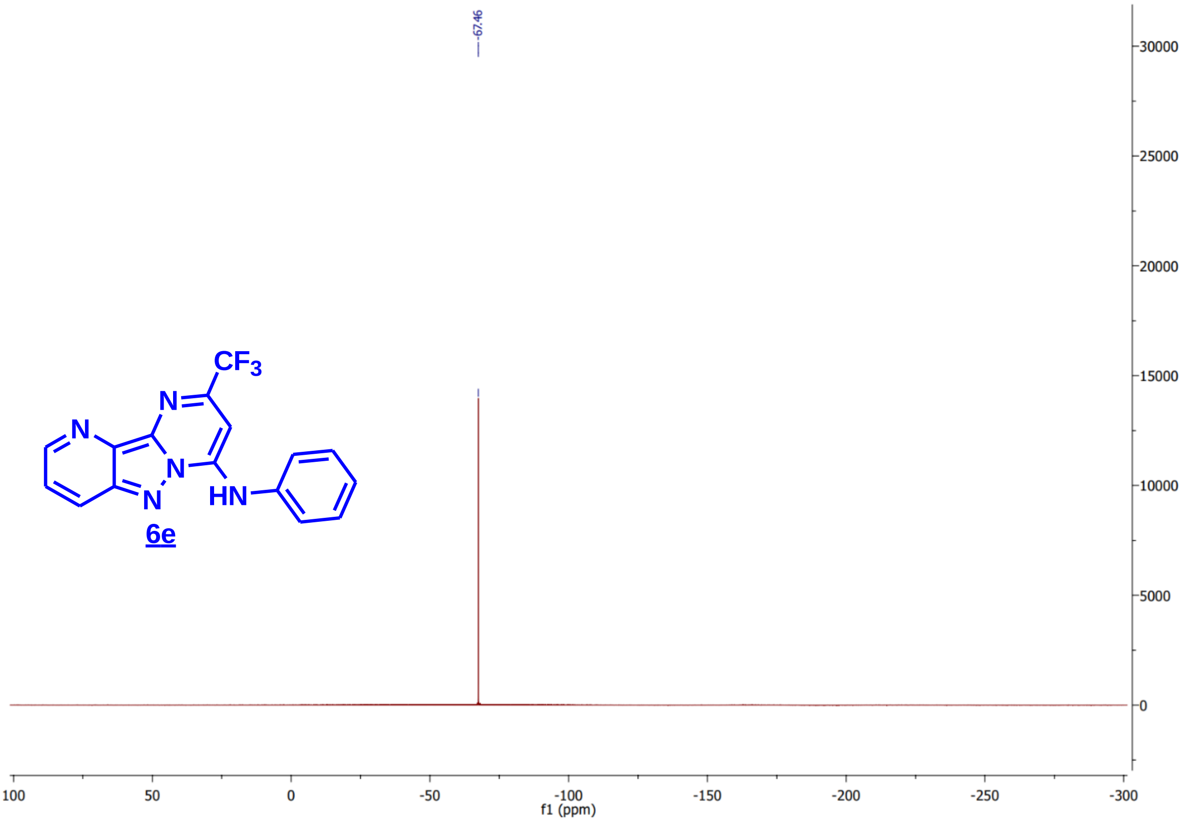

^{13}C NMR (75 MHz, CDCl$_3$)

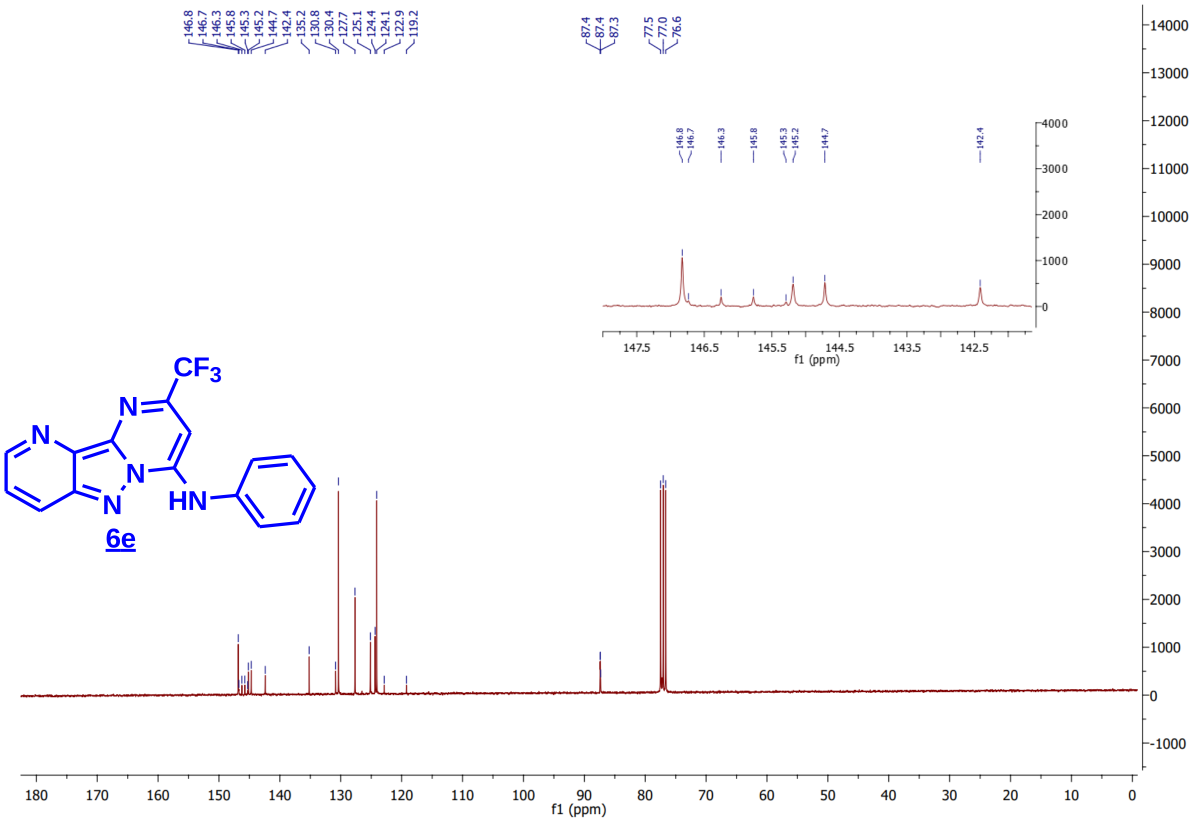

_N_-(4-methylphenyl)-9-(trifluoromethyl)pyrido[3',2':3,4]pyrazolo[1,5-_a_]pyrimidin-7-amine (6f)

^{1}H NMR (300 MHz, $CDCl_3$)

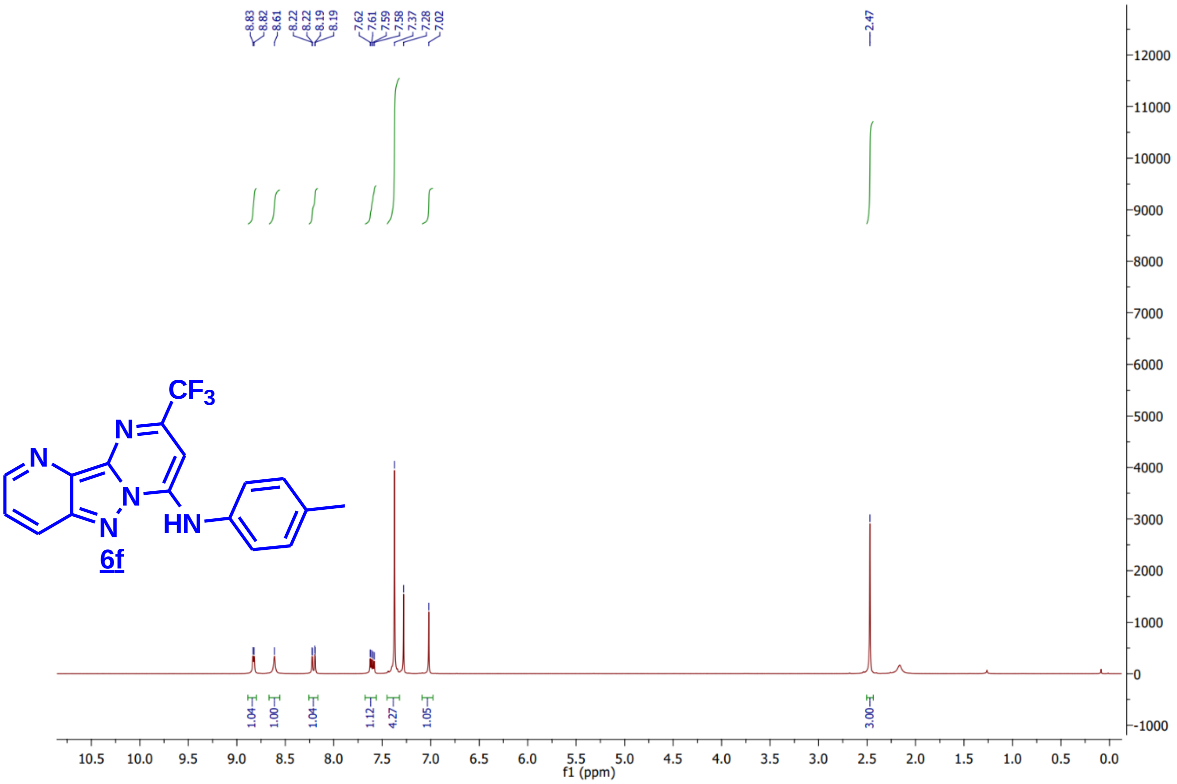

^{19}F NMR (282 MHz,$CDCl_3$)

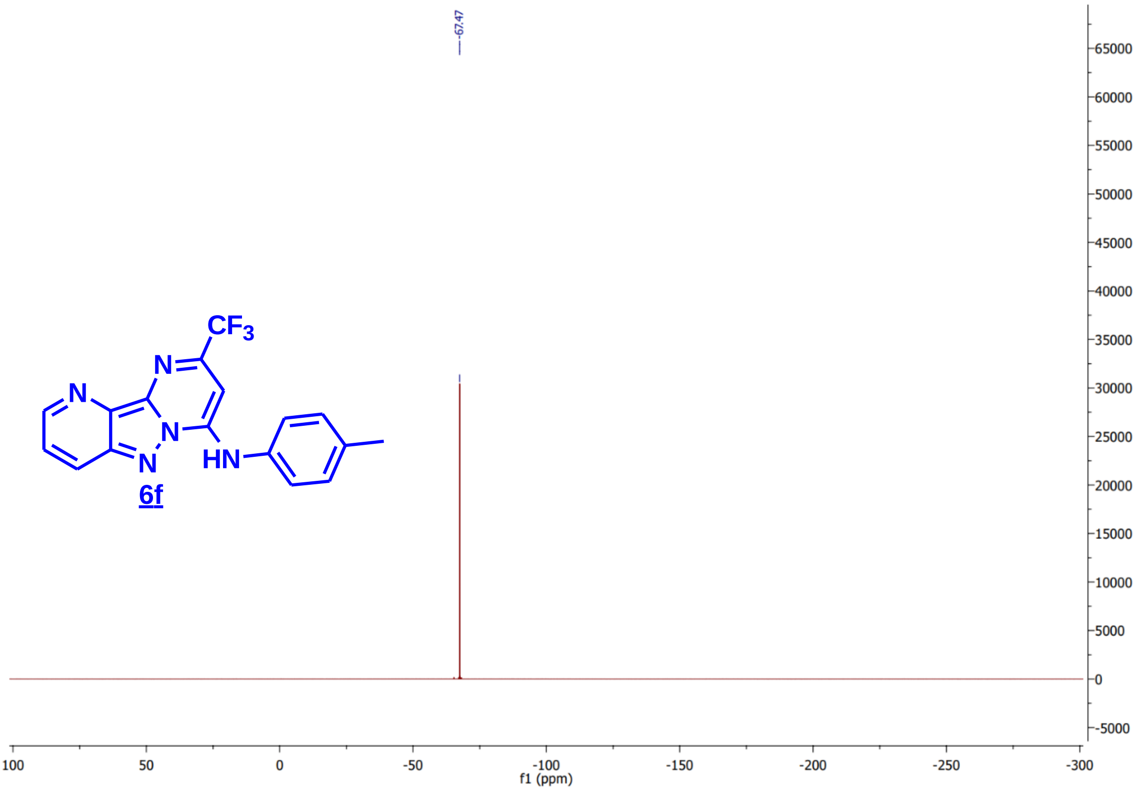

^{13}C NMR (75 MHz, $CDCl_3$)

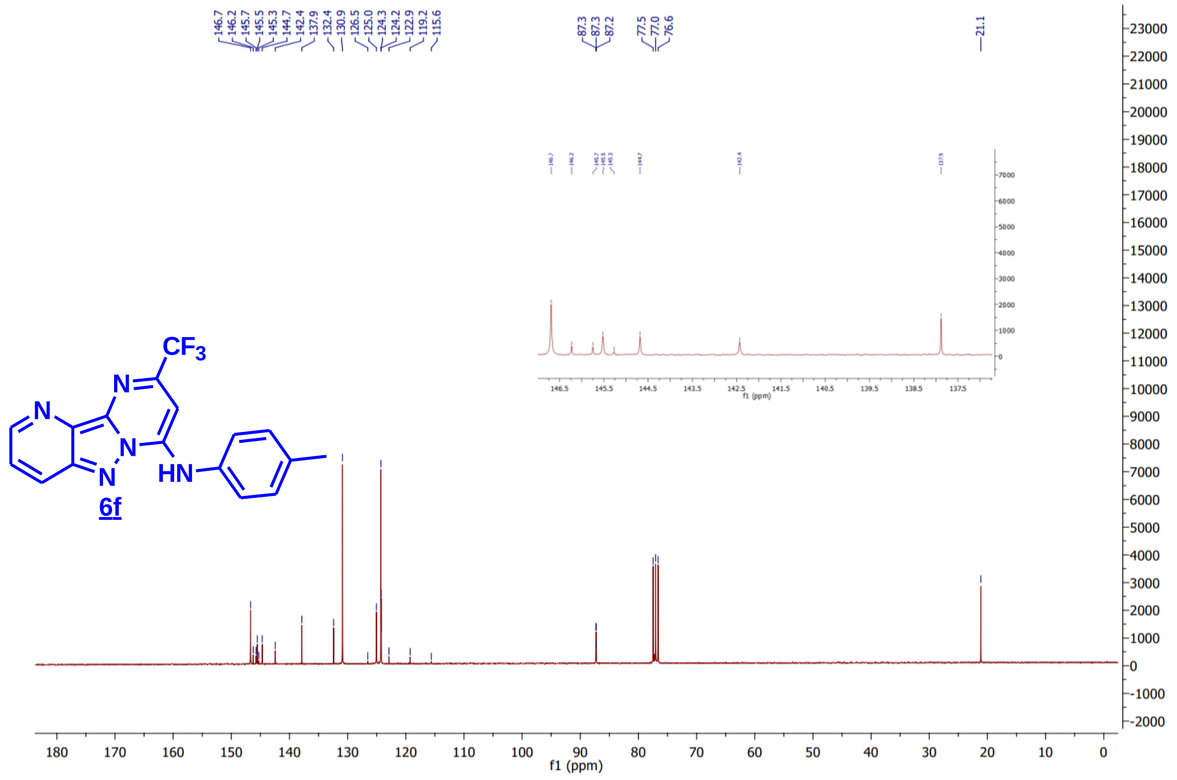

***N*-(4-bromophenyl)-9-(trifluoromethyl)pyrido[3',2':3,4]pyrazolo[1,5-*a*]pyrimidin-7-amine (6g)**

^{1}H NMR (300 MHz, $CDCl_3$)

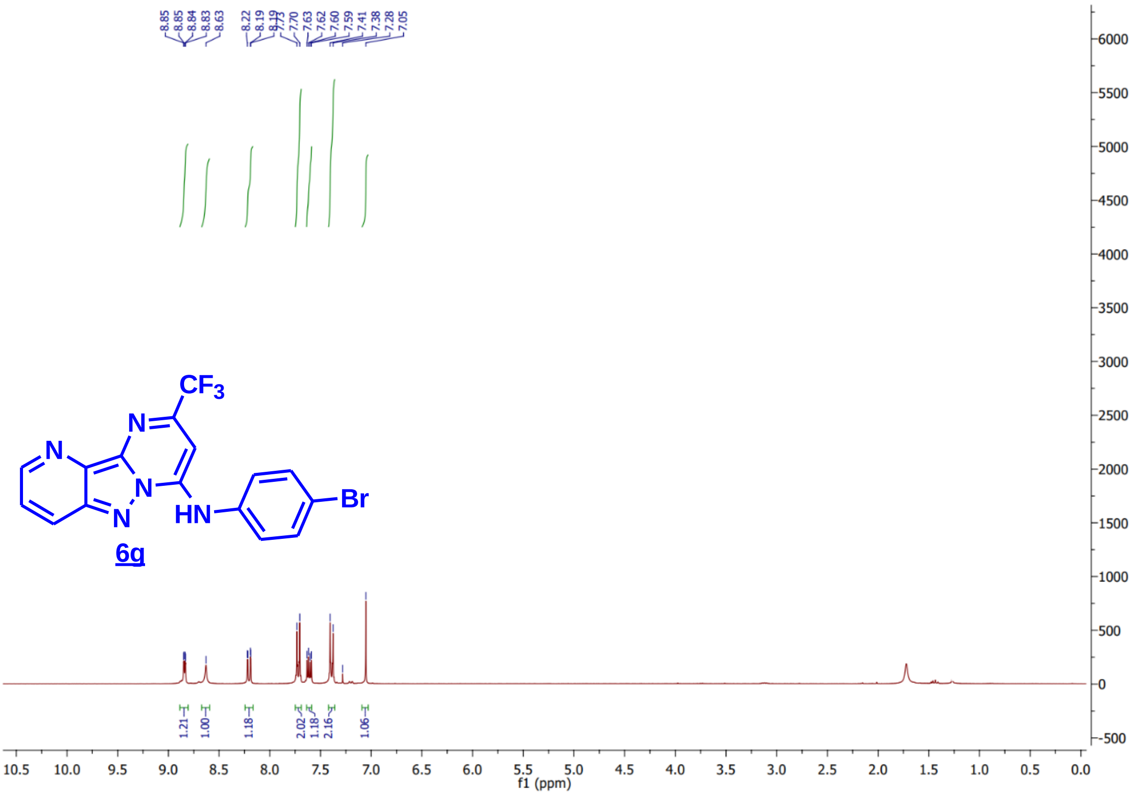

^{19}F NMR (282 MHz,$CDCl_3$)

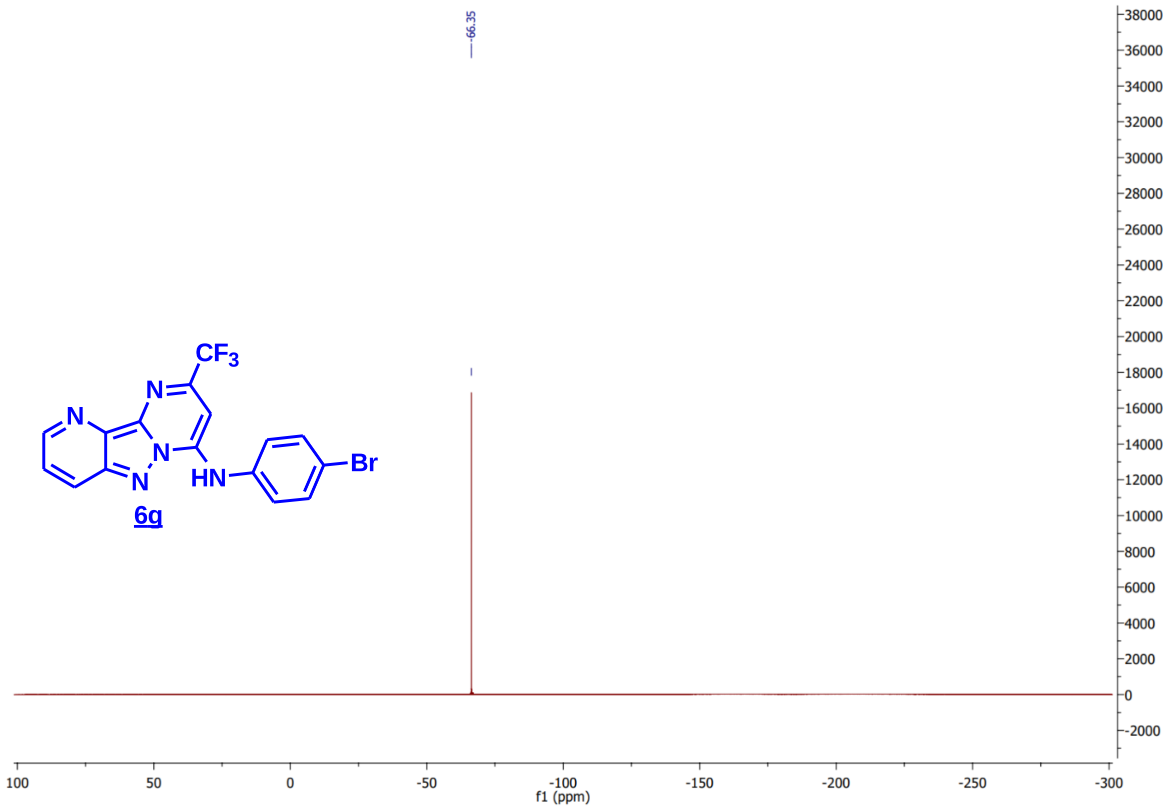

^{13}C NMR (75 MHz, DMSO-d_6)

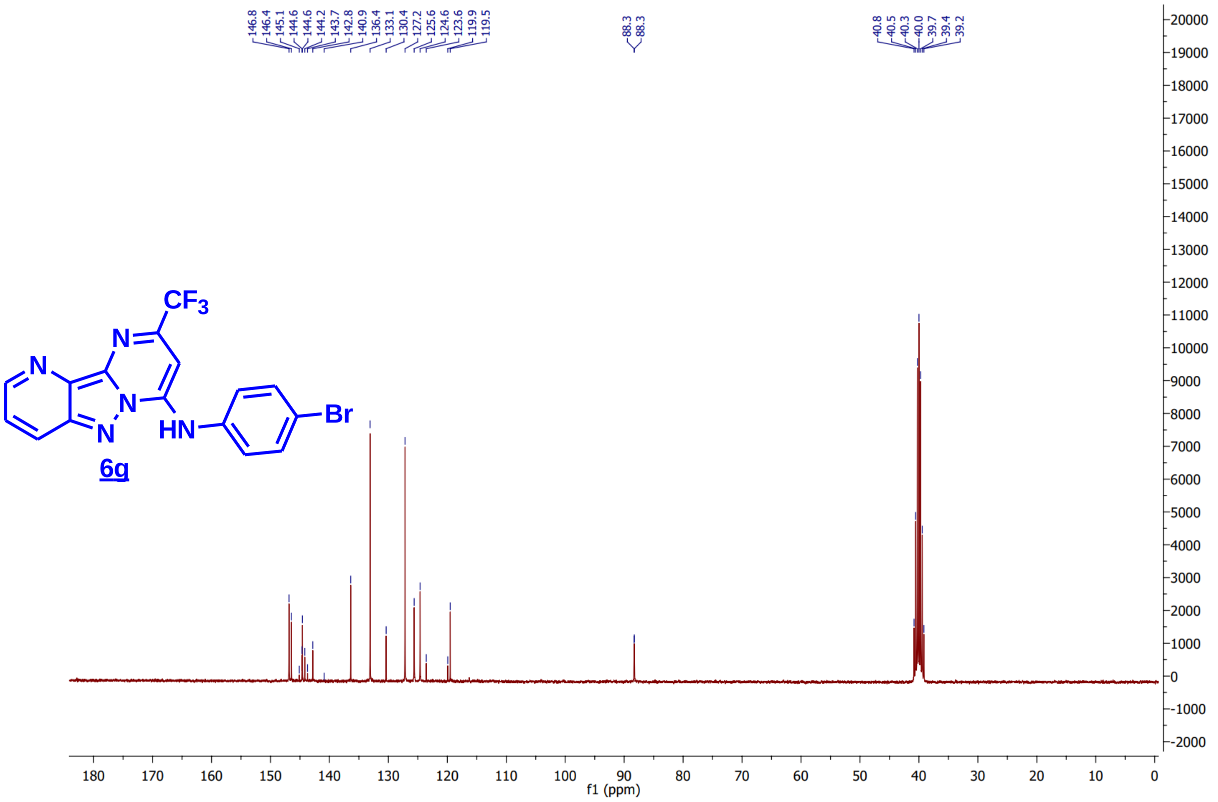

Ethyl-{[2-(trifluoromethyl)pyrido[2',3':3,4]pyrazolo[1,5-*a*]pyrimidin-4-yl]sulfanyl}acetate (7a)

^{1}H NMR (300 MHz, DMSO-d$_6$)

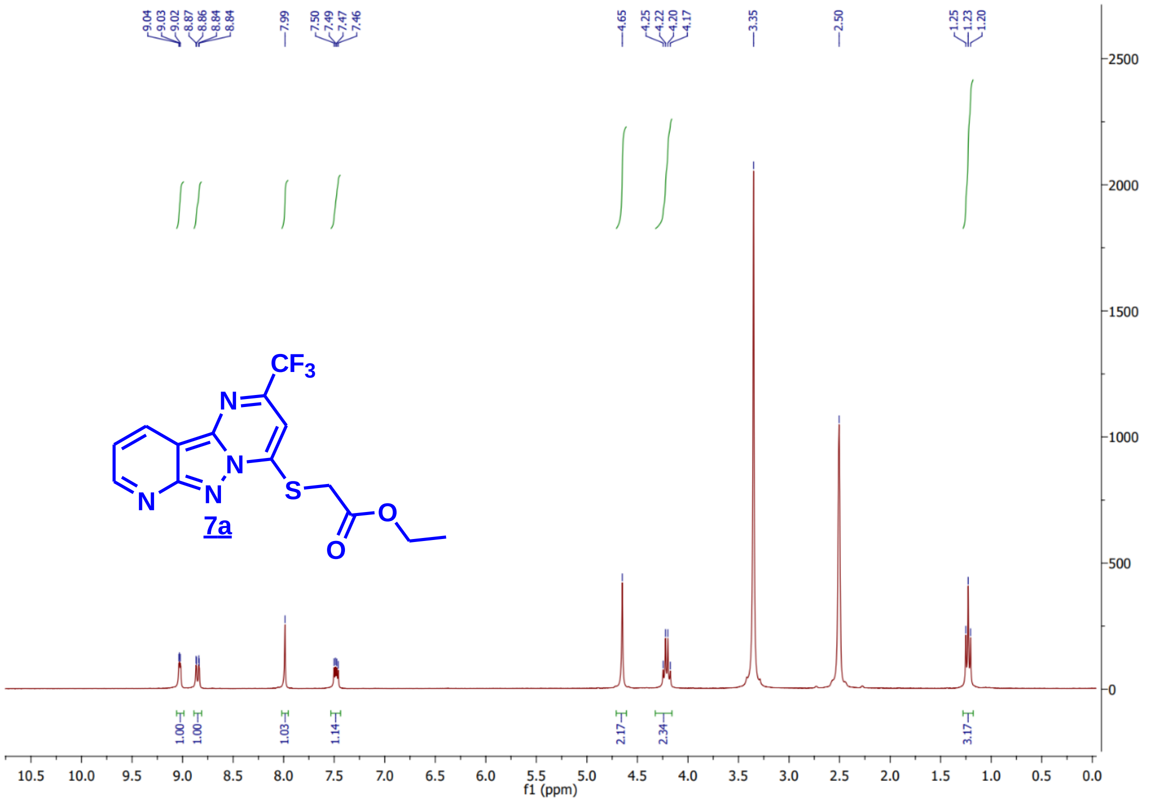

^{19}F NMR (282 MHz, DMSO-d$_6$)

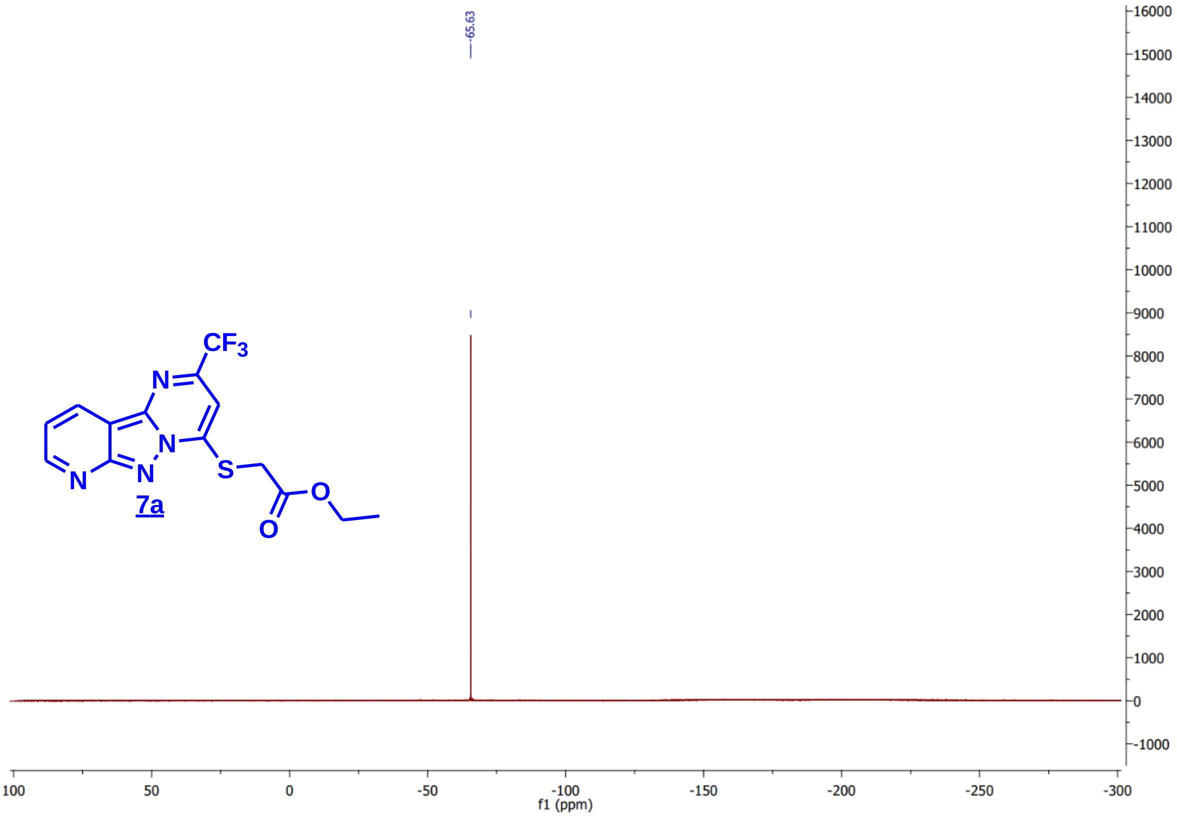

^{13}C NMR (75 MHz, DMSO-d$_6$)

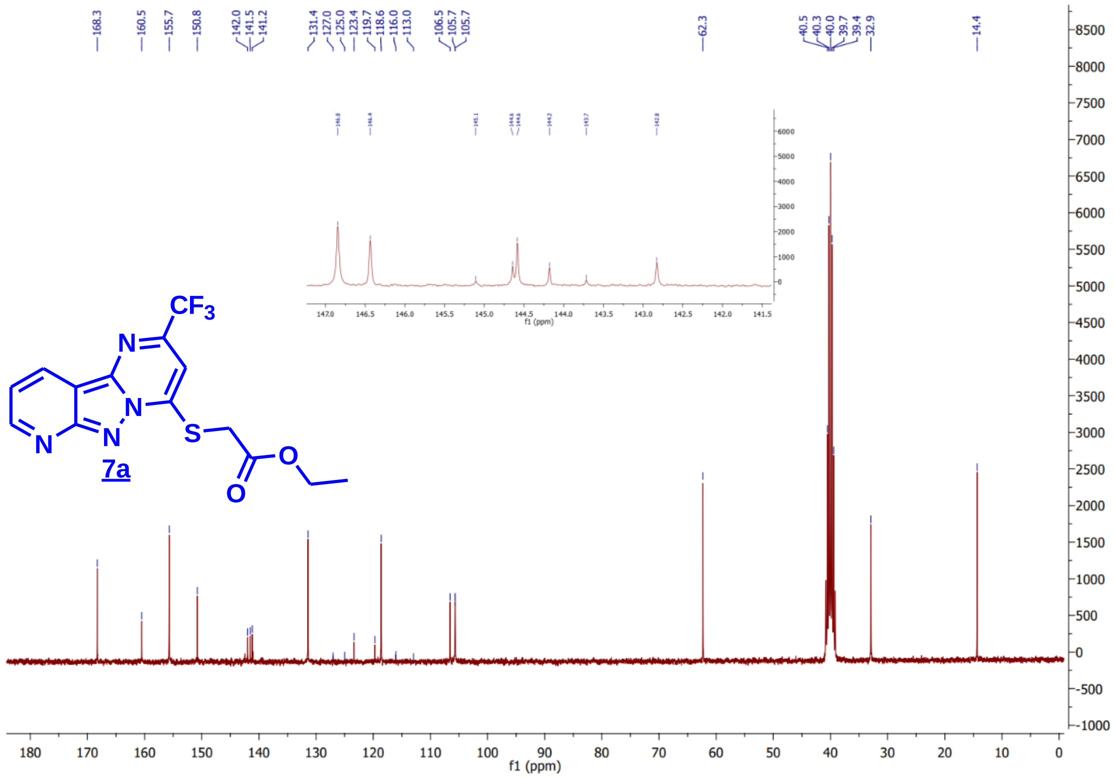

4-[(4-methoxyphenyl)sulfanyl]-2-(trifluoromethyl)pyrido[2',3':3,4]pyrazolo[1,5-*a*]pyrimidine (7b)

^{1}H NMR (300 MHz, $CDCl_3$)

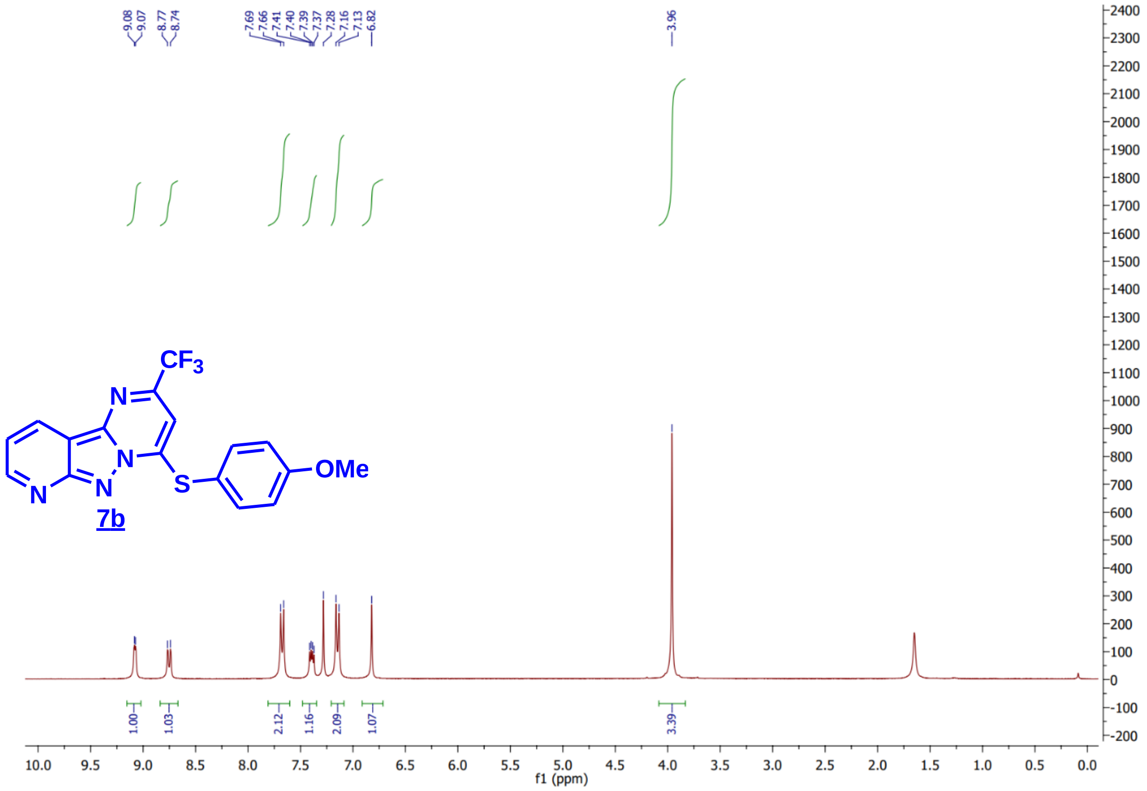

^{19}F NMR (282 MHz, $CDCl_3$)

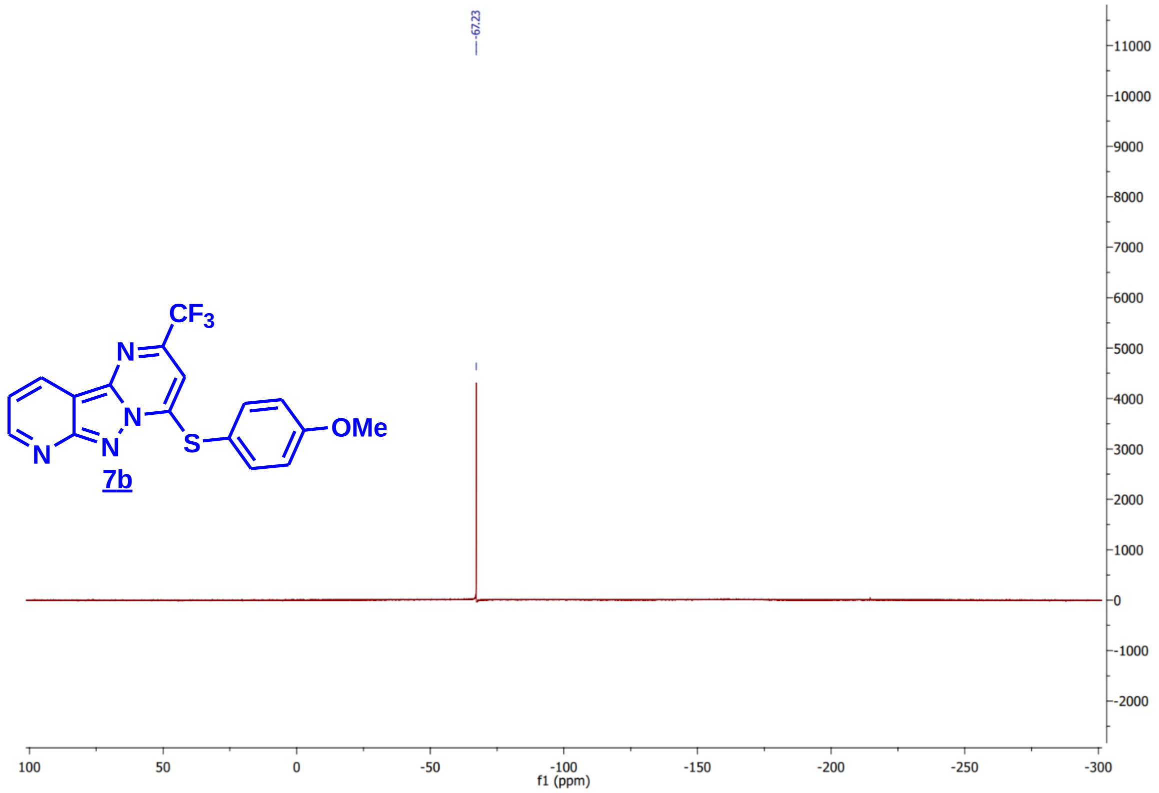

^{13}C NMR (75 MHz, $CDCl_3$)

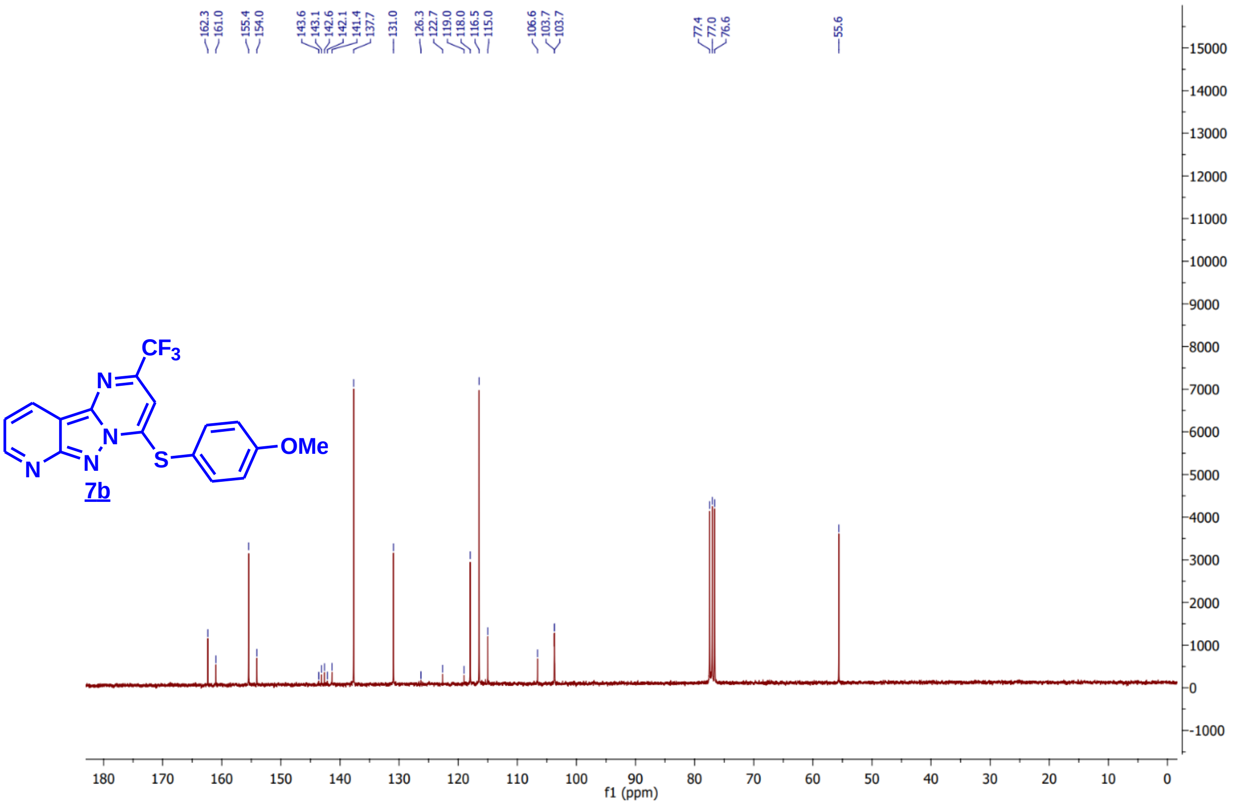

4-[(4-fluorophenyl)sulfanyl]-2-(trifluoromethyl)pyrido[2',3':3,4]pyrazolo[1,5-*a*]pyrimidine (7c)

^{1}H NMR (300 MHz, DMSO-d$_6$)

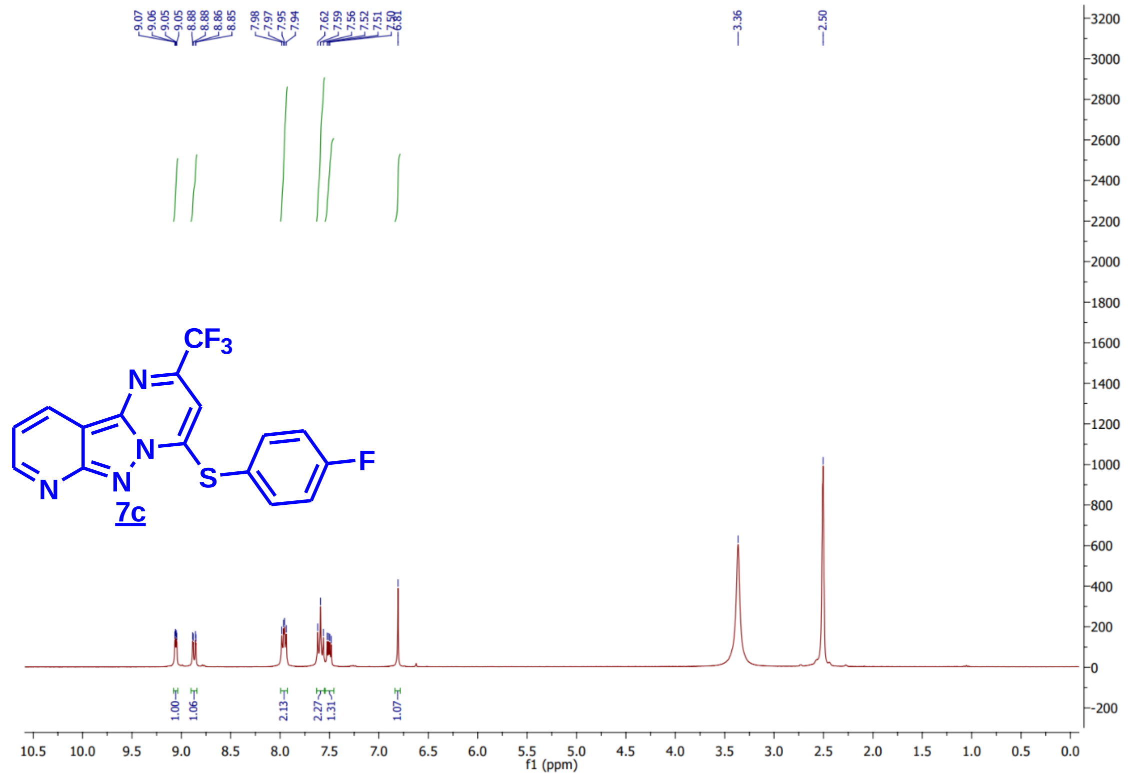

^{19}F NMR (282 MHz, DMSO-d$_6$)

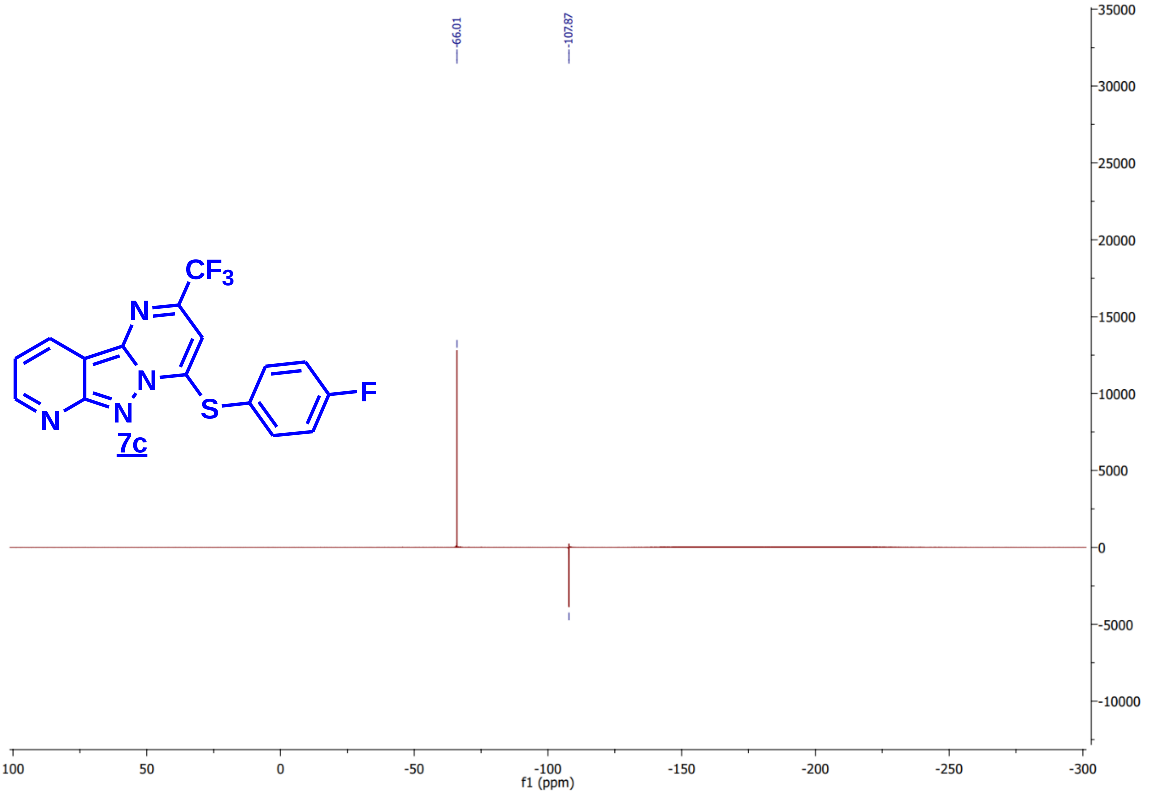

^{13}C NMR (75 MHz, DMSO-d$_6$)

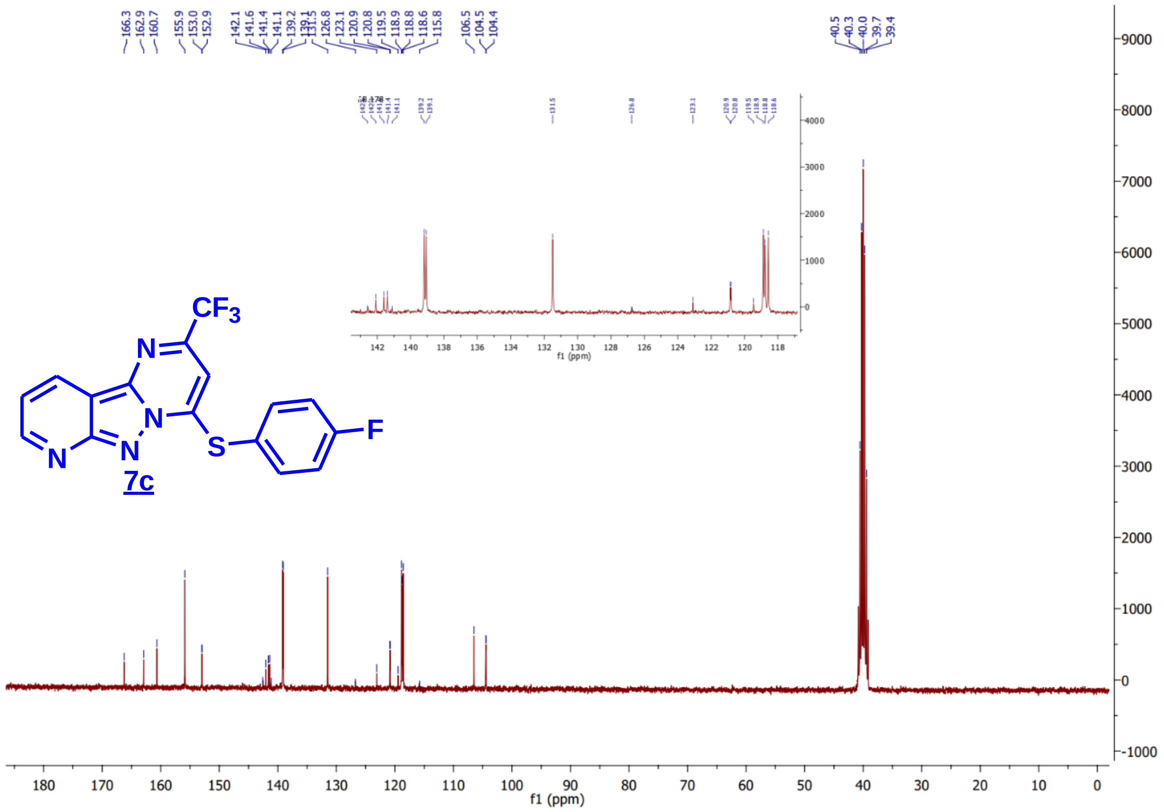

7-ethoxy-9-(trifluoromethyl)pyrido[3',2':3,4]pyrazolo[1,5-*a*]pyrimidine (8a)

^{1}H NMR (300 MHz, $CDCl_3$)

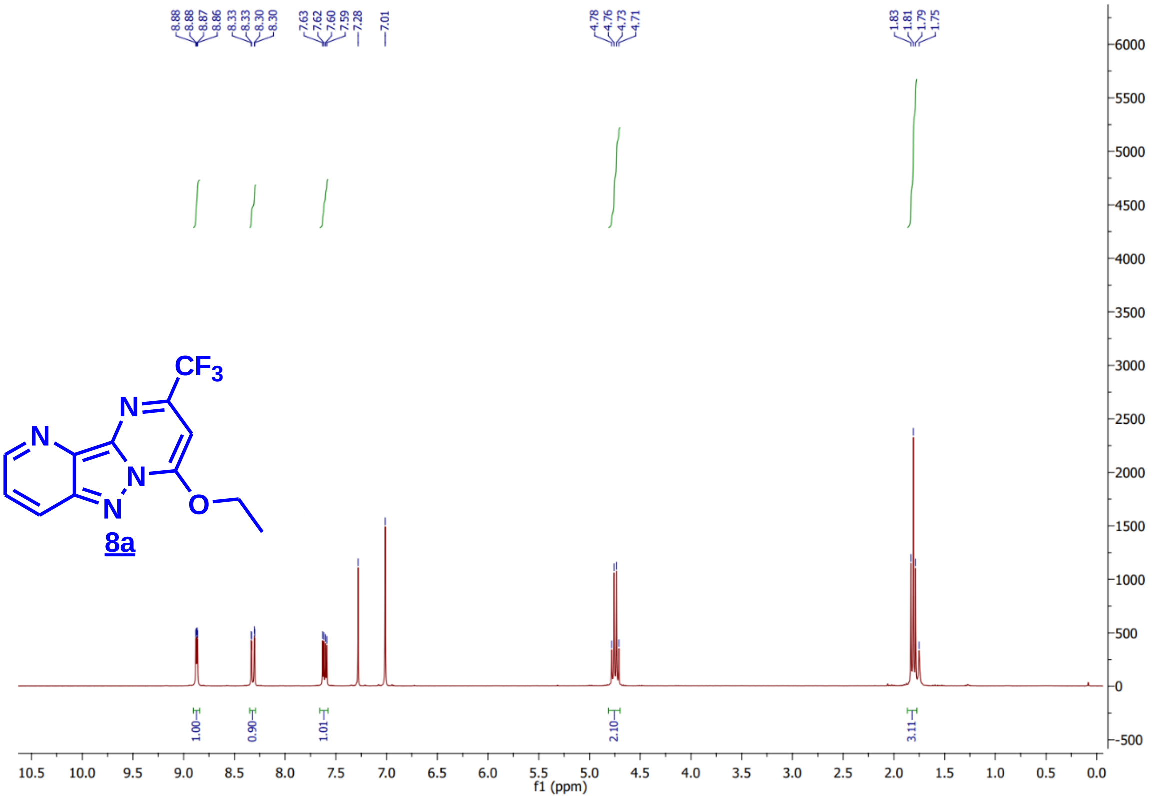

^{19}F NMR (282 MHz, CDCl3)

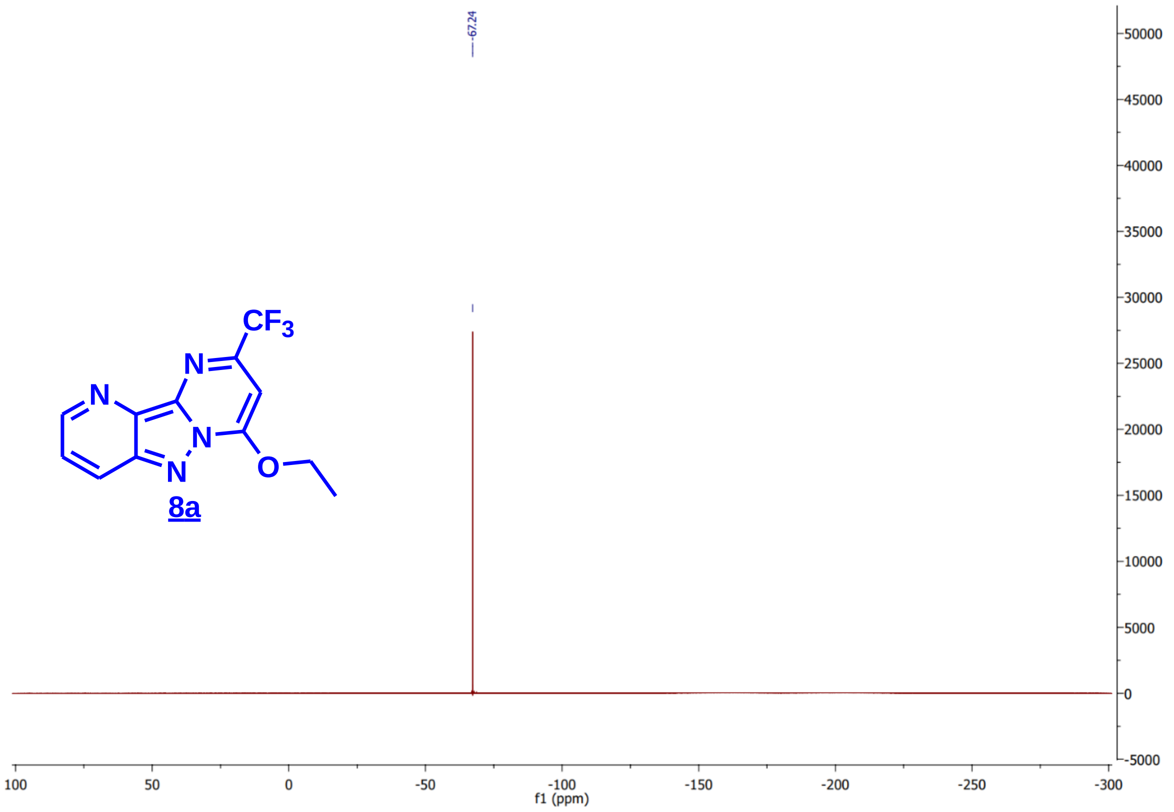

^{13}C NMR (75 MHz, CDCl3)

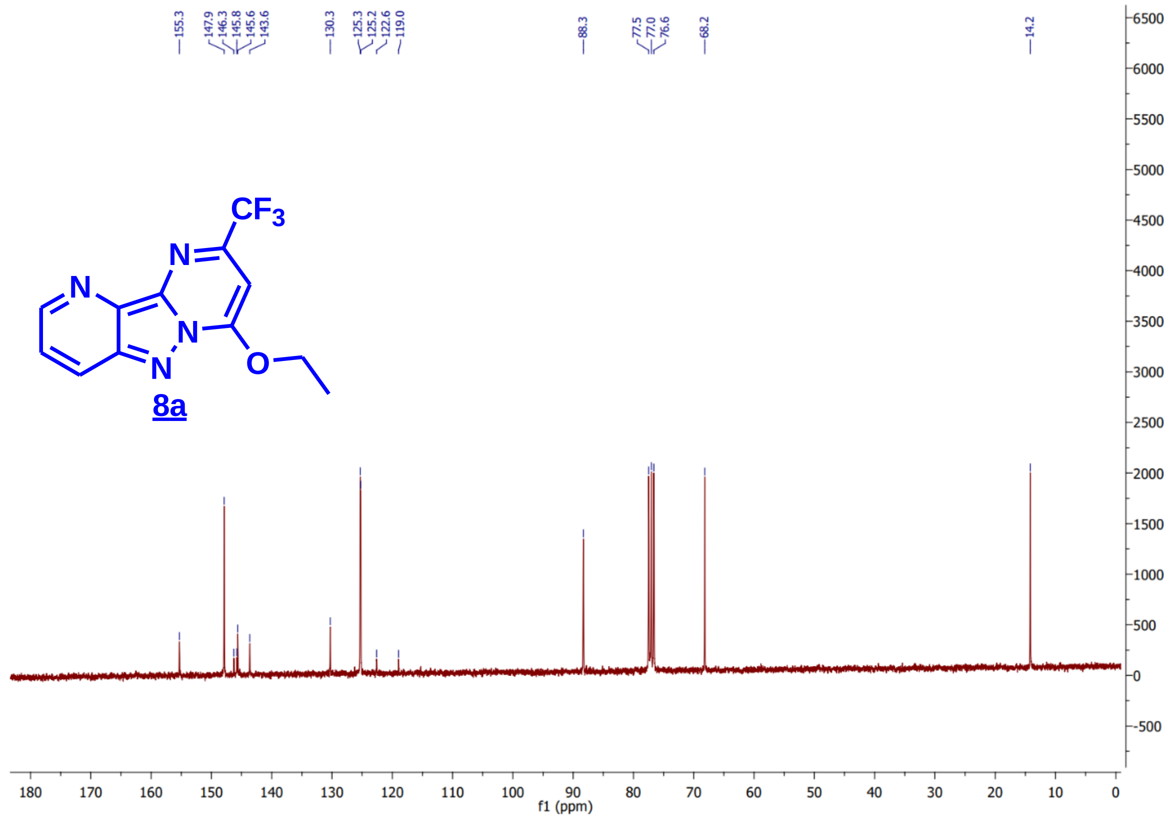

7-methoxy-9-(trifluoromethyl)pyrazino[2',3':3,4]pyrazolo[1,5-*a*]pyrimidine (8b)

^{1}H NMR (300 MHz, $CDCl_3$)

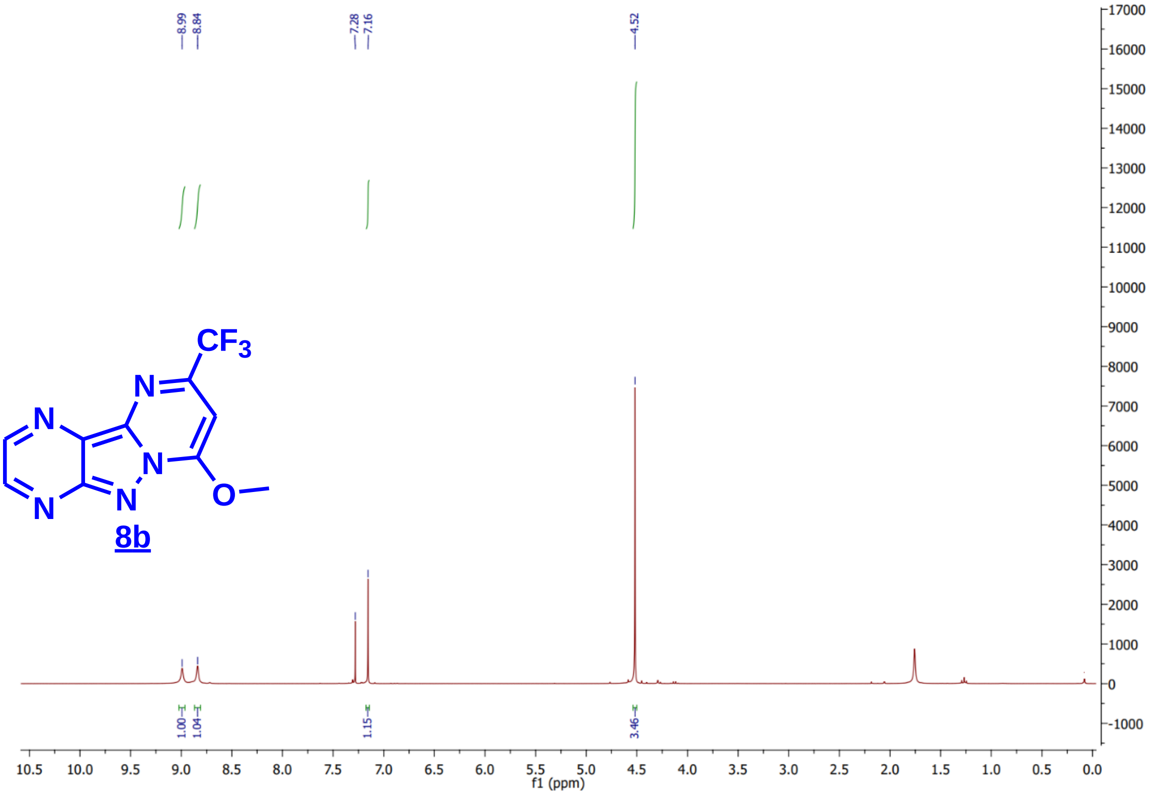

^{19}F NMR (282 MHz, $CDCl_3$)

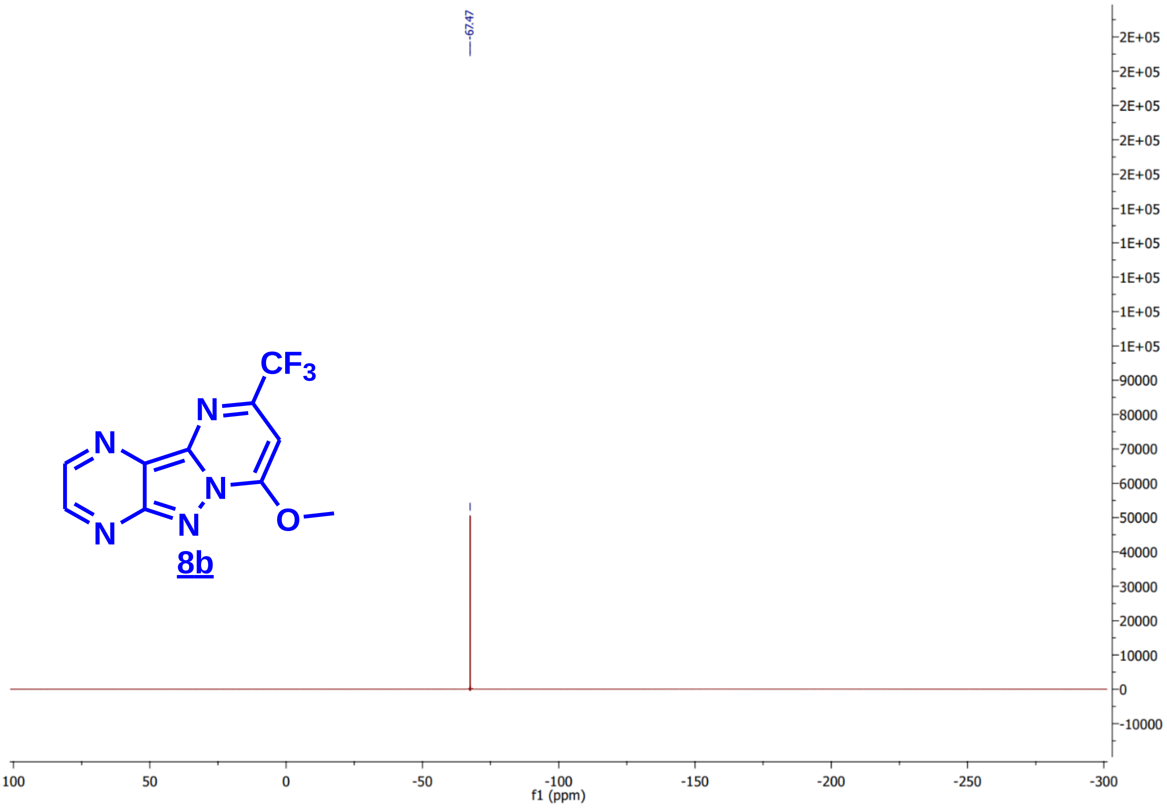

^{13}C NMR (75 MHz, $CDCl_3$)

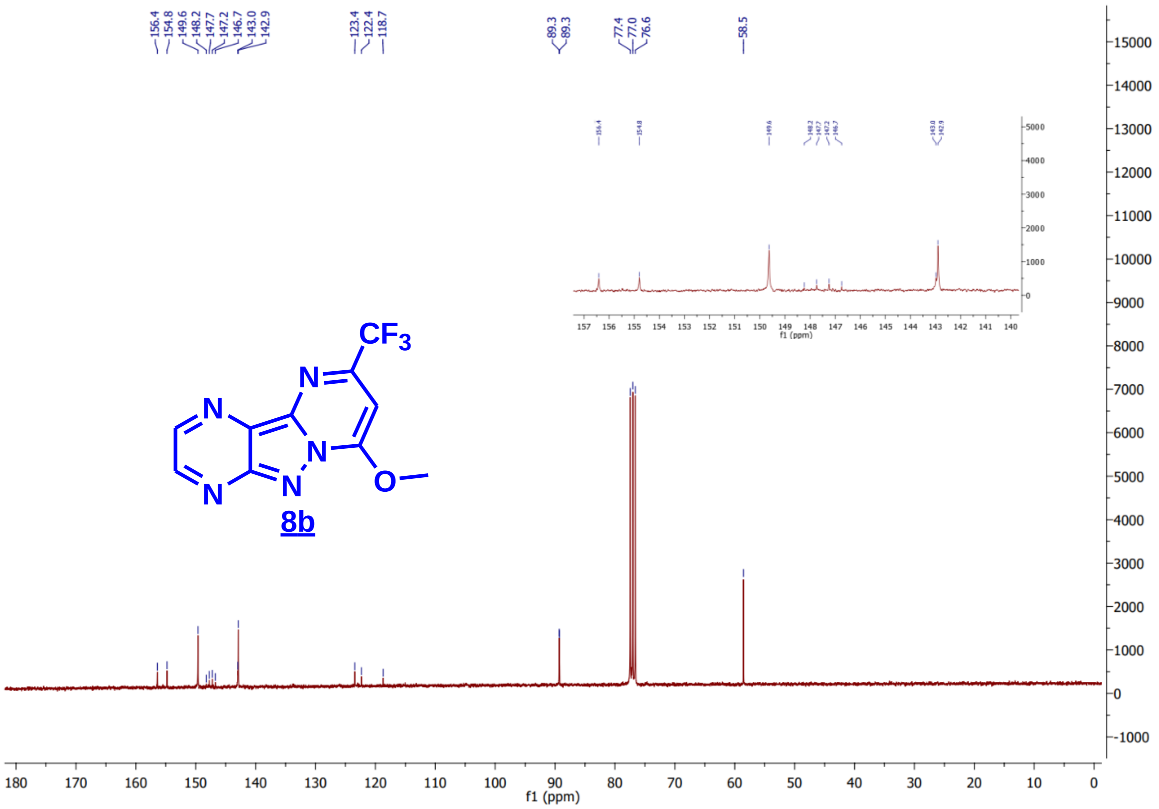

7-phenoxy-9-(trifluoromethyl)pyrazino[2',3':3,4]pyrazolo[1,5-*a*]pyrimidine (8c)

^{1}H NMR (300 MHz, $CDCl_3$)

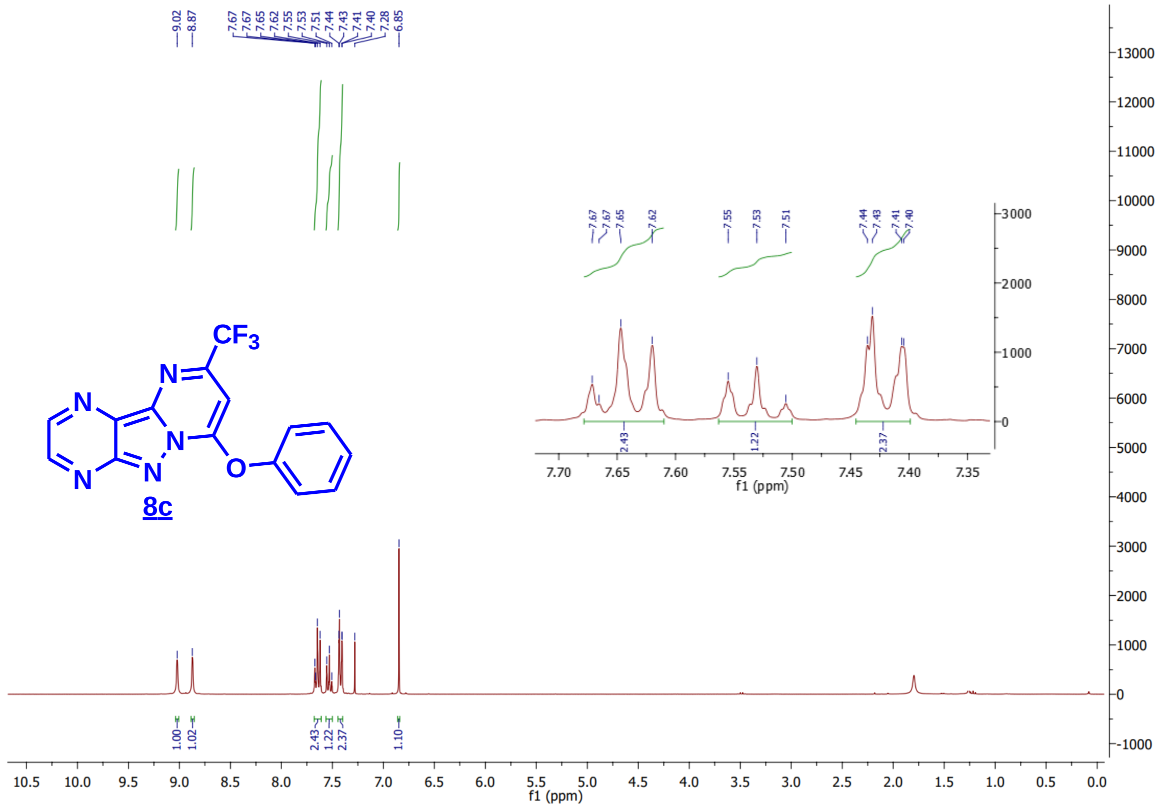

^{19}F NMR (282 MHz, $CDCl_3$)

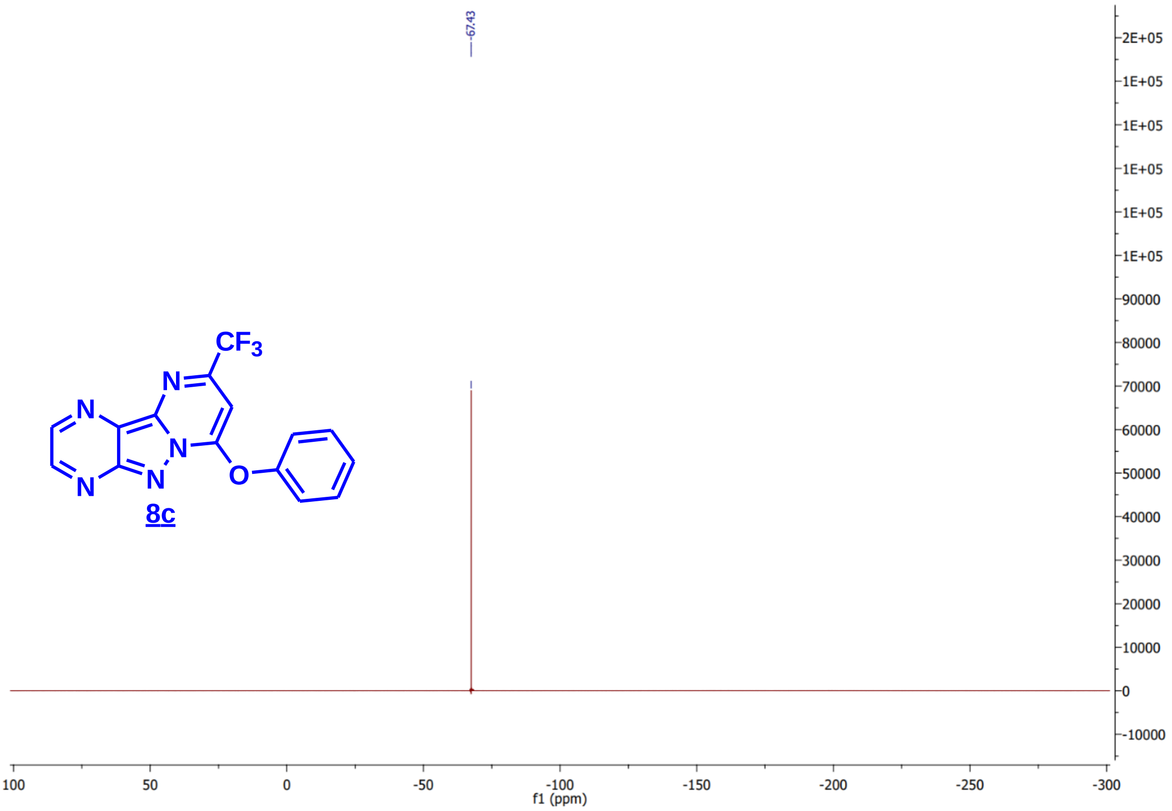

^{13}C NMR (75 MHz, $CDCl_3$)

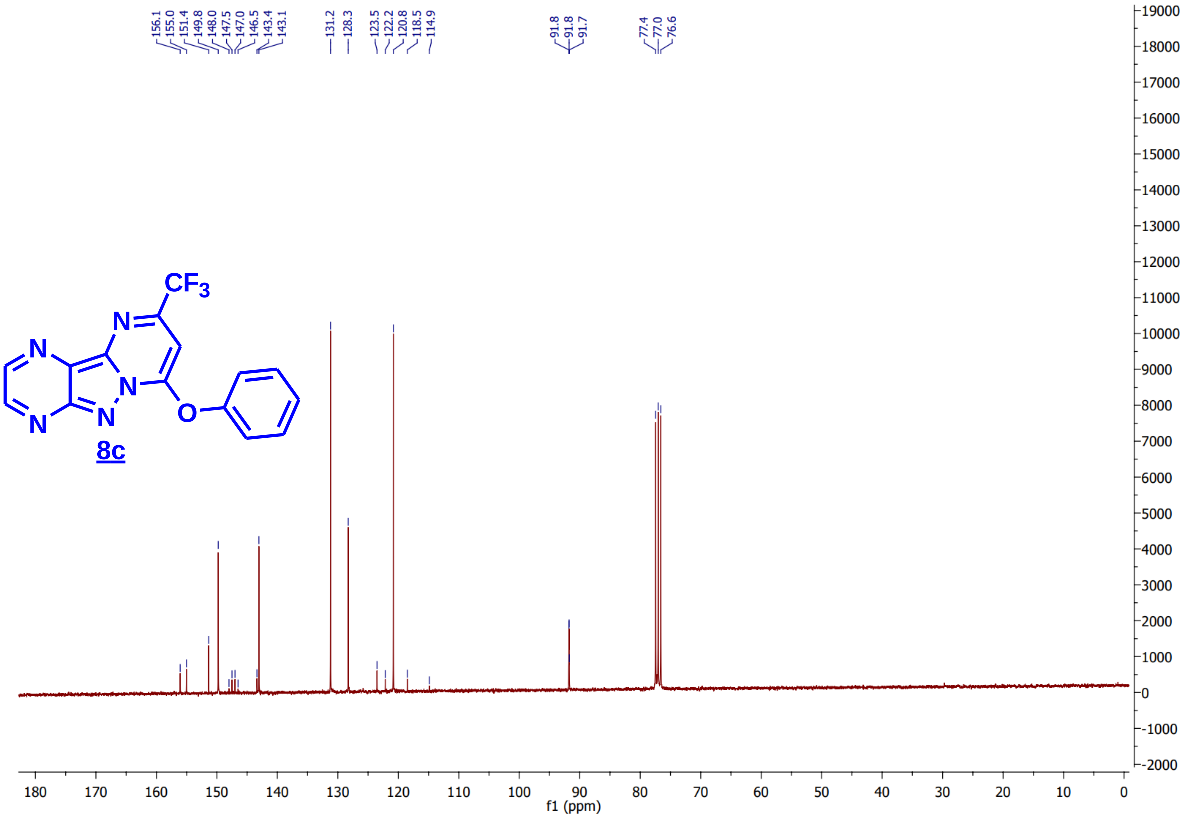

Supplement: Supplementary file 1 [file molecules-29-00044-s001.zip › molecules-2769145-supplementary.pdf]
